# Supplementary material for: Elucidating the path to Plasmodium prolyl-tRNA synthetase inhibitors that overcome halofuginone resistance
Source: Nat Commun. 2022 Aug 25;13:4976. doi: 10.1038/s41467-022-32630-4 (PMC9403976; doi:10.1038/s41467-022-32630-4)
Supplement: Supplementary file 1 — Supplementary Information [file 41467_2022_32630_MOESM1_ESM.pdf]

Supplementary Information

Elucidating the path to *Plasmodium* prolyl-tRNA synthetase inhibitors that overcome halofuginone-resistance

Authors

Mark A. Tye[1,2,3], N. Connor Payne[1,4‡], Catrine Johansson[5,6,‡], Kritika Singh[1,7], Sofia A. Santos[1], Lọla Fagbami[1,2,3,8], Akansha Pant[3], Kayla Sylvester[9], Madeline R. Luth[10], Sofia Marques[11], Malcolm Whitman[12], Maria M. Mota[11], Elizabeth A. Winzeler[10], Amanda K. Lukens[8], Emily R. Derbyshire[9], Udo Oppermann[5,6], Dyann F. Wirth[3,8], Ralph Mazitschek[1,3,8*].

Affiliations

[1]Center for Systems Biology, Massachusetts General Hospital, Boston, MA, USA

[2]Harvard Graduate School of Arts and Sciences, Cambridge, MA, USA

[3]Harvard T.H. Chan School of Public Health, Boston, MA, USA

[4]Department of Chemistry & Chemical Biology, Harvard University, Cambridge, MA, USA

[5]Botnar Research Centre, NIHR Oxford Biomedical Research Unit, University of Oxford, Oxford, UK

[6]Centre for Medicines Discovery, University of Oxford, UK

[7]Department of Bioengineering, Northeastern University; Boston, MA, USA

[8]Broad Institute of MIT and Harvard, Cambridge, MA, USA

[9]Department of Chemistry, Duke University, Durham, NC, USA

[10]Department of Pediatrics; University of California, San Diego; La Jolla, CA, USA

[11]Instituto de Medicina Molecular, Faculdade de Medicina, Universidade de Lisboa, Lisbon, Portugal

[12]Department of Developmental Biology, Harvard School of Dental Medicine, Boston, MA, USA

* Corresponding author: Ralph Mazitschek <ralph@broadinstitute.org>

‡ contributed equally

Table of Contents:

I. Supplementary Figures

1. Supplementary Figure 1. Comparison of non-conserved ProRS residues adjacent to the active site.
2. Supplementary Figure 2. Asexual blood stage *P. falciparum* activity of ATP-site targeted pyrazinamide-derived ProRS ligands.
3. Supplementary Figure 3. Additional chemical structures.
4. Supplementary Figure 4. Additional characterization of TR-FRET tracers MAT379 (**24**) and MAT425
5. Supplementary Figure 5. ProRS substrate affinity determination.
6. Supplementary Figure 6. TR-FRET-based assay data for *Pf*cProRS in the absence of substrates – Set 1.
7. Supplementary Figure 7. TR-FRET-based assay data for *Pf*cProRS in the absence of substrates – Set 2.
8. Supplementary Figure 8. TR-FRET-based assay data for *Pf*cProRS in the presence of proline – Set 1.
9. Supplementary Figure 9. TR-FRET-based assay data for *Pf*cProRS in the presence of proline – Set 2.
10. Supplementary Figure 10. TR-FRET-based assay data for *Pf*cProRS in the presence of ATP.
11. Supplementary Figure 11. TR-FRET-based assay data for *Hs*ProRS in the absence of substrates – Set 1.
12. Supplementary Figure 12. TR-FRET-based assay data for *Hs*ProRS in the absence of substrates – Set 2.
13. Supplementary Figure 13. TR-FRET-based assay data for *Hs*ProRS in the presence of proline – Set 1.
14. Supplementary Figure 14. TR-FRET-based assay data for *Hs*ProRS in the presence of proline – Set 2.
15. Supplementary Figure 15. TR-FRET-based assay data for *Hs*ProRS in the presence of ATP.
16. Supplementary Figure 16. Correlation between TR-FRET pK_D values and *P. falciparum* asexual blood stage growth assay pEC_{50} values.
17. Supplementary Figure 17. Characterization of dual-site ligands binding *Hs*ProRS in the absence or presence of proline.
18. Supplementary Figure 18. Structural comparison of free and ligand-bound *Hs*ProRS.
19. Supplementary Figure 19. Additional characterization of triple-site ligands binding ProRS.
20. Supplementary Figure 20. ^{1}H NMR of compound **45**.
21. Supplementary Figure 21. ^{13}C NMR of compound **45**.
22. Supplementary Figure 22. ^{1}H NMR of compound **2**.
23. Supplementary Figure 23. ^{13}C NMR of compound **2**.
24. Supplementary Figure 24. ^{1}H NMR of compound NCP26.
25. Supplementary Figure 25. ^{13}C NMR of compound NCP26.
26. Supplementary Figure 26. ^{1}H NMR of compound **4**.
27. Supplementary Figure 27. ^{13}C NMR of compound **4**.
28. Supplementary Figure 28. ^{1}H NMR of compound **5**.
29. Supplementary Figure 29. ^{13}C NMR of compound **5**.
30. Supplementary Figure 30. ^{1}H NMR of compound **6**.
31. Supplementary Figure 31. ^{13}C NMR of compound **6**.
32. Supplementary Figure 32. ^{1}H NMR of compound **7**.

33. Supplementary Figure 33. ^{13}C NMR of compound **7**.
34. Supplementary Figure 34. ^{1}H NMR of compound **8**.
35. Supplementary Figure 35. ^{13}C NMR of compound **8**.
36. Supplementary Figure 36. ^{1}H NMR of compound **9**.
37. Supplementary Figure 37. ^{13}C NMR of compound **9**.
38. Supplementary Figure 38. ^{1}H NMR of compound **10**.
39. Supplementary Figure 39. ^{13}C NMR of compound **10**.
40. Supplementary Figure 40. ^{1}H NMR of compound **11**.
41. Supplementary Figure 41. ^{13}C NMR of compound **11**.
42. Supplementary Figure 42. ^{1}H NMR of compound **12**.
43. Supplementary Figure 43. ^{13}C NMR of compound **12**.
44. Supplementary Figure 44. ^{1}H NMR of compound **13**.
45. Supplementary Figure 45. ^{13}C NMR of compound **13**.
46. Supplementary Figure 46. ^{1}H NMR of compound **14**.
47. Supplementary Figure 47. ^{13}C NMR of compound **14**.
48. Supplementary Figure 48. ^{1}H NMR of compound **15**.
49. Supplementary Figure 49. ^{13}C NMR of compound **15**.
50. Supplementary Figure 50. ^{1}H NMR of compound **16**.
51. Supplementary Figure 51. ^{13}C NMR of compound **16**.
52. Supplementary Figure 52. DEPT-135 NMR of compound **16**.
53. Supplementary Figure 53. ^{1}H-^{1}H COSY NMR of compound **16**.
54. Supplementary Figure 54. ^{1}H-^{13}C HMBC NMR of compound **16**.
55. Supplementary Figure 55. ^{1}H-^{13}C HSQC NMR of compound **16**.
56. Supplementary Figure 56. ^{1}H-^{1}H NOESY NMR of compound **16**.
57. Supplementary Figure 57. ^{1}H NMR of compound **17**.
58. Supplementary Figure 58. ^{13}C NMR of compound **17**.
59. Supplementary Figure 59. ^{1}H NMR of compound **18**.
60. Supplementary Figure 60. ^{13}C NMR of compound **18**.
61. Supplementary Figure 61. ^{1}H NMR of compound **19** in $CDCl_3$.
62. Supplementary Figure 62. ^{13}C NMR of compound **19** in $CDCl_3$.
63. Supplementary Figure 63. ^{1}H NMR of compound **19** in d_6DMSO.
64. Supplementary Figure 64. ^{1}H NMR of compound **20**.
65. Supplementary Figure 65. ^{13}C NMR of compound **20**.
66. Supplementary Figure 66. ^{1}H NMR of compound **21**.
67. Supplementary Figure 67. ^{13}C NMR of compound **21**.
68. Supplementary Figure 68. ^{1}H NMR of compound **22**.
69. Supplementary Figure 69. ^{13}C NMR of compound **22**.
70. Supplementary Figure 70. ^{1}H NMR of compound **23**.
71. Supplementary Figure 71. ^{13}C NMR of compound **23**.
72. Supplementary Figure 72. ^{1}H NMR of compound **41**.
73. Supplementary Figure 73. ^{13}C NMR of compound **41**.
74. Supplementary Figure 74. ^{1}H NMR of compound **42**.
75. Supplementary Figure 75. ^{13}C NMR of compound **42**.
76. Supplementary Figure 76. ^{1}H NMR of compound MAT379.
77. Supplementary Figure 77. ^{13}C NMR of compound MAT379.
78. Supplementary Figure 78. DEPT-135 NMR of compound MAT379.
79. Supplementary Figure 79. ^{1}H-^{1}H COSY NMR of compound MAT379.
80. Supplementary Figure 80. ^{1}H-^{13}C HMBC NMR of compound MAT379.
81. Supplementary Figure 81. ^{1}H-^{13}C HSQC NMR of compound MAT379.
82. Supplementary Figure 82. ^{1}H NMR of compound **43**.
83. Supplementary Figure 83. ^{13}C NMR of compound **43**.
84. Supplementary Figure 84. ^{1}H NMR of compound **44**.

85. Supplementary Figure 85. ^{13}C NMR of compound **44**.
86. Supplementary Figure 86. ^{1}H NMR of compound MAT425.
87. Supplementary Figure 87. ^{1}H NMR of compound **31**.
88. Supplementary Figure 88. ^{13}C NMR of compound **31**.
89. Supplementary Figure 89. ^{1}H NMR of compound MAT334.
90. Supplementary Figure 90. ^{13}C NMR of compound MAT334.
91. Supplementary Figure 91. ^{1}H NMR of compound **32**.
92. Supplementary Figure 92. ^{13}C NMR of compound **32**.
93. Supplementary Figure 93. ^{1}H NMR of compound MAT345.
94. Supplementary Figure 94. ^{13}C NMR of compound MAT345.
95. Supplementary Figure 95. DEPT-135 NMR of compound MAT345.
96. Supplementary Figure 96. ^{1}H-^{1}H COSY NMR of compound MAT345.
97. Supplementary Figure 97. ^{1}H-^{13}C HMBC NMR of compound MAT345.
98. Supplementary Figure 98. ^{1}H-^{13}C HSQC NMR of compound MAT345.
99. Supplementary Figure 99. ^{1}H NMR of compound **54**.
100. Supplementary Figure 100. ^{13}C NMR of compound **54**.
101. Supplementary Figure 101. ^{1}H NMR of compound **37**.
102. Supplementary Figure 102. ^{13}C NMR of compound **37**.
103. Supplementary Figure 103. DEPT-135 NMR of compound **37**.
104. Supplementary Figure 104. ^{1}H-^{1}H COSY NMR of compound **37**.
105. Supplementary Figure 105. ^{1}H-^{13}C HMBC NMR of compound **37**.
106. Supplementary Figure 106. ^{1}H-^{13}C HSQC NMR of compound **37**.
107. Supplementary Figure 107. ^{1}H NMR of compound MAT436.
108. Supplementary Figure 108. ^{13}C NMR of compound MAT436.
109. Supplementary Figure 109. DEPT-135 NMR of compound MAT436.
110. Supplementary Figure 110. ^{1}H-^{1}H COSY NMR of compound MAT436.
111. Supplementary Figure 111. ^{1}H-^{13}C HMBC NMR of compound MAT436.
112. Supplementary Figure 112. ^{1}H-^{13}C HSQC NMR of compound MAT436.
113. Supplementary Figure 113. ^{1}H NMR of compound *iso*-MAT436.
114. Supplementary Figure 114. ^{13}C NMR of compound *iso*-MAT436.
115. Supplementary Figure 115. DEPT-135 NMR of compound *iso*-MAT436.
116. Supplementary Figure 116. ^{1}H-^{1}H COSY NMR of compound *iso*-MAT436.
117. Supplementary Figure 117. ^{1}H-^{13}C HMBC NMR of compound *iso*-MAT436.
118. Supplementary Figure 118. ^{1}H-^{13}C HSQC NMR of compound *iso*-MAT436.
119. Supplementary Figure 119. ^{1}H NMR of compound **38**.
120. Supplementary Figure 120. ^{13}C NMR of compound **38**.
121. Supplementary Figure 121. DEPT-135 NMR of compound **38**.
122. Supplementary Figure 122. ^{1}H-^{1}H COSY NMR of compound **38**.
123. Supplementary Figure 123. ^{1}H-^{13}C HMBC NMR of compound **38**.
124. Supplementary Figure 124. ^{1}H-^{13}C HSQC NMR of compound **38**.
125. Supplementary Figure 125. ^{1}H NMR of compound **35**.
126. Supplementary Figure 126. ^{13}C NMR of compound **35**.
127. Supplementary Figure 127. DEPT-135 NMR of compound **35**.
128. Supplementary Figure 128. ^{1}H-^{1}H COSY NMR of compound **35**.
129. Supplementary Figure 129. ^{1}H-^{13}C HMBC NMR of compound **35**.
130. Supplementary Figure 130. ^{1}H-^{13}C HSQC NMR of compound **35**.
131. Supplementary Figure 131. ^{1}H NMR of compound **39**.
132. Supplementary Figure 132. ^{13}C NMR of compound **39**.
133. Supplementary Figure 133. DEPT-135 NMR of compound **39**.
134. Supplementary Figure 134. ^{1}H-^{1}H COSY NMR of compound **39**.
135. Supplementary Figure 135. ^{1}H-^{13}C HMBC NMR of compound **39**.
136. Supplementary Figure 136. ^{1}H-^{13}C HSQC NMR of compound **39**.

137. Supplementary Figure 137. ^{1}H NMR of compound **36**.
138. Supplementary Figure 138. ^{13}C NMR of compound **36**.
139. Supplementary Figure 139. DEPT-135 NMR of compound **36**.
140. Supplementary Figure 140. ^{1}H-^{1}H COSY NMR of compound **36**.
141. Supplementary Figure 141. ^{1}H-^{13}C HMBC NMR of compound **36**.
142. Supplementary Figure 142. ^{1}H-^{13}C HSQC NMR of compound **36**.
143. Supplementary Figure 143. ^{1}H NMR of compound **46**.
144. Supplementary Figure 144. ^{13}C NMR of compound **46**.
145. Supplementary Figure 145. ^{1}H NMR of compound **47**.
146. Supplementary Figure 146. ^{13}C NMR of compound **47**.
147. Supplementary Figure 147. ^{1}H NMR of compound **48**.
148. Supplementary Figure 148. ^{13}C NMR of compound **48**.
149. Supplementary Figure 149. ^{1}H NMR of compound **49**.
150. Supplementary Figure 150. ^{13}C NMR of compound **49**.
151. Supplementary Figure 151. ^{1}H NMR of compound **50**.
152. Supplementary Figure 152. ^{13}C NMR of compound **50**.

II. Supplementary Tables

1. Supplementary Table 1. Crystallographic data and refinement statistics

III. Supplementary Methods

1. Synthetic procedures and compound characterization

IV. Supplementary References

Supplementary Figures

a

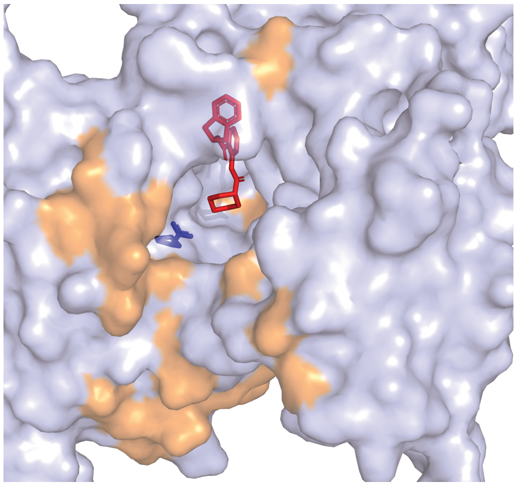

b

```
                180       190       200       210       220       230         240
PfcProRS  EYVQIKEDTTSKARVDKKEDVQEEMAKNEELQNNNNNNKNNSNSNNNNNNNNNHIKDTILK--GKLLSNNEVEDNKSKDS
          : .:.: .  :   :. :  :.   :.... ......::.... :. ...:... ::   .  : : :..  : .  : .
HsProRS   ELLQLKAQYKSLIGVEYKP-VSATGAEDKDKKKKEKENKSEKQ-NKPQKQNDGQRKDPSKNQGGGLSSSGAGEGQGPKKQ
              940       950        960       970        980       990      1000      1010

        250       260       270       280       290       300       310       320
PfcProRS  NILGITSKKIENFSDWYTQVIVKSELIEYYDISGCYILRPAAYYIWECVQAFFNKEIKKLNVENSYFPLFVTKNKLEKEK
          . ::. .:: ::..:::.:::.:::.:::.:::::::::: :: ::: .. ::. :::::.::: :::.::... :::::
HsProRS   TRLGLEAKKEENLADWYSQVITKSEMIEYHDISGCYILRPWAYAIWEAIKDFFDAEIKKLGVENCYFPMFVSQSALEKEK
               1020      1030      1040      1050      1060      1070      1080      1090

        330       340       350       360       370       380       390       400
PfcProRS  NHIEGFSPEVAWVTKYGDSNLPEEIAIRPTSETIMYSVFPKWIRSYRDLPLKLNQWNTVVRWEFKQPTPFIRTREFLWQE
          .:.  :.:::::::. : ..: : ::::::::::.:: .. ::..:.::::.::::: .:::::::.: ::.:::::::::
HsProRS   THVADFAPEVAWVTRSGKTELAEPIAIRPTSETVMYPAYAKWVQSHRDLPIKLNQWCNVVRWEFKHPQPFLRTREFLWQE
               1100      1110      1120      1130      1140      1150      1160      1170

        410       420       430       440       450       460       470       480
PfcProRS  GHTAHKNEEEAVKLVFDILDLYRRWYEEYLAVPIIKGIKSEGEKFGGANFTSTAEAFISENGRAIQAATSHYLGTNFAKM
          ::.:  . :::.. :..::::: . ::: ::.:..:: :.: :::.:...:.: ::::: .:::::..:::.:: ::.::
HsProRS   GHSAFATMEEAAEEVLQILDLYAQVYEELLAIPVVKGRKTEKEKFAGGDYTTTIEAFISASGRAIQGGTSHHLGQNFSKM
               1180      1190      1200      1210      1220      1230      1240      1250

        490         500       510       520       530       540       550           560
PfcProRS  FKIEFEDEN--EVKQYVHQTSWGCTTRSIGIMIMTHGDDKGLVLPPNVSKYKVVIVPIFYKTT----DENAIHSYCKDIE
          :.: ::: .    ::...:.::: :::.::.: :.:::. :::::: :.  .:::.:    ..    :..:. . :.: .
HsProRS   FEIVFEDPKIPGEKQFAYQNSWGLTTRTIGVMTMVHGDNMGLVLPPRVACVQVVIIPCGITNALSEEDKEALIAKCNDYR
               1260      1270      1280      1290      1300      1310      1320      1330

              570       580       590       600       610       620       630       640
PfcProRS  KILKNAQINCVYDDRASYSPGYKFNHWELRGIPIRIEVGPKDLQNNSCVIVRRDNNEKCNVKKESVLLETQQMLVDIHKN
          . : ...:    : : .::::.:::::::.:.:::.::::.:... . : ::::..:: .: .. .  . : .: ::. .
HsProRS   RRLLSVNIRVRADLRDNYSPGWKFNHWELKGVPIRLEVGPRDMKSCQFVAVRRDTGEKLTVAENEAETKLQAILEDIQVT
               1340      1350      1360      1370      1380      1390      1400      1410

              650       660       670       680       690       700       710       720
PfcProRS  LFLKAKKKLDDSIVQVTSFSEVMNALNKKKMVLAPWCEDIATEEEIKKETQRLSLNQTNSETTLSGAMKPLCIPLDQP--
          :: .:.. :   .: .... . .. :.. :.:  :.: .:  :. ::: : :   .:     . : . : ::::. .:
HsProRS   LFTRASEDLKTHMVVANTMEDFQKILDSGKIVQIPFCGEIDCEDWIKKTTAR---DQDLEPGAPSMGAKSLCIPF-KPLC
               1420      1430      1440      1450      1460         1470      1480

                730       740
PfcProRS  PMPPNMKCFWSGKPAKRWCLFGRSY
           . :. ::  . .::: . ::::::
HsProRS   ELQPGAKCVCGKNPAKYYTLFGRSY
         1490      1500      1510
```

Supplementary Figure 1. Comparison of non-conserved ProRS residues adjacent to the active site. a, Comparison of co-crystal structure of T-3767758 (**2**, red) and proline (blue) bound to *Hs*ProRS (PDB: 5VAD) with *Pfc*ProRS (PDB: 6T7K). The surface of *Pfc*ProRS is shown in light blue and non-conserved residues are highlighted in orange. **b**, Sequence alignment between *Hs*ProRS and *Pfc*ProRS. Active site residues are in bold with non-conserved active site or adjacent residues in orange.

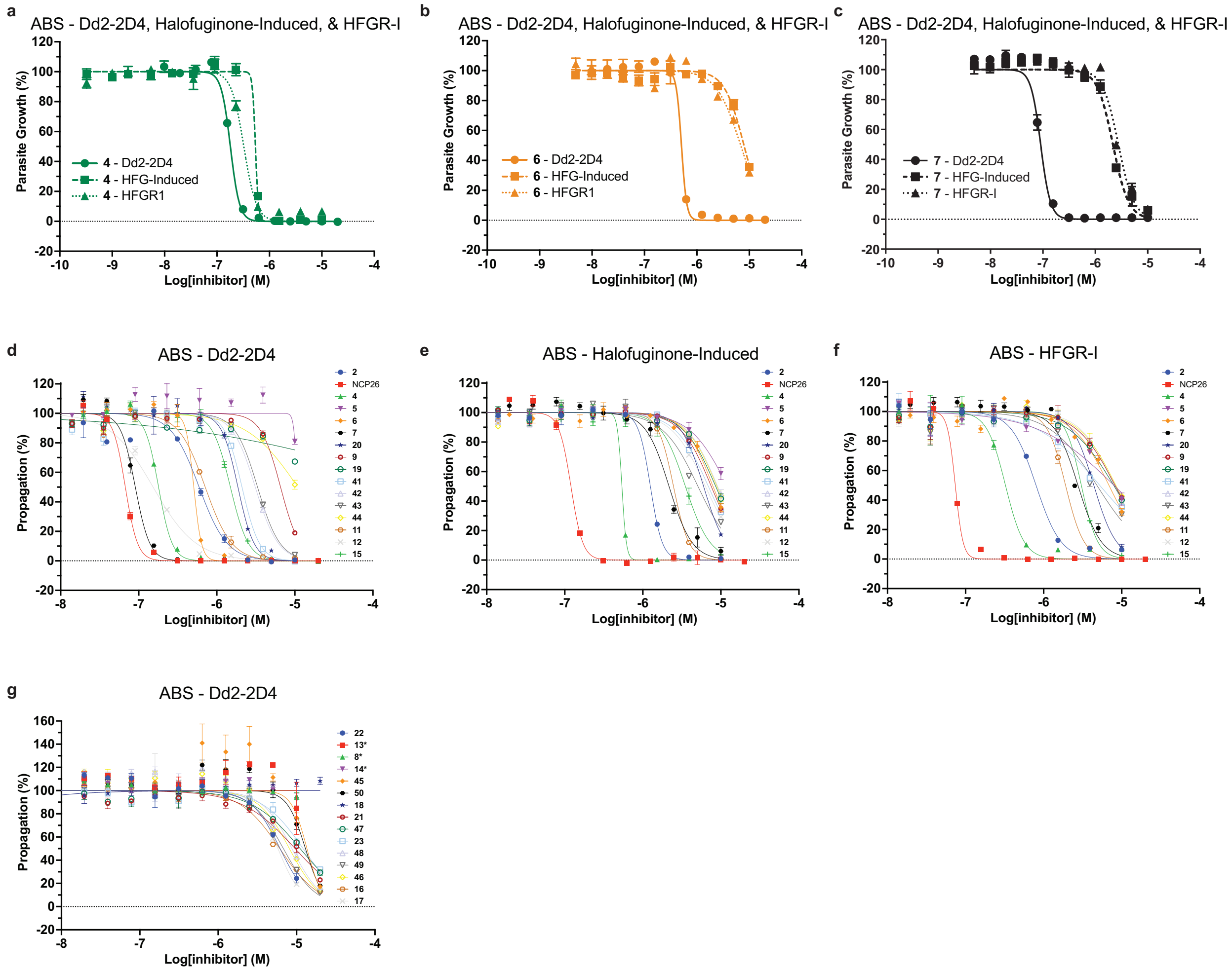

Supplementary Figure 2. Asexual blood stage *P. falciparum* activity of ATP-site targeted pyrazinamide-derived ProRS inhibitors. a-c, *In vitro* characterization of pyrazinamides **4** (**a**), **6** (**b**), and **7** (**c**) in wildtype (Dd2-2D4; circles and solid lines), halofuginone-induced (squares and dashed lines), and HFGR-I (triangles and dotted lines) ABS *P. falciparum* parasites. **d-g**, *In vitro* characterization of ProRS inhibitors in Dd2-2D4 wildtype (**d** and **g**), halofuginone-induced (**e**), and HFGR-I (**f**) ABS *P. falciparum* parasites. Data in panels **d** and **g** was split for visualization purposes only. Data are expressed as mean ± s.d. (*n* = 3 biological replicates) and are representative of at least 3 independent experiments, except for **8**, **13**, and **14** which had one independent experiment each in Dd2-2D4. In panels **a-c**, **4** is shown in green, **6** is shown in orange, and **7** is shown in black. In panels **d-f**, inhibitors are displayed as: **2** (blue, solid circles), NCP26 (red, solid squares), **4** (green, solid upwards triangles), **5** (purple, solid downwards triangles), **6** (orange, solid diamonds), **7** (black, solid hexagons), **20** (blue, stars), **9** (brown, hollow circles), **19** (green, hollow circles), **41** (light blue, hollow squares), **42** (light grey, hollow upwards triangles), **43** (grey, hollow downwards triangles), **44** (yellow, hollow diamonds), **11** (light brown, hollow hexagons), **12** (grey, 'X's), and **15** (green, crosses). In panel **g**, inhibitors are displayed as: **22** (blue, solid circles), **13** (red, solid squares), **8** (green, solid upwards triangles), **14** (purple, solid downwards triangles), **45** (orange, solid diamonds), **50** (black, solid hexagons), **18** (blue, stars), **21** (brown, hollow circles), **47** (green, hollow circles), **23** (light blue, hollow squares), **48** (light grey, hollow upwards triangles), **49** (grey, hollow downwards triangles), **46** (yellow, hollow diamonds), **16** (light brown, hollow hexagons), and **17** (grey, 'X's).

Abbreviations: ABS = asexual blood stage; HFG-induced = halofuginone-induced.

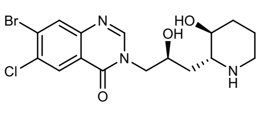

Halofugionol (**26**)

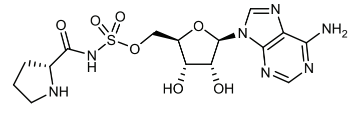

D-ProSA (**27**)

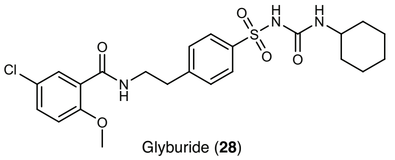

Glyburide (**28**)

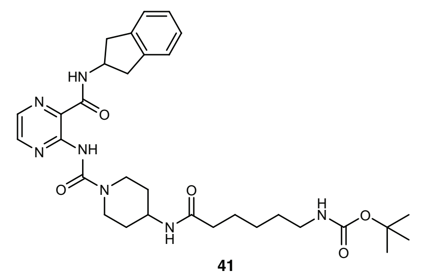

41

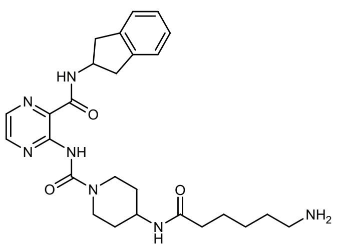

42

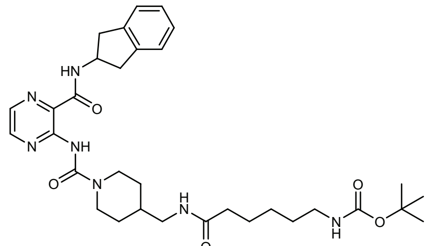

43

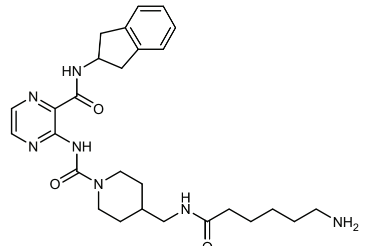

44

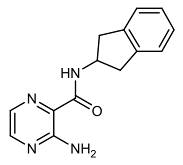

45

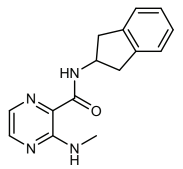

46

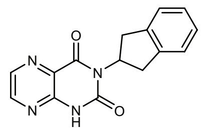

47

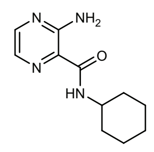

48

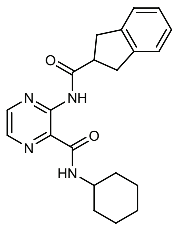

49

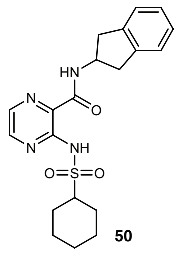

50

Supplementary Figure 3. Additional chemical structures. Structures of additional pyrazinamide inhibitors and of reference compounds halofuginol (**26**, relative stereochemistry), D-ProSA (**27**), and glyburide (**28**).

a b

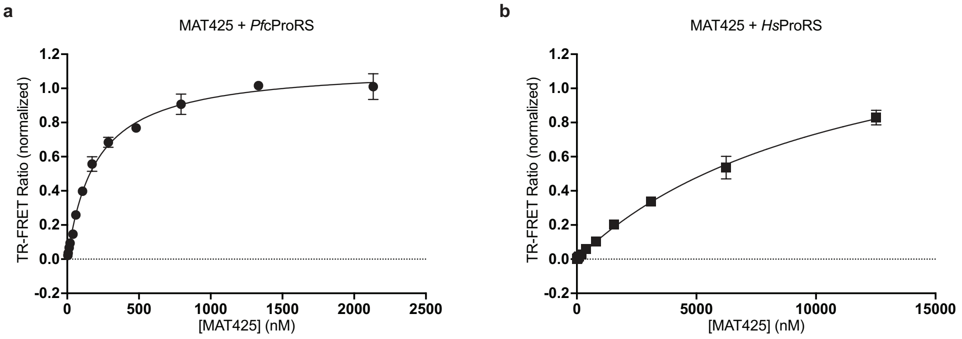

c

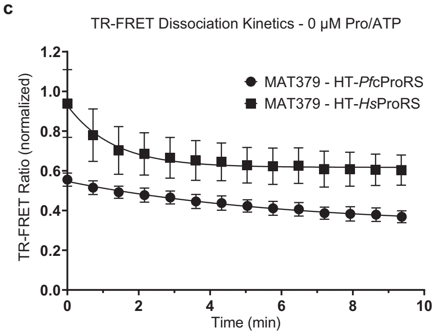

d

	*Pf*cProRS			*Hs* ProRS		
	mean	lower 95% CI limit	upper 95% CI limit	mean	lower 95% CI limit	upper 95% CI limit
k_{off} (min^{-1})	0.159	0.147	0.172	0.527	0.427	0.681
$k_{on,obs}$ ($M^{-1}min^{-1}$)	-	-	-	-	-	-
$k_{on,calc}$ ($M^{-1}min^{-1}$)	1.59E+06	1.47E+06	1.71E+06	3.10E+05	2.40E+05	4.01E+05

Supplementary Figure 4. Additional characterization of TR-FRET tracers MAT379 (24) and MAT425. a-**b**, Saturation binding of fluorescent tracer MAT425 to (**a**) CoraFluor-1-labeled HT-*Pf*cProRS (1 nM) or (**b**) CoraFluor-1-labeled HT-*Hs*ProRS (1.5 nM). TR-FRET ratios were normalized relative to 10 µM ProSA (~20,000x K_D). Data in **a** and **b** are shown as mean ± s.d. (*n* = 3 independent replicate wells) and are representative of at least 2 independent experiments. **c**, Determination of dissociation kinetics for tracer MAT379. An equilibrated solution of 100 nM CoraFluor-1-labeled HT-*Pf*cProRS (circles) or HT-*Hs*ProRS (squares) and ~EC_{80} MAT379 (560 nM for HT-*Pf*cProRS and 7 µM for HT-*Hs*ProRS) was diluted 10-fold into assay buffer containing no ProRS or MAT379 and the TR-FRET ratio (520/490 nm) was measured in ~45 s intervals over the course of 10 min. TR-FRET ratios were normalized relative to both the initial time point and 10 µM ProSA (~20,000x K_D) prior to dilution. Kinetics data in **c** are expressed as mean ± s.d. (*n* = 23 independent replicate wells) and are representative of ≥ 2 independent experiments. **d**, Summary of binding kinetics data for MAT379 determined using the TR-FRET-based ligand displacement assay. The dissociation rates (k_{off}) were experimentally determined, but the association rates (k_{on}) were too fast to measure ($k_{on,obs}$) so they were instead calculated ($k_{on,calc}$) using the corresponding equilibrium dissociation constant (K_D) and k_{off} value (see Supplementary Methods).

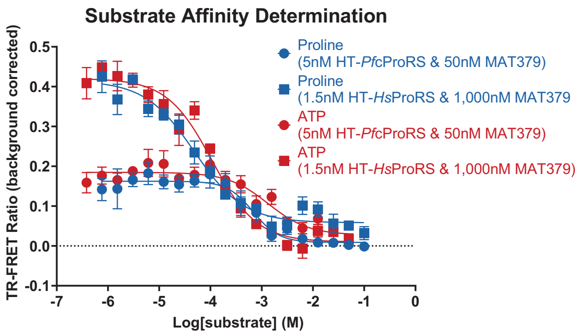

Supplementary Figure 5. ProRS Substrate Affinity Determination. Dose-response titration of proline (blue) or ATP (red) using CoraFluor-1-labeled HT-*Pfc*ProRS (5 nM, circles) or CoraFluor-1-labeled HT-*Hs*ProRS (1.5 nM, squares) and tracer MAT379 (50 nM for HT-*Pfc*ProRS and 1,000 nM for HT-*Hs*ProRS). TR-FRET ratios were background-corrected relative to 10 µM ProSA (~20,000x K_D). Data are expressed as mean ± s.d. (n = 4 independent replicate wells) and are representative of ≥ 2 independent experiments.

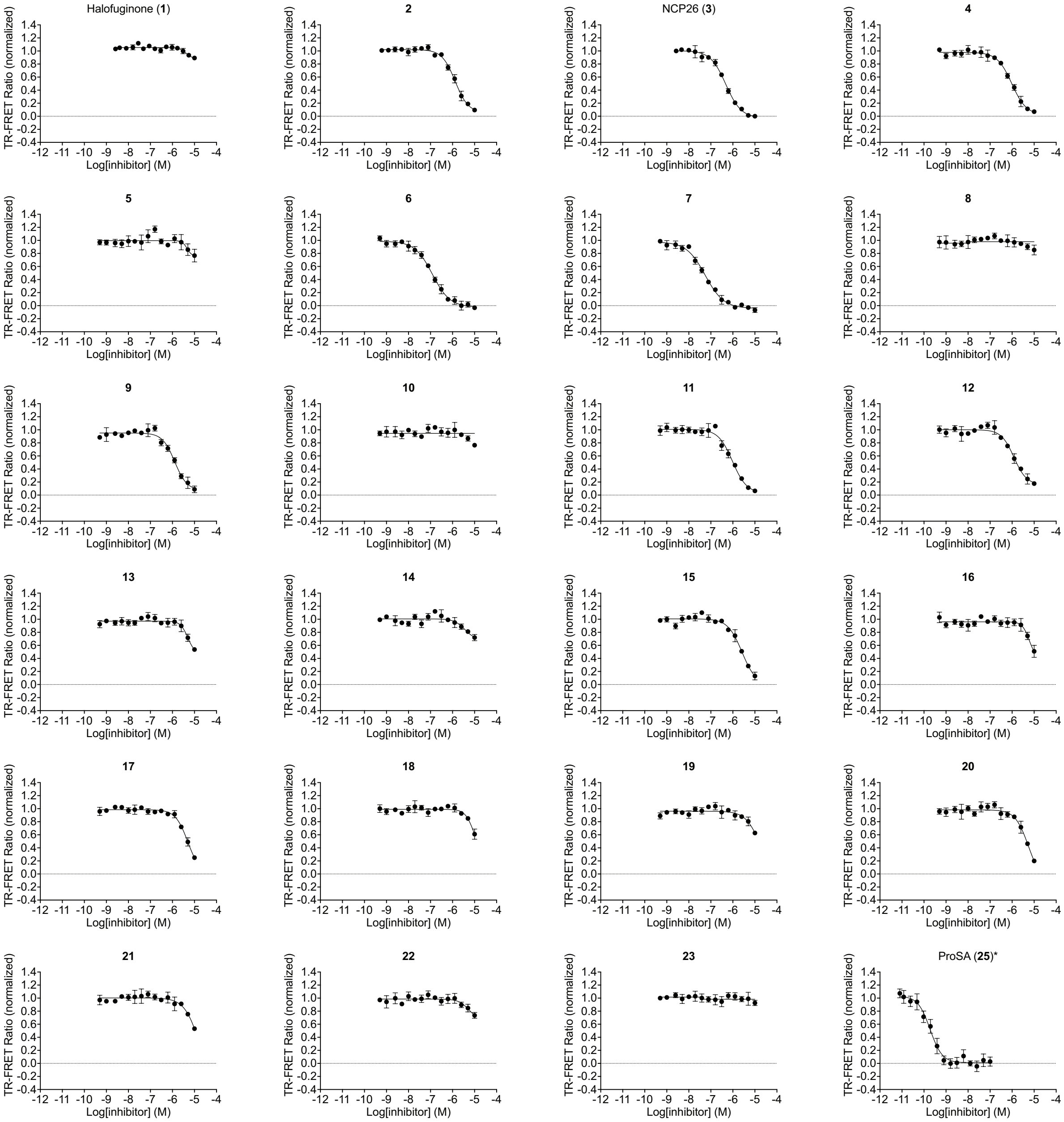

Supplementary Figure 6. TR-FRET-based assay data for *Pf*cProRS in the absence of substrates – Set 1. Dose-response titration of ProRS inhibitors in the absence of substrates using CoraFluor-1-labeled HT-*Pf*cProRS (0.020-1 nM) and MAT379 as tracer at 250 nM (2.5x K_D). Compounds marked with * were supplemented with 1 nM CoraFluor-1-labeled anti-His6 antibody. Data are expressed as mean ± s.d. (*n* = 3 independent replicate wells except for NCP26 where *n* = 2 and ProSA where *n* = 6) and are representative of ≥ 2 independent experiments.

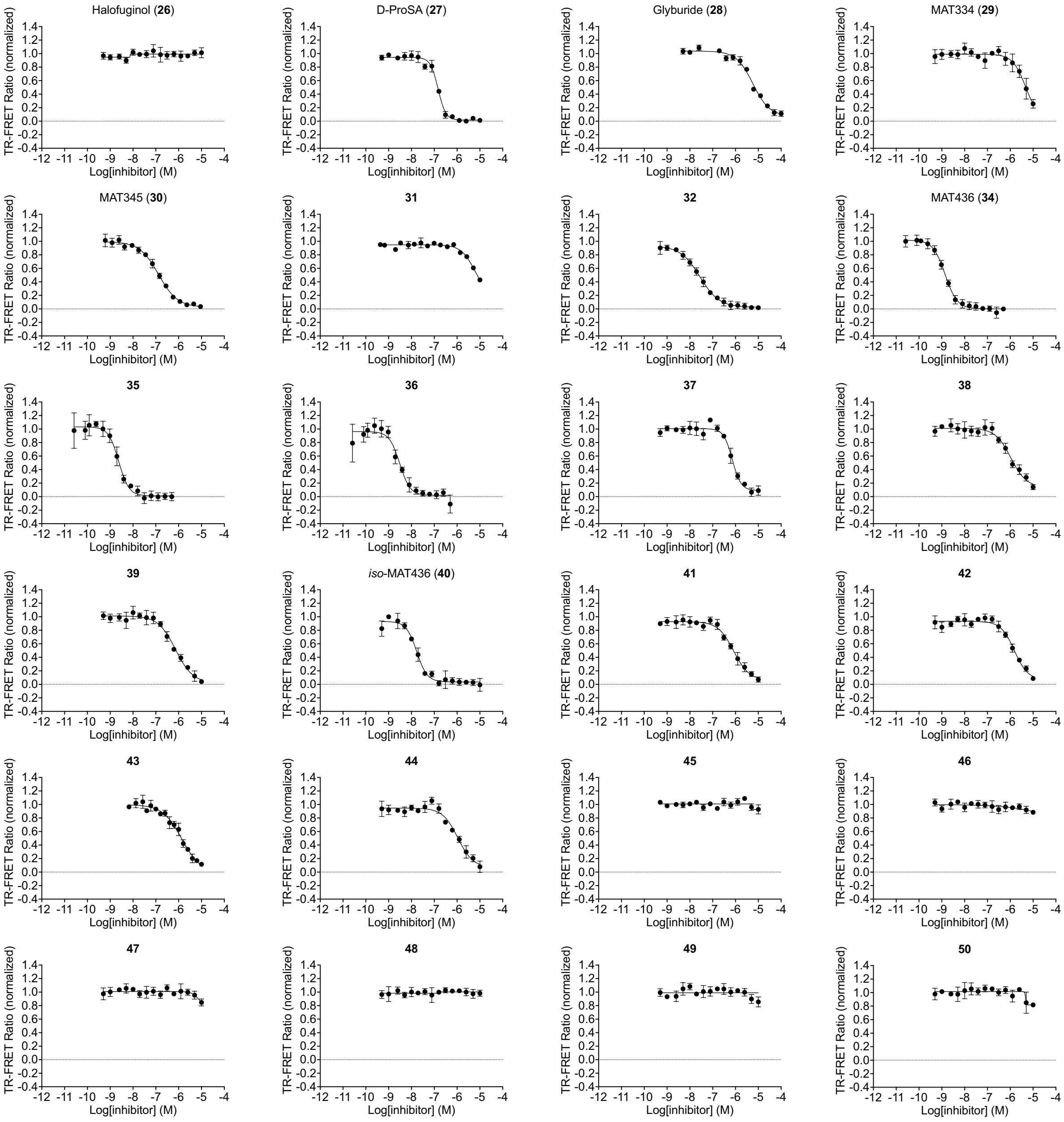

Supplementary Figure 7. TR-FRET-based assay data for *Pf*cProRS in the absence of substrates – Set 2. Dose-response titration of ProRS inhibitors in the absence of substrates using CoraFluor-1-labeled HT-*Pf*cProRS (0.020-1 nM) and MAT379 as tracer at 250 nM (2.5x K_D). Compounds marked with ** were titrating ProRS under these conditions. Data are expressed as mean ± s.d. (n = 3 independent replicate wells except for **31** where n = 2 and for MAT436, **35**, and **36** where n = 6) and are representative of ≥ 2 independent experiments.

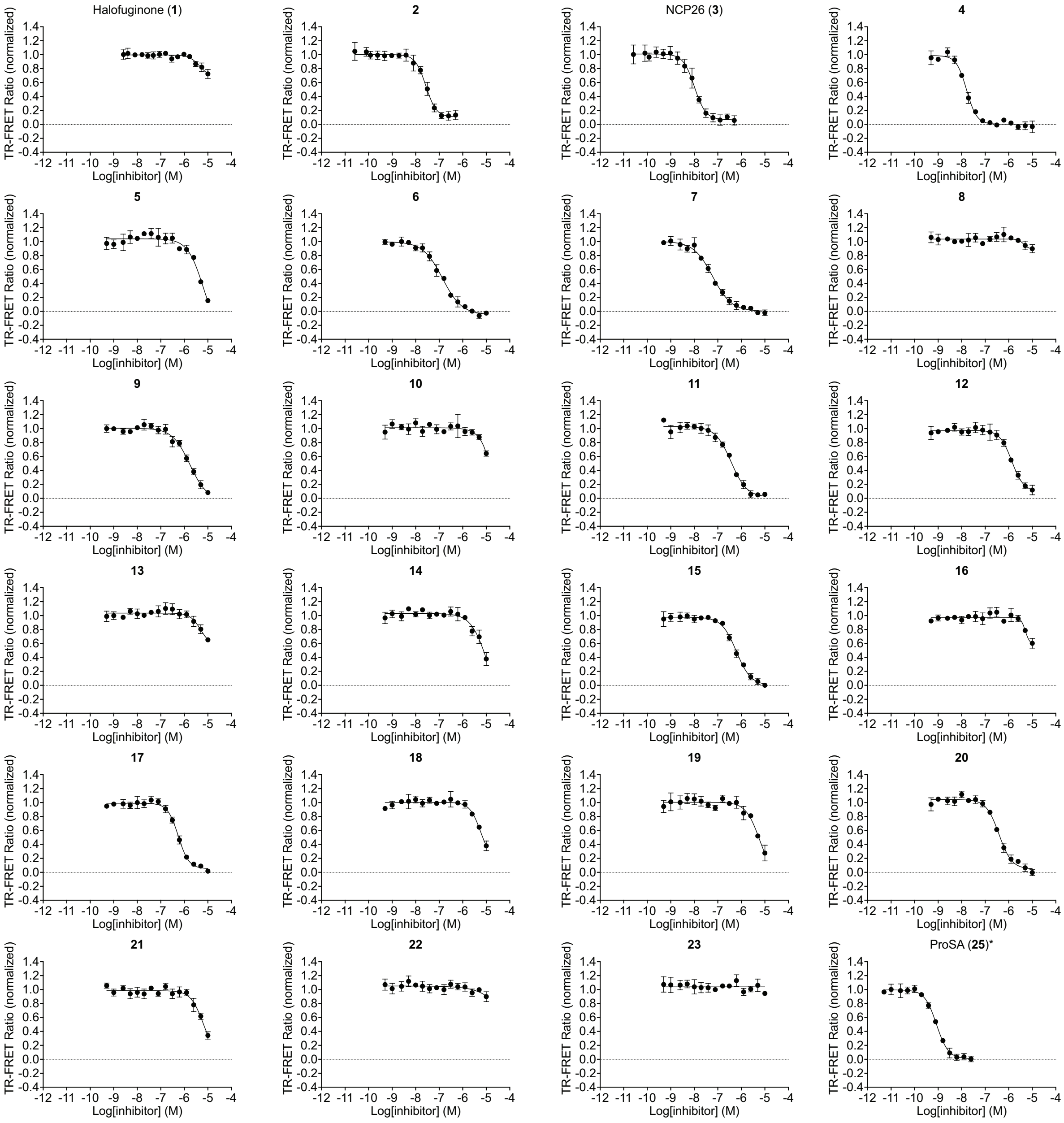

Supplementary Figure 8. TR-FRET-based assay data for *Pf*cProRS in the presence of proline – Set 1. Dose-response titration of ProRS inhibitors in the presence of 100 µM Pro using CoraFluor-1-labeled HT-*Pf*cProRS (0.020-1 nM) and MAT379 as tracer at 250 nM (2.5x K_D). Compounds marked with ** were titrating ProRS under these conditions. Data are expressed as mean ± s.d. (n = 3 independent replicate wells except for **2** and NCP26 where n = 6) and are representative of ≥ 2 independent experiments.

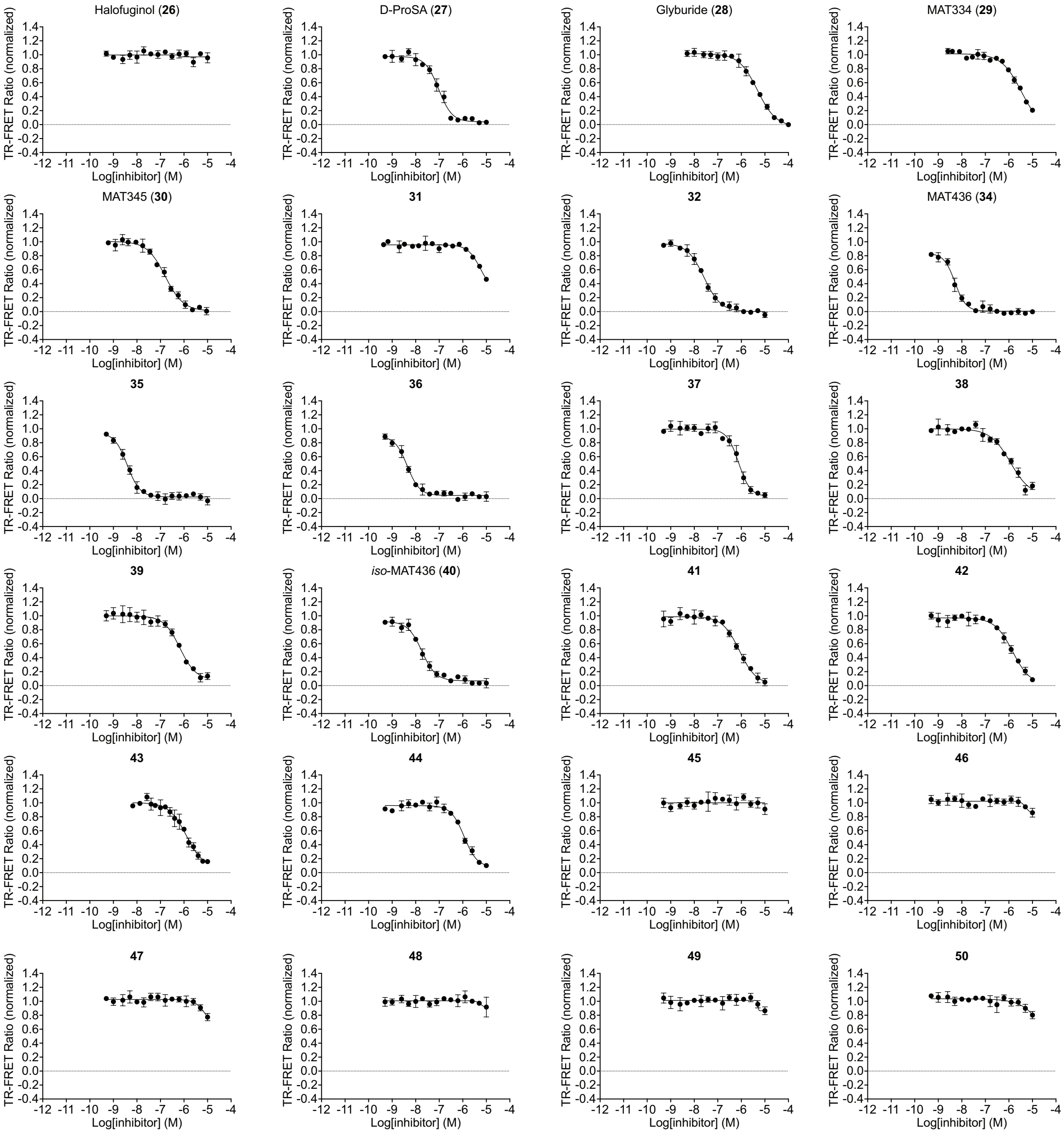

Supplementary Figure 9. TR-FRET-based assay data for *Pf*cProRS in the presence of proline – Set 2. Dose-response titration of ProRS inhibitors in the presence of 100 µM Pro using CoraFluor-1-labeled HT-*Pf*cProRS (0.020-1 nM) and MAT379 as tracer at 250 nM (2.5x K_D). Compounds marked with * were supplemented with 1 nM CoraFluor-1-labeled anti-His6 antibody. Compounds marked with ** were titrating ProRS under these conditions. Data are expressed as mean ± s.d. (n = 3 independent replicate wells except for **31** where n = 2) and are representative of ≥ 2 independent experiments.

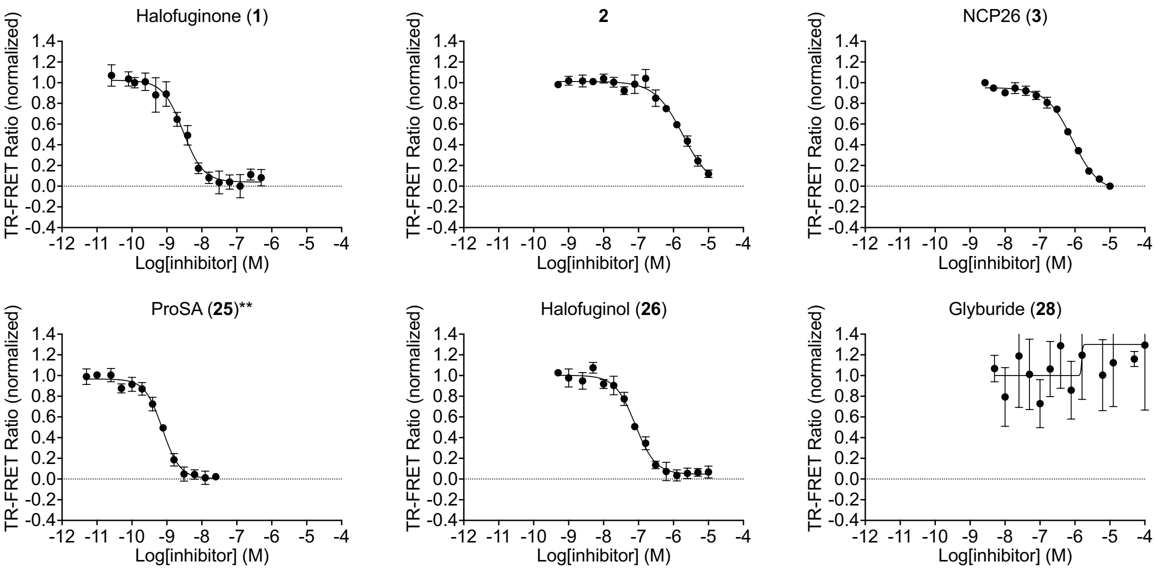

Supplementary Figure 10. TR-FRET-based assay data for *Pf*cProRS in the presence of ATP. Dose-response titration of ProRS inhibitors in the presence of 500 µM ATP using CoraFluor-1-labeled HT-*Pf*cProRS (0.020-1 nM) and MAT379 as tracer at 250 nM (2.5x K_D). Compounds marked with ** were titrating ProRS under these conditions. Data are expressed as mean ± s.d. (n = 3 independent replicate wells except for halofuginone where n = 6 and for NCP26 where n = 2) and are representative of ≥ 2 independent experiments.

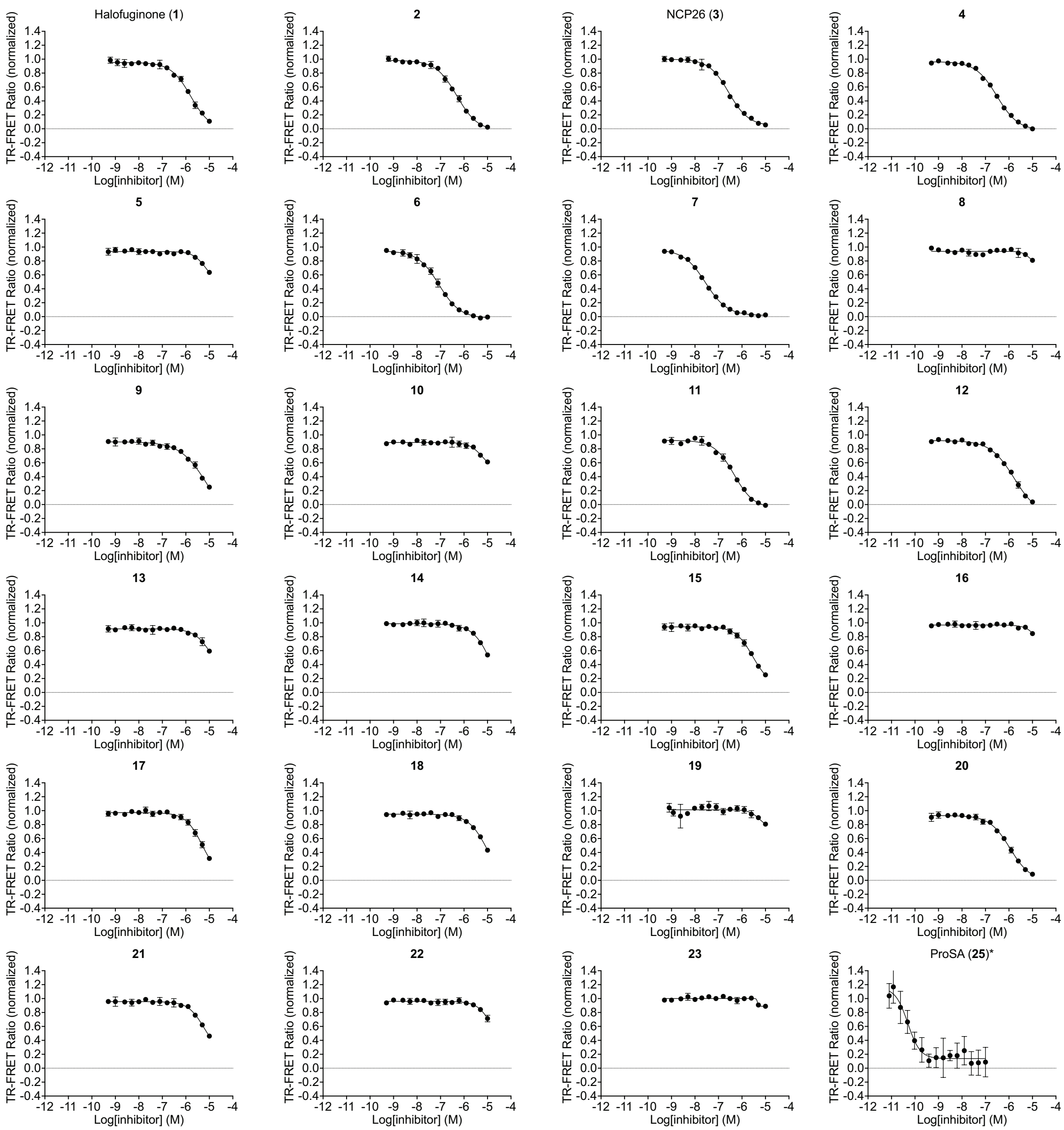

Supplementary Figure 11. TR-FRET-based assay data for *Hs*ProRS in the absence of substrates – Set 1. Dose-response titration of ProRS inhibitors in the absence of substrates using CoraFluor-1-labeled HT-*Hs*ProRS (0.050-1.5 nM) and MAT379 as tracer at 250 nM (0.15x K_D for HT-*Hs*ProRS). Compounds marked with * were supplemented with 1 nM CoraFluor-1-labeled anti-His6 antibody. Data are expressed as mean ± s.d. (n = 3 independent replicate wells except for ProSA where n = 6) and are representative of ≥ 2 independent experiments.

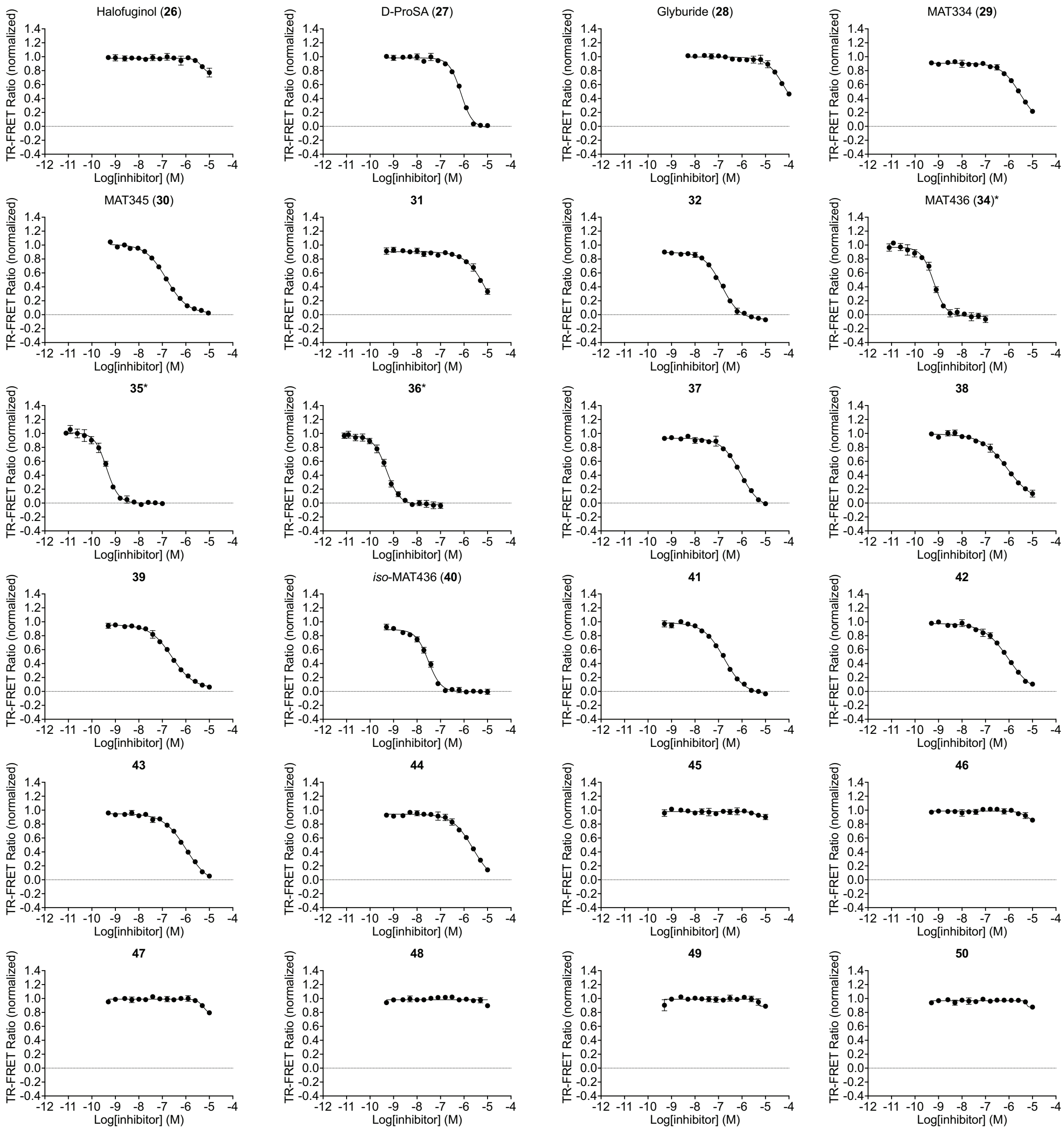

Supplementary Figure 12. TR-FRET-based assay data for *Hs*ProRS in the absence of substrates – Set 2. Dose-response titration of ProRS inhibitors in the absence of substrates using CoraFluor-1-labeled HT-*Hs*ProRS (0.050-1.5 nM) and MAT379 as tracer at 250 nM (0.15x K_D for HT-*Hs*ProRS). Compounds marked with * were supplemented with 1 nM CoraFluor-1-labeled anti-His6 antibody. Data are expressed as mean ± s.d. (n = 3 independent replicate wells except for MAT436, **35**, and **36** where n = 6) and are representative of ≥ 2 independent experiments.

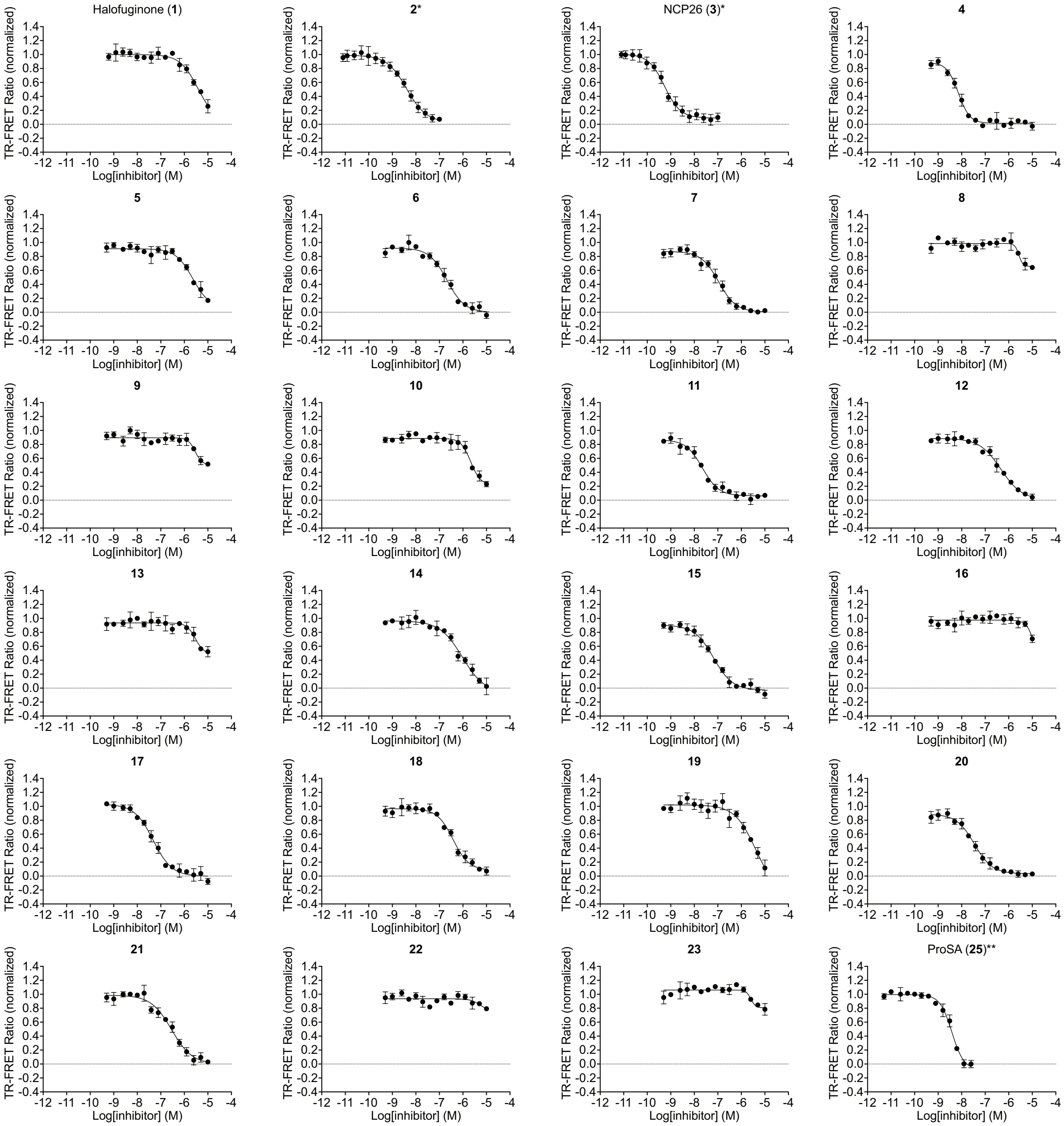

Supplementary Figure 13. TR-FRET-based assay data for *Hs*ProRS in the presence of proline – Set 1. Dose-response titration of ProRS inhibitors in the presence of 100 µM Pro using CoraFluor-1-labeled HT-*Hs*ProRS (0.050-1.5 nM) and MAT379 as tracer at 250 nM (0.15x K_D for HT-*Hs*ProRS). Compounds marked with * were supplemented with 1 nM CoraFluor-1-labeled anti-His6 antibody. Compounds marked with ** were titrating ProRS under these conditions. Data are expressed as mean ± s.d. (*n* = 3 independent replicate wells except for **2** and NCP26 where *n* = 6) and are representative of ≥ 2 independent experiments.

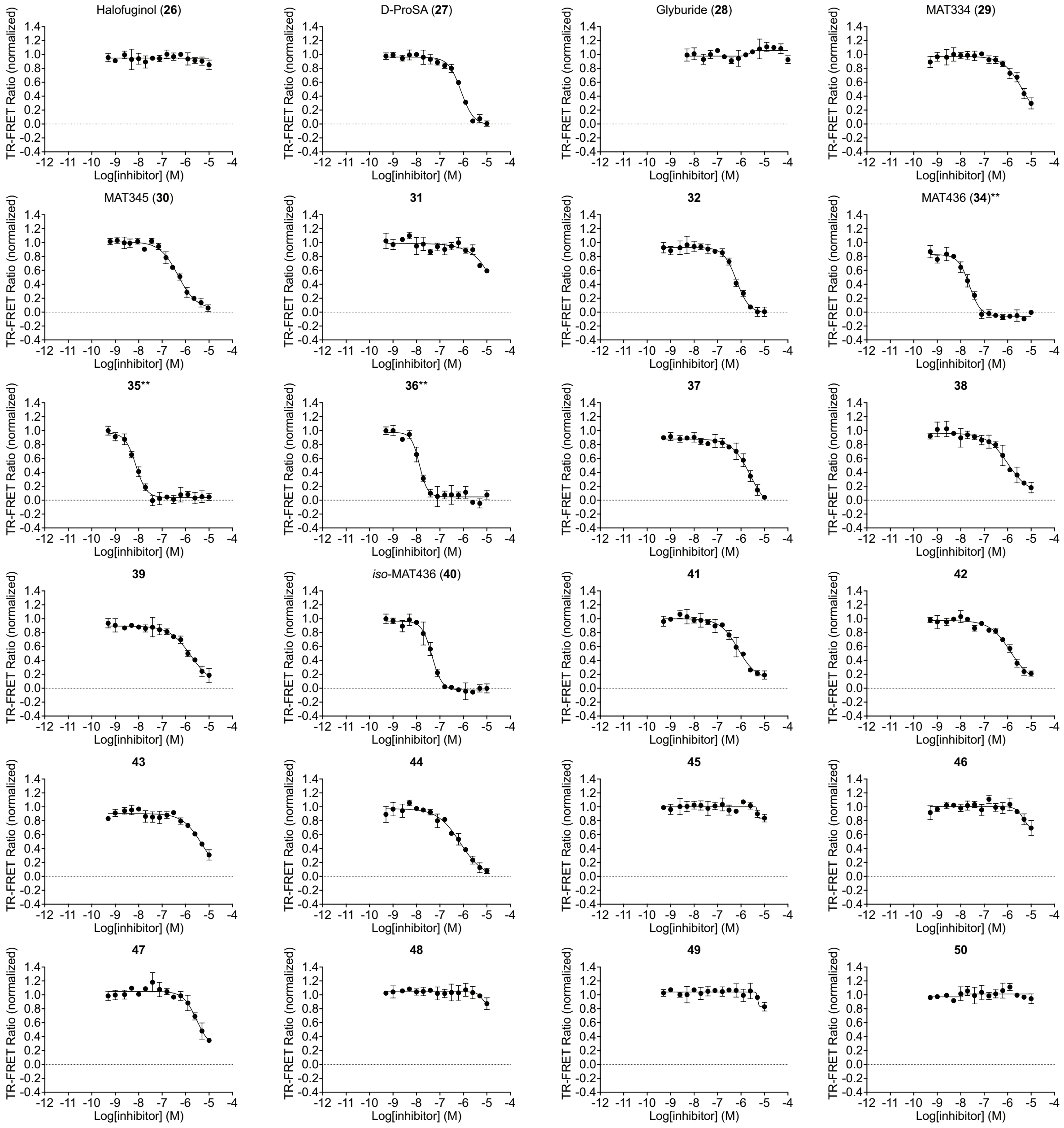

Supplementary Figure 14. TR-FRET-based assay data for *Hs*ProRS in the presence of proline – Set 2. Dose-response titration of ProRS inhibitors in the presence of 100 µM Pro using CoraFluor-1-labeled HT-*Hs*ProRS (0.050-1.5 nM) and MAT379 as tracer at 250 nM (0.15x K_D for HT-*Hs*ProRS). Compounds marked with ** were titrating ProRS under these conditions. Data are expressed as mean ± s.d. (*n* = 3 independent replicate wells) and are representative of ≥ 2 independent experiments.

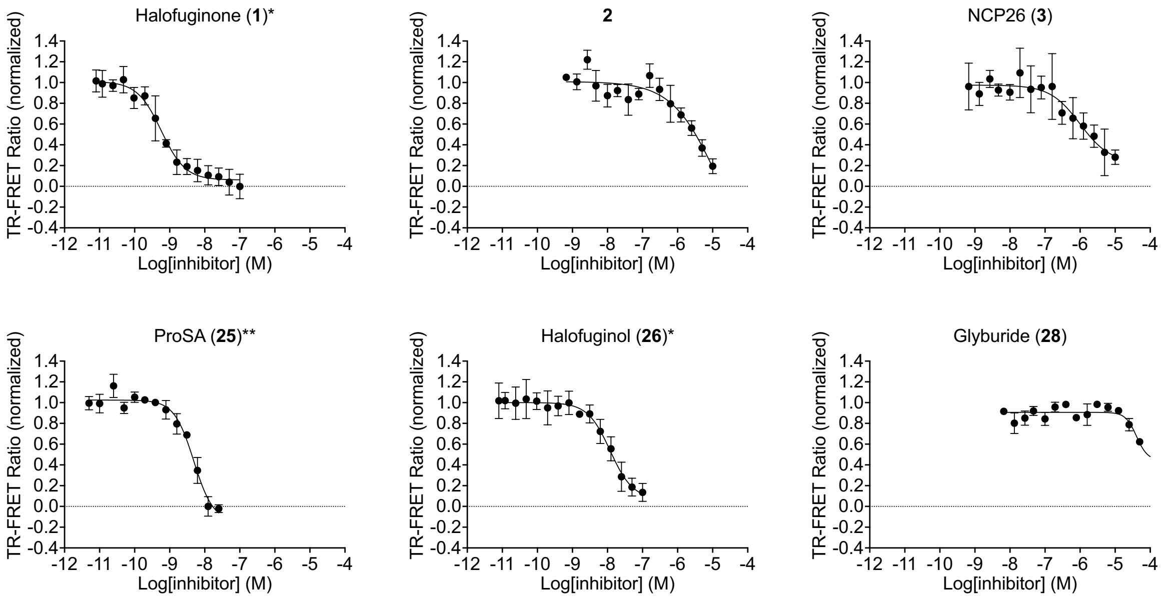

Supplementary Figure 15. TR-FRET-based assay data for *Hs*ProRS in the presence of ATP. Dose-response titration of ProRS inhibitors in the presence of 500 µM ATP using CoraFluor-1-labeled HT-*Hs*ProRS (0.050-1.5 nM) and MAT379 as tracer at 250 nM (0.15x K_D). Compounds marked with * were supplemented with 1 nM CoraFluor-1-labeled anti-His6 antibody. Compounds marked with ** were titrating ProRS under these conditions. Data are expressed as mean ± s.d. (n = 2 independent replicate wells except for halofuginone and halofuginol where n = 6) and are representative of ≥ 2 independent experiments.

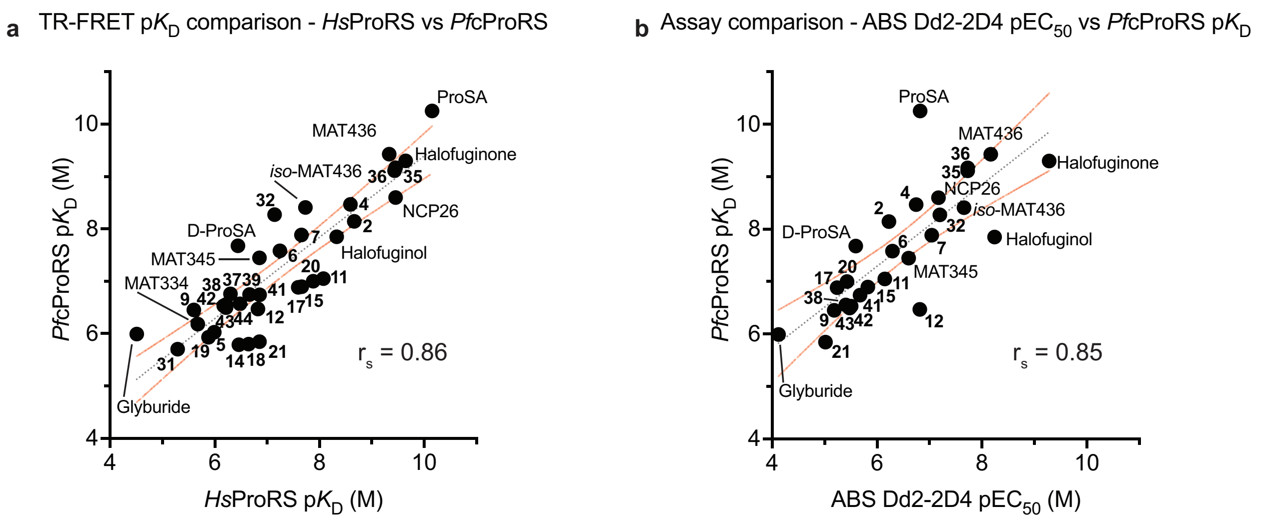

Supplementary Fig. 16. Correlation between TR-FRET pK_D values and *P. falciparum* asexual blood stage growth assay pEC$_{50}$ values. a, Comparison of TR-FRET pK_D values for HT-*Hs*ProRS (x-axis) vs HT-*Pf*cProRS (y-axis). **b**, Comparison of asexual blood stage (ABS) *P. falciparum* Dd2-2D4 pEC$_{50}$ (x-axis) vs HT-*Pf*cProRS TR-FRET pK_D value (y-axis). Data are expressed as the respective mean values and are representative of ≥ 2 independent experiments. TR-FRET pK_D values shown are from the highest affinity conditions (i.e. data from absence of substrates for ATP- and proline-competitive inhibitors, 100 µM Pro for proline-uncompetitive inhibitors, and 500 µM ATP for ATP-uncompetitive inhibitors). Spearman's rank correlation coefficients (r_s) for both plots are calculated using only pyrazinamide compounds (i.e. excluding ProSA, D-ProSA, halofuginone, halofuginol, and glyburide).

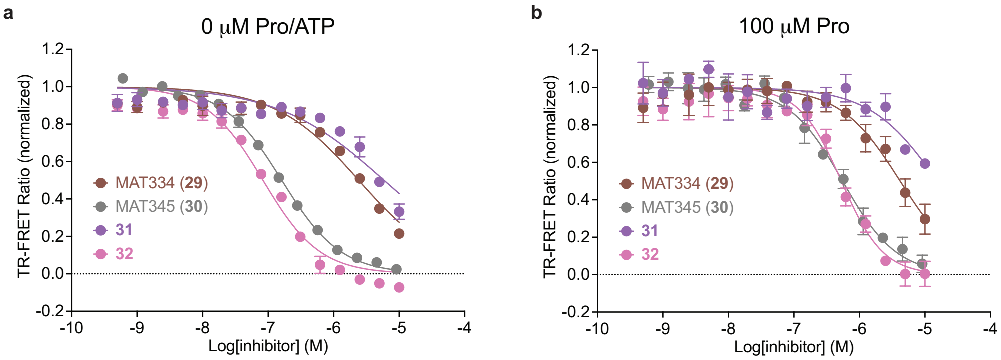

Supplementary Figure 17. Characterization of dual-site ligands binding *Hs*ProRS in the absence or presence of proline. Dose-response titration of pyrazinamide-proline hybrids in the (**a**) absence or (**b**) presence of 100 µM Pro using CoraFluor-1-labeled HT-*Hs*ProRS (1.5 nM) and MAT379 as tracer at 0.15x K_D (250 nM). Data are expressed as mean ± s.d. (n = 3 independent replicate wells) and are representative of ≥ 2 independent experiments. Inhibitors are color coded: MAT334 (brown), MAT345 (grey), **31** (purple), and **32** (pink).

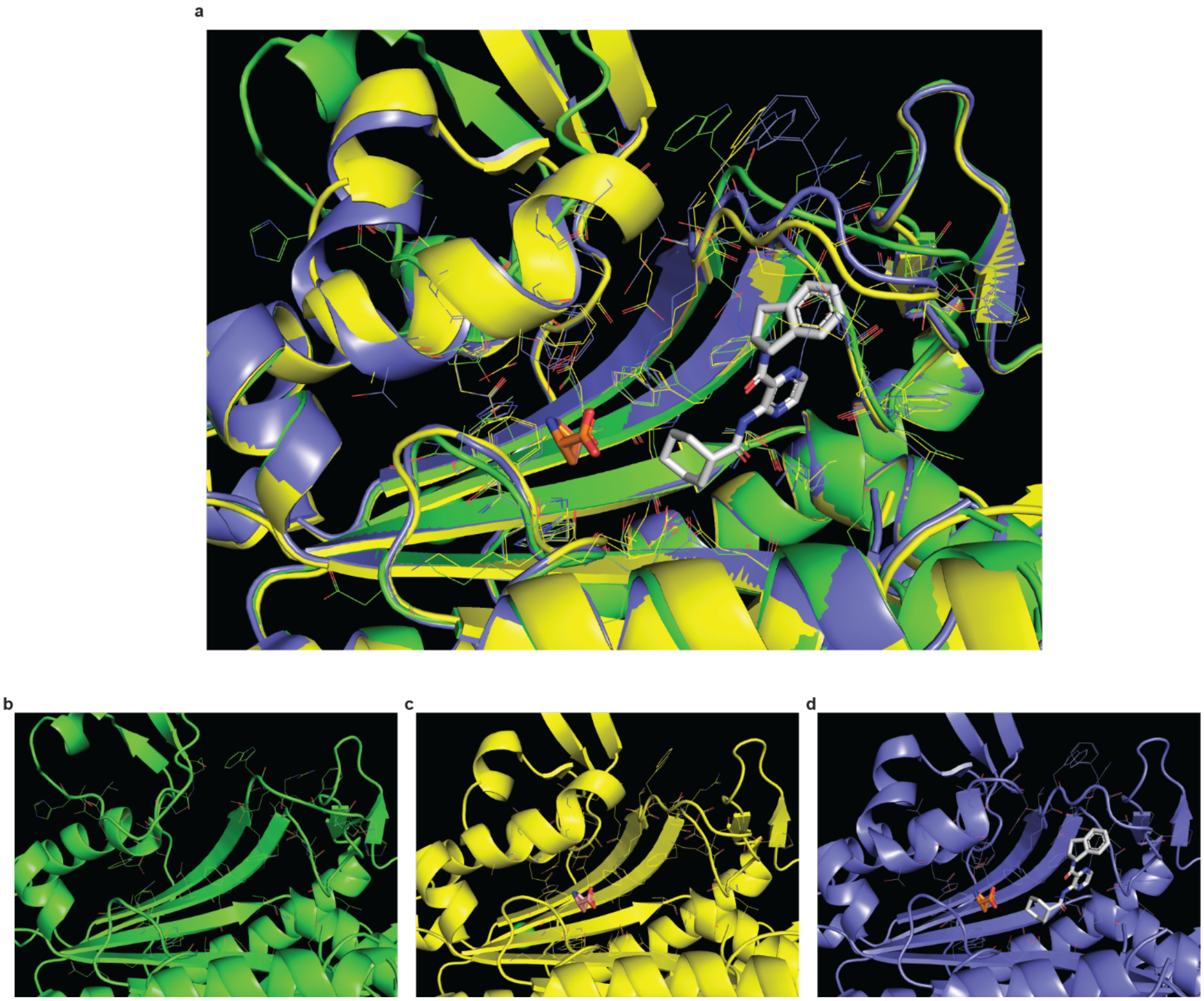

Supplementary Figure 18. Structural comparison of free and ligand-bound *Hs*ProRS. a, Overlay of *Hs*ProRS crystal structures in the apo state (green, PDB: 4K86), bound to proline alone (yellow and pink, respectively; PDB: 7OSY), and bound to both proline and **2** (blue, orange, and white, respectively; PDB: 5VAD) reveals significant allosteric structural changes upon proline binding, including the ATP-binding pocket and the active site entry. Selected residues in and adjacent to the active site are shown as lines. **b**, Apo crystal structure of *Hs*ProRS (PDB: 4K86). **c**, Co-crystal structure of *Hs*ProRS (yellow) bound to proline (pink, PDB: 7OSY). **d**, Co-crystal structure of *Hs*ProRS (blue) bound to proline (orange) and **2** (white, PDB: 5VAD).

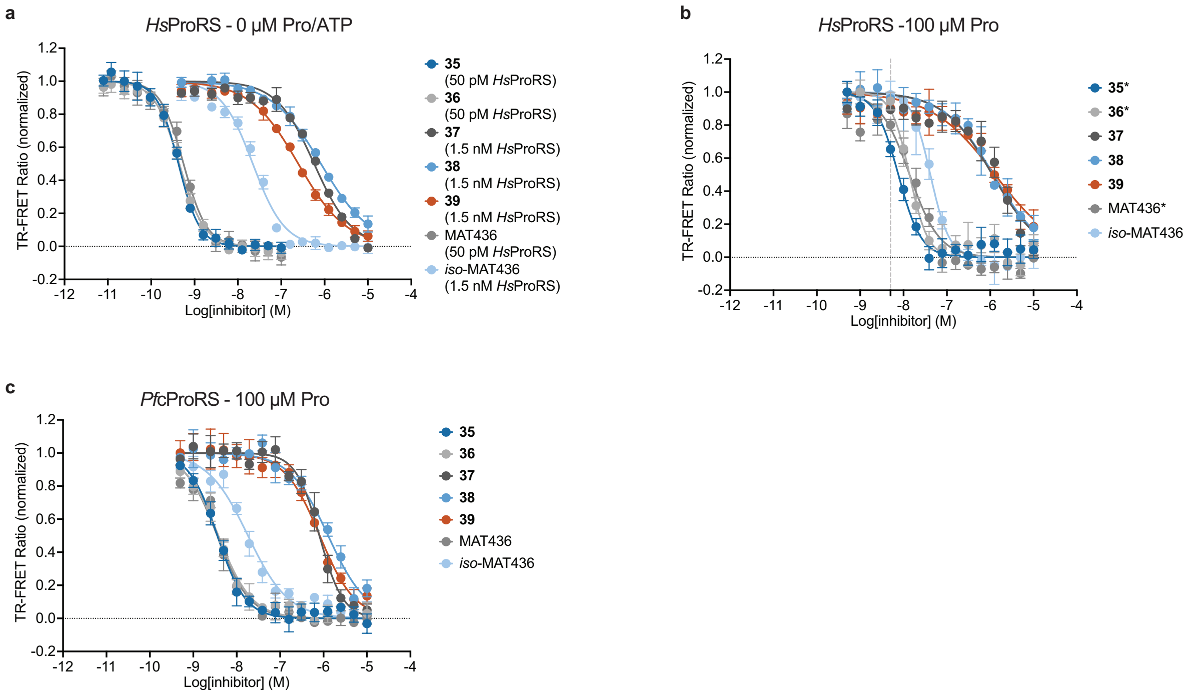

Supplementary Figure 19. Additional characterization of triple-site ligands binding ProRS. a-b, Dose-response titration of pyrazinamide-halofuginone hybrids in the absence (**a**) or presence (**b**) of 100 µM Pro using indicated concentration of CoraFluor-1-labeled HT-*Hs*ProRS and MAT379 as tracer at 0.15x K_D (250 nM). Samples with 50 pM HT-*Hs*ProRS were supplemented with 1 nM CoraFluor-1-labeled anti-His6 antibody. Compounds marked with * are titrating *Hs*ProRS under these conditions. **c**, Dose-response titration of pyrazinamide-halofuginone hybrids in the presence of 100 µM Pro using CoraFluor-1-labeled HT-*Pfc*ProRS (0.5 nM) and MAT379 as tracer at 2.5x K_D (250 nM). TR-FRET data in **a** and **b** are expressed as mean ± s.d. (n = 3 independent replicate wells for samples with 1.5 nM HT-*Hs*ProRS and n = 6 independent replicate wells for samples with 50 pM HT-*Hs*ProRS and 1 nM antibody) and are representative of ≥ 2 independent experiments. TR-FRET data in **c** are expressed as mean ± s.d. (n = 3 independent replicate wells) and are representative of ≥ 2 independent experiments. Inhibitors are color coded: **35** (dark blue), **36** (light grey), **37** (dark grey), **38** (blue), **39** (red), MAT436 (grey), and *iso*-MAT436 (light blue).

Supplementary Fig. 20. ^{1}H NMR of compound **45**.
d_6DMSO

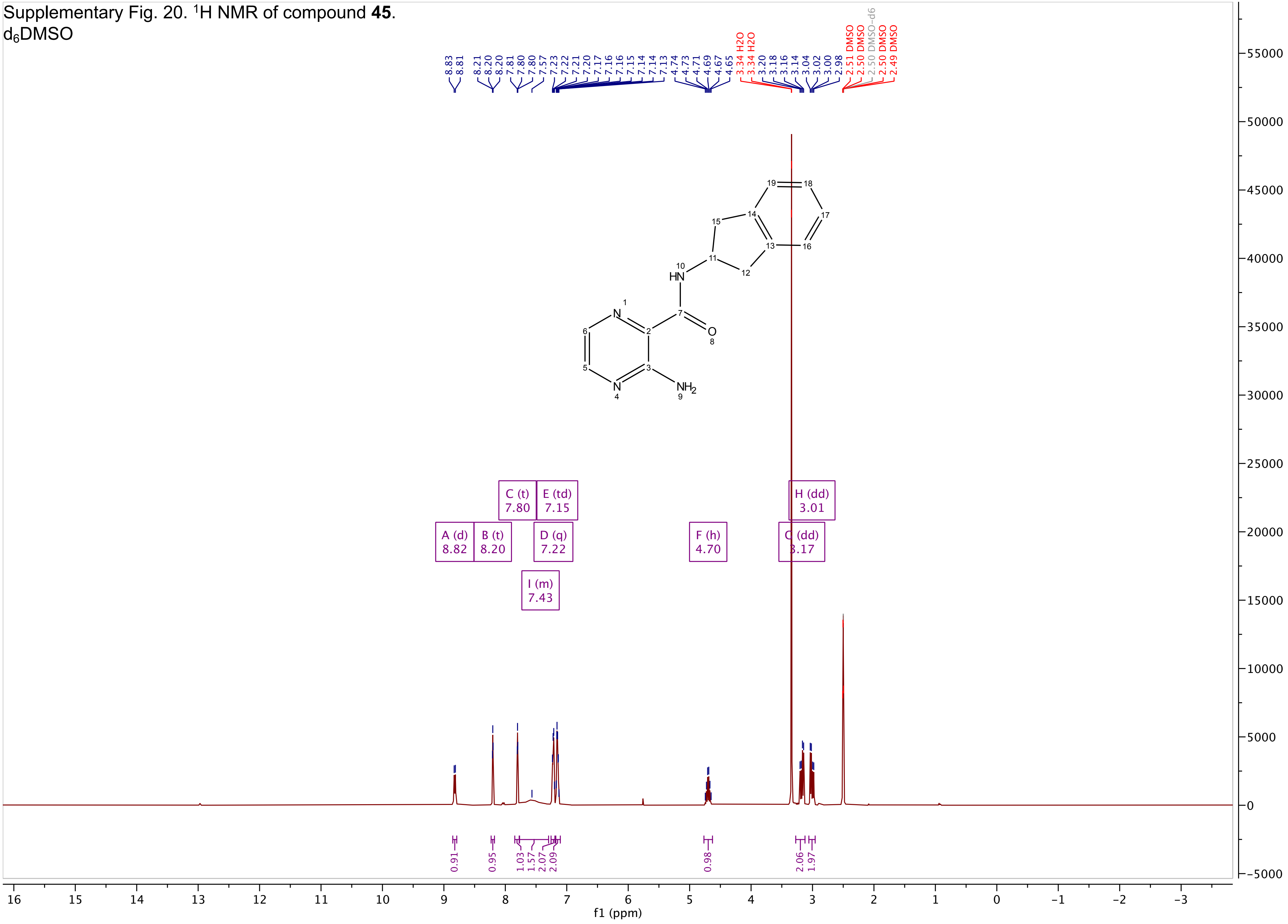

Supplementary Fig. 21. ^{13}C NMR of compound **45**.
d_6DMSO

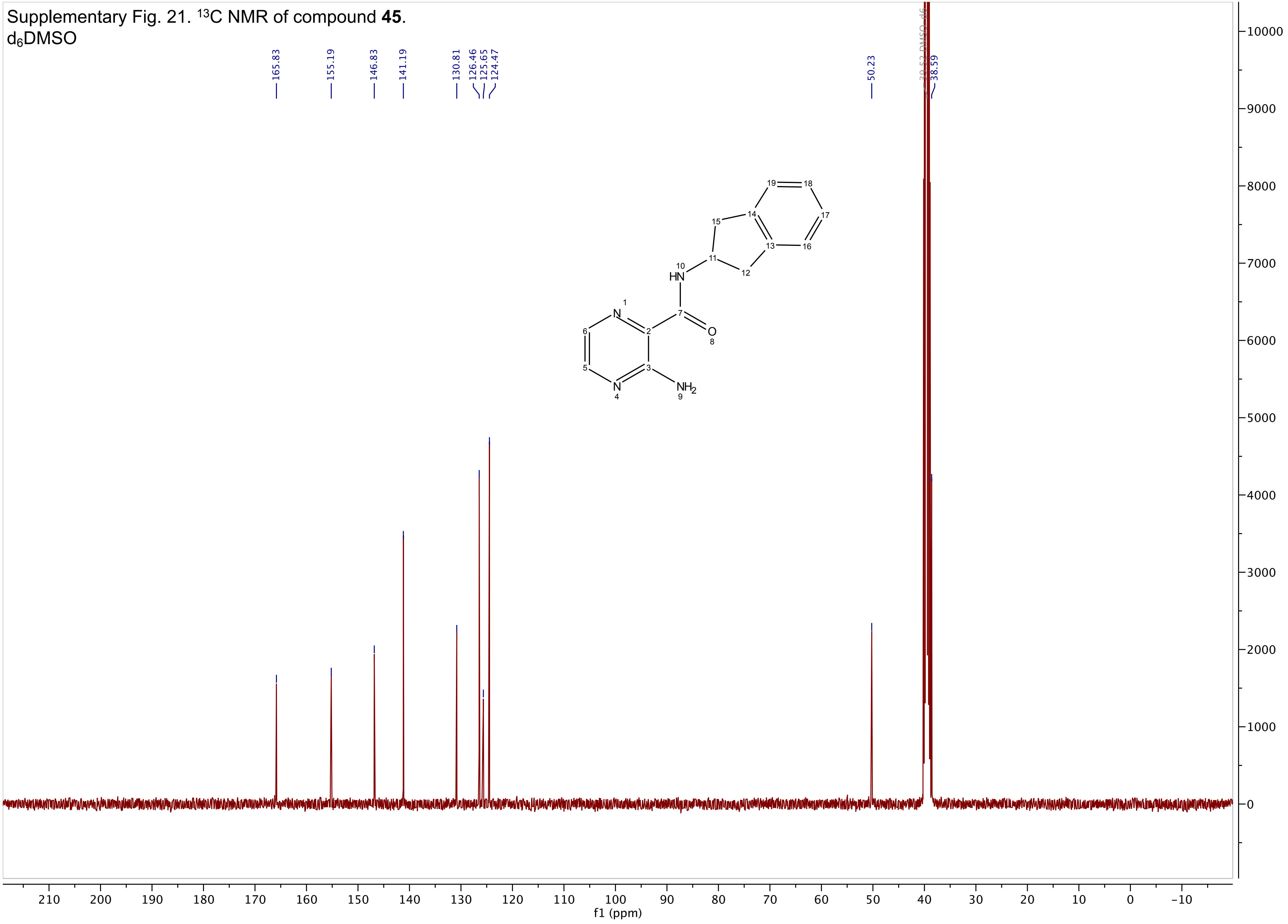

Supplementary Fig. 22. ^{1}H NMR of compound **2**.
$CDCl_3$

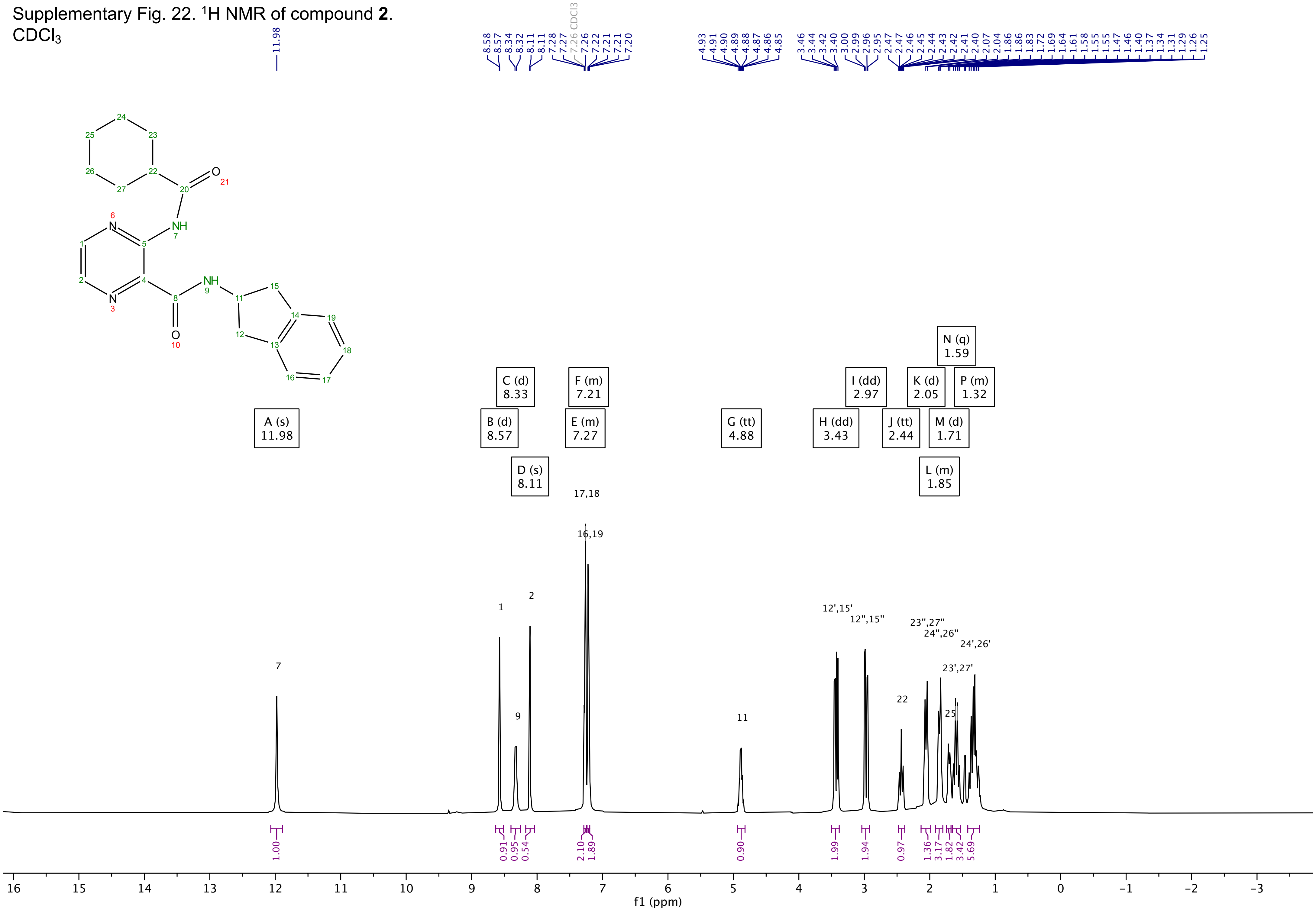

Supplementary Fig. 23. ^{13}C NMR of compound **2**.
$CDCl_3$

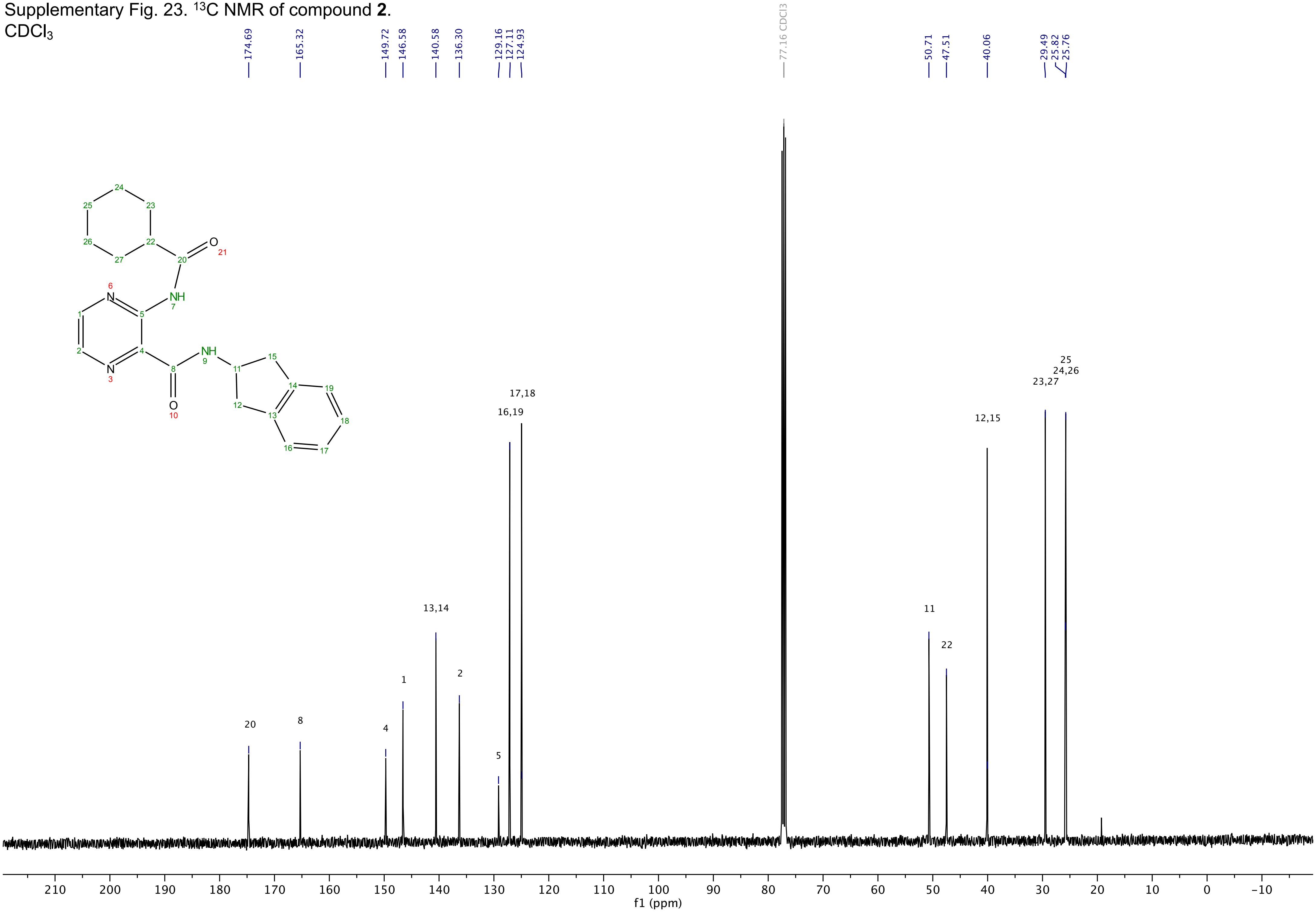

Supplementary Fig. 24. ^{1}H NMR of compound NCP26.
$CDCl_3$

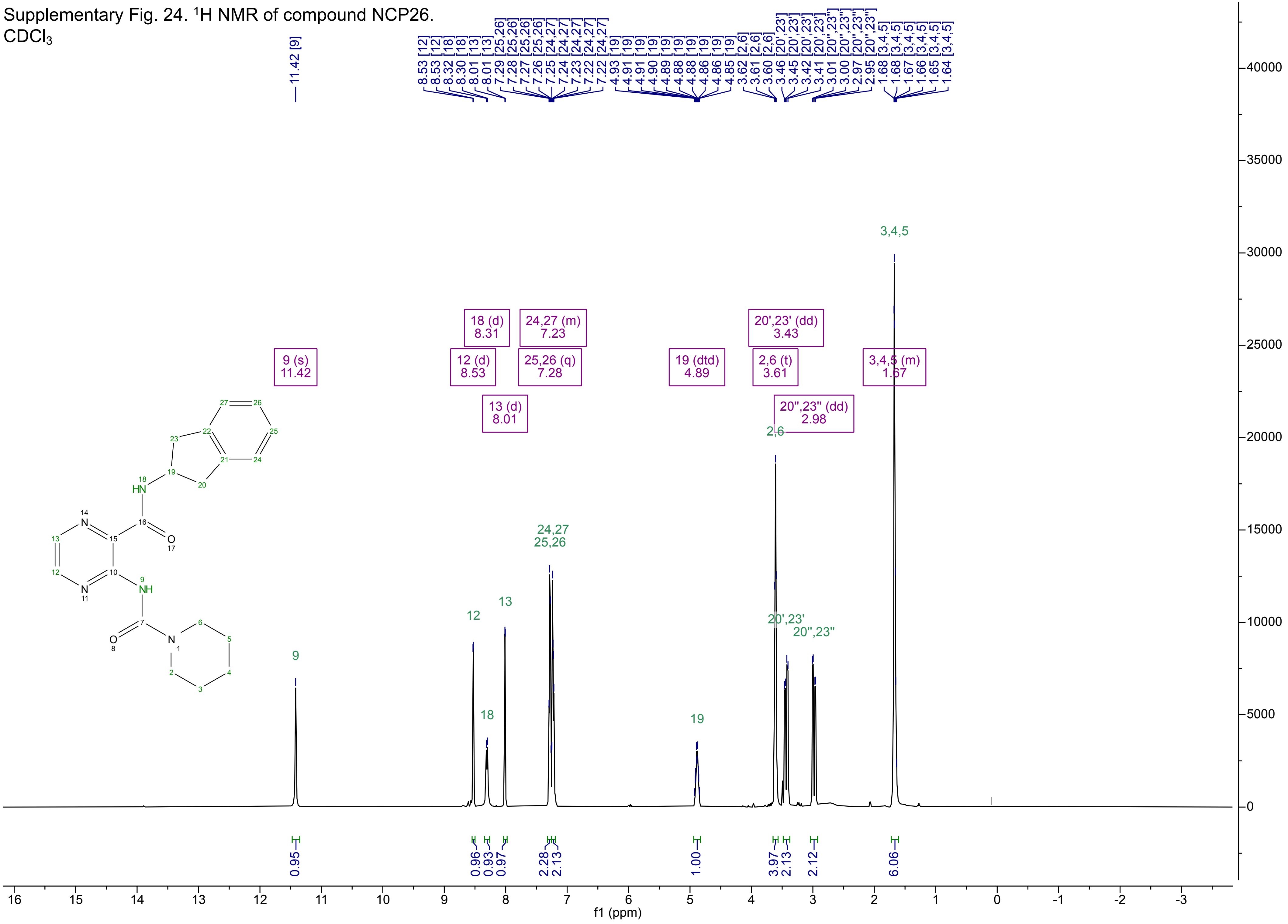

Supplementary Fig. 25. ^{13}C NMR of compound NCP26.
$CDCl_3$

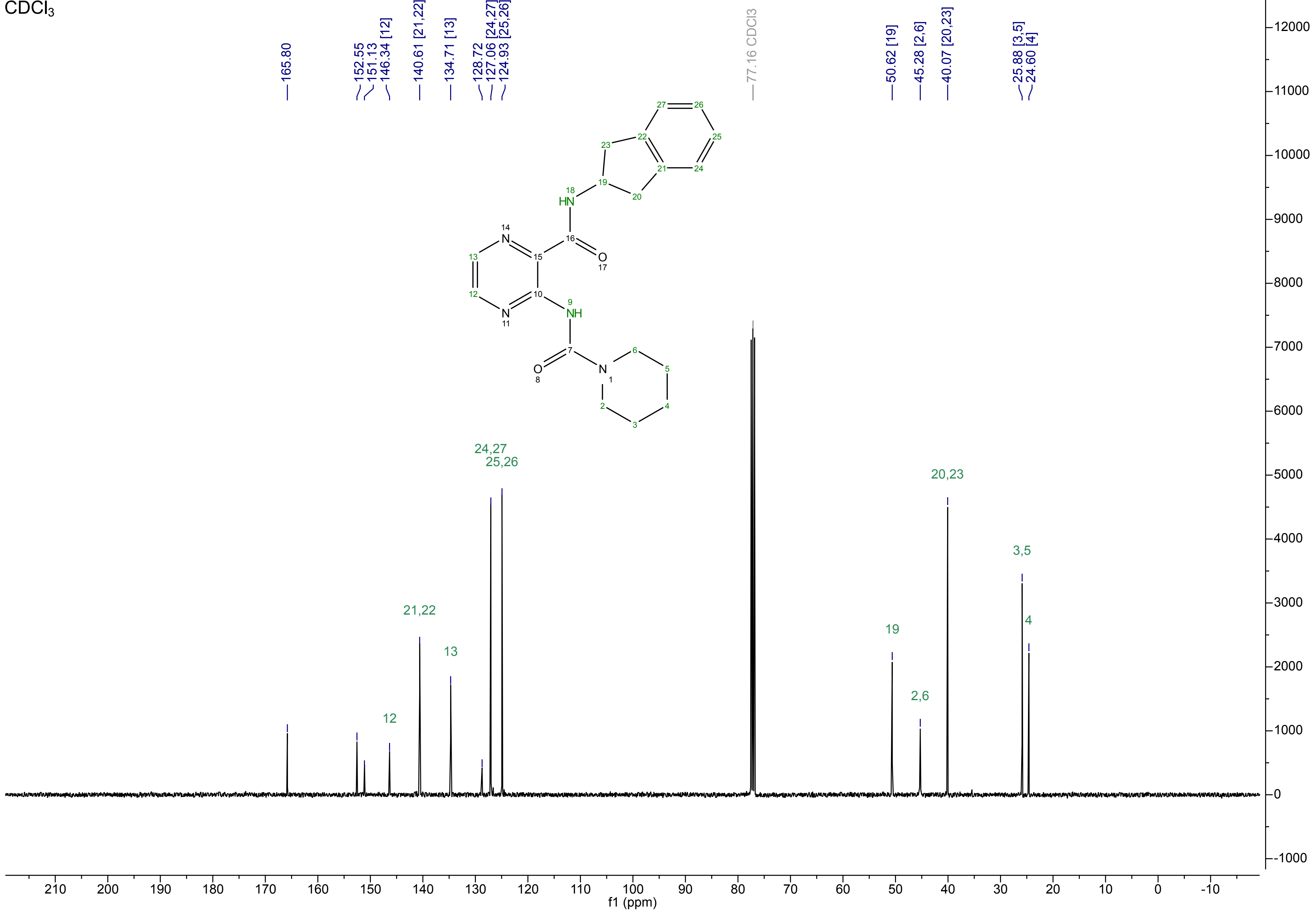

Supplementary Fig. 26. ^{1}H NMR of compound **4**.
$CDCl_3$

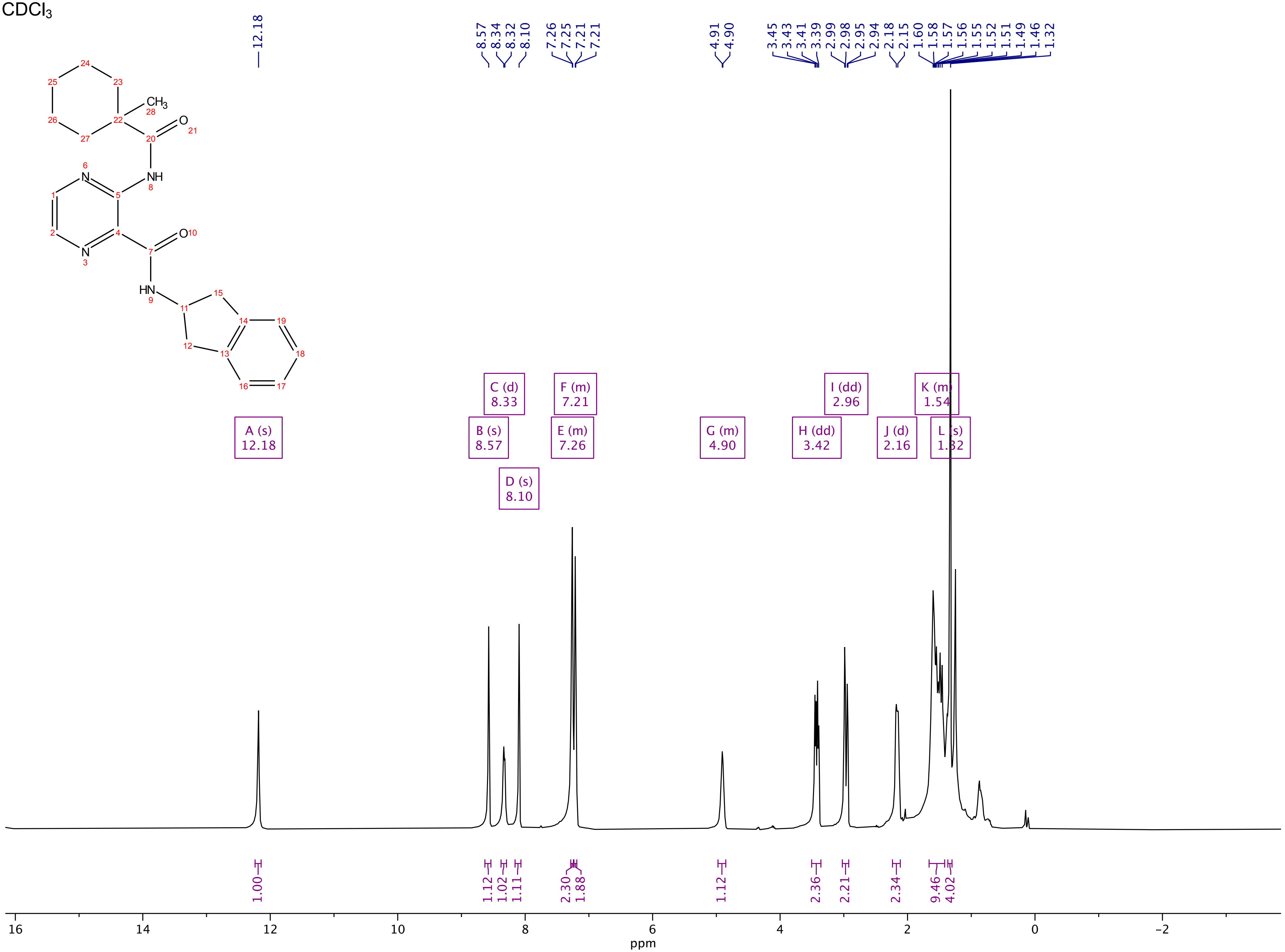

Supplementary Fig. 27. ^{13}C NMR of compound **4**.
$CDCl_3$

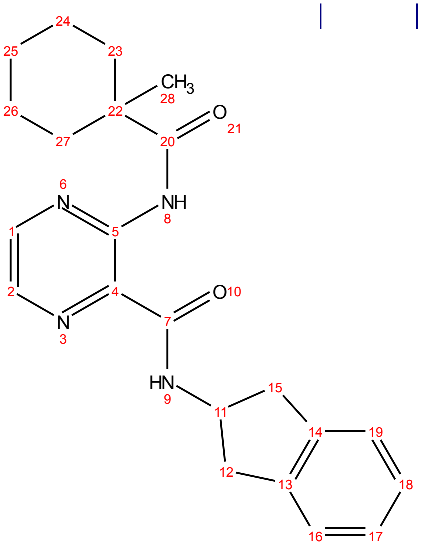

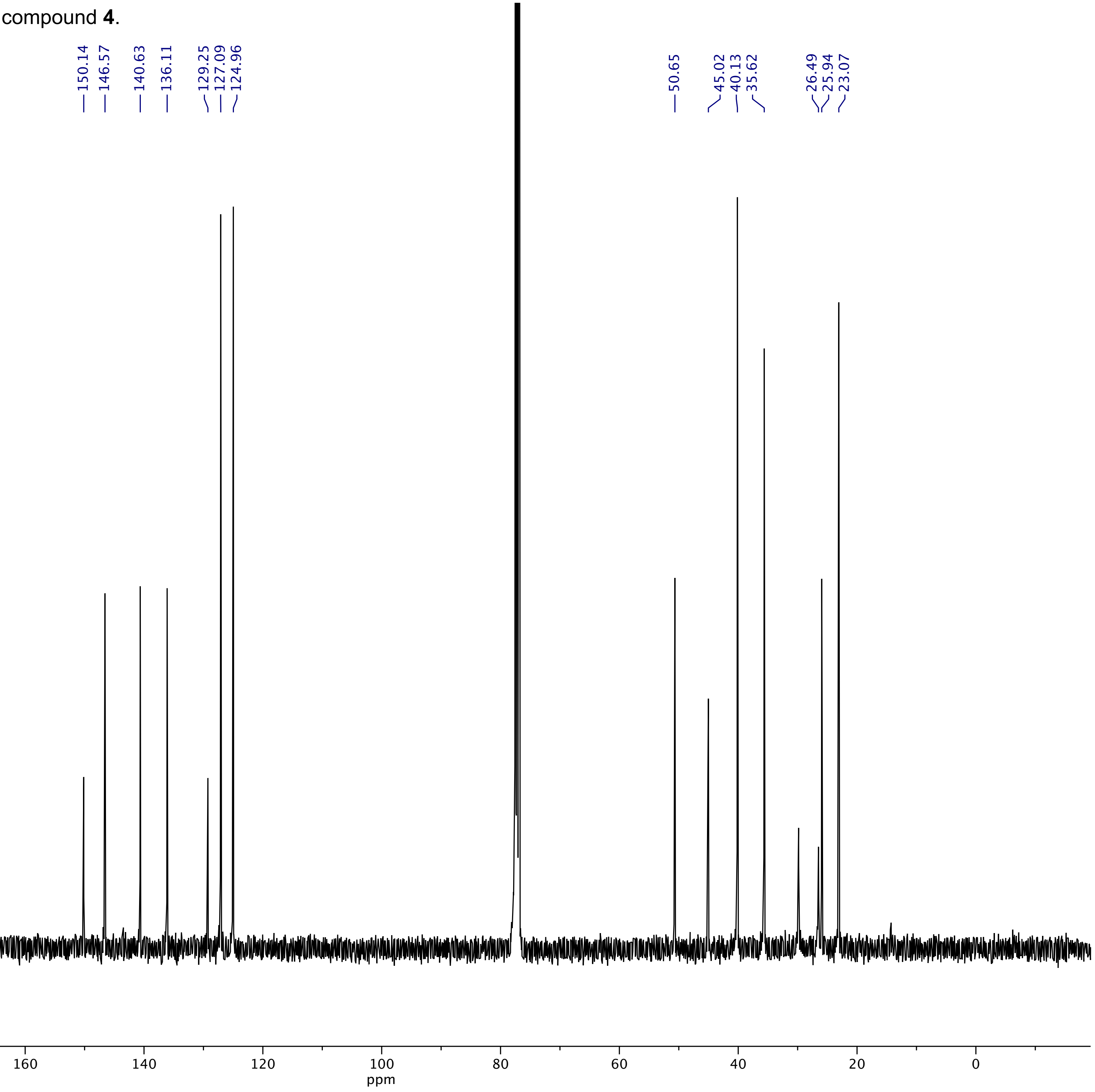

Supplementary Fig. 28. ^{1}H NMR of compound **5**.
$CDCl_3$

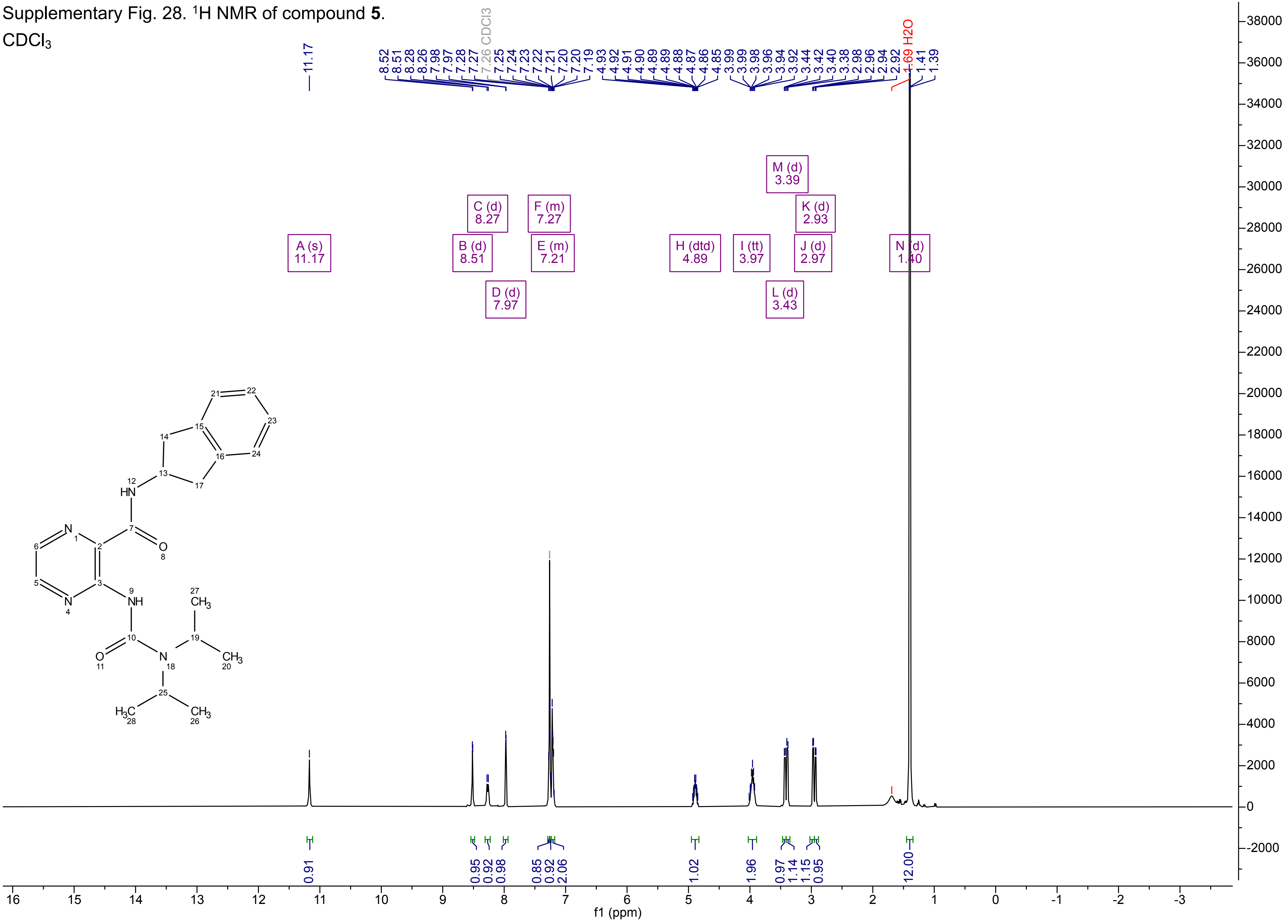

Supplementary Fig. 29. ^{13}C NMR of compound **5**.

$CDCl_3$

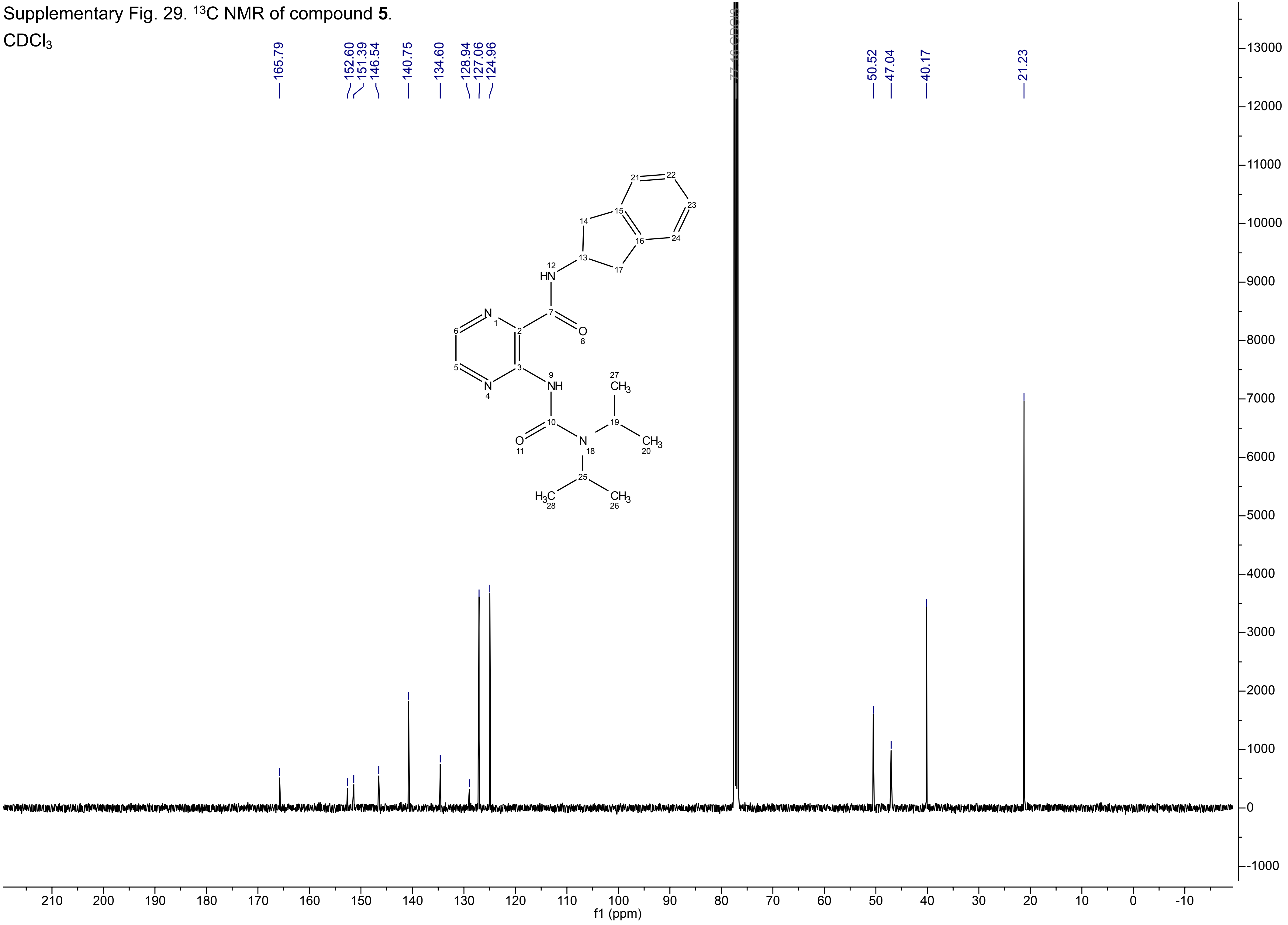

Supplementary Fig. 30. ^{1}H NMR of compound **6**.

$CDCl_3$

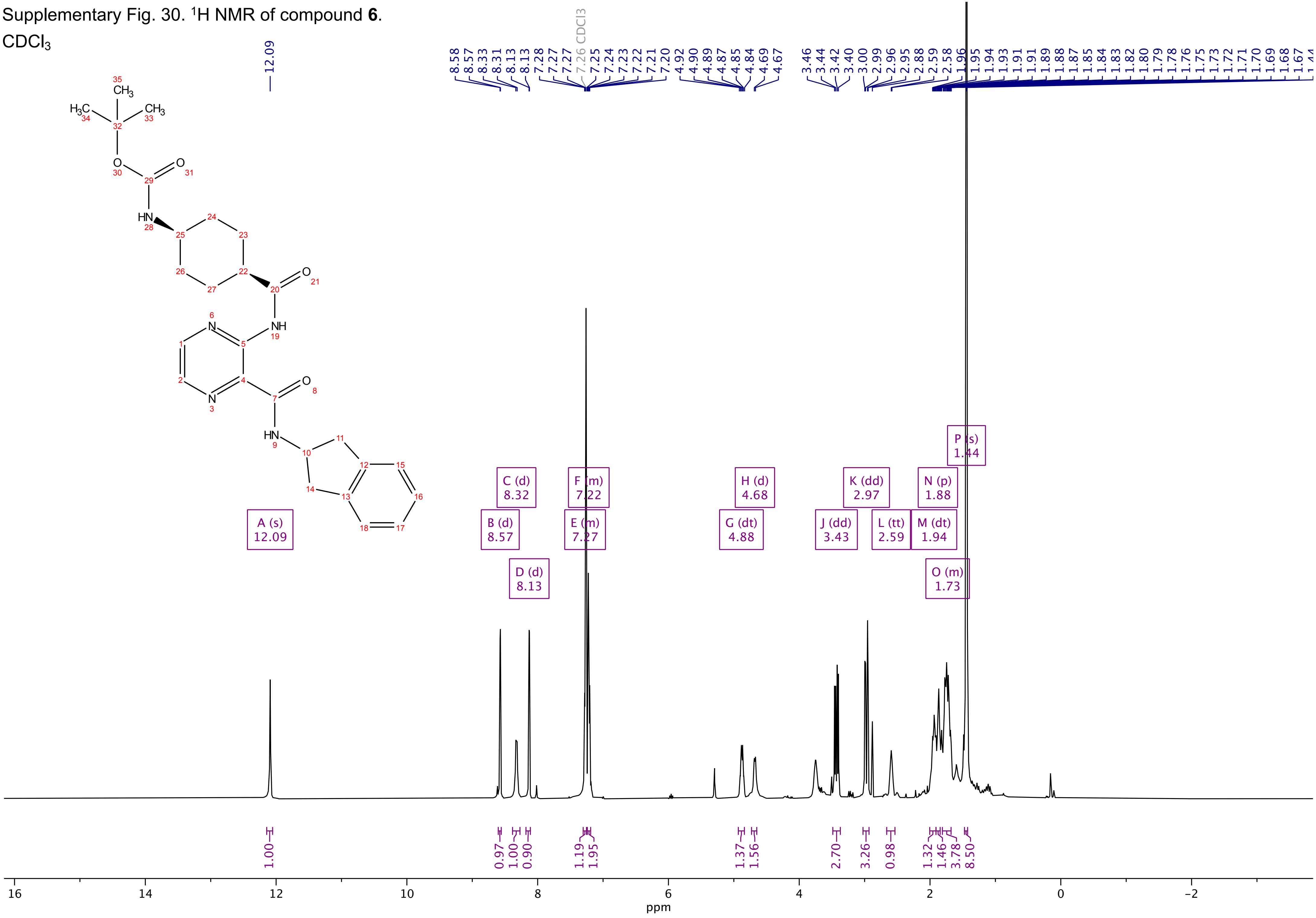

Supplementary Fig. 31. ^{13}C NMR of compound **6**.

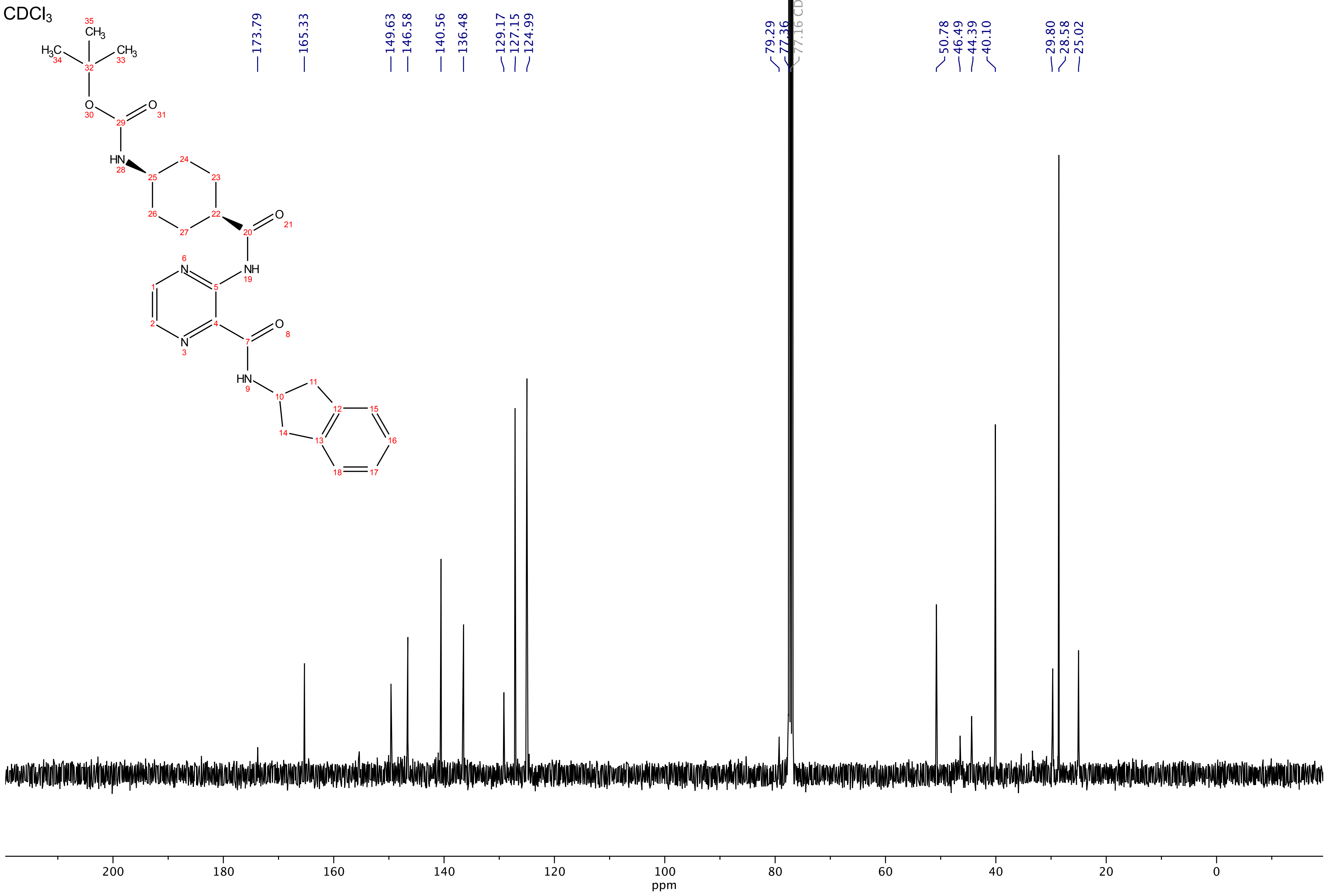

Supplementary Fig. 32. ^{1}H NMR of compound **7**.

$CDCl_3$

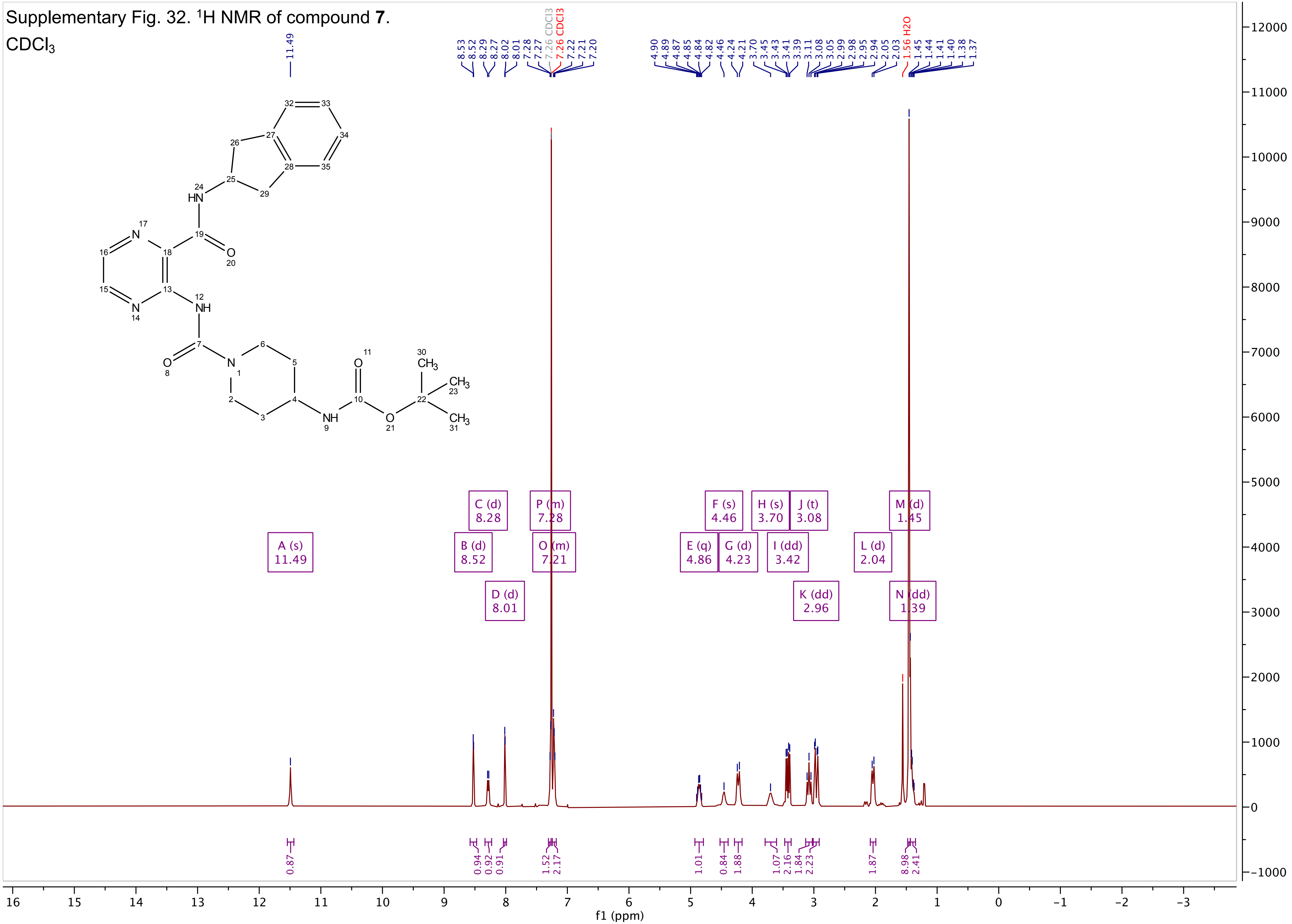

Supplementary Fig. 33. ^{13}C NMR of compound **7**.

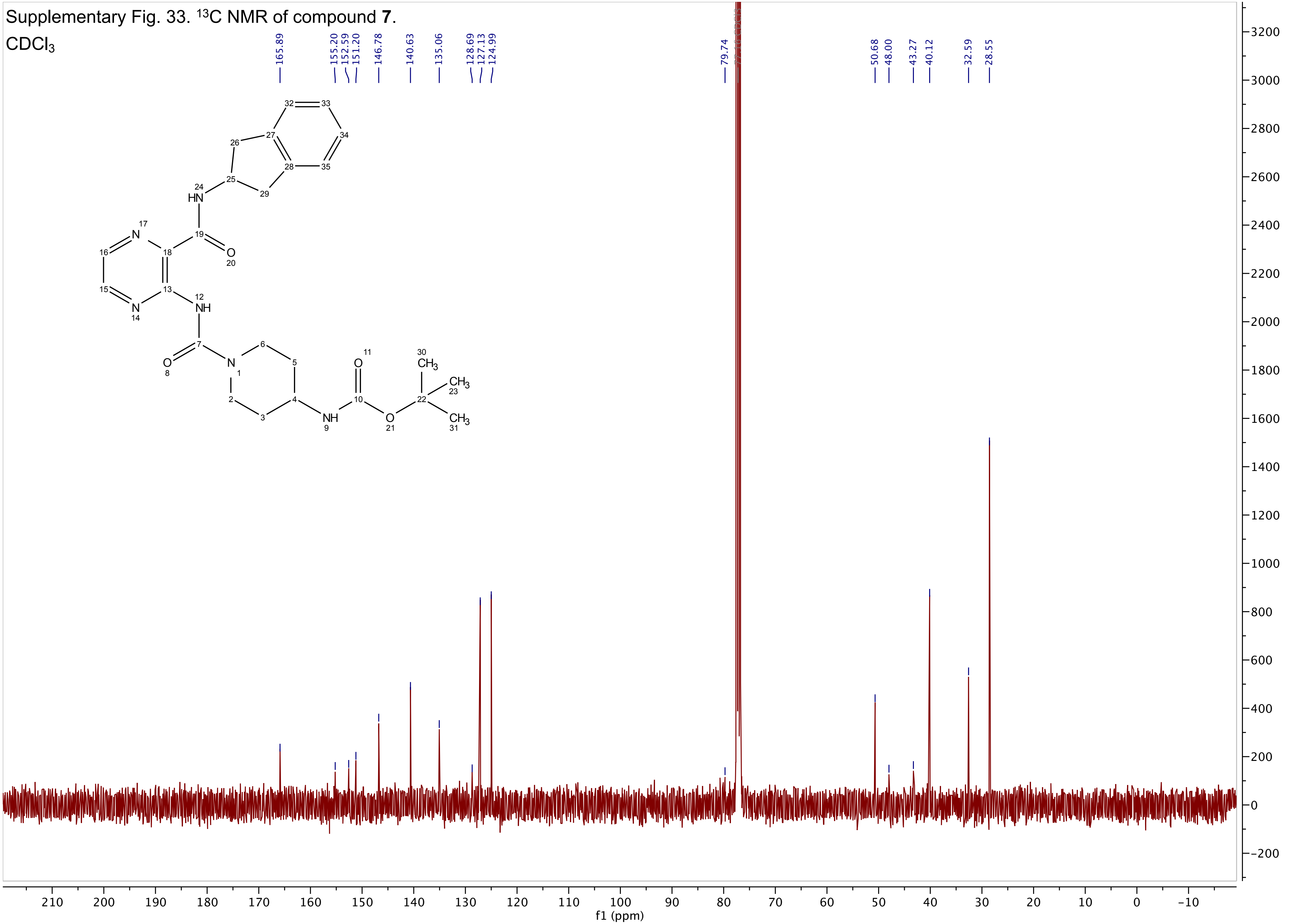

Supplementary Fig. 34. ^{1}H NMR of compound **8**.

d$_6$DMSO

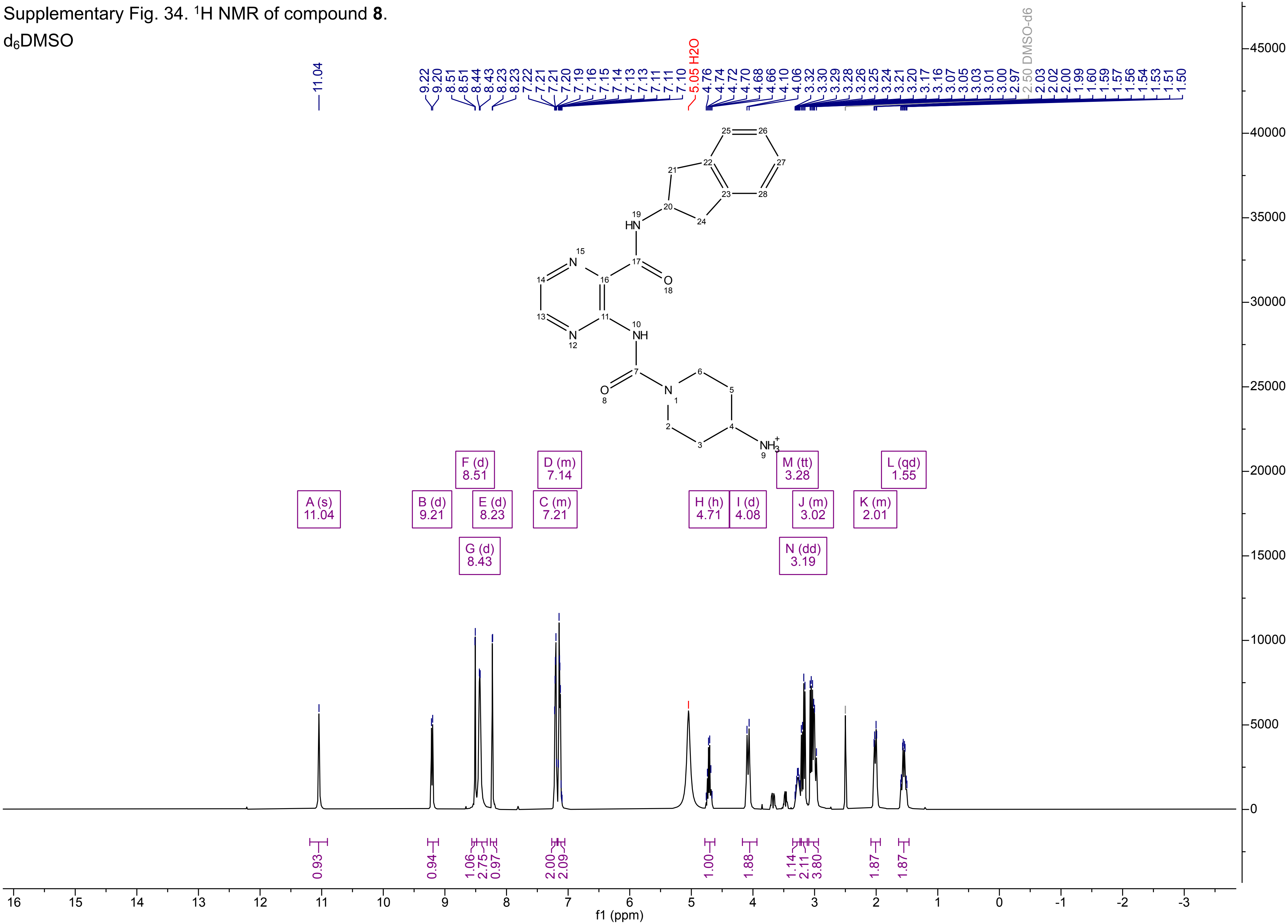

Supplementary Fig. 35. ^{13}C NMR of compound **8**.

d_6DMSO

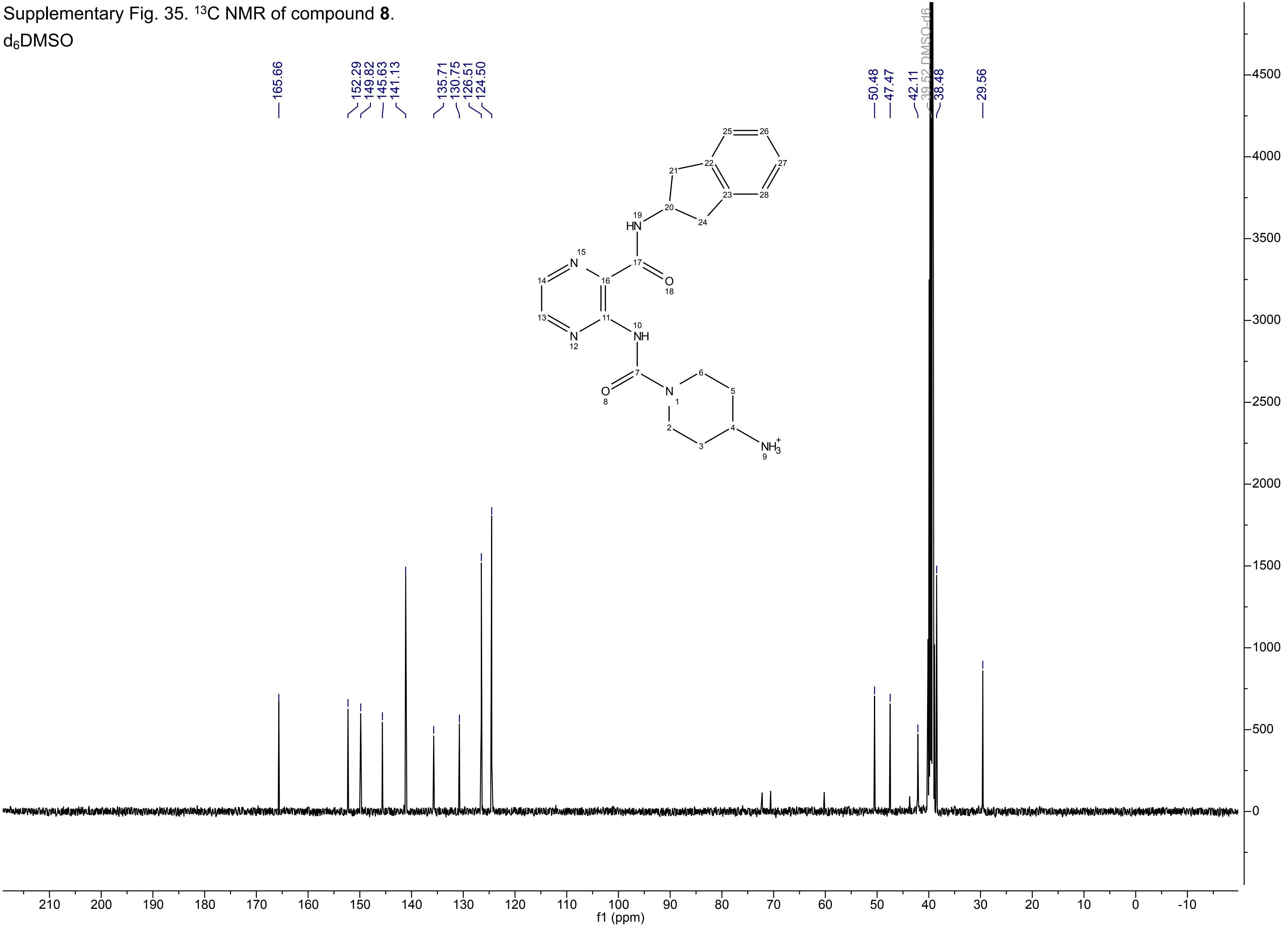

Supplementary Fig. 36. ^1H NMR of compound **9**.
$CDCl_3$

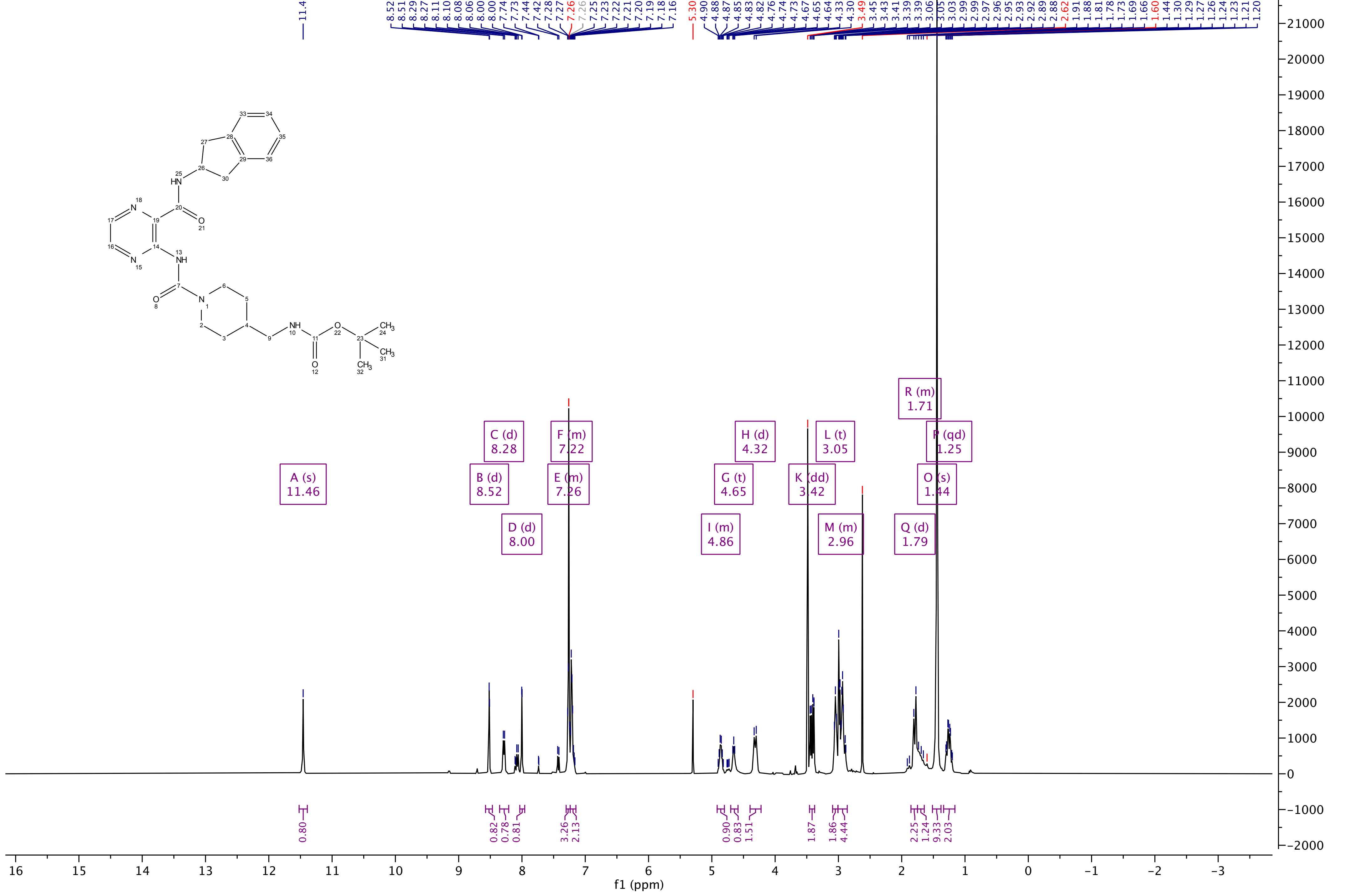

Supplementary Fig. 37. ^{13}C NMR of compound **9**.
$CDCl_3$

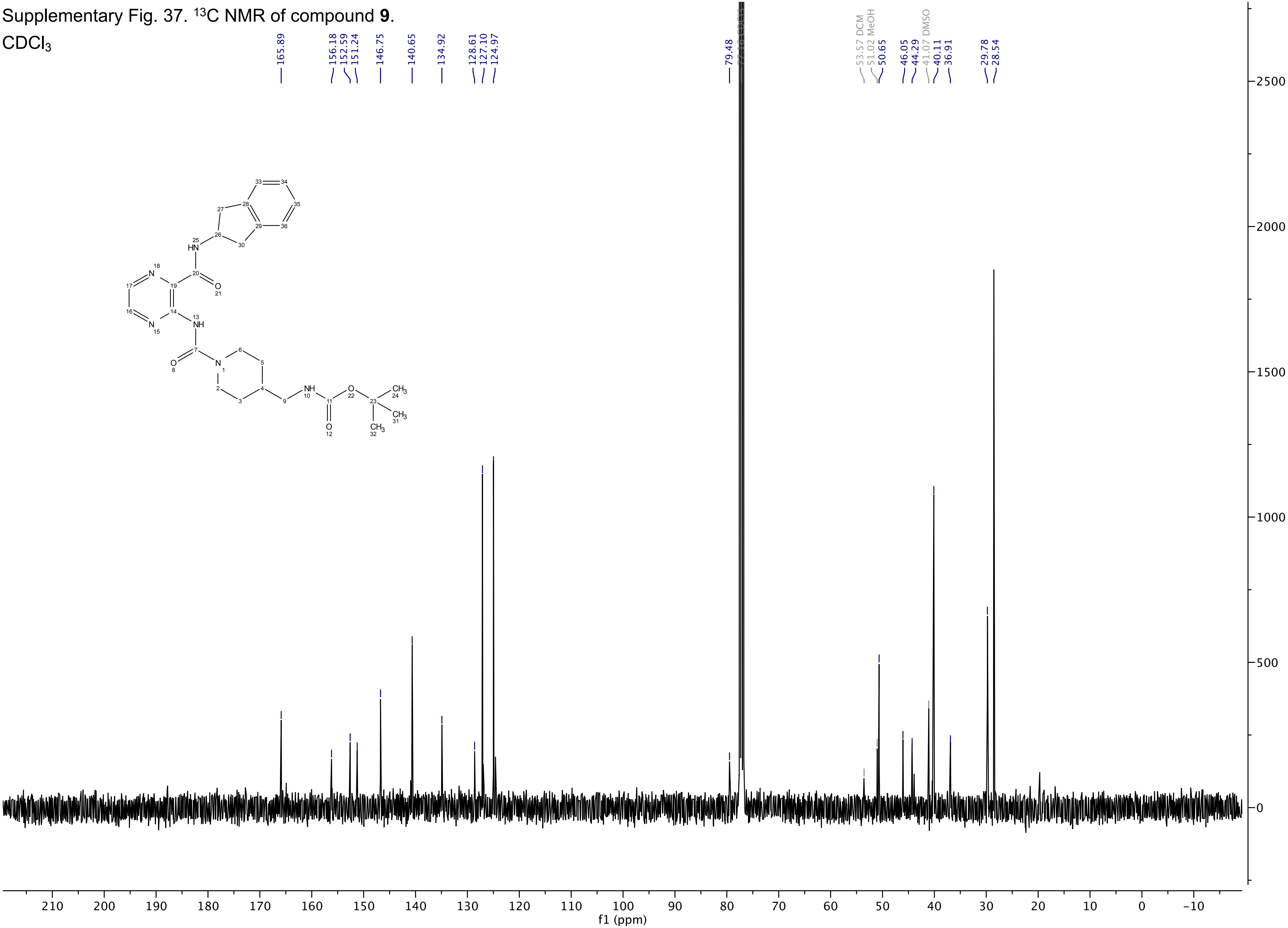

Supplementary Fig. 38. ^{1}H NMR of compound **10**.
d_6DMSO

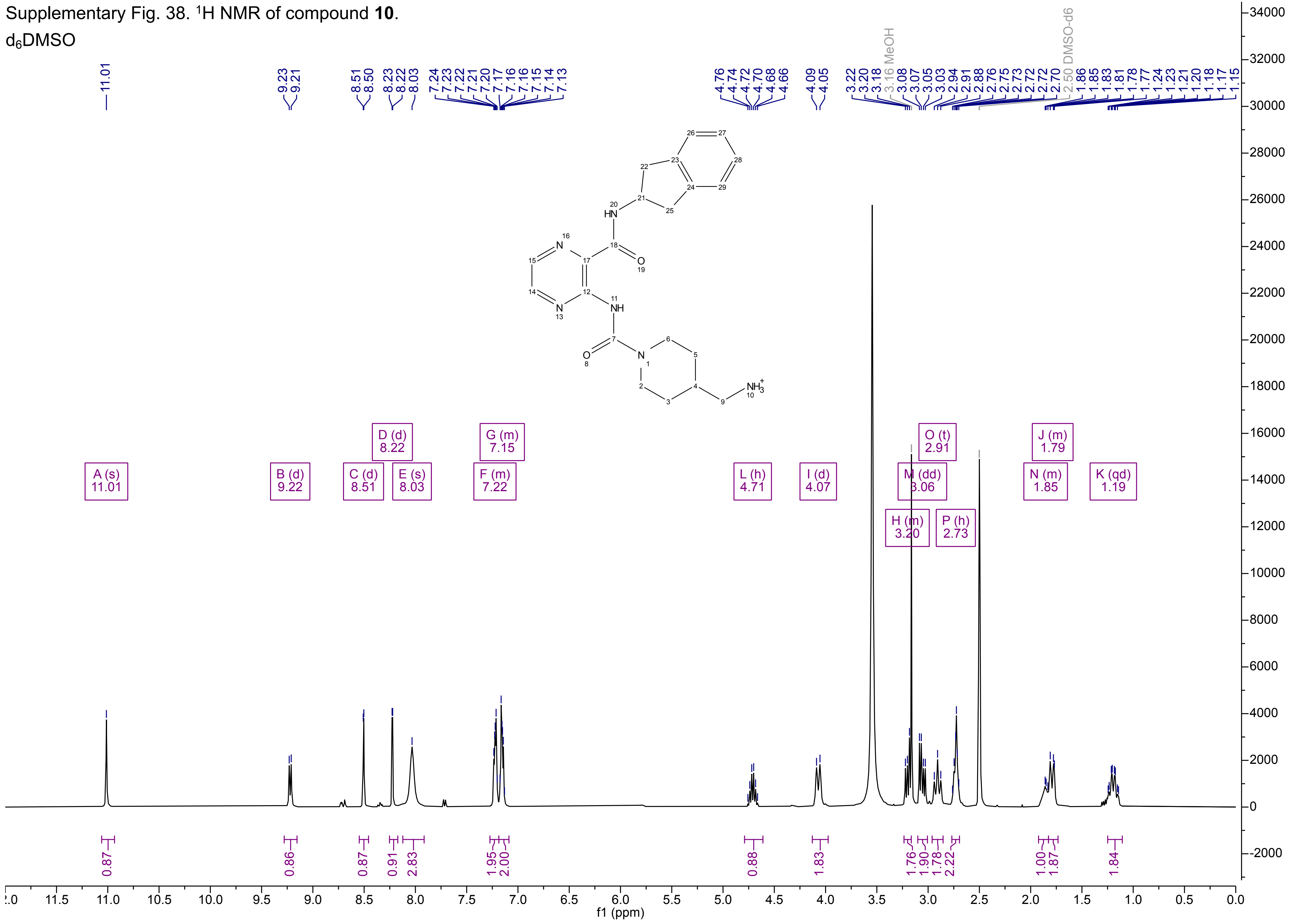

Supplementary Fig. 39. ^{13}C NMR of compound **10**.

d_6DMSO

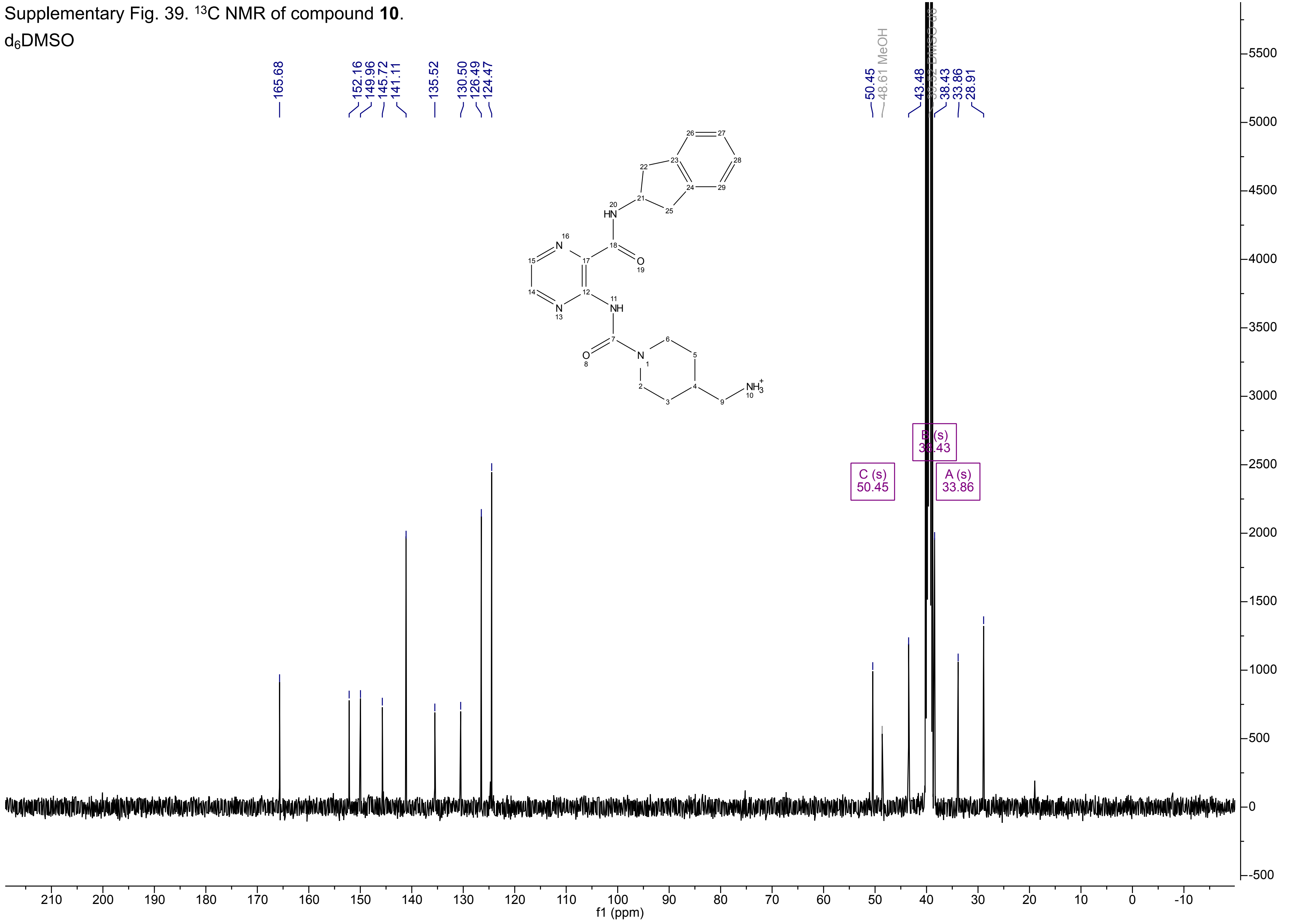

Supplementary Fig. 40. ^{1}H NMR of compound **11**.

$CDCl_3$

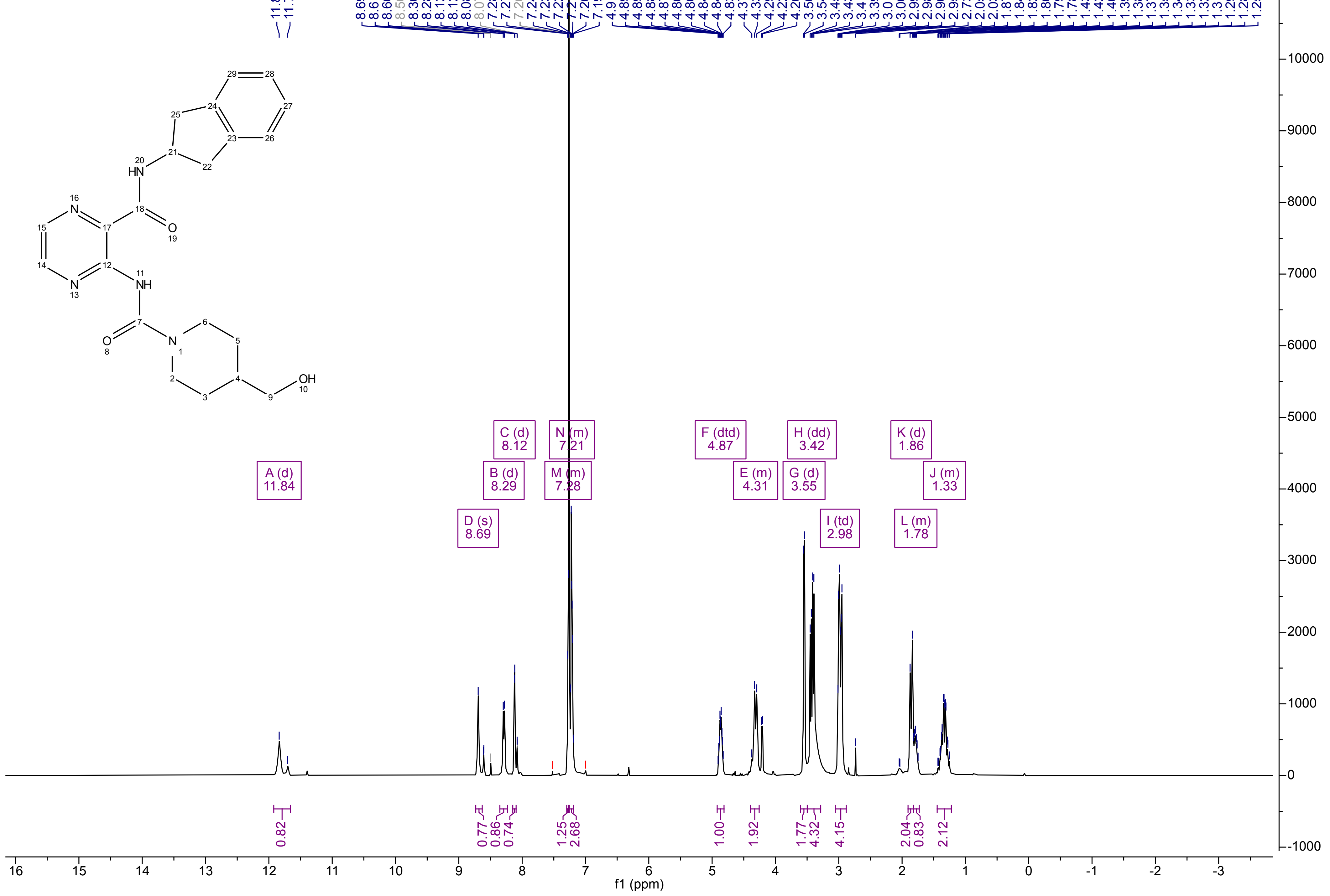

Supplementary Fig. 41. ^{13}C NMR of compound **11**.

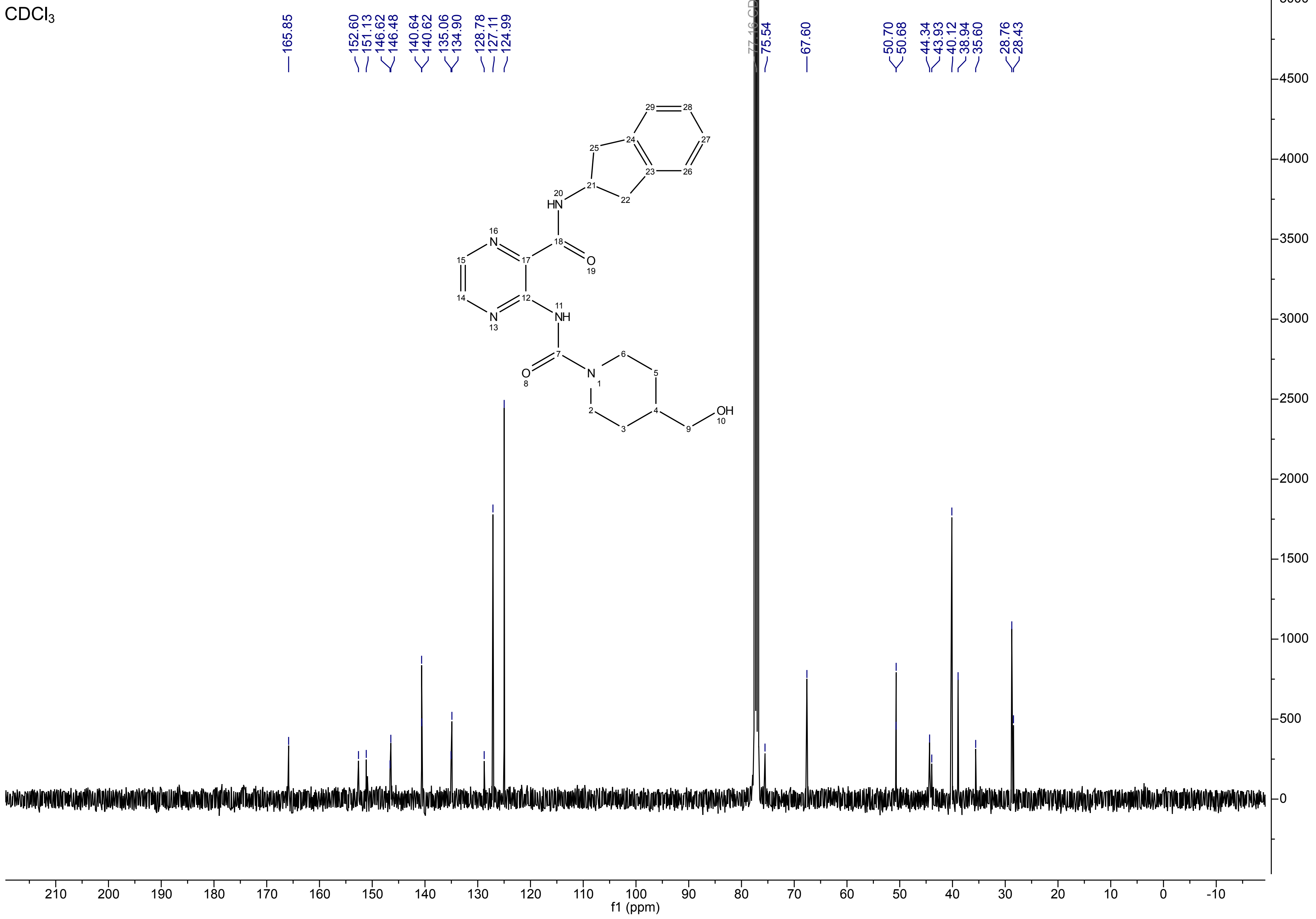

Supplementary Fig. 42. ^{1}H NMR of compound **12**.

$CDCl_3$

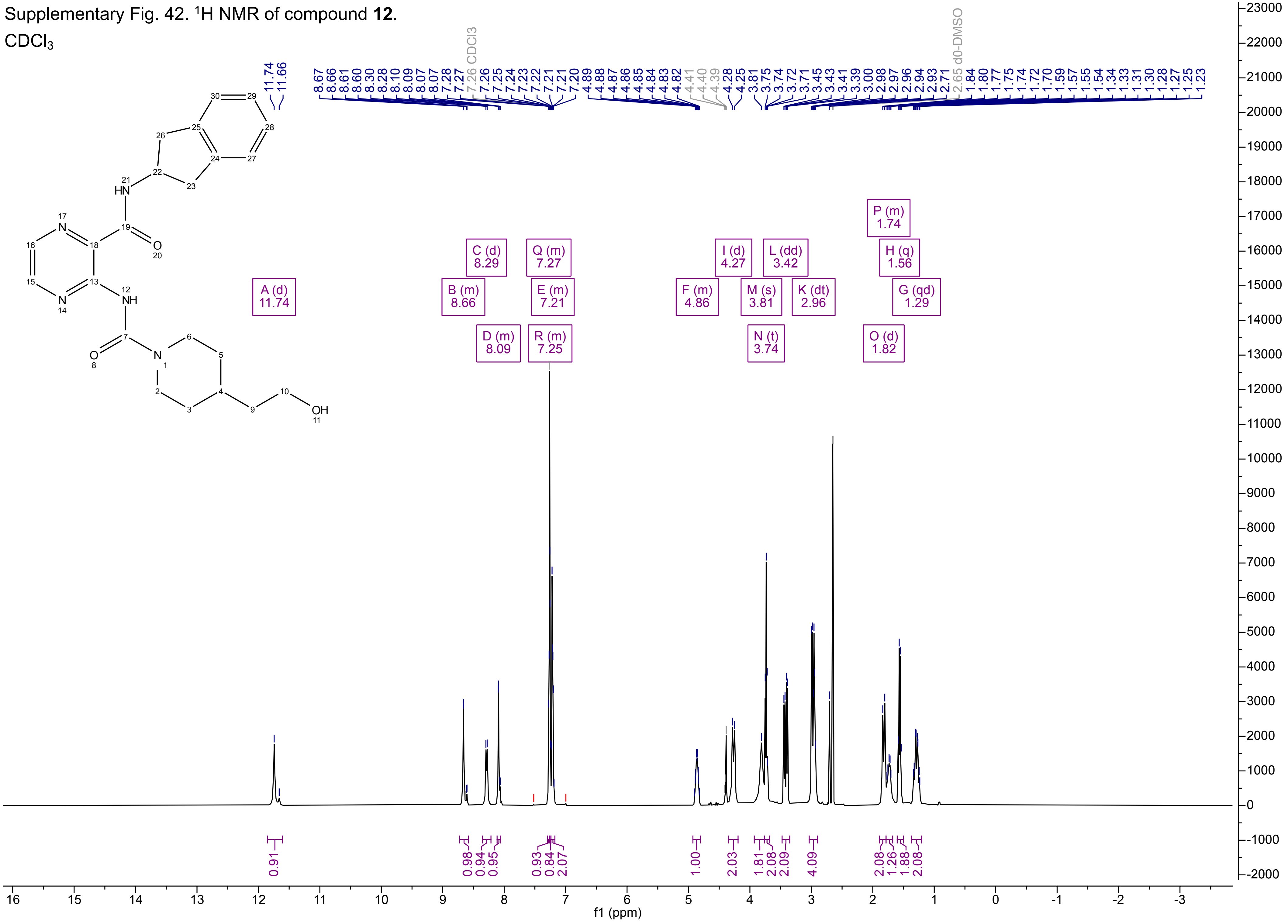

Supplementary Fig. 43. ^{13}C NMR of compound **12**.

$CDCl_3$

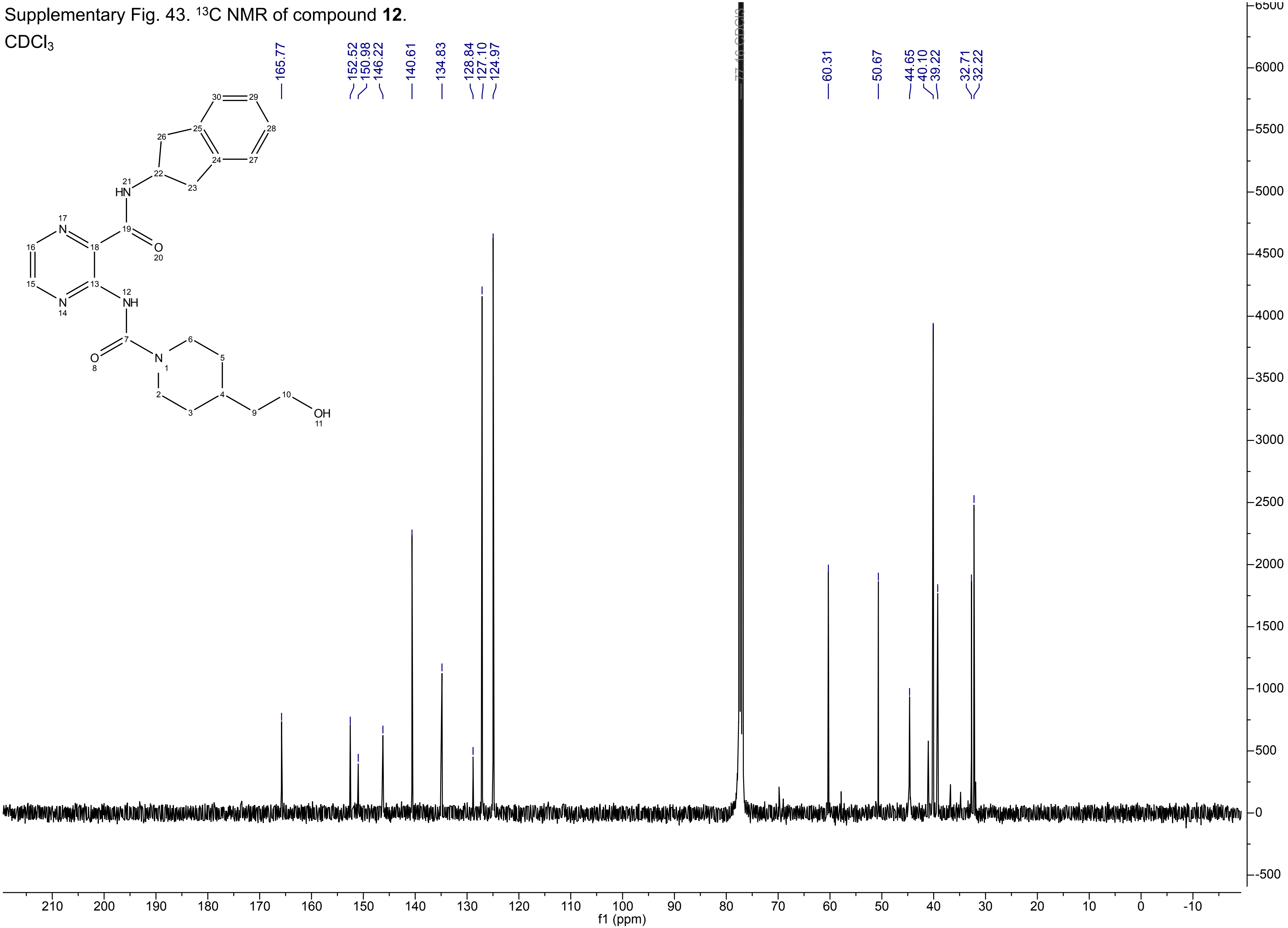

Supplementary Fig. 44. ^{1}H NMR of compound **13**.

$CDCl_3$

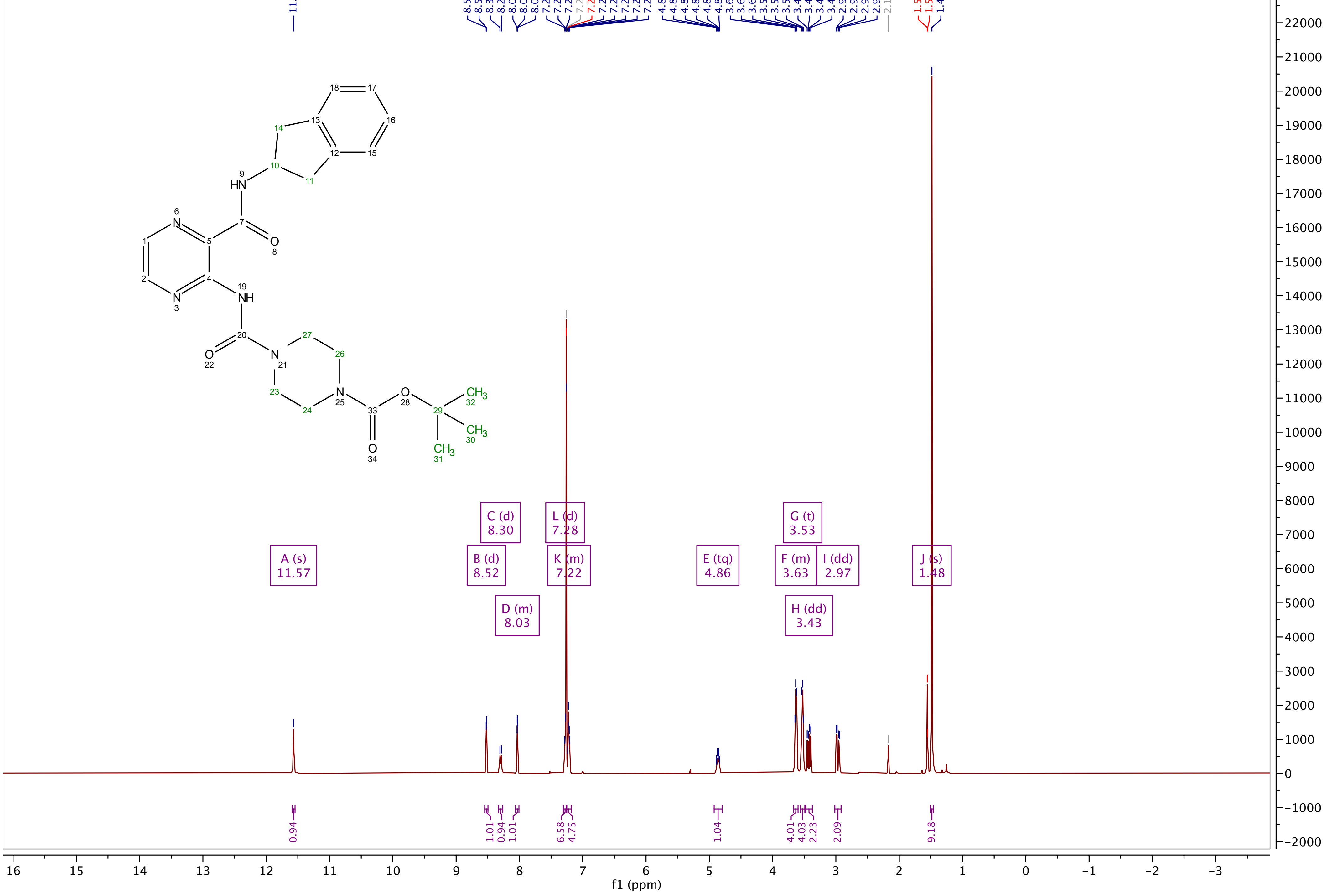

Supplementary Fig. 45. ^{13}C NMR of compound **13**.
$CDCl_3$

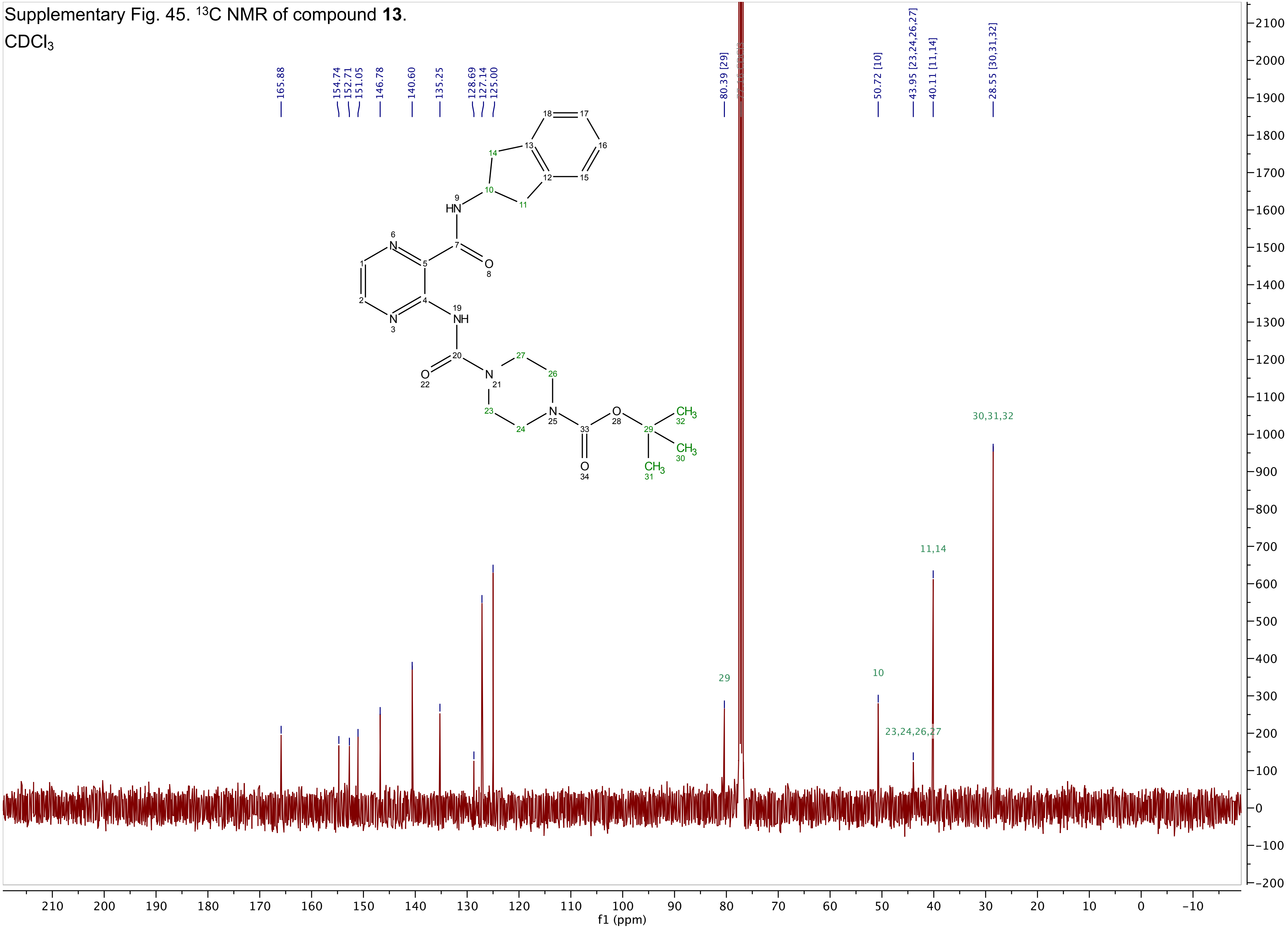

Supplementary Fig. 46. ^{1}H NMR of compound **14**.

d$_6$DMSO

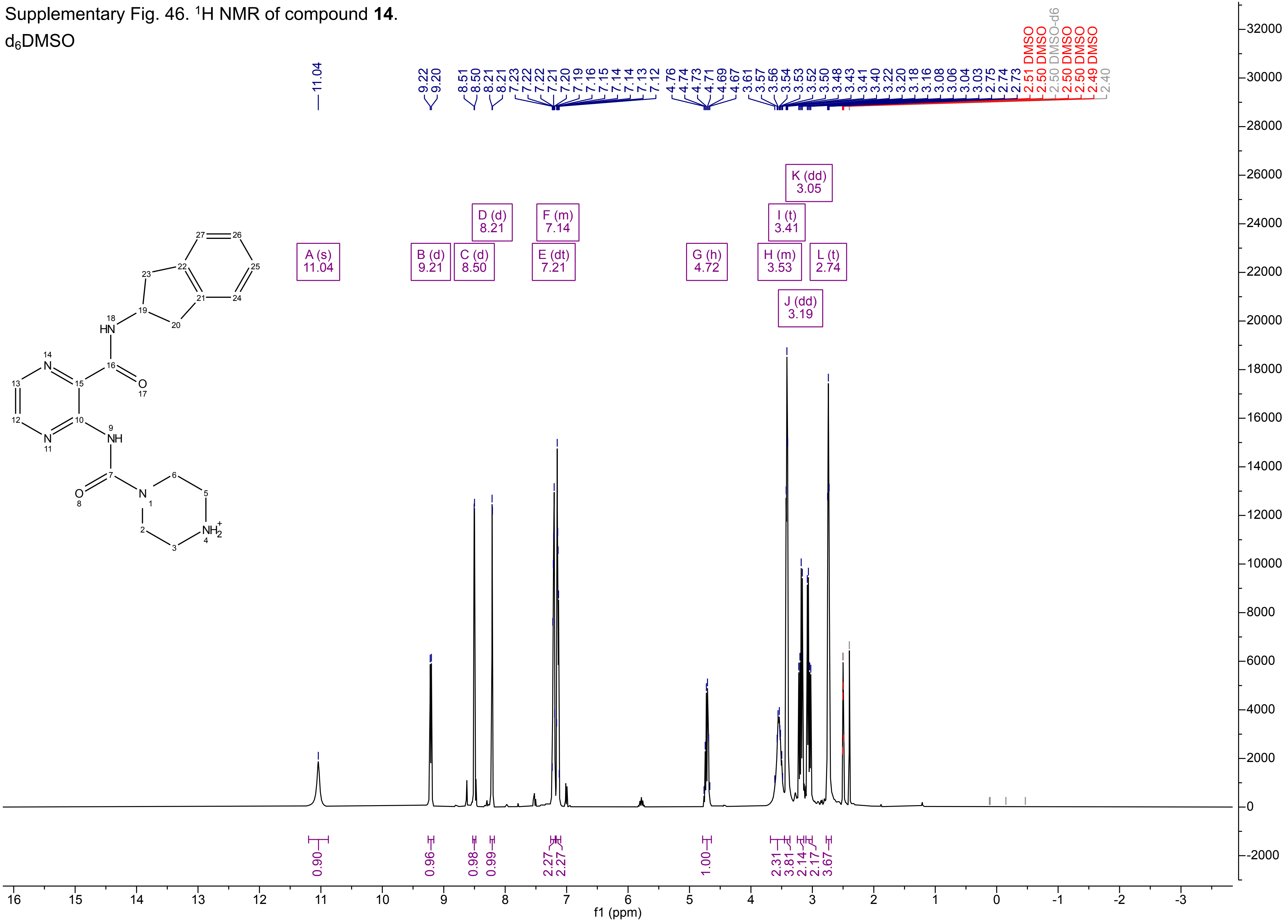

Supplementary Fig. 47. ^{13}C NMR of compound **14**.

d_6DMSO

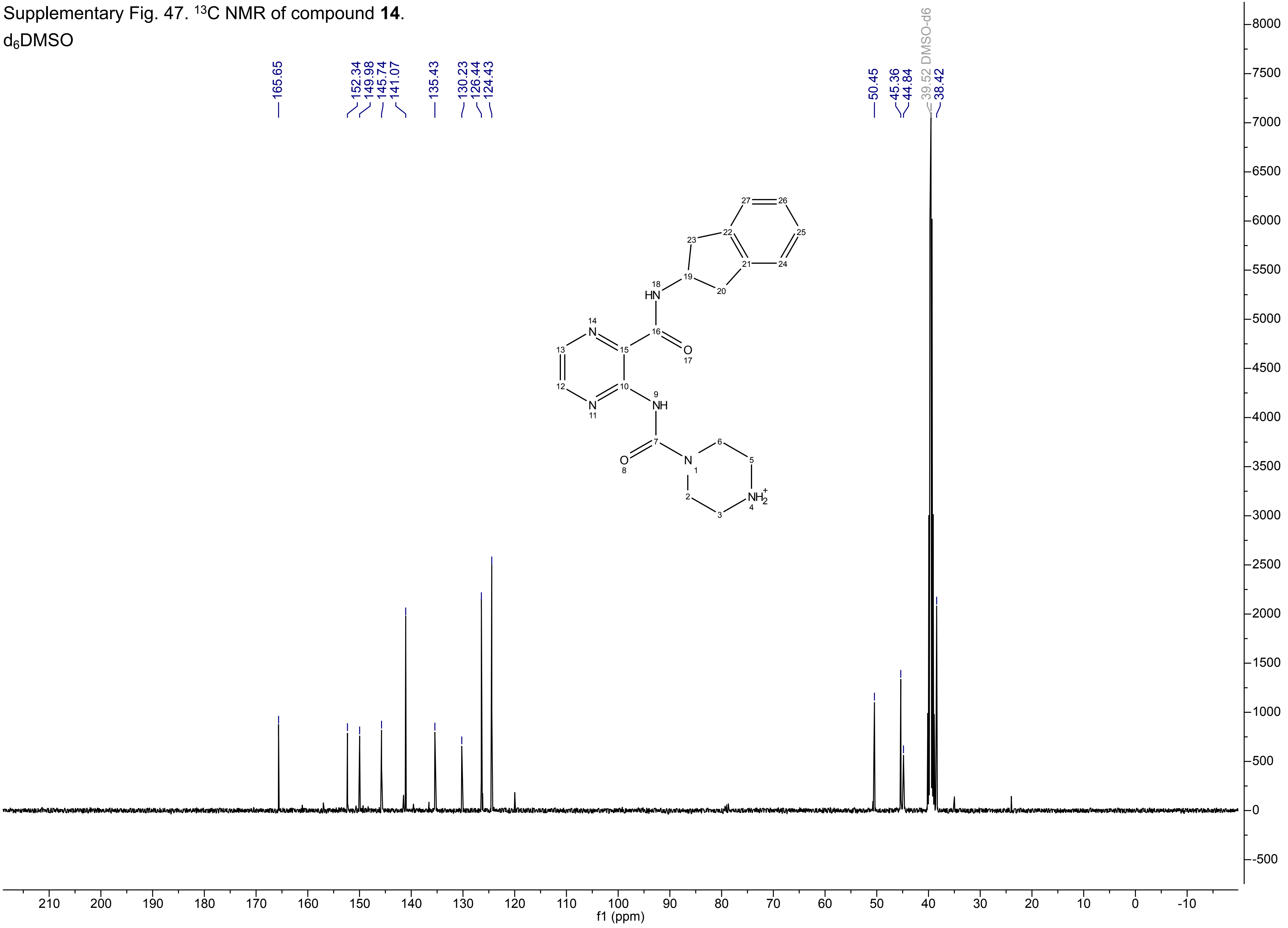

Supplementary Fig. 48. ^{1}H NMR of compound **15**.

$CDCl_3$

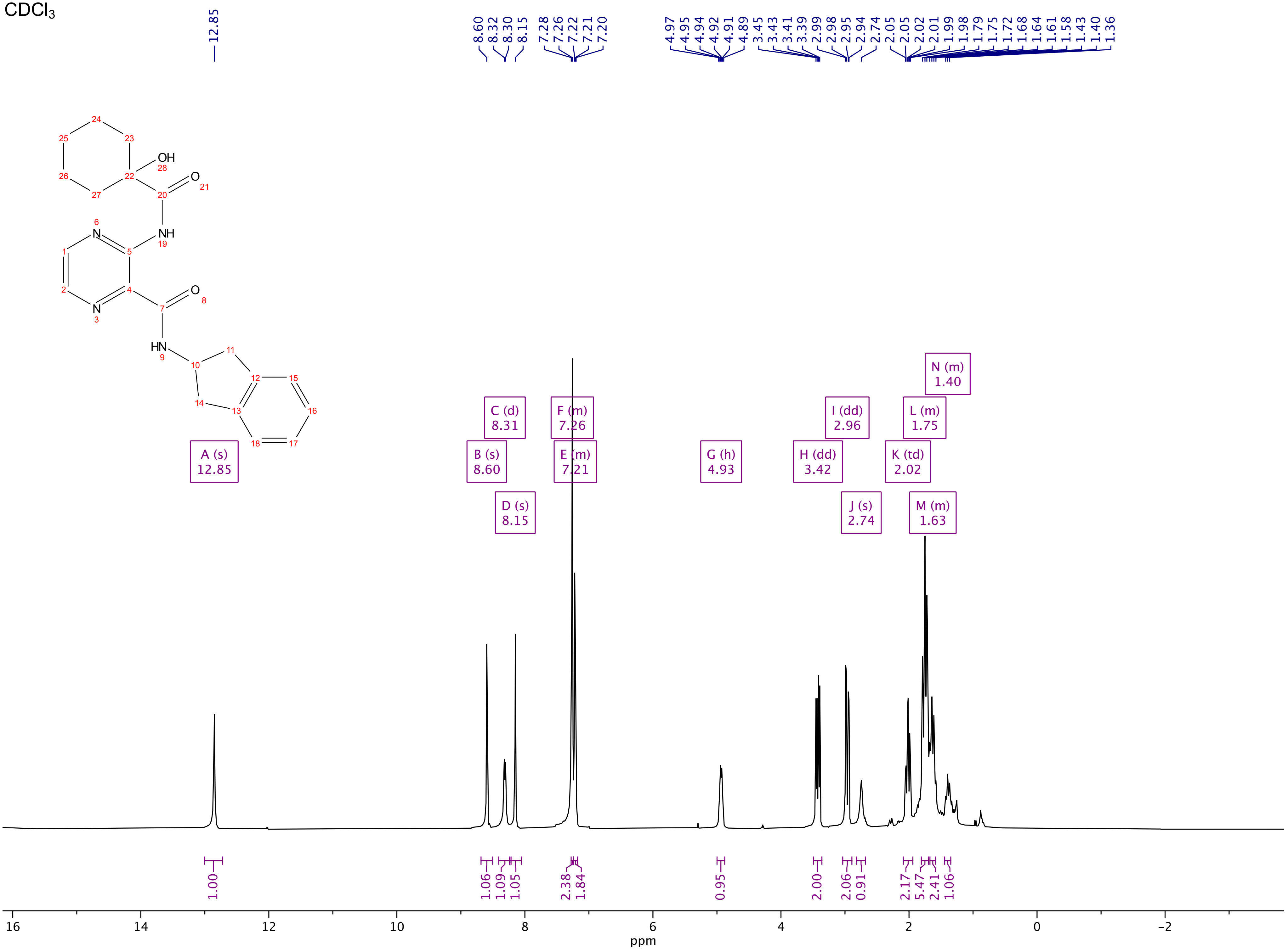

Supplementary Fig. 49. ^{13}C NMR of compound **15**.

$CDCl_3$

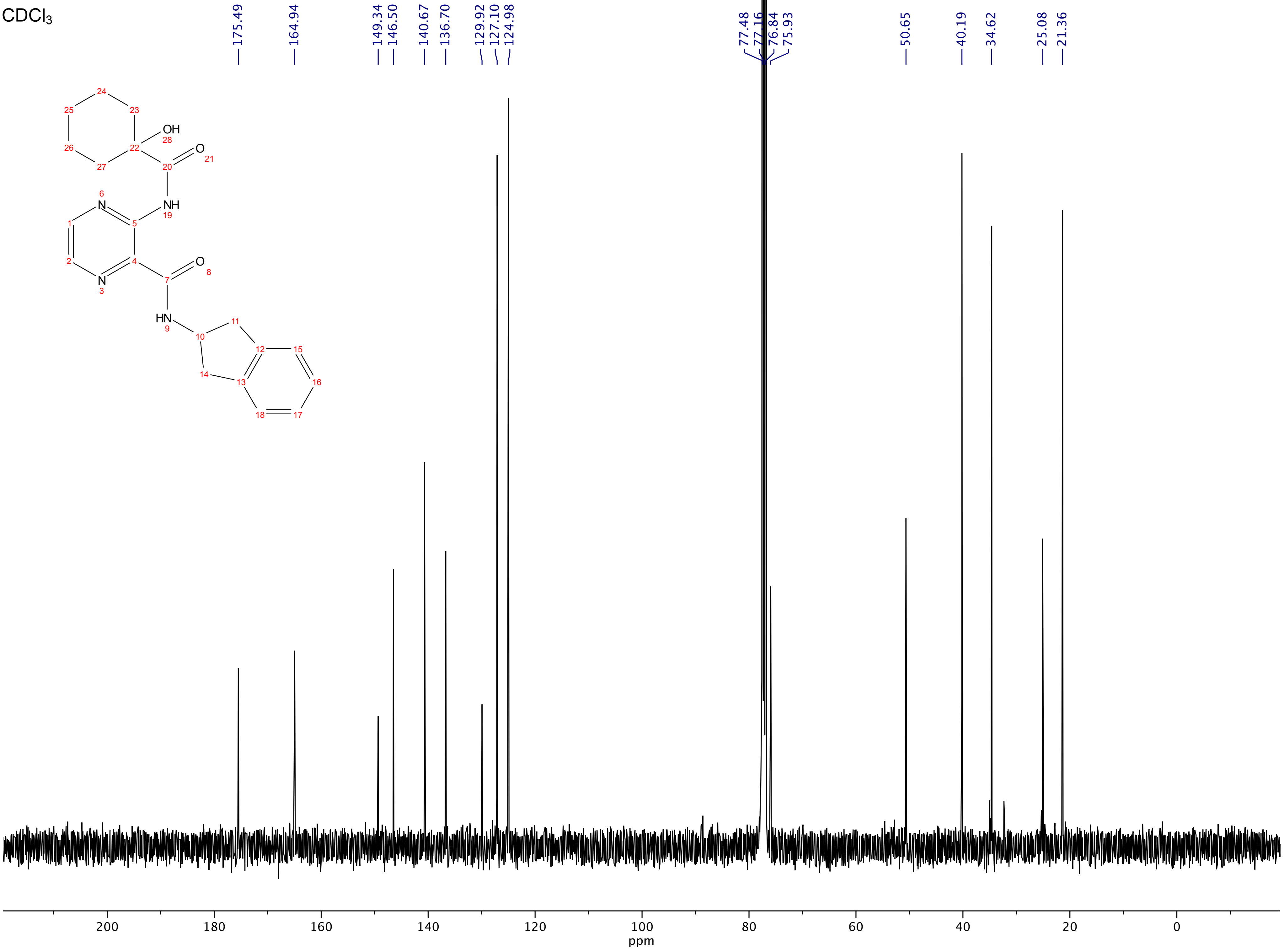

Supplementary Fig. 50. ^{1}H NMR of compound **16**.

$CDCl_3$

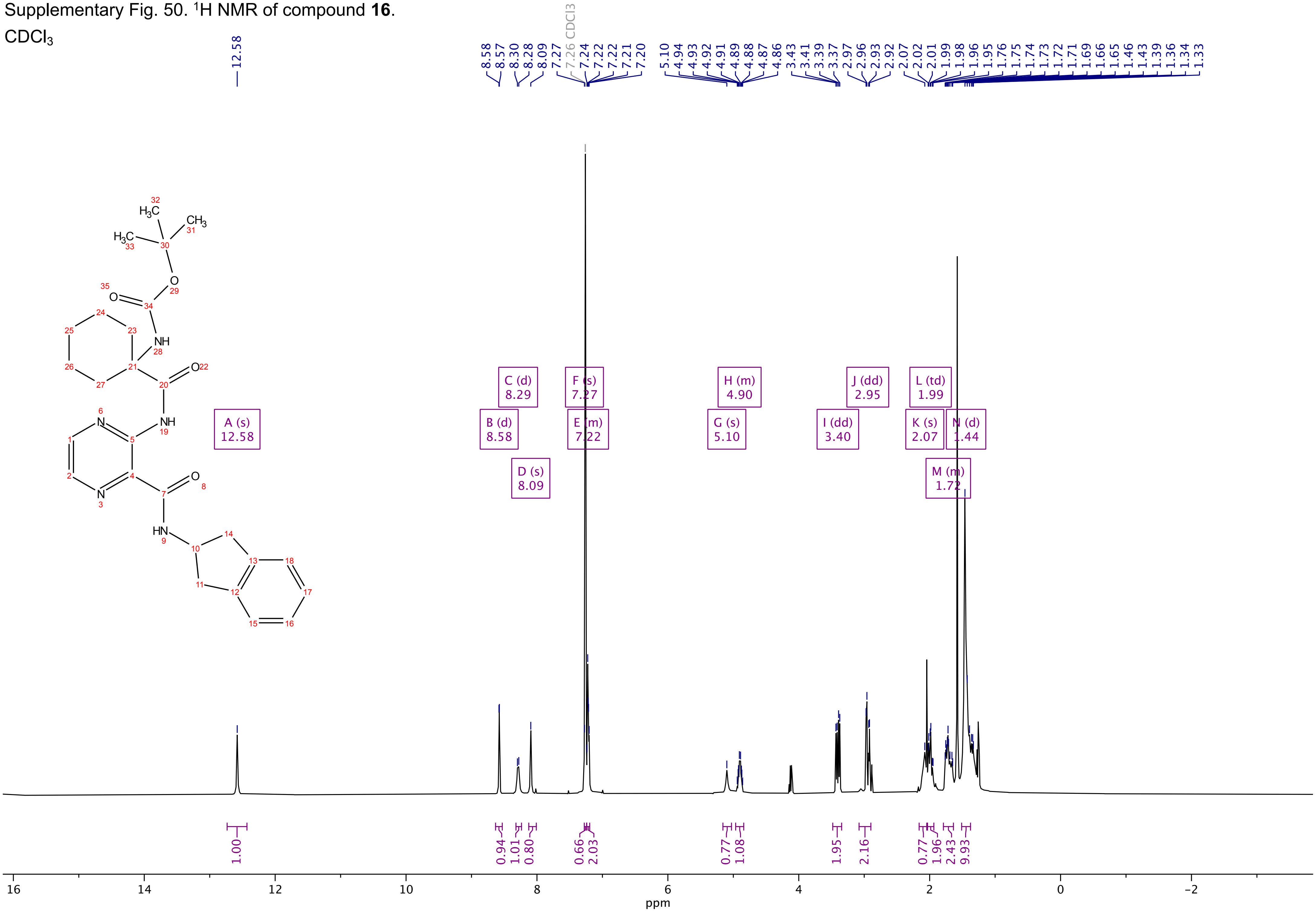

Supplementary Fig. 51. ^{13}C NMR of compound **16**.

$CDCl_3$

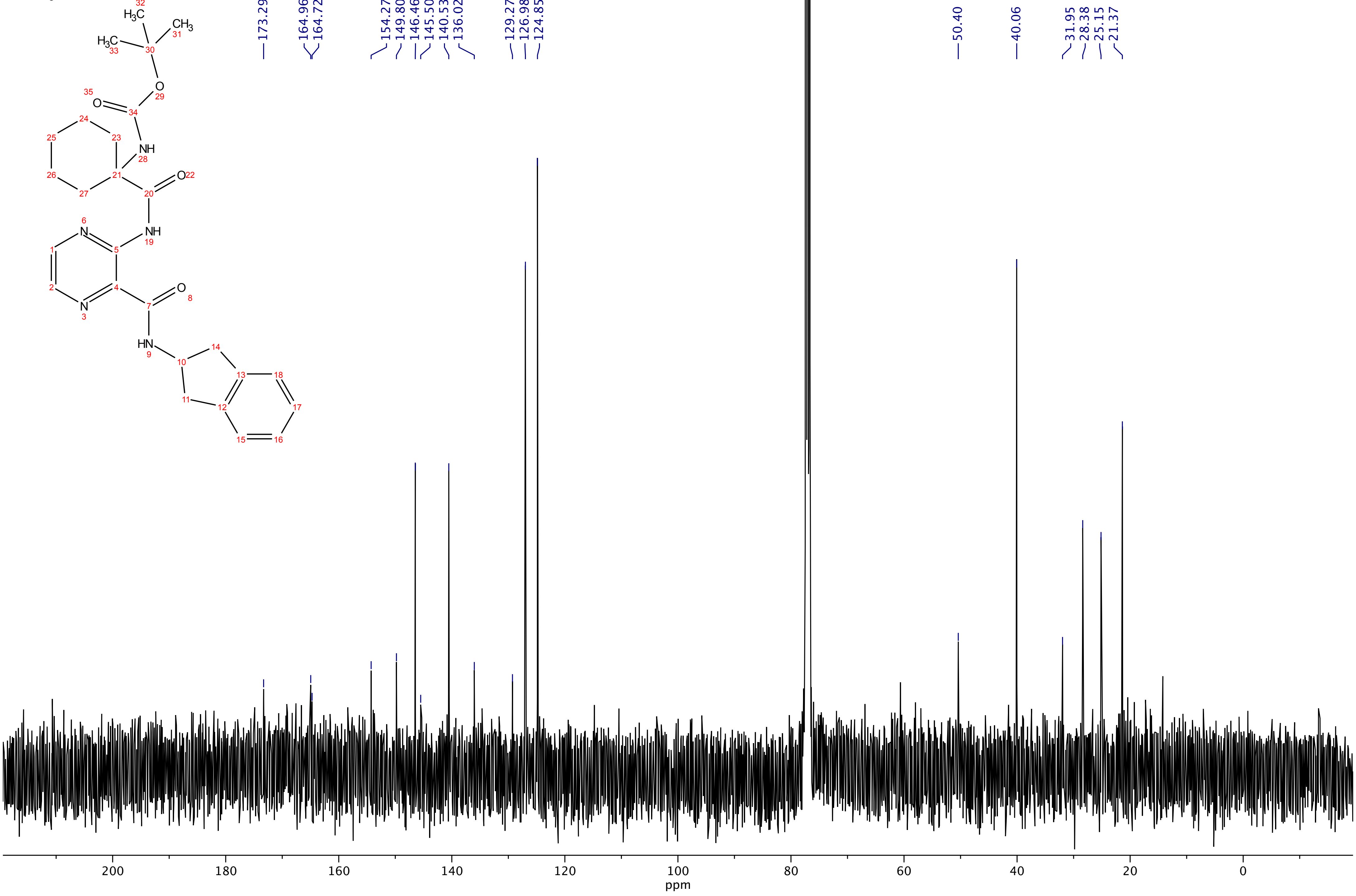

Supplementary Fig. 52. DEPT-135 NMR of compound **16**.

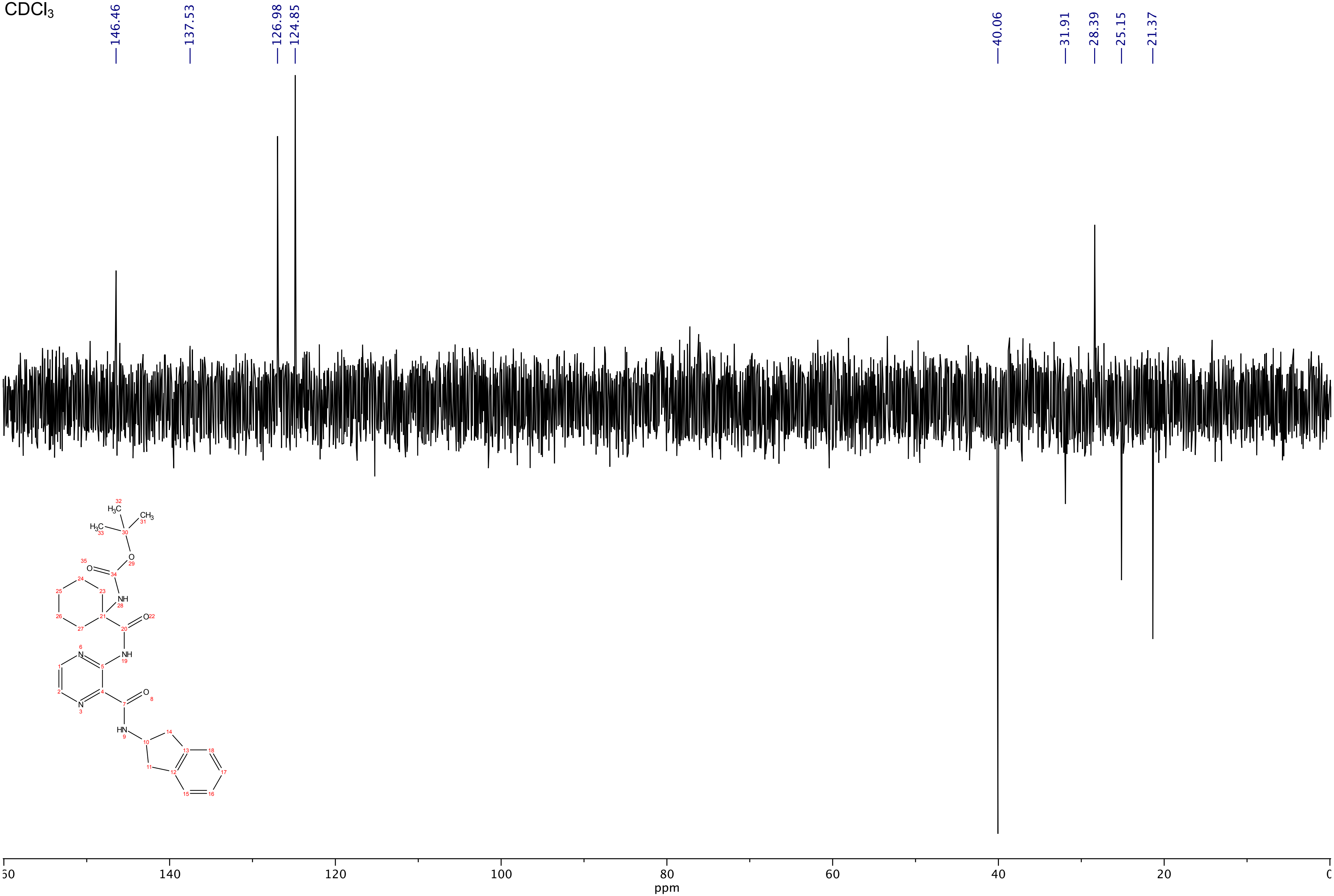

Supplementary Fig. 53. ^{1}H-^{1}H COSY NMR of compound **16**.
$CDCl_3$

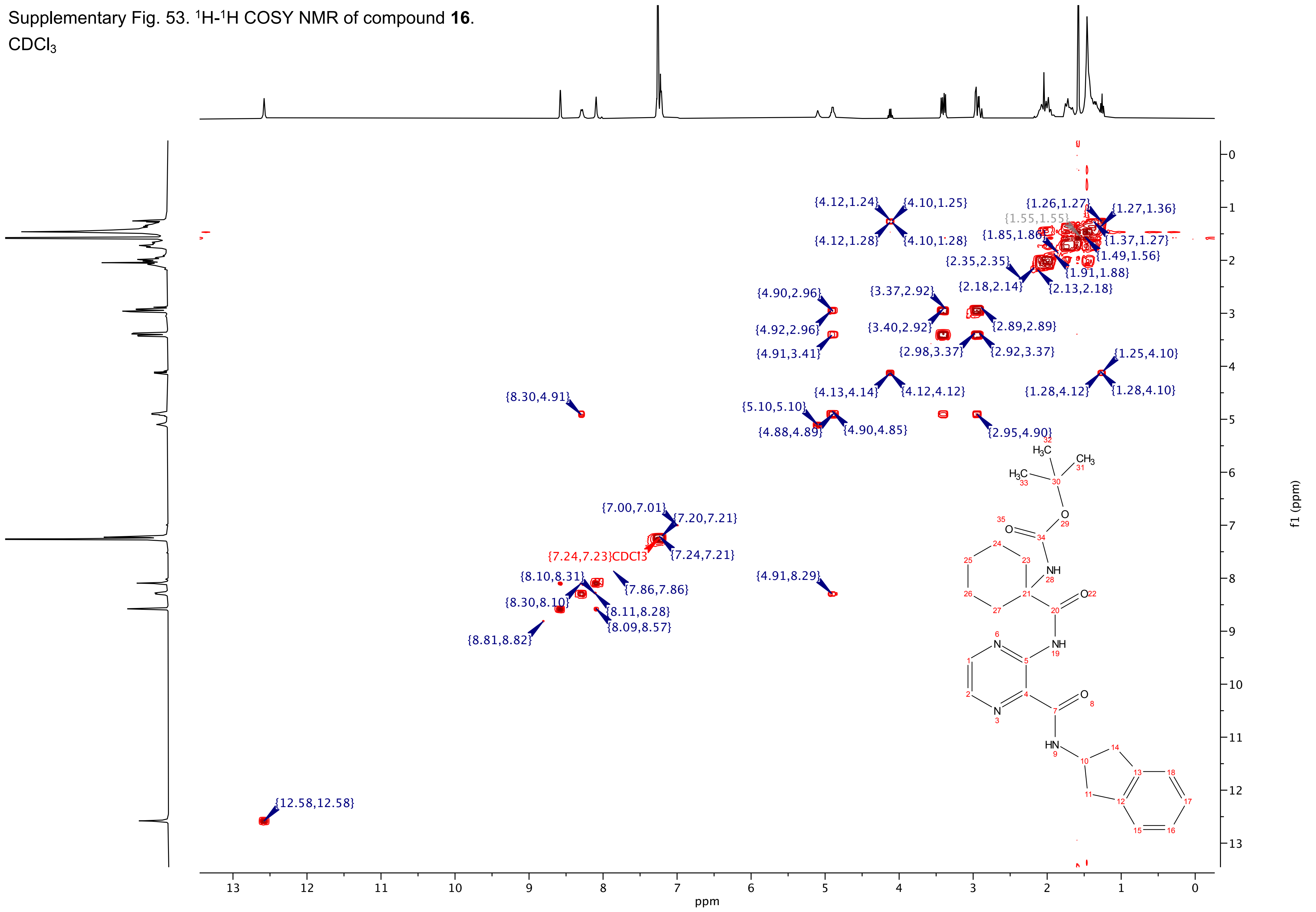

Supplementary Fig. 54. ^{1}H-^{13}C HMBC NMR of compound **16**.
$CDCl_3$

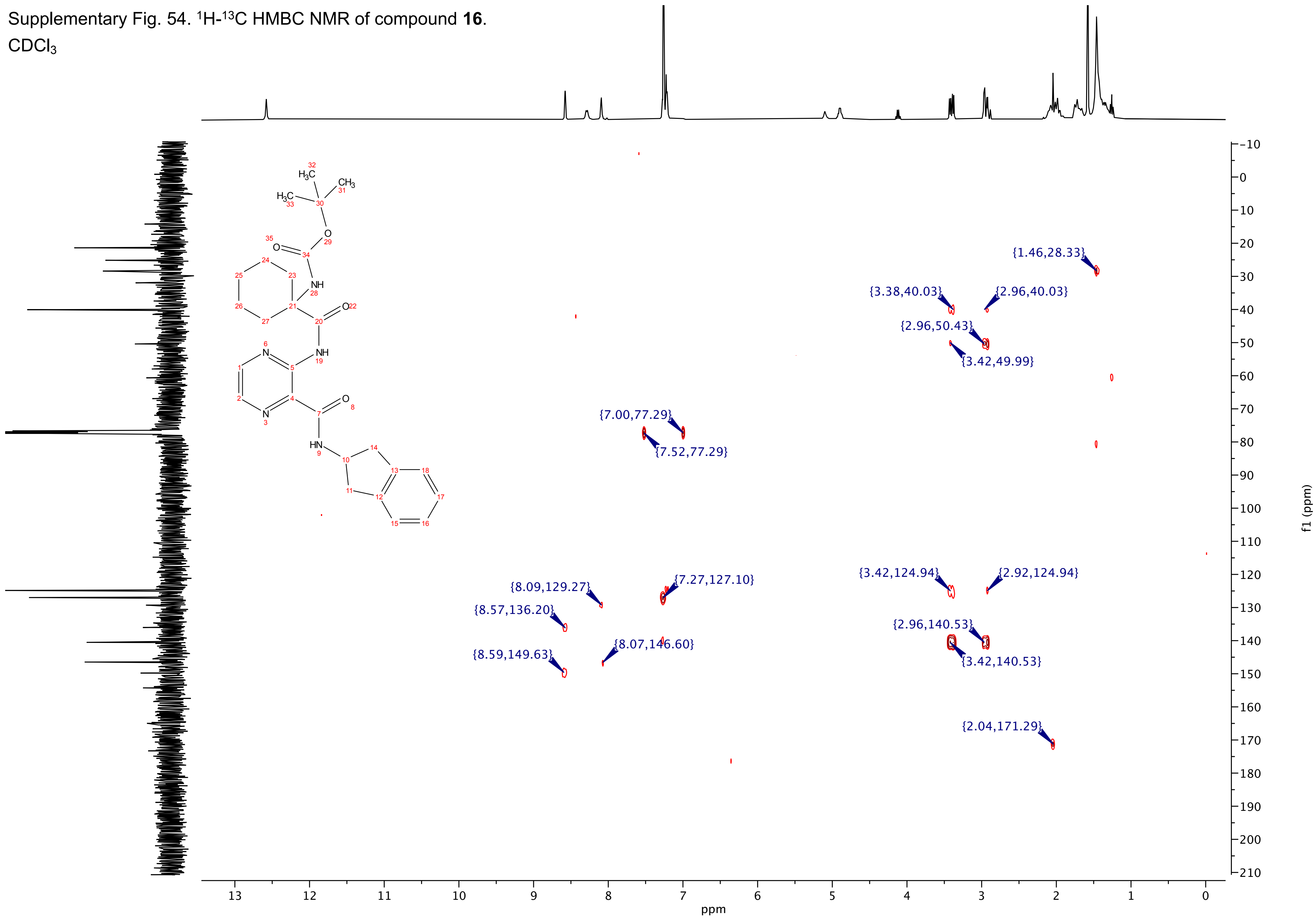

Supplementary Fig. 55. ^1H-^{13}C HSQC NMR of compound **16**.
$CDCl_3$

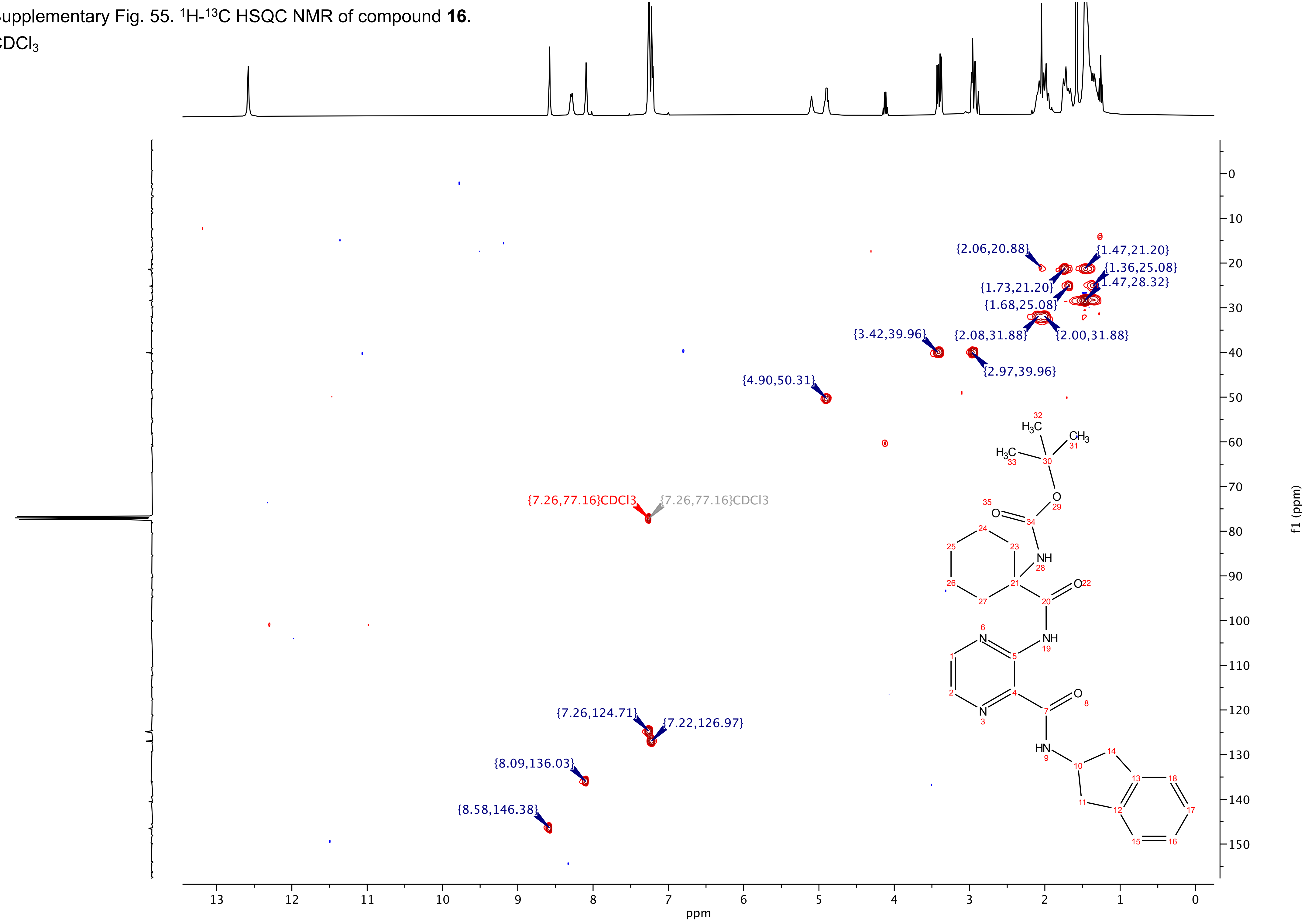

Supplementary Fig. 56. ^1H-^1H NOESY NMR of compound **16**.

$CDCl_3$

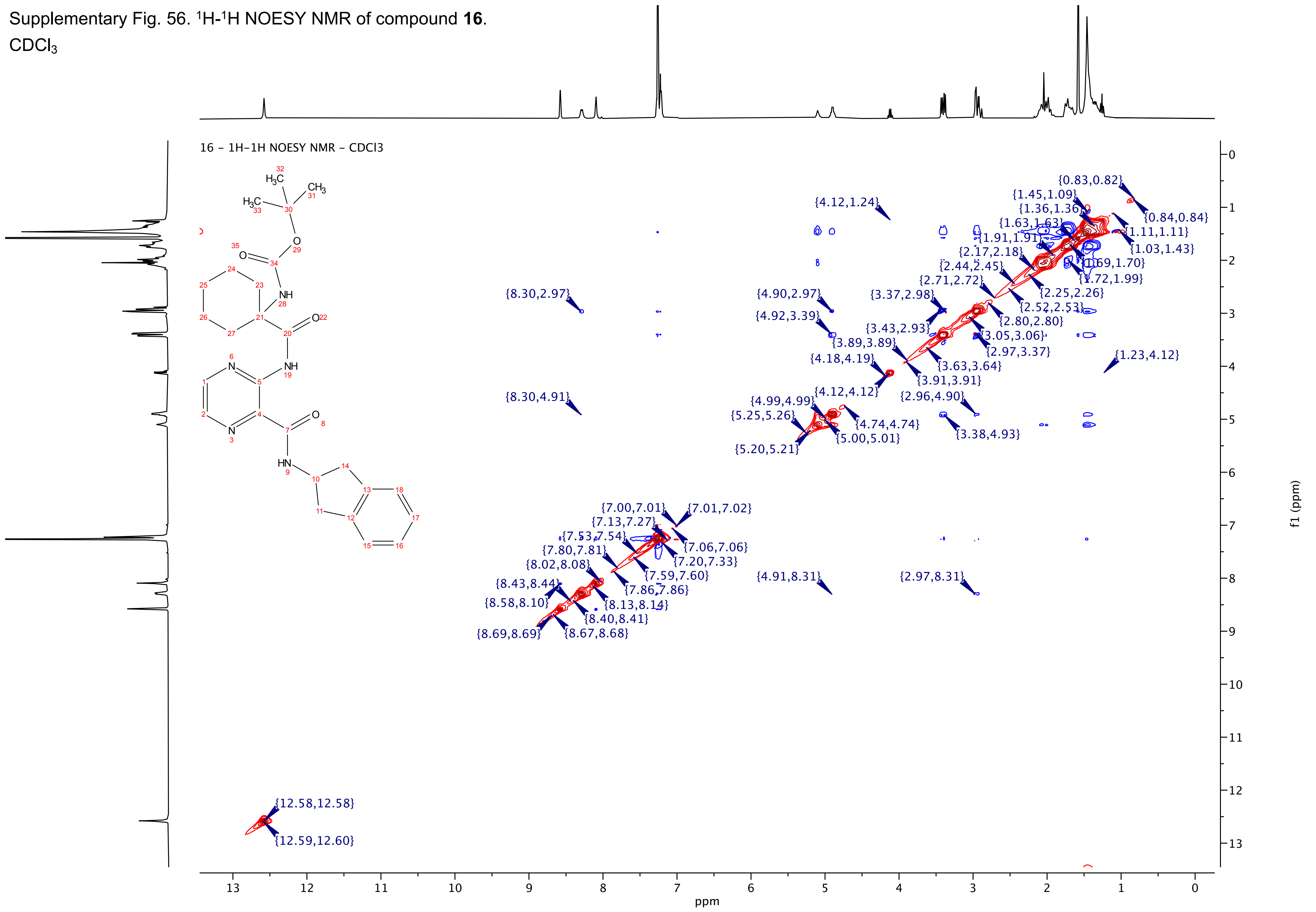

Supplementary Fig. 57. ^{1}H NMR of compound **17**.
d_7-DMF

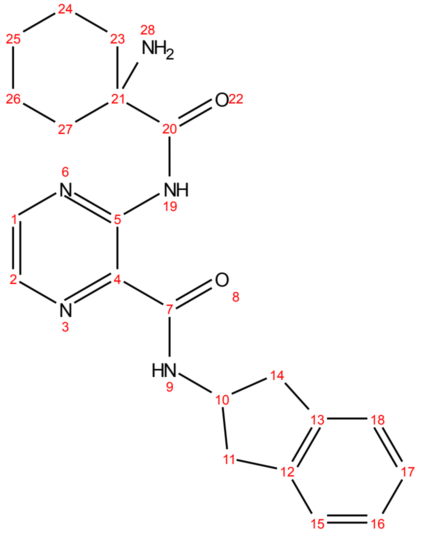

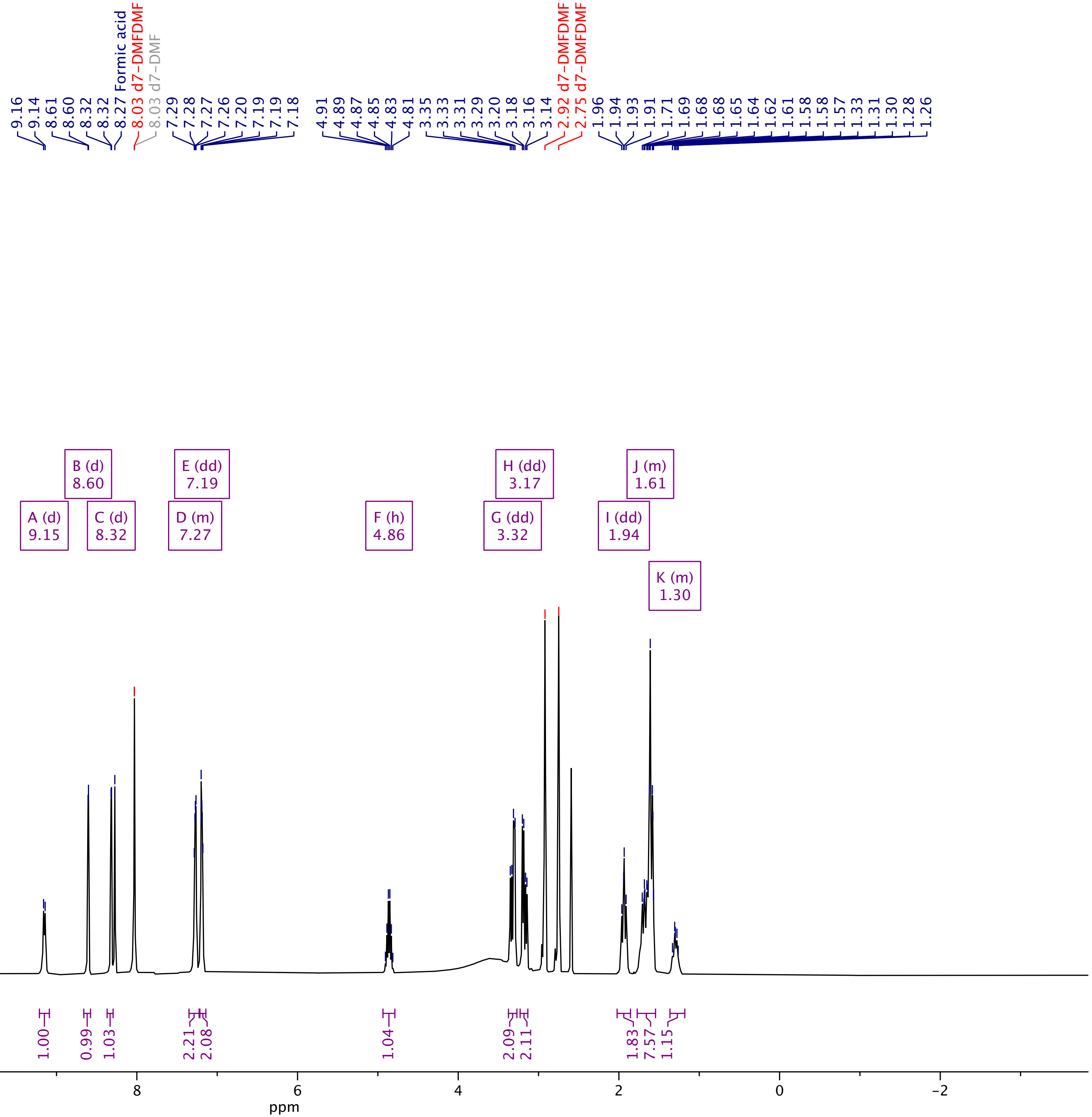

Supplementary Fig. 58. ^{13}C NMR of compound **17**.

d_7-DMF

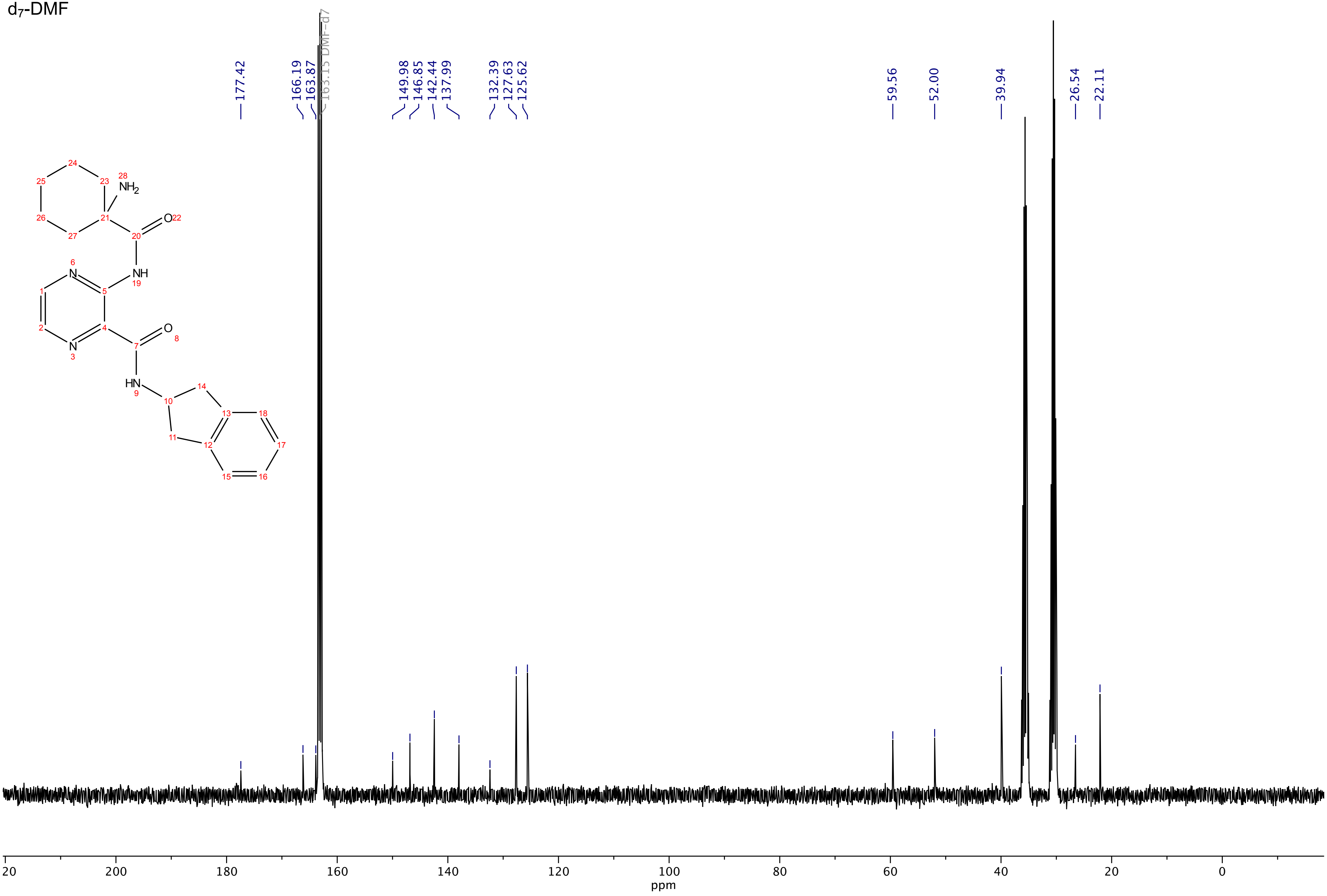

Supplementary Fig. 59. ^{1}H NMR of compound **18**.

$CDCl_3$

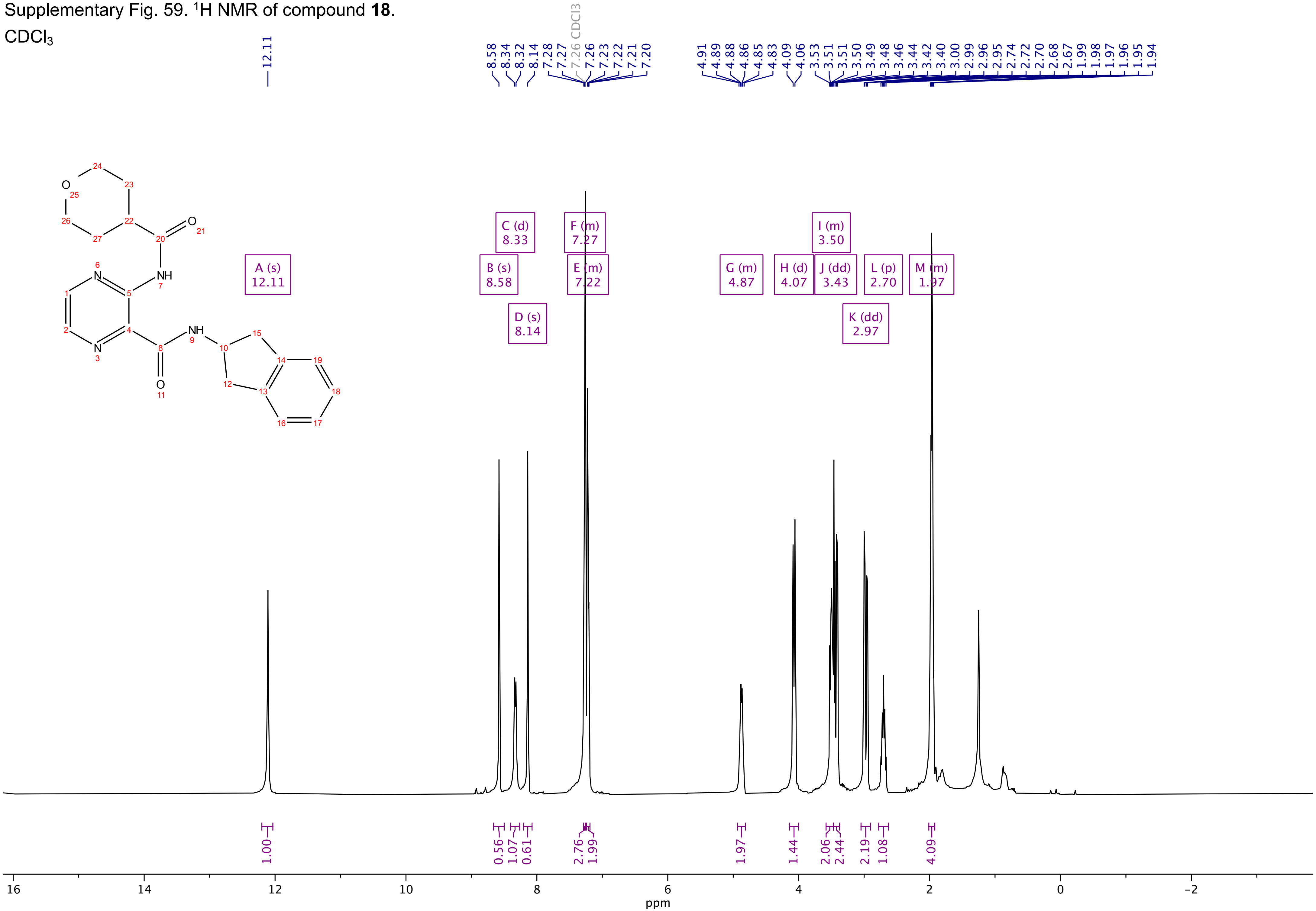

Supplementary Fig. 60. ^{13}C NMR of compound **18**.

CDCl$_3$

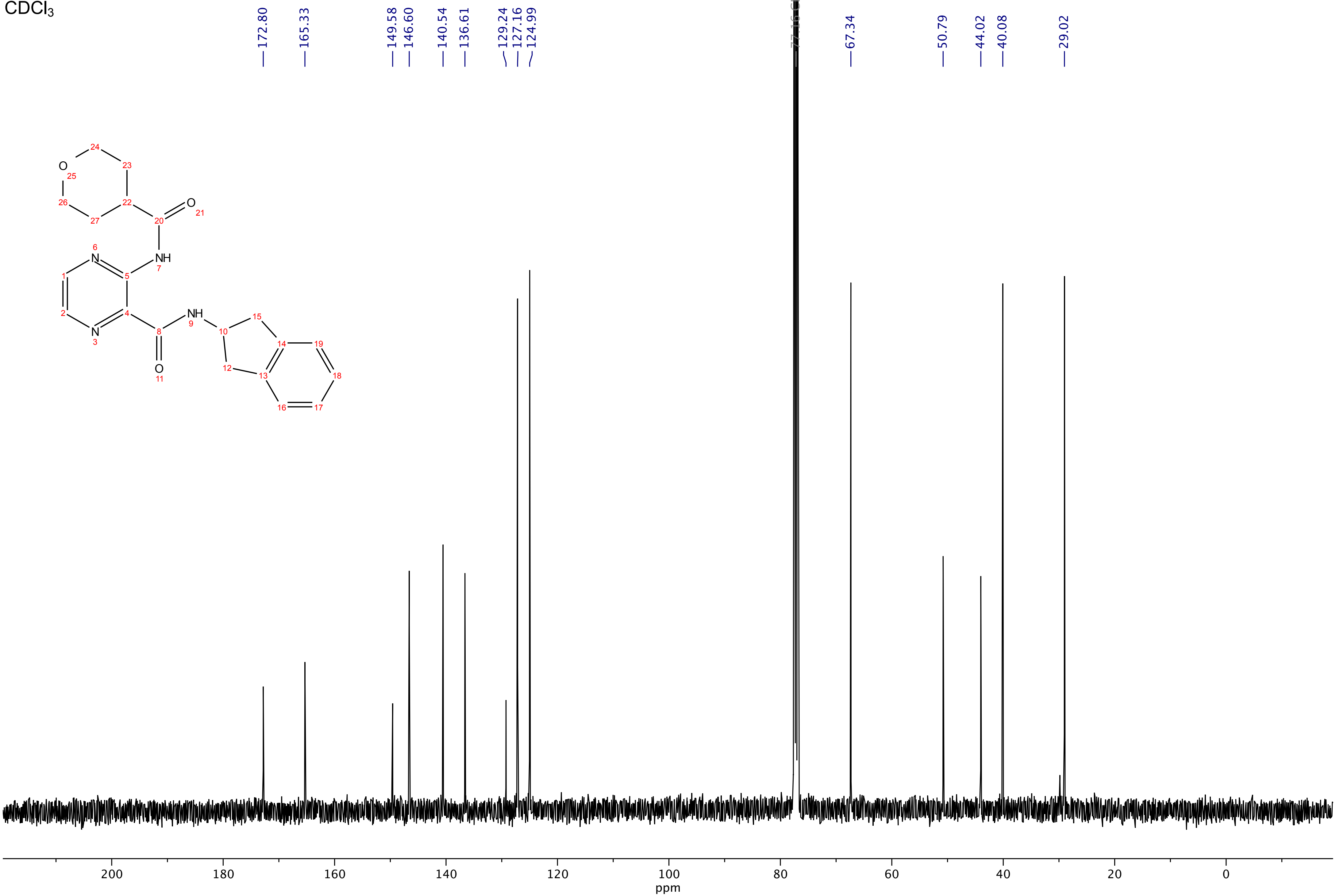

Supplementary Fig. 61. ^{1}H NMR of compound **19**.

$CDCl_3$

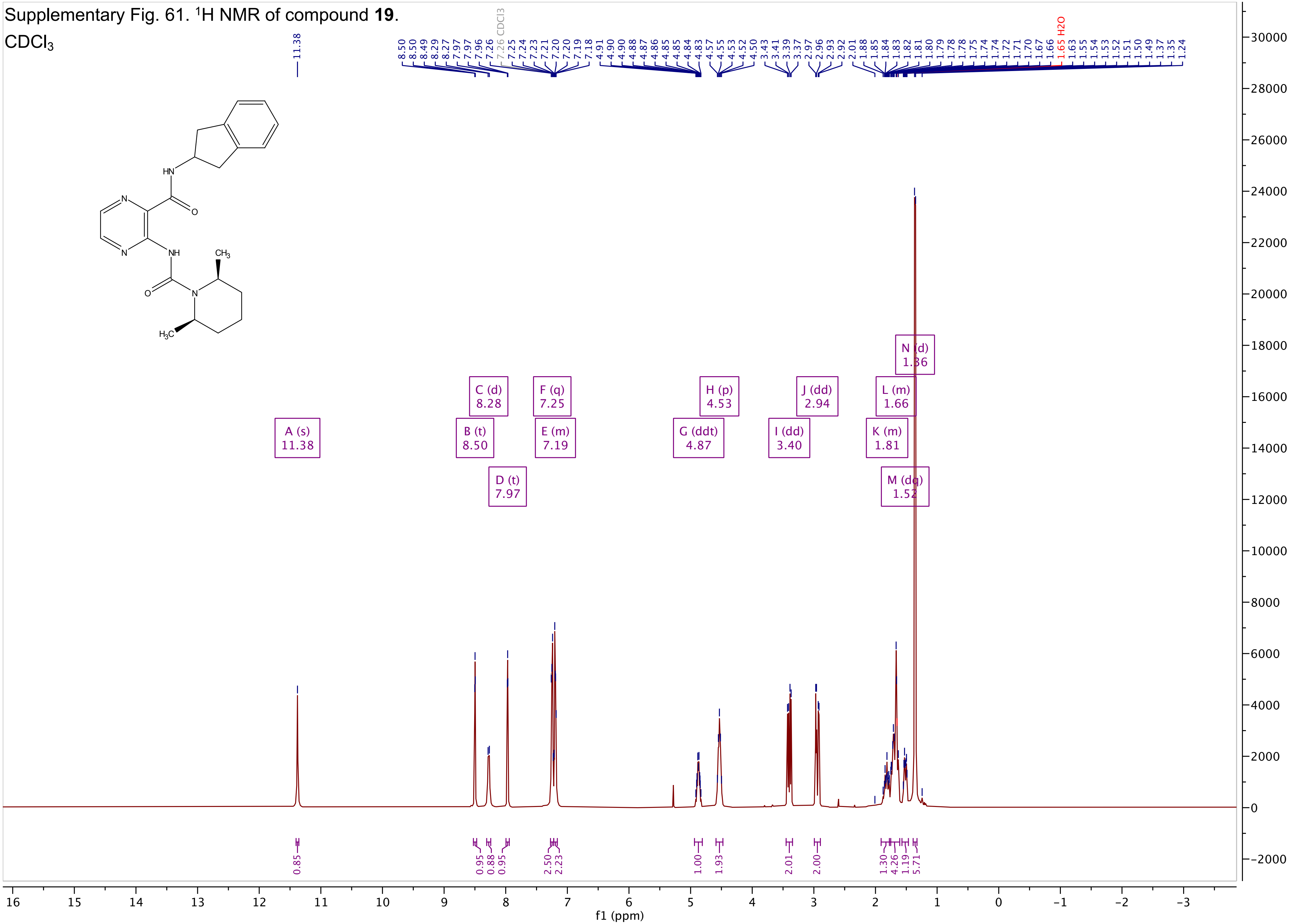

Supplementary Fig. 62. ^{13}C NMR of compound **19**.

$CDCl_3$

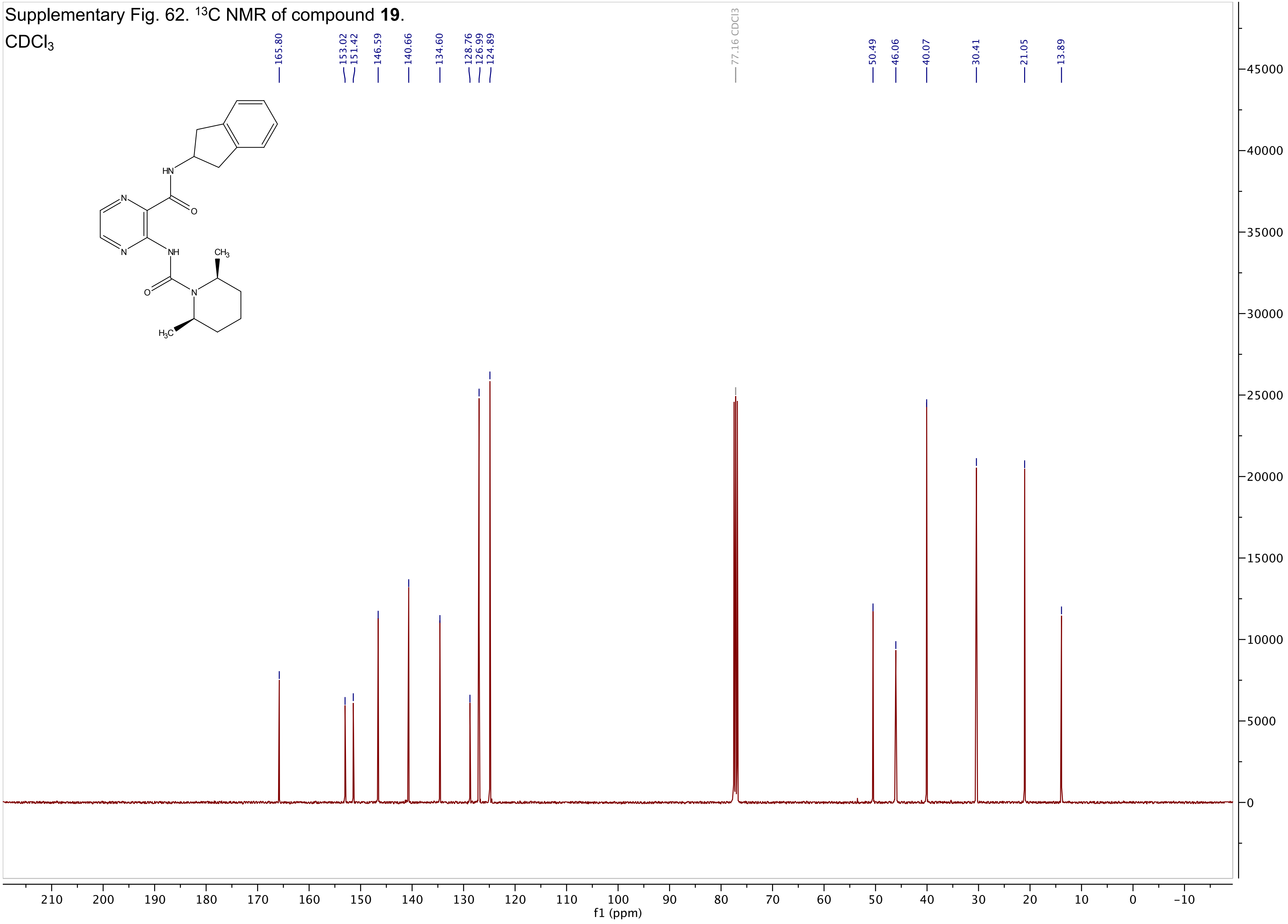

Supplementary Fig. 63. ^{1}H NMR of compound **19**.

d_6DMSO

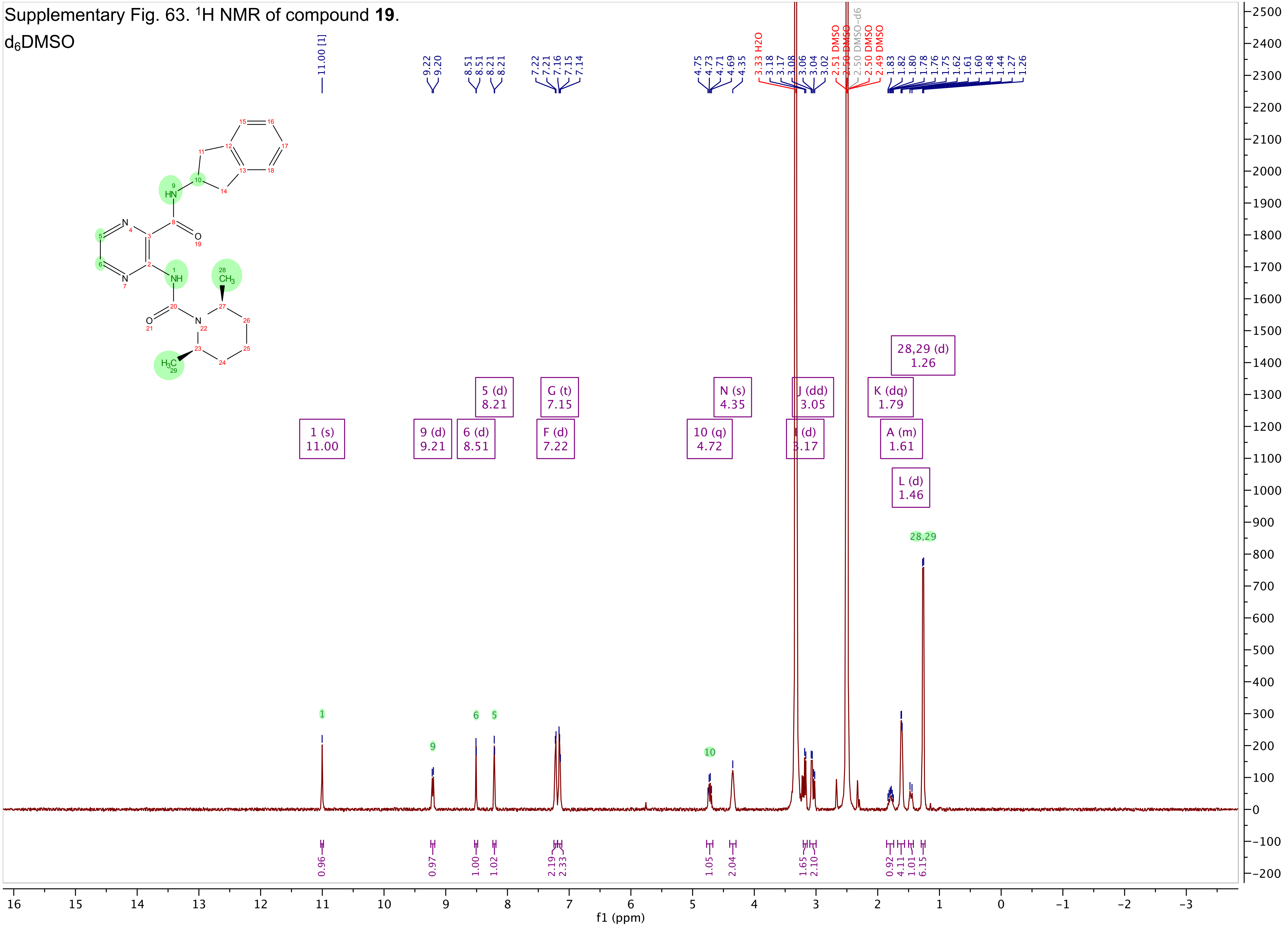

Supplementary Fig. 64. ^{1}H NMR of compound **20**.

$CDCl_3$

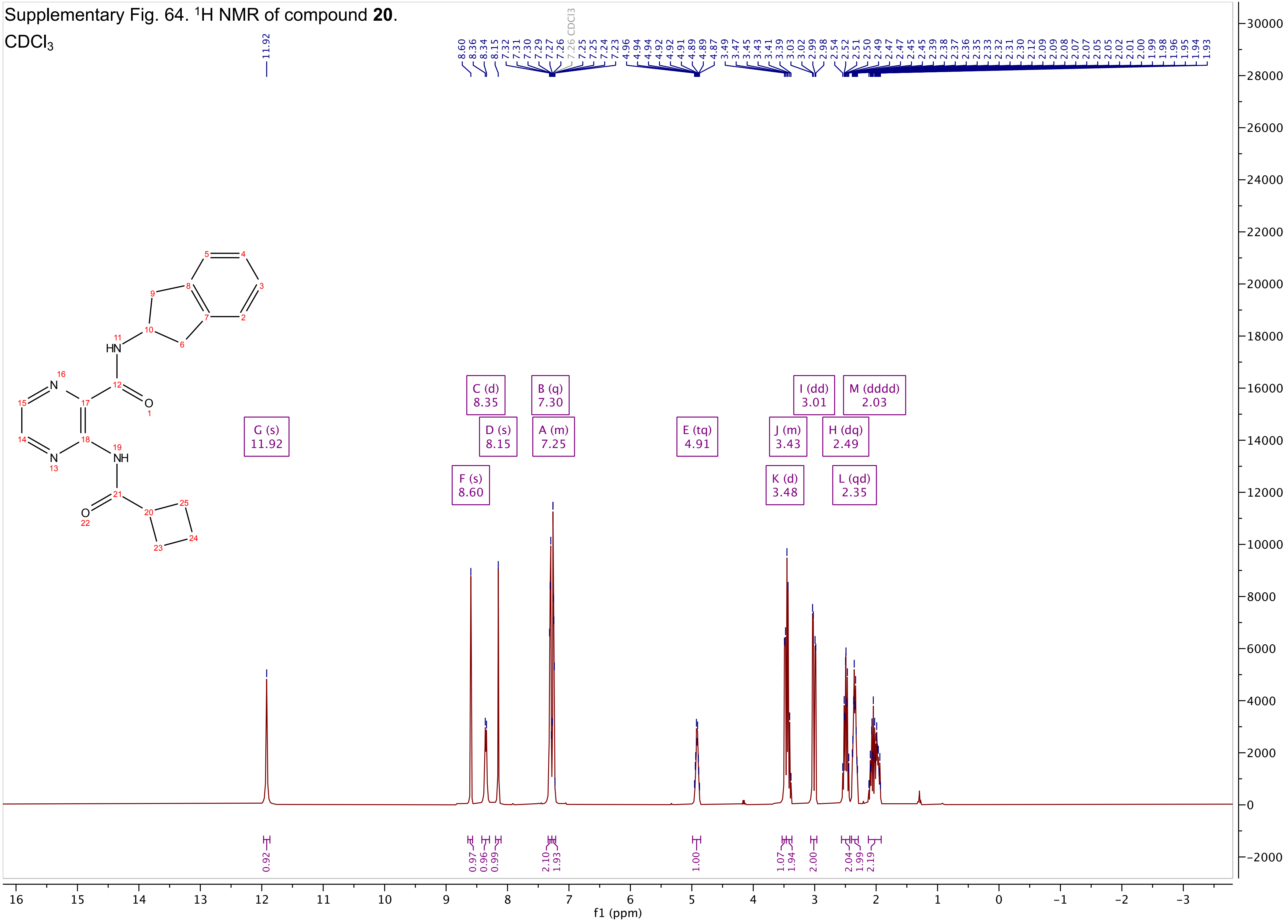

Supplementary Fig. 65. ^{13}C NMR of compound **20**.

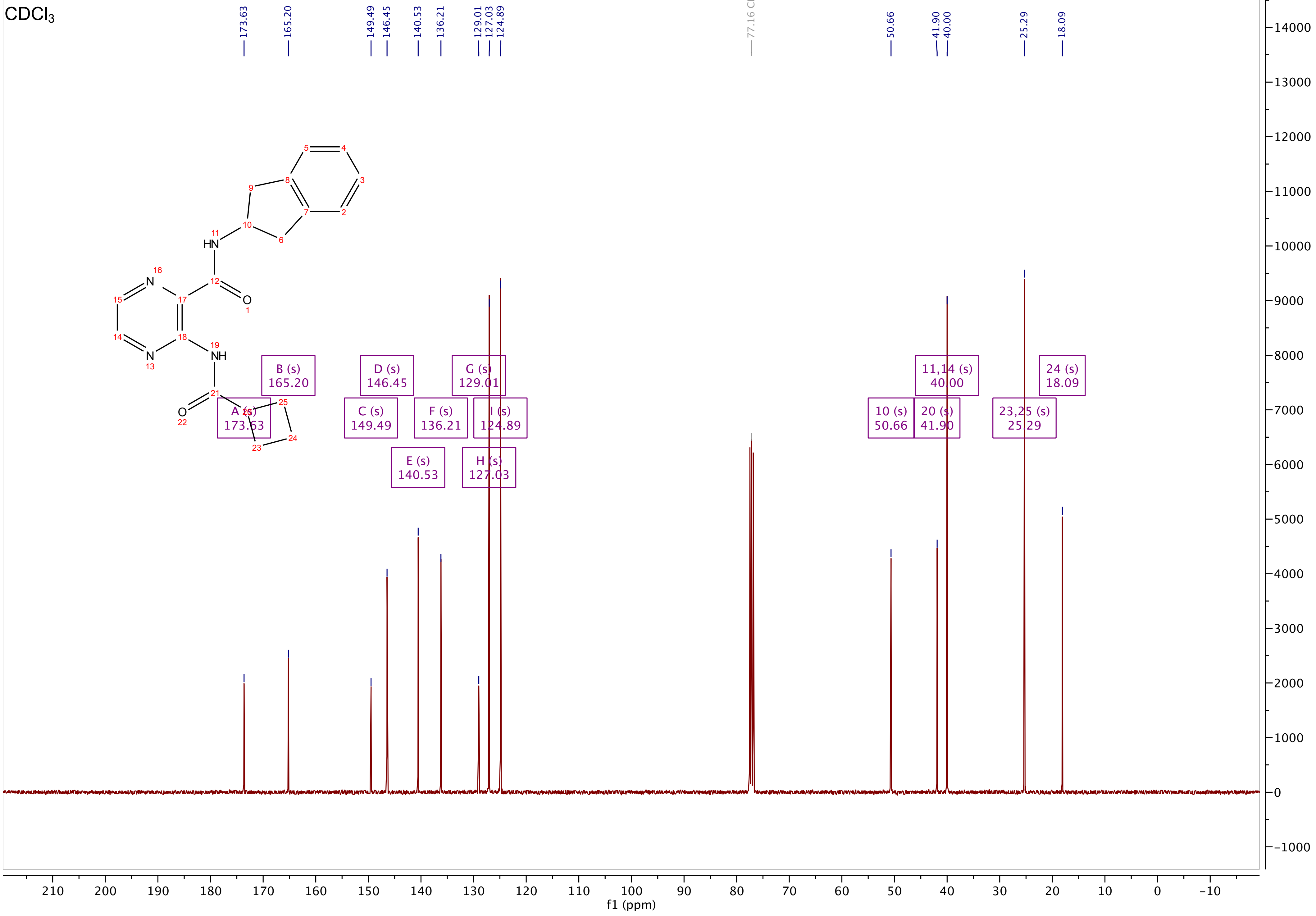

Supplementary Fig. 66. ^{1}H NMR of compound **21**.

$CDCl_3$

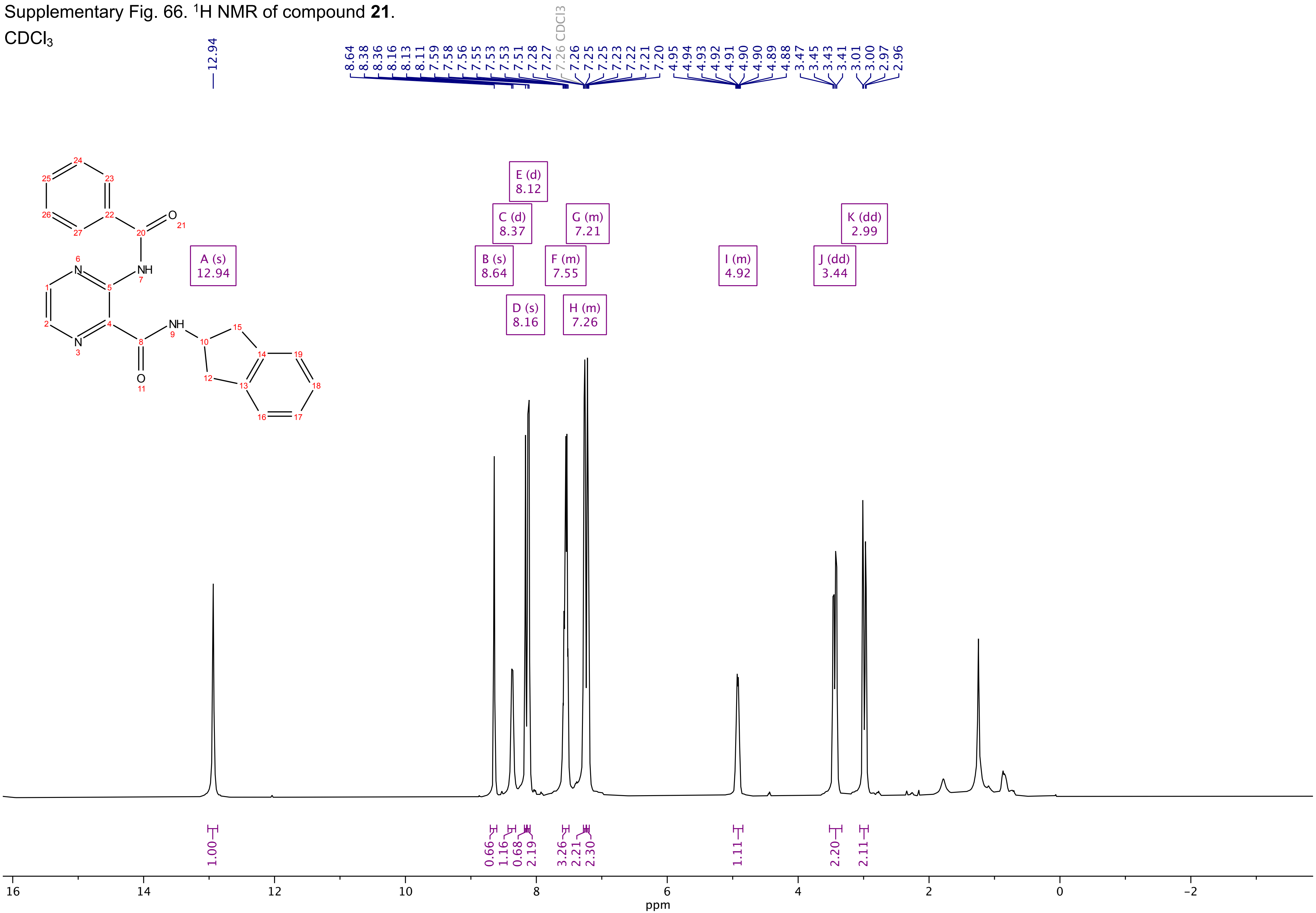

Supplementary Fig. 67. ^{13}C NMR of compound **21**.

$CDCl_3$

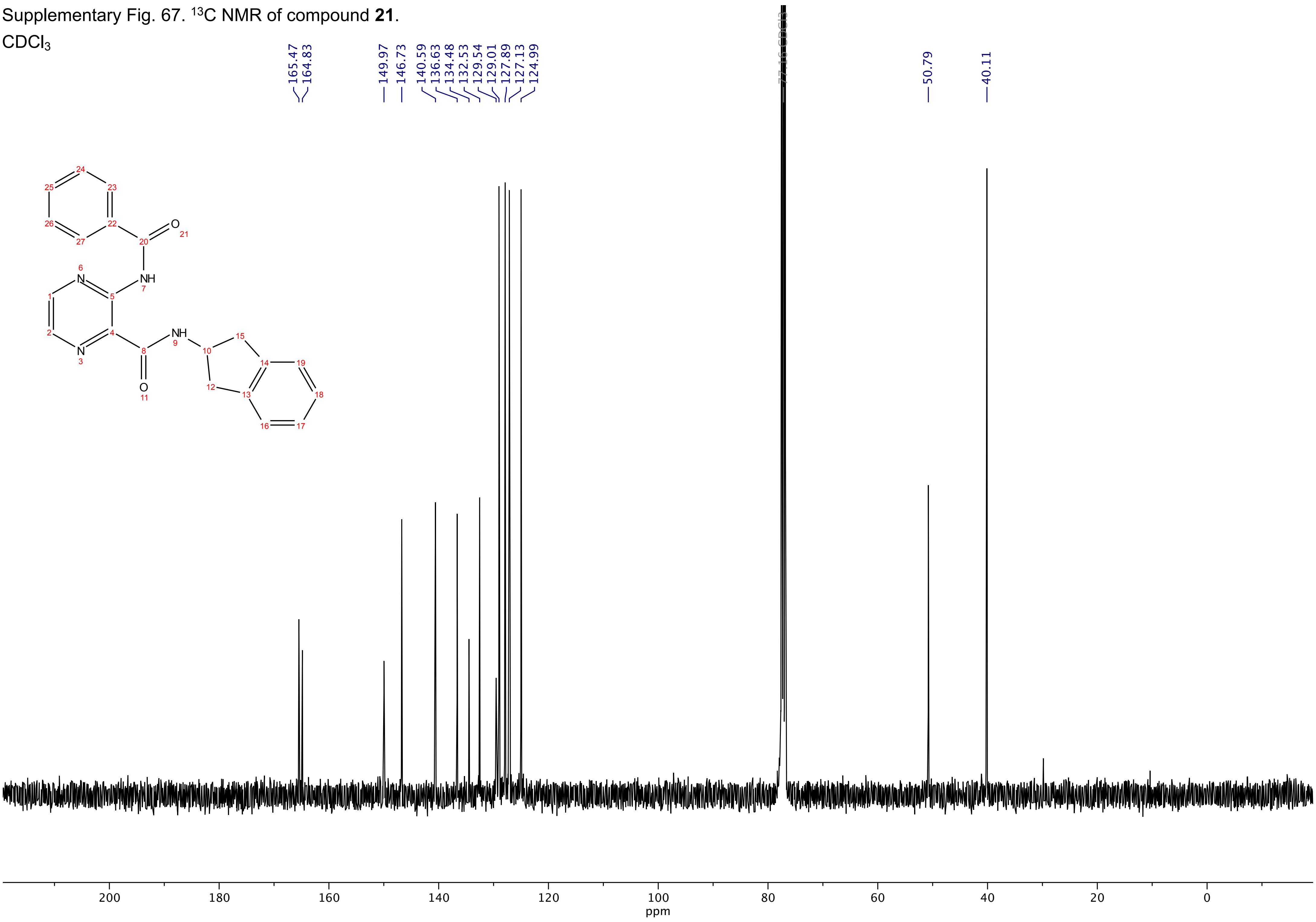

Supplementary Fig. 68. ^{1}H NMR of compound **22**.

CD_3OD

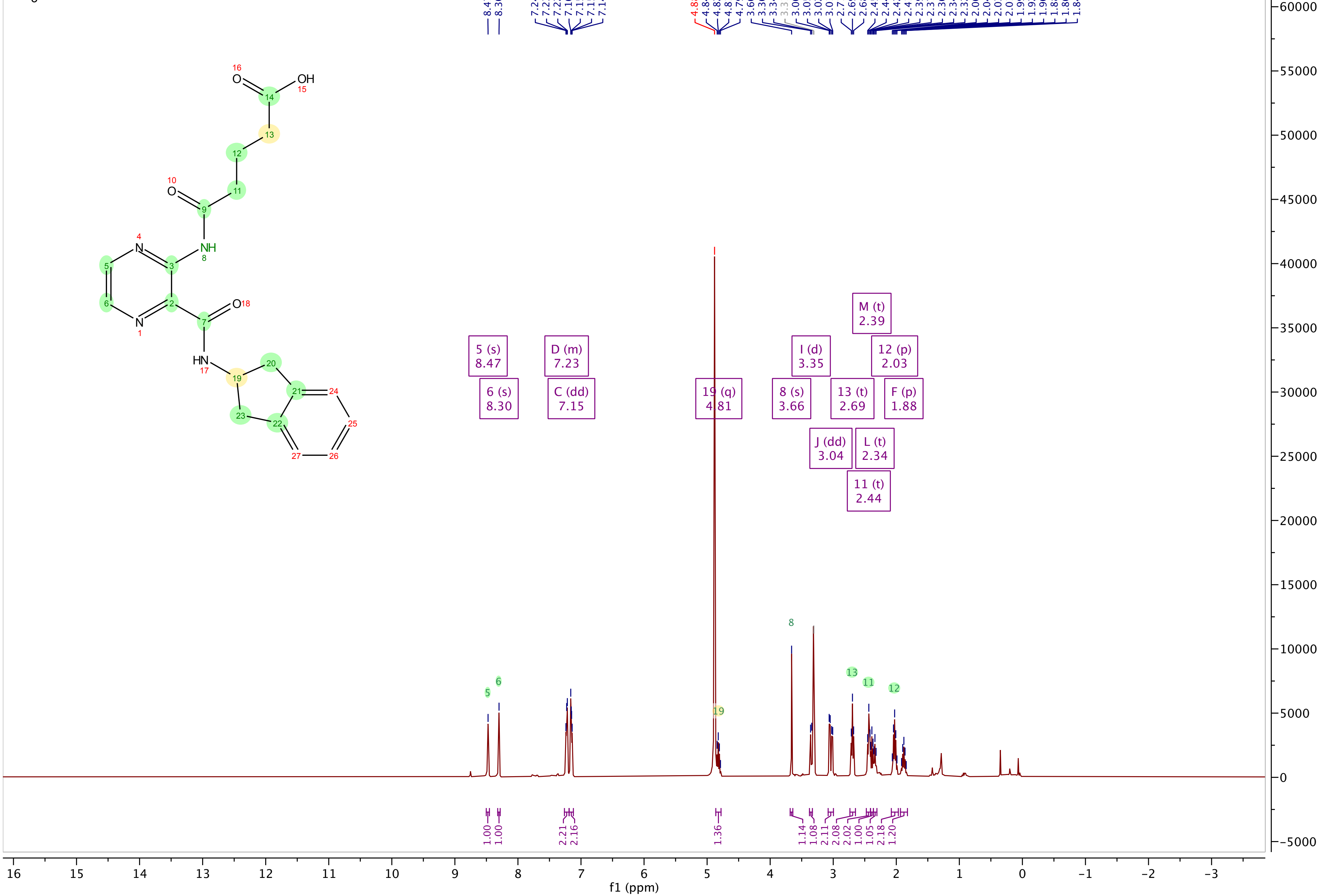

Supplementary Fig. 69. ^{13}C NMR of compound **22**.

CD_3OD

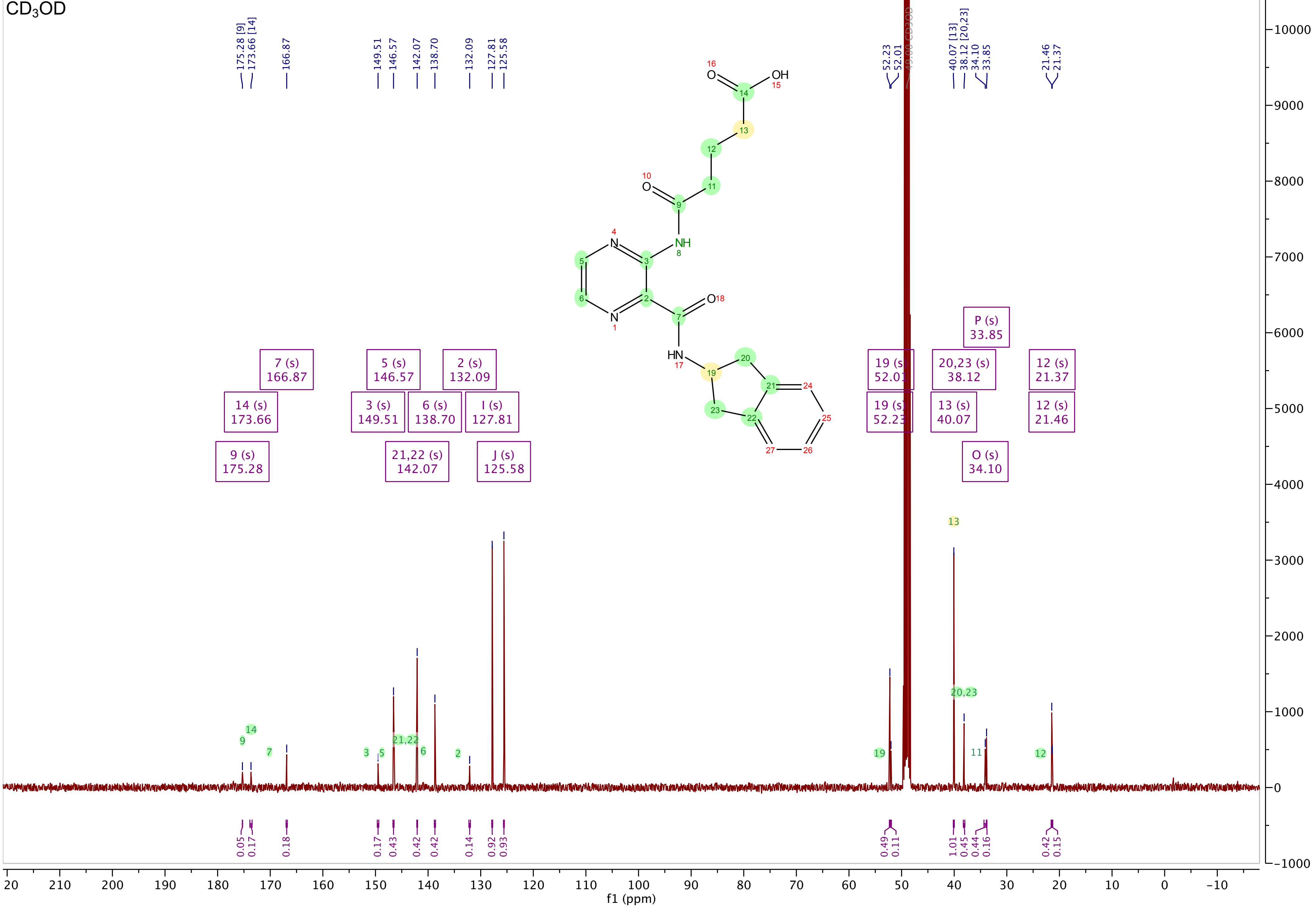

Supplementary Fig. 70. ^{1}H NMR of compound **23**.
d_6DMSO

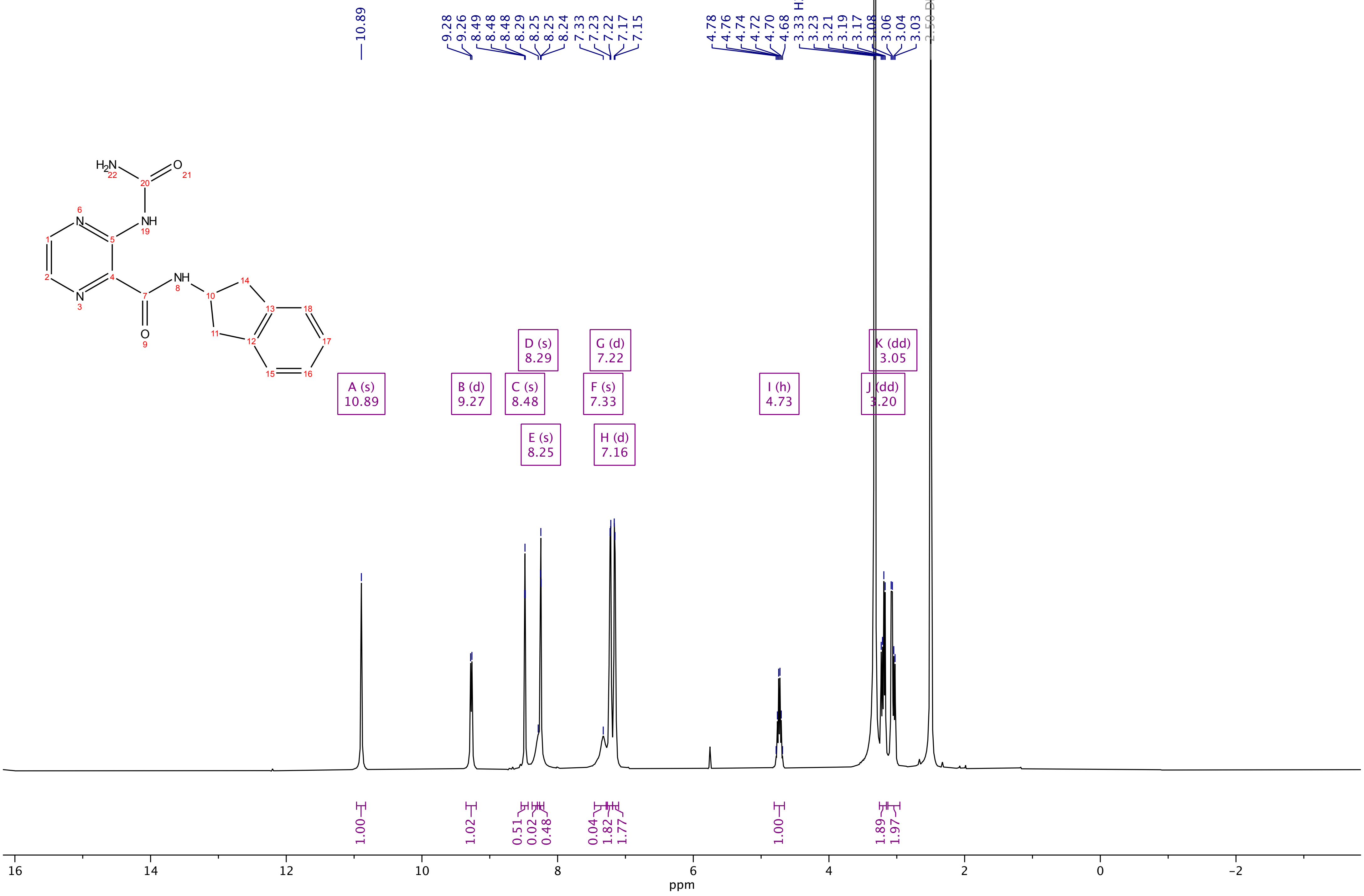

Supplementary Fig. 71. ^{13}C NMR of compound **23**.

d_6DMSO

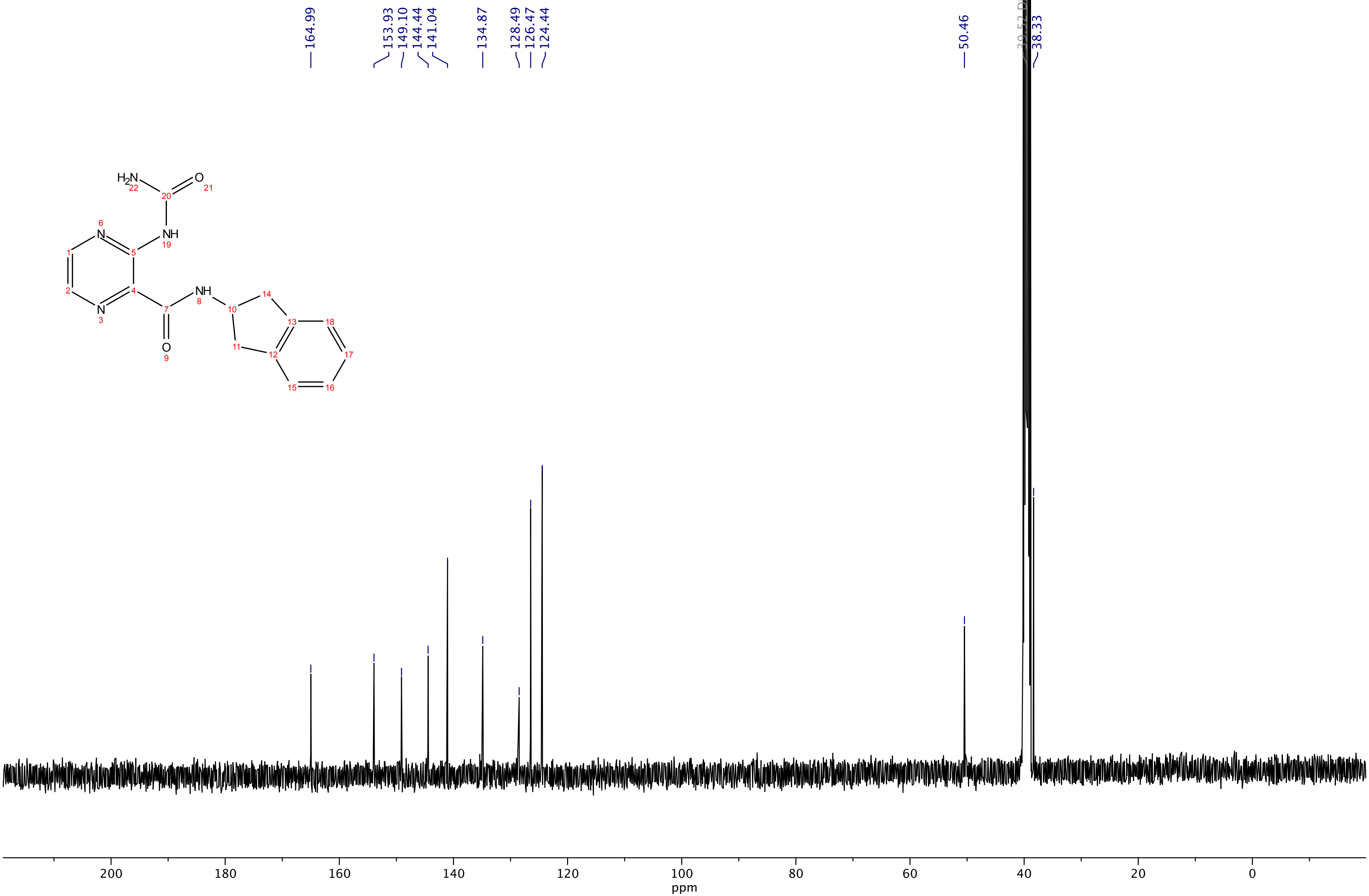

Supplementary Fig. 72. ^{1}H NMR of compound **41**.

$CDCl_3$

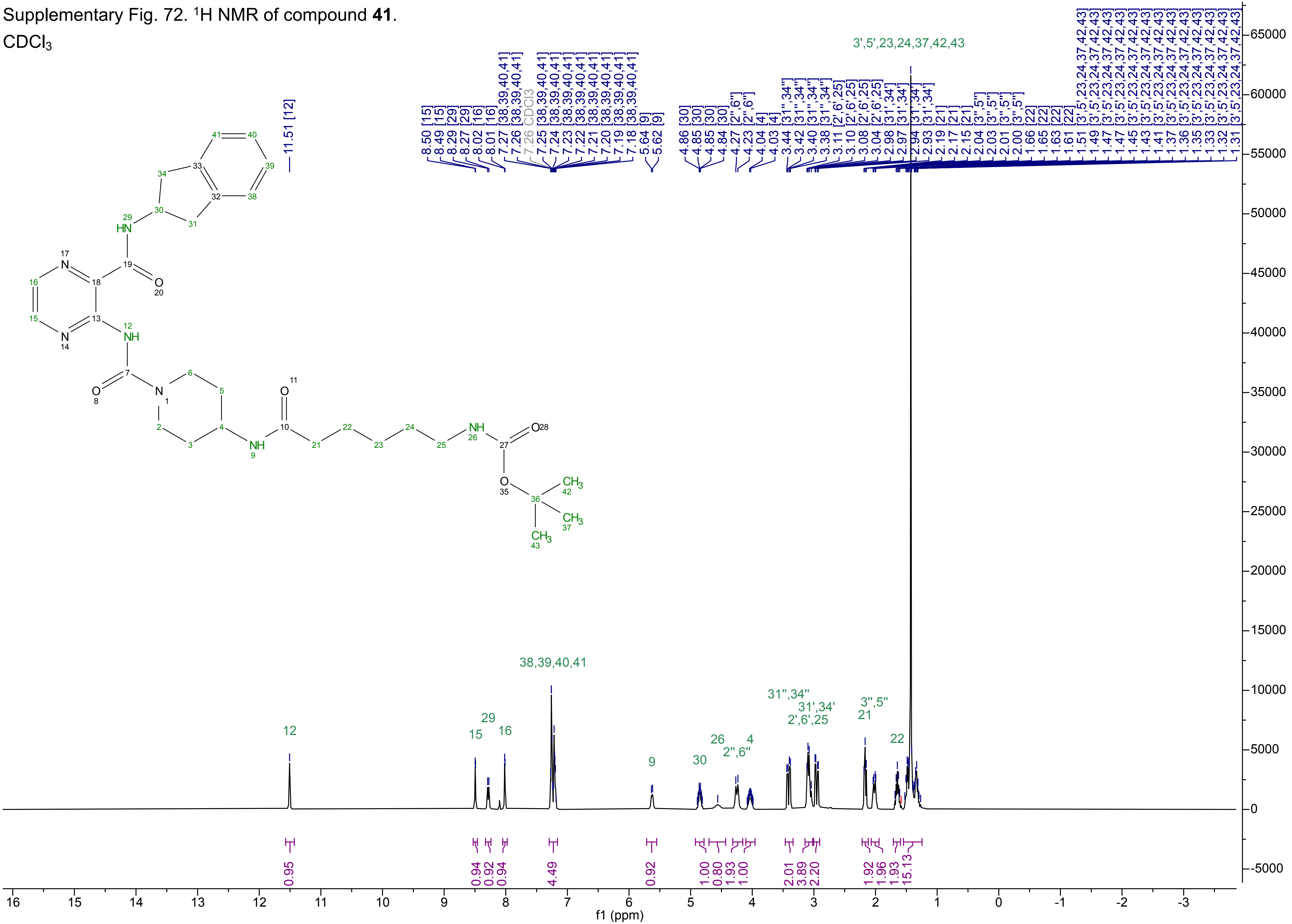

Supplementary Fig. 73. ^{13}C NMR of compound **41**.

$CDCl_3$

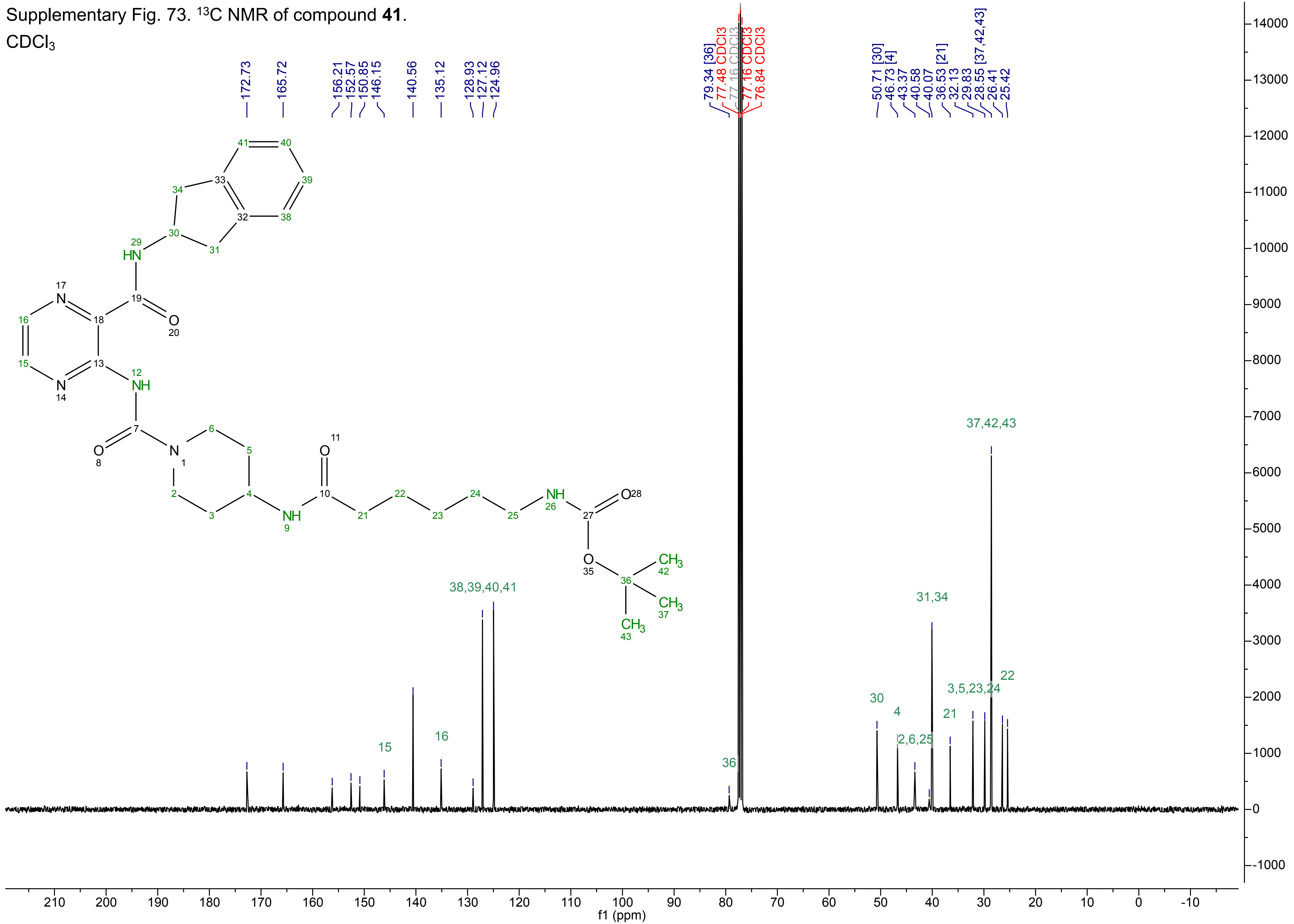

Supplementary Fig. 74. ^{1}H NMR of compound **42**.

CD_3OD

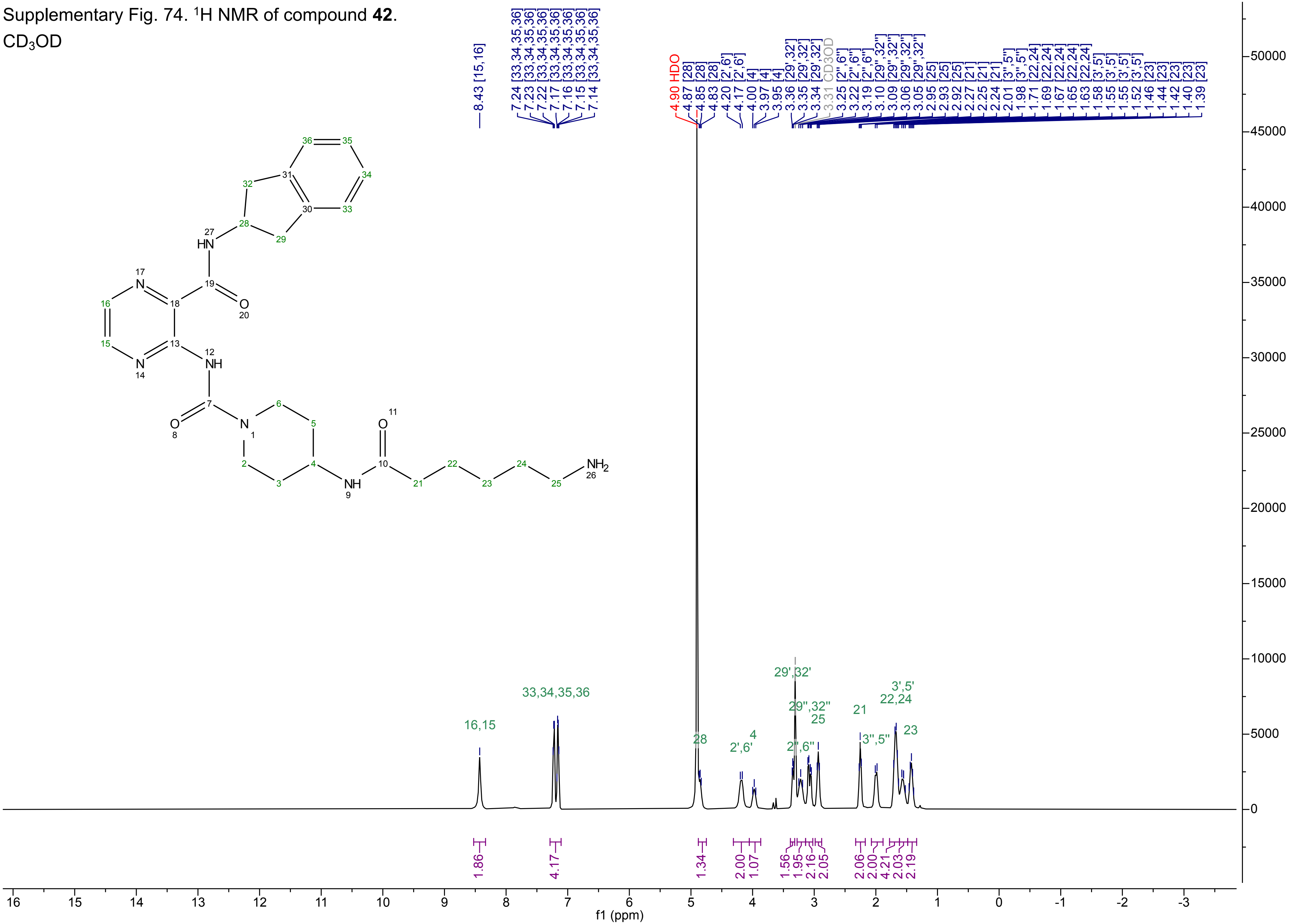

Supplementary Fig. 75. ^{13}C NMR of compound **42**.

CD_3OD

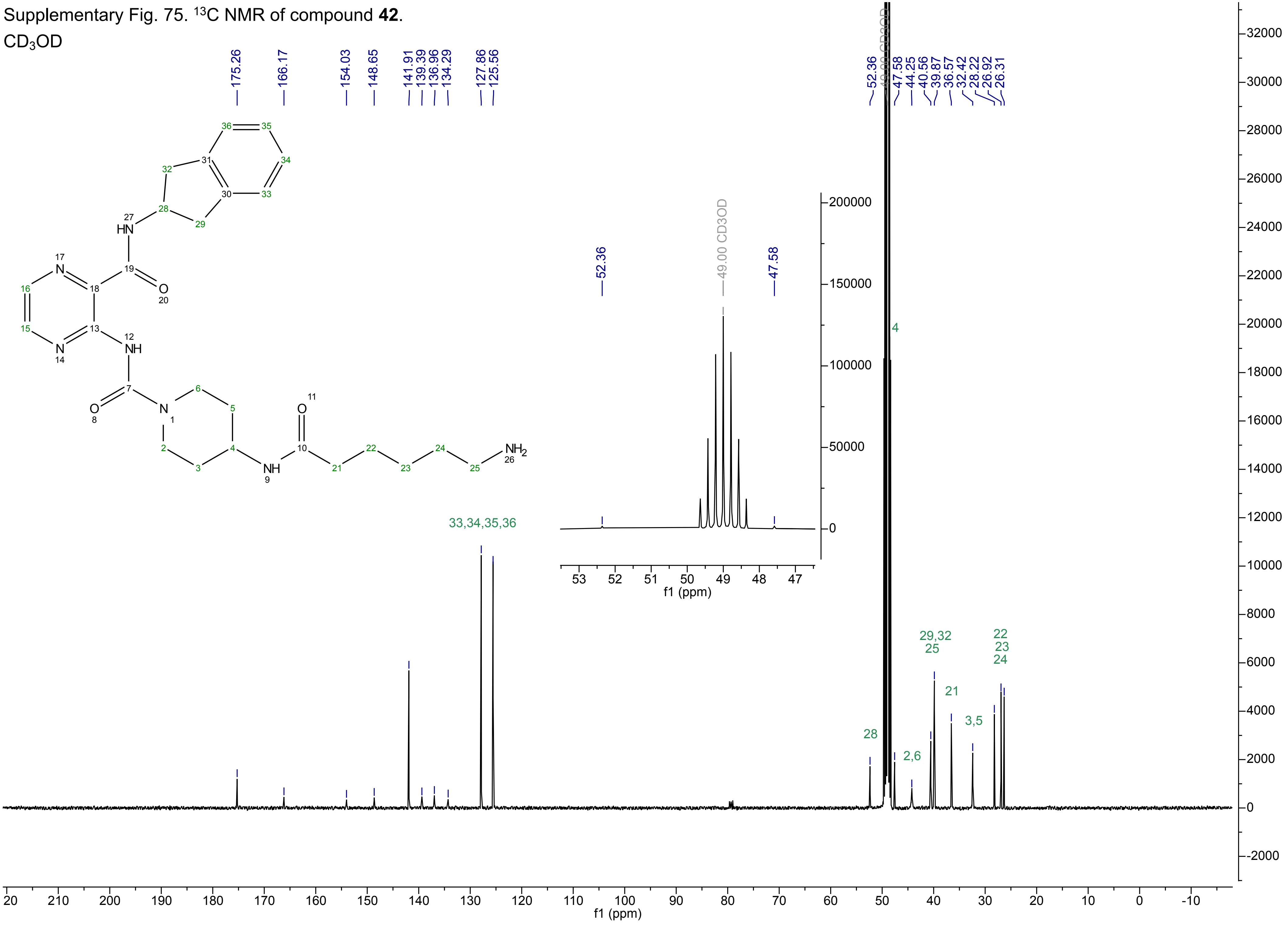

Supplementary Fig. 76. ^{1}H NMR of compound MAT379.

d_6DMSO

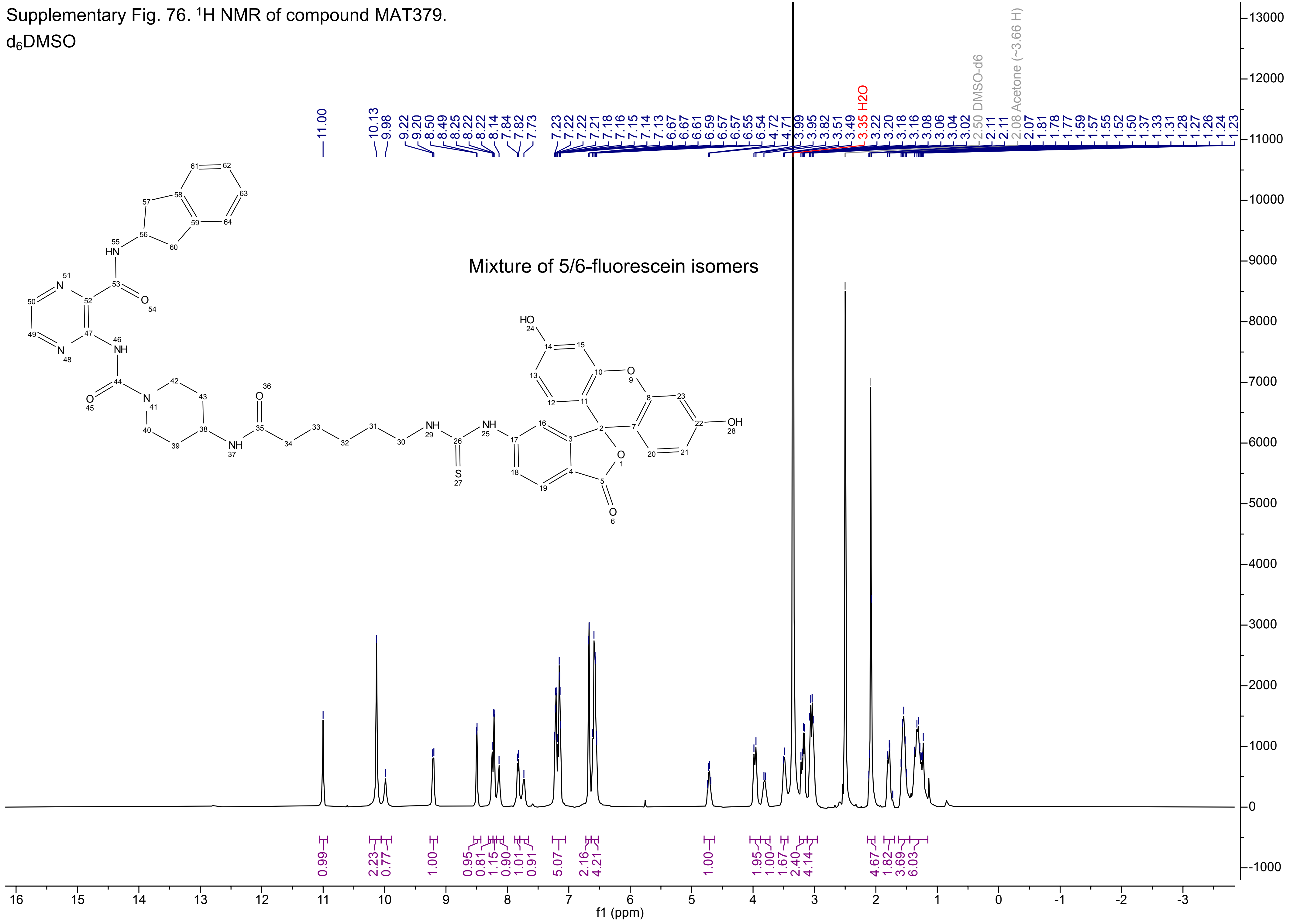

Supplementary Fig. 77. ^{13}C NMR of compound MAT379.

d_6DMSO

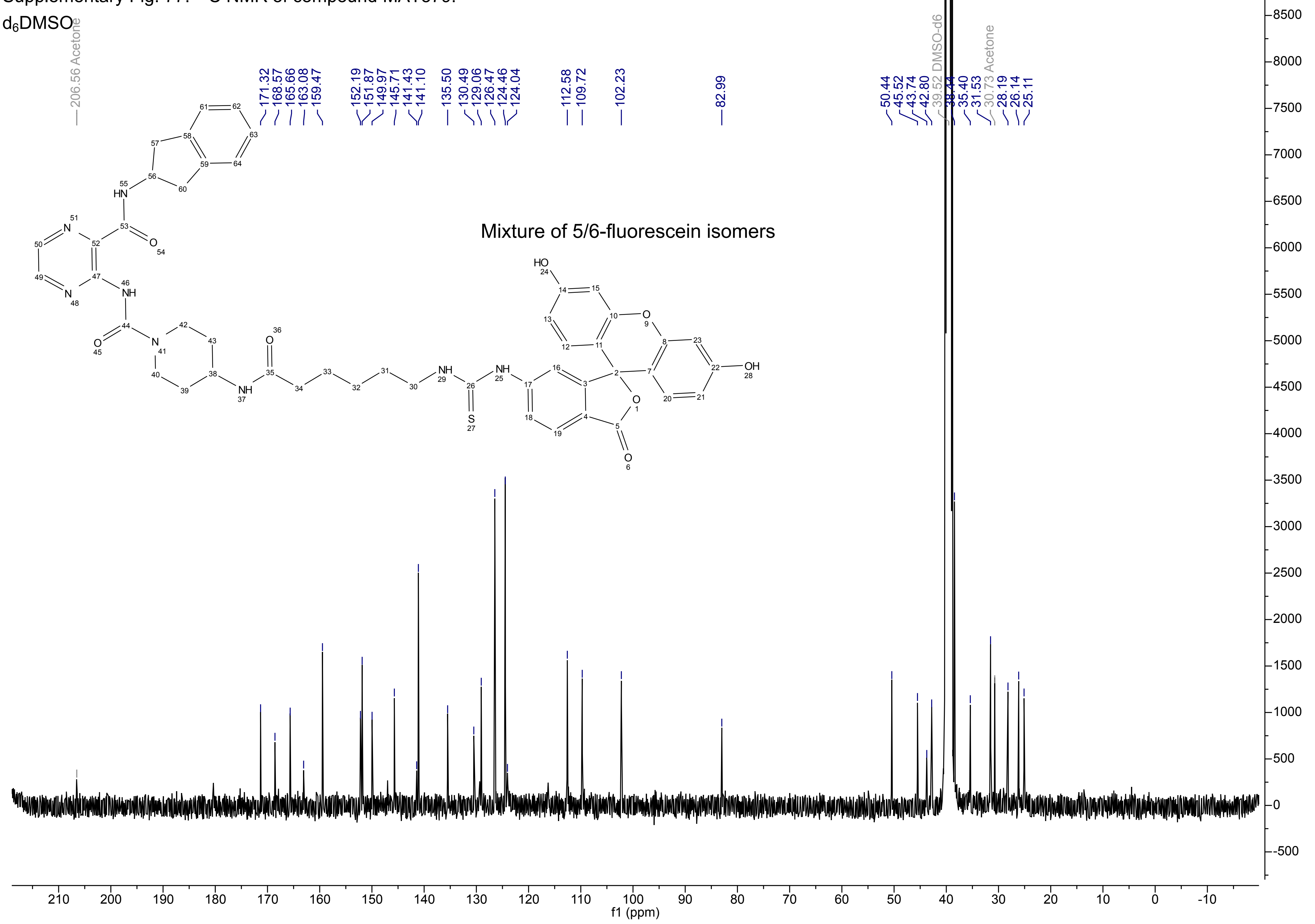

Supplementary Fig. 78. DEPT-135 NMR of compound MAT379.

d_6DMSO

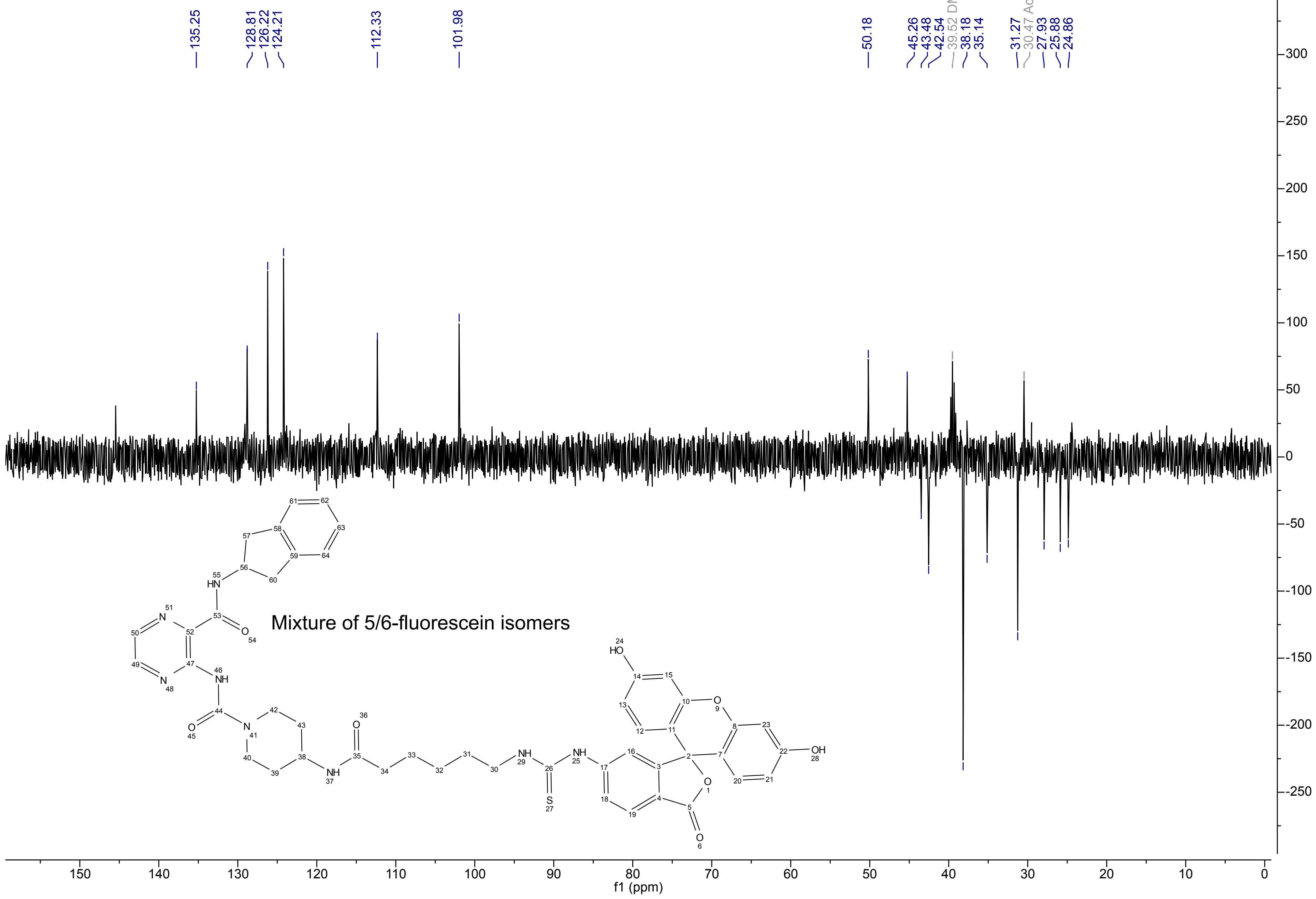

Supplementary Fig. 79. ^1H-^1H COSY NMR of compound MAT379.
d$_6$DMSO

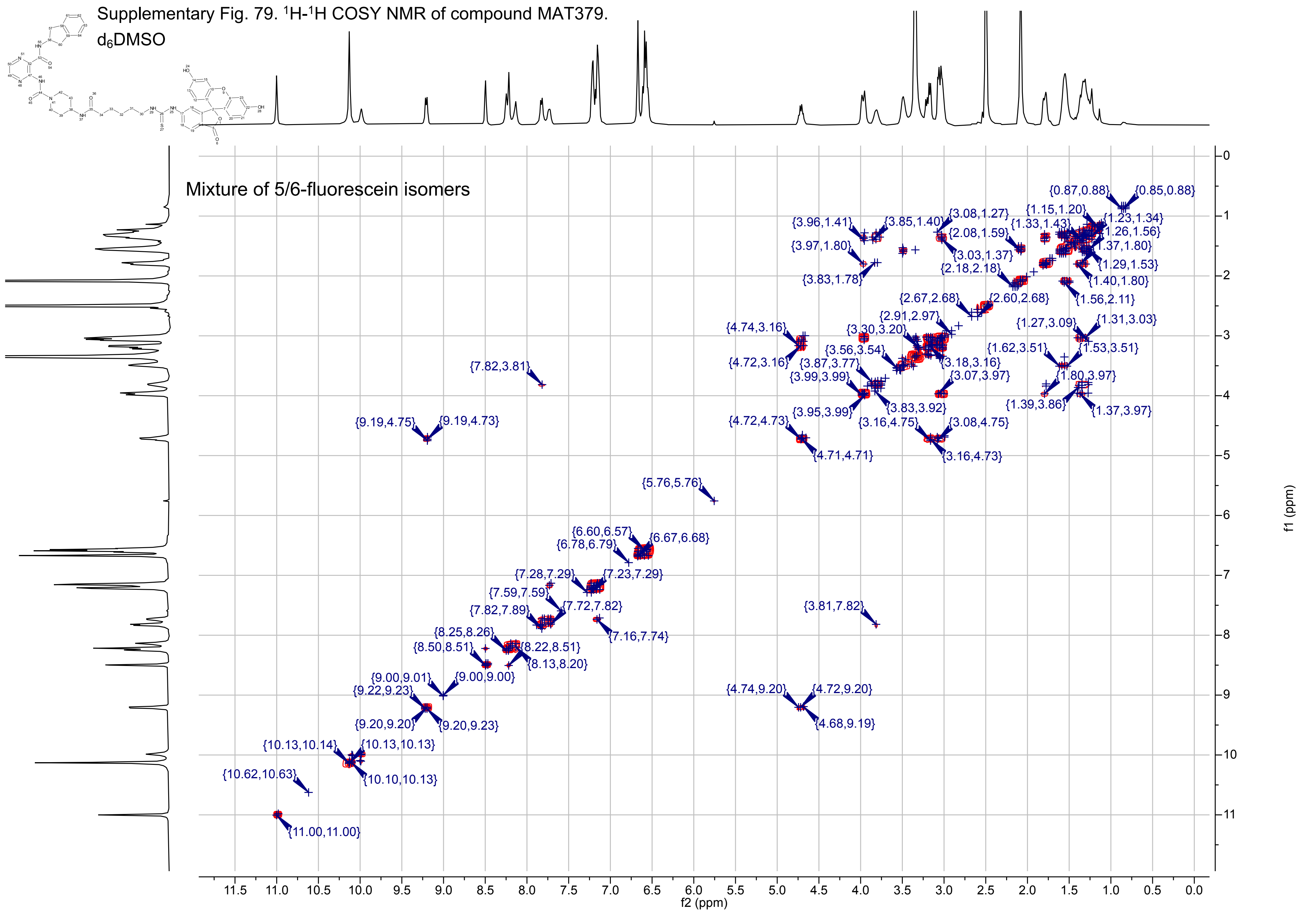

Supplementary Fig. 80. ^{1}H-^{13}C HMBC NMR of compound MAT379.
d_6DMSO

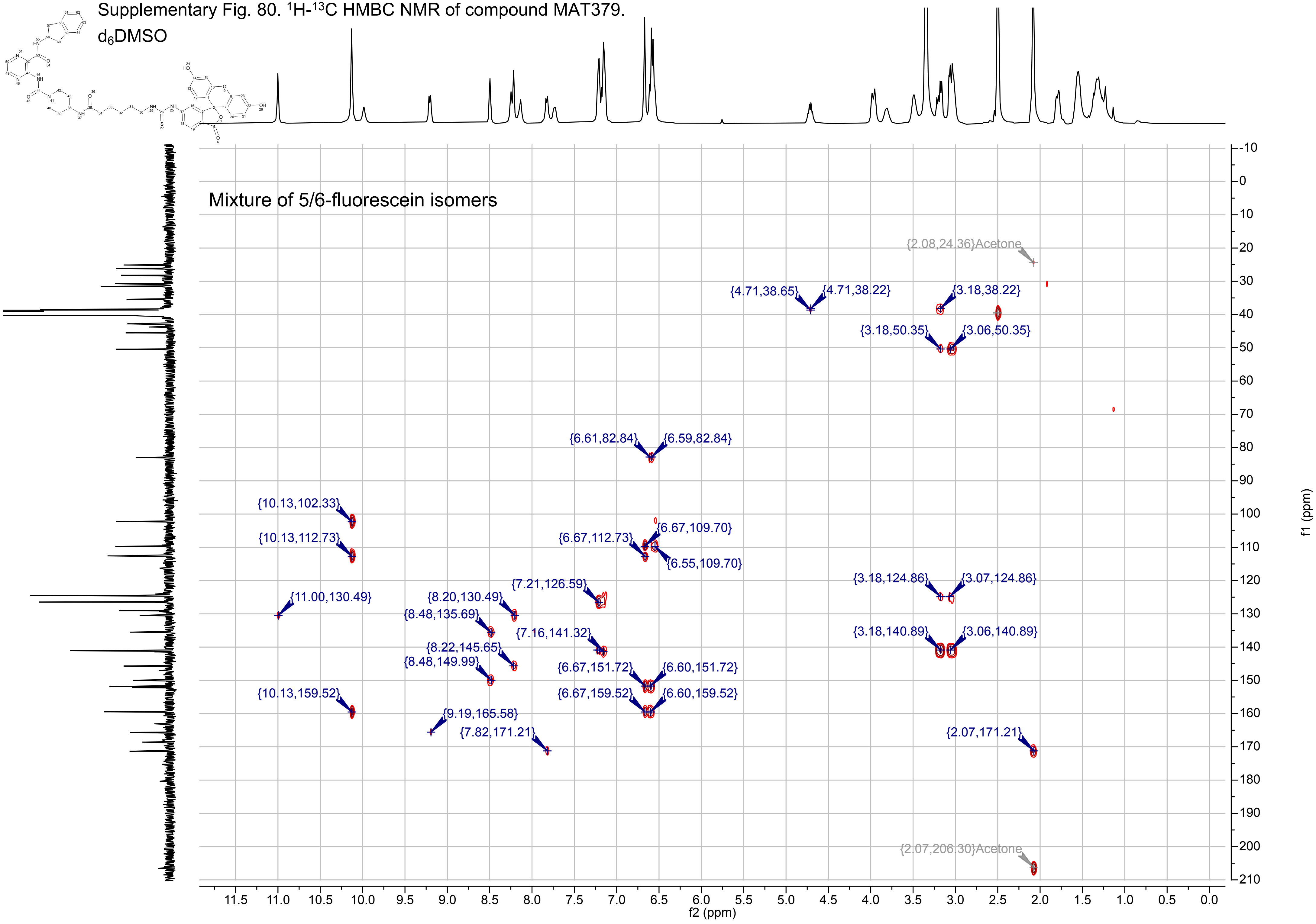

Supplementary Fig. 81. ^{1}H-^{13}C HSQC NMR of compound MAT379.

d_6DMSO

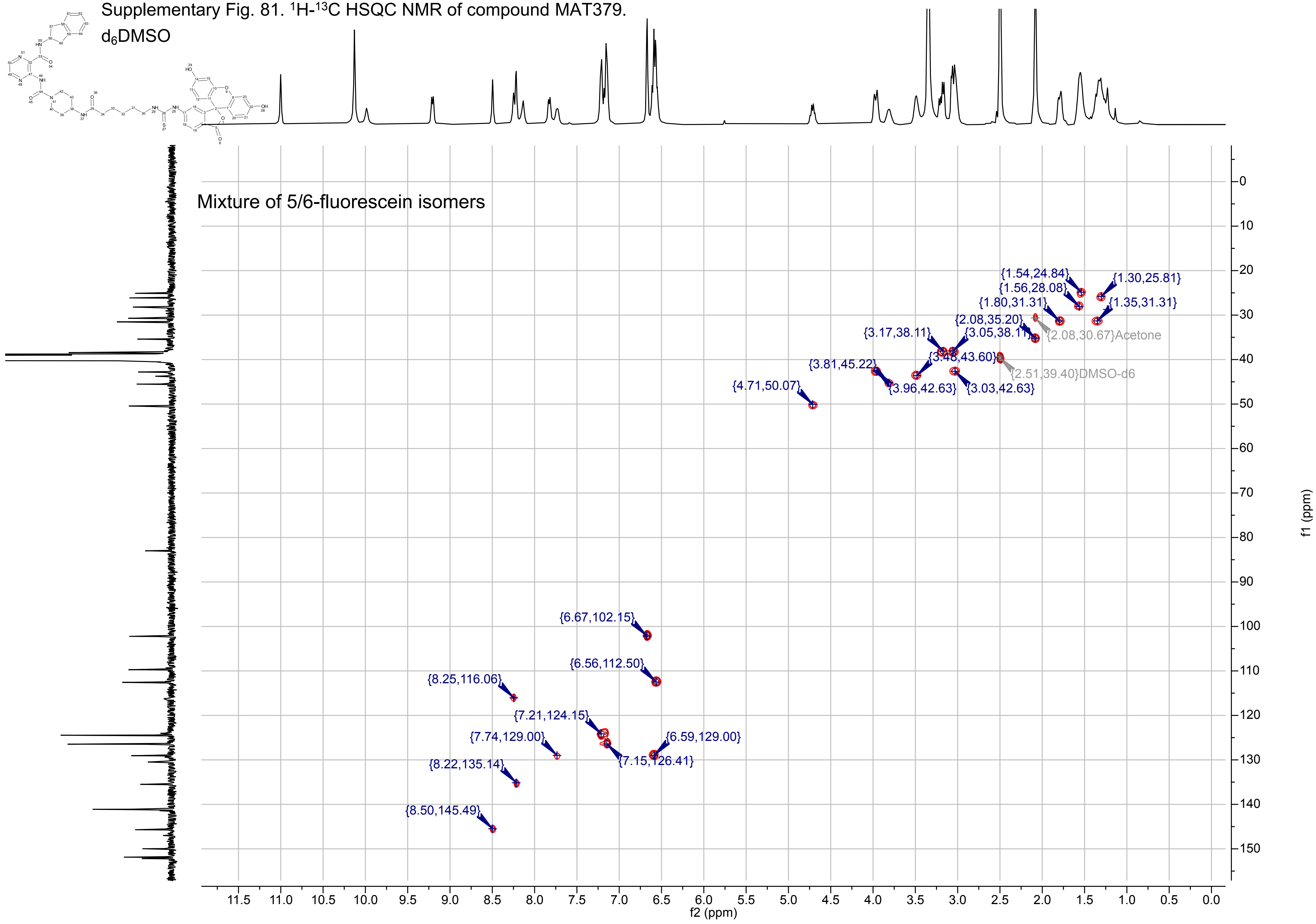

Supplementary Fig. 82. ^{1}H NMR of compound **43**.
$CDCl_3$

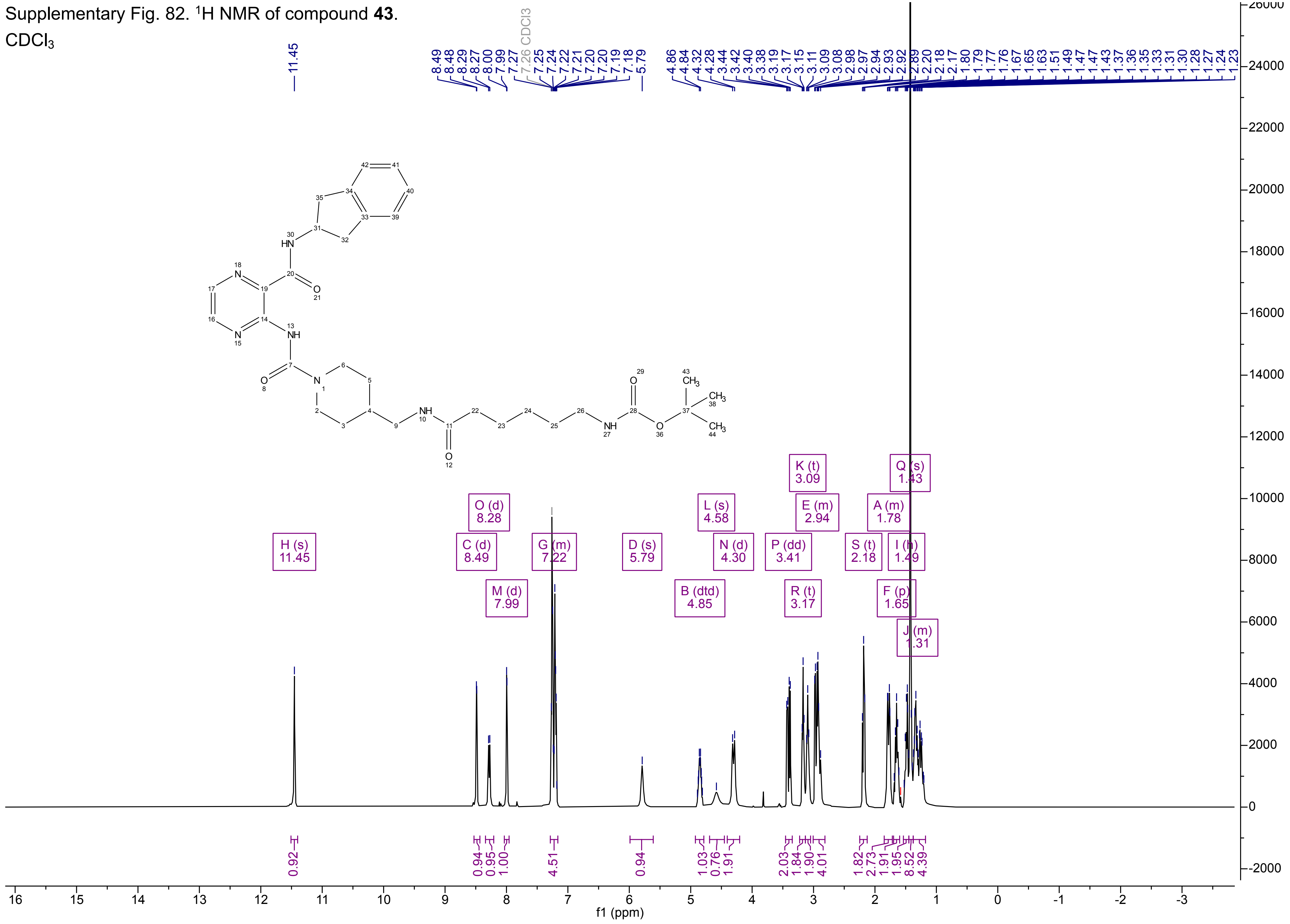

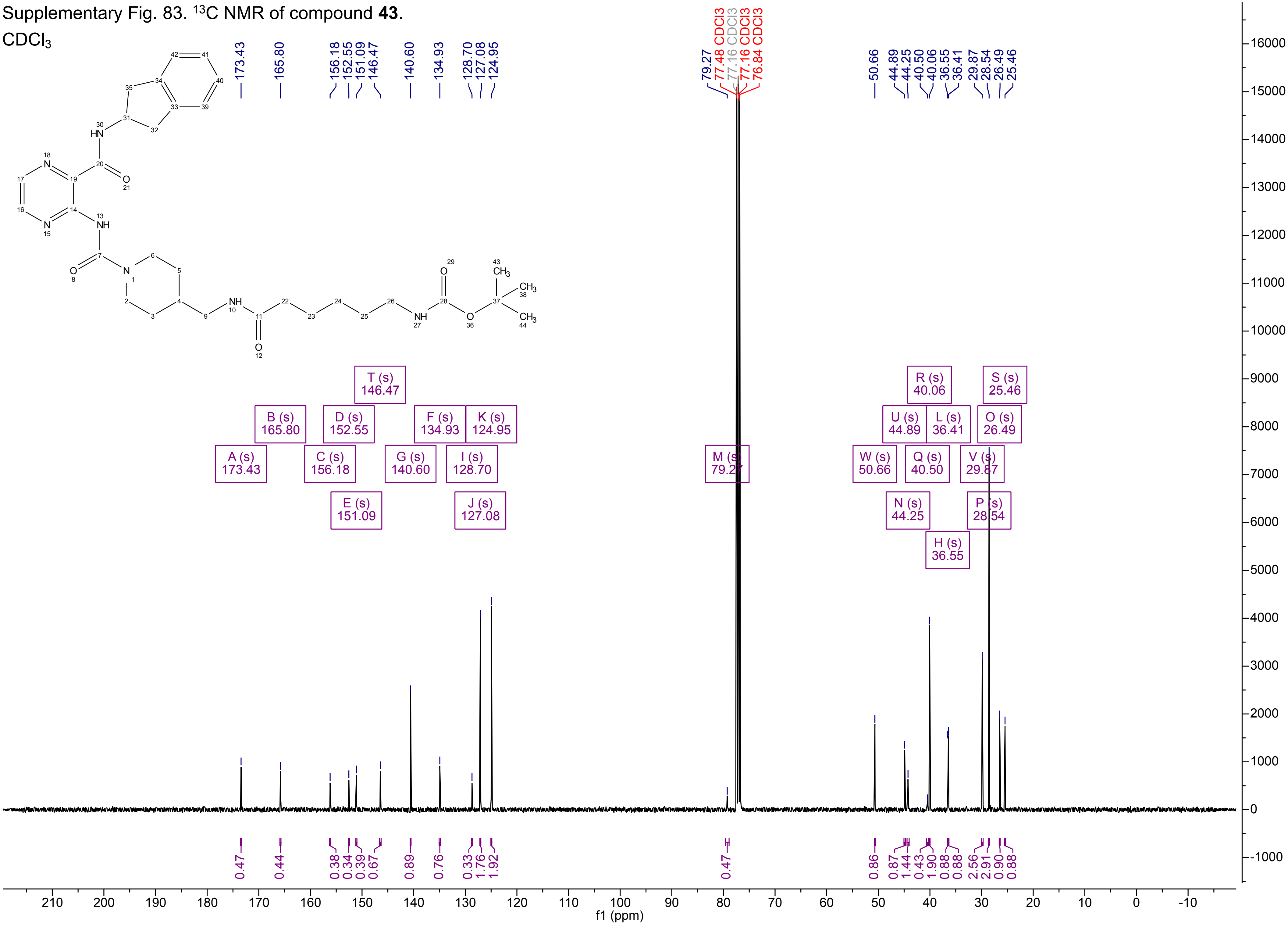

Supplementary Fig. 83. ^{13}C NMR of compound **43**.

$CDCl_3$

Supplementary Fig. 84. ^{1}H NMR of compound **44**.
CD_3OD

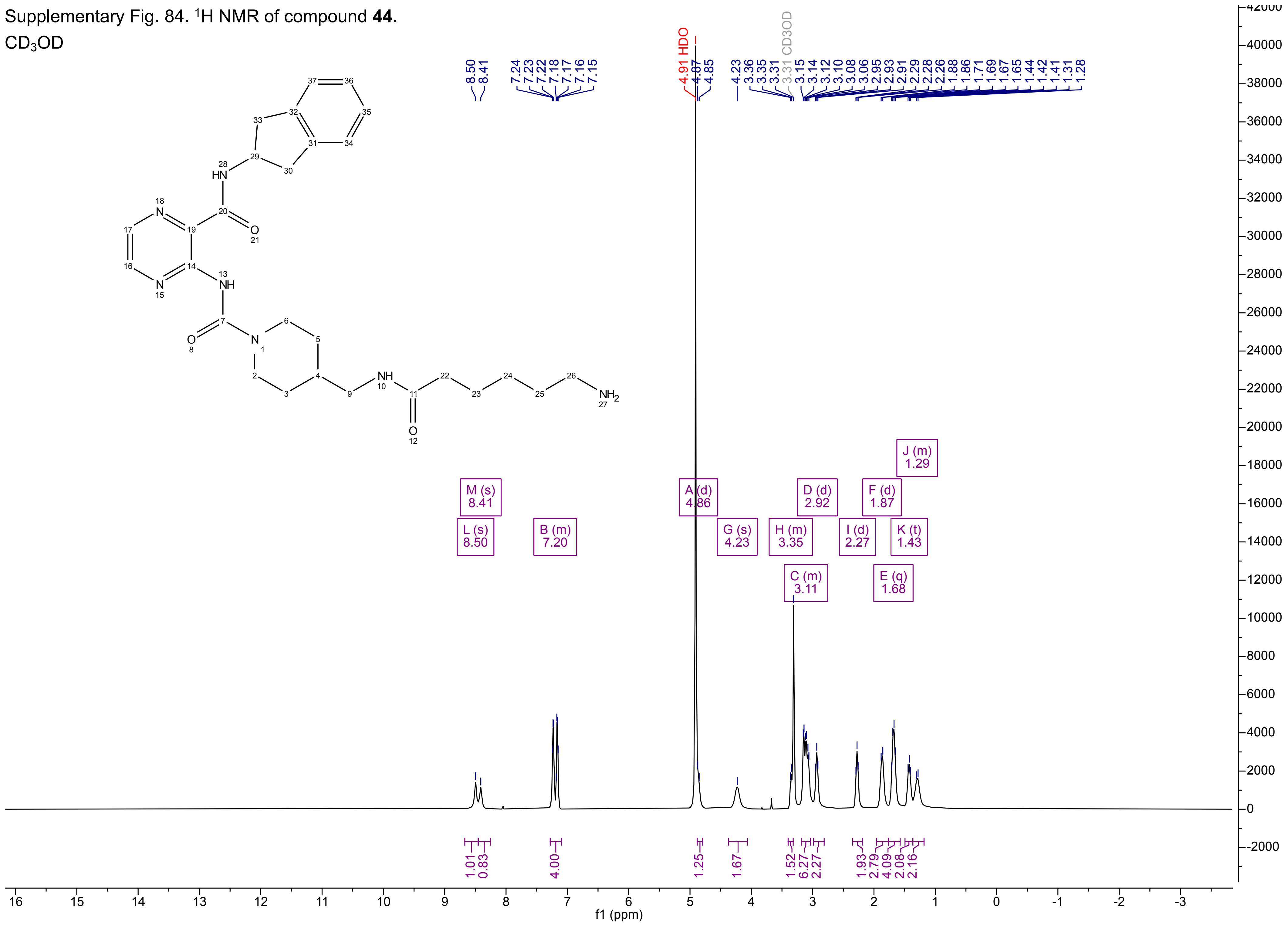

Supplementary Fig. 85. ^{13}C NMR of compound **44**.

CD_3OD

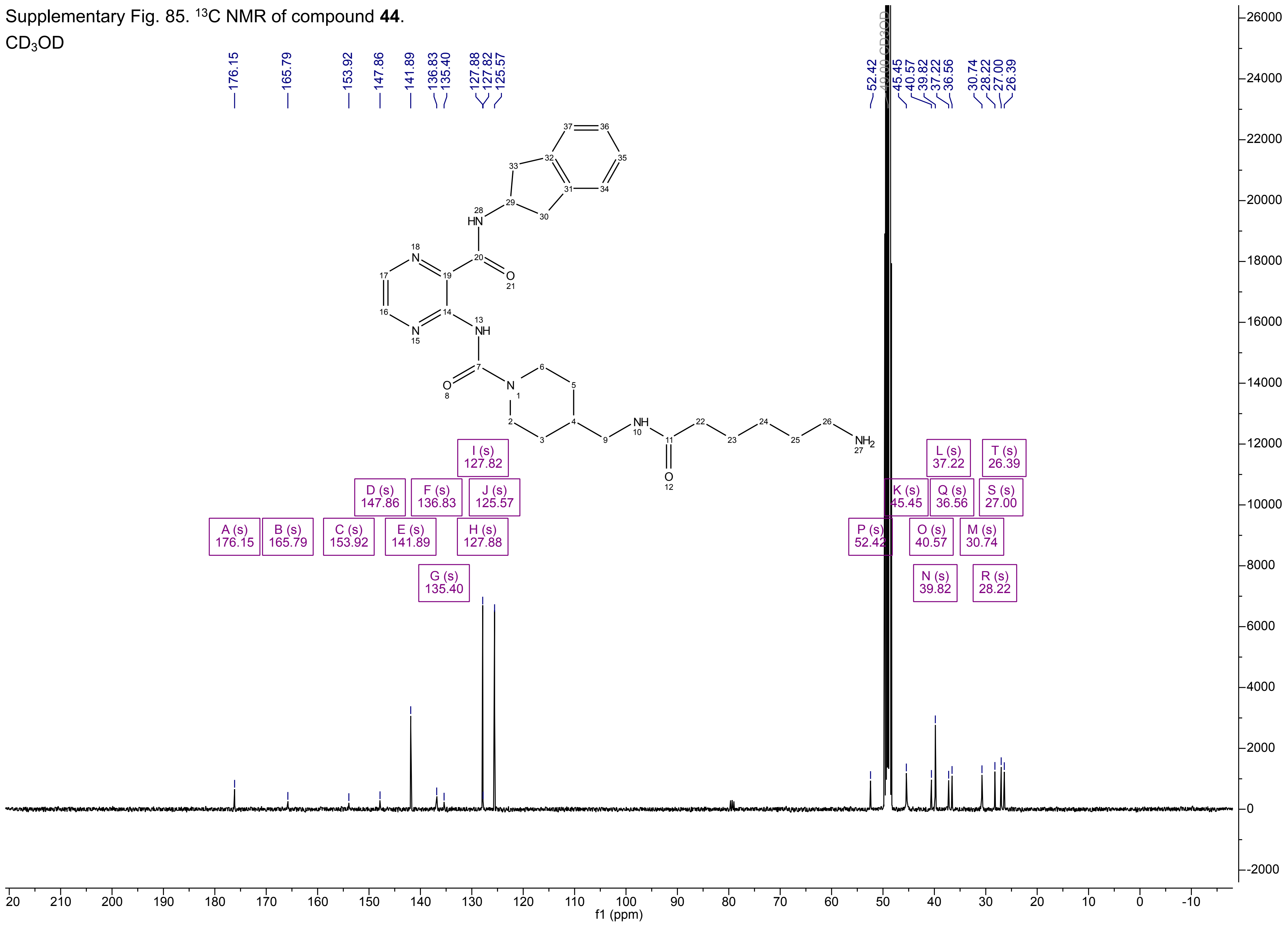

Supplementary Fig. 86. ^{1}H NMR of compound MAT425.

d_6DMSO

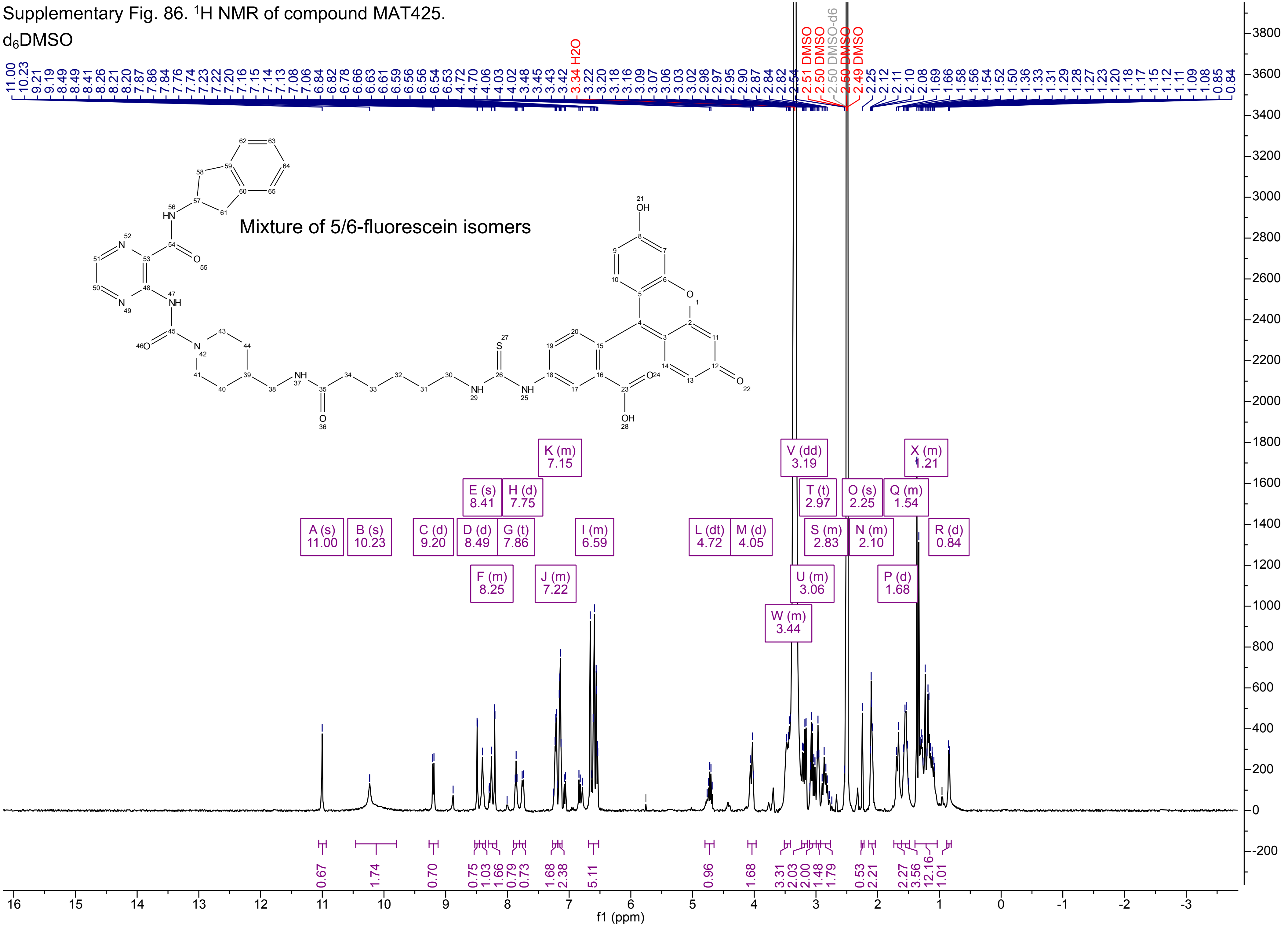

Supplementary Fig. 87. ^{1}H NMR of compound **31**.

d$_6$DMSO

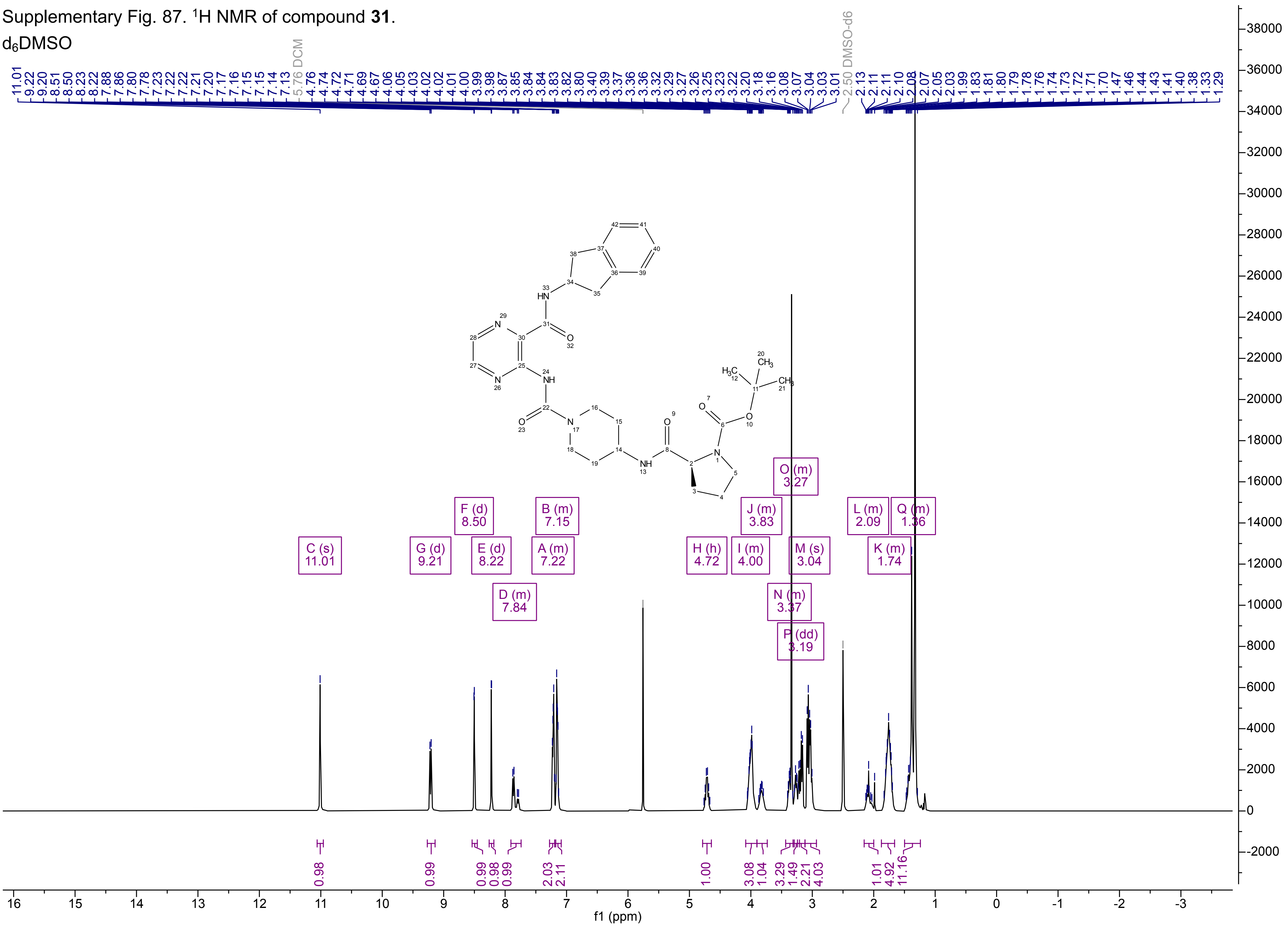

Supplementary Fig. 88. ^{13}C NMR of compound **31**.

d_6DMSO

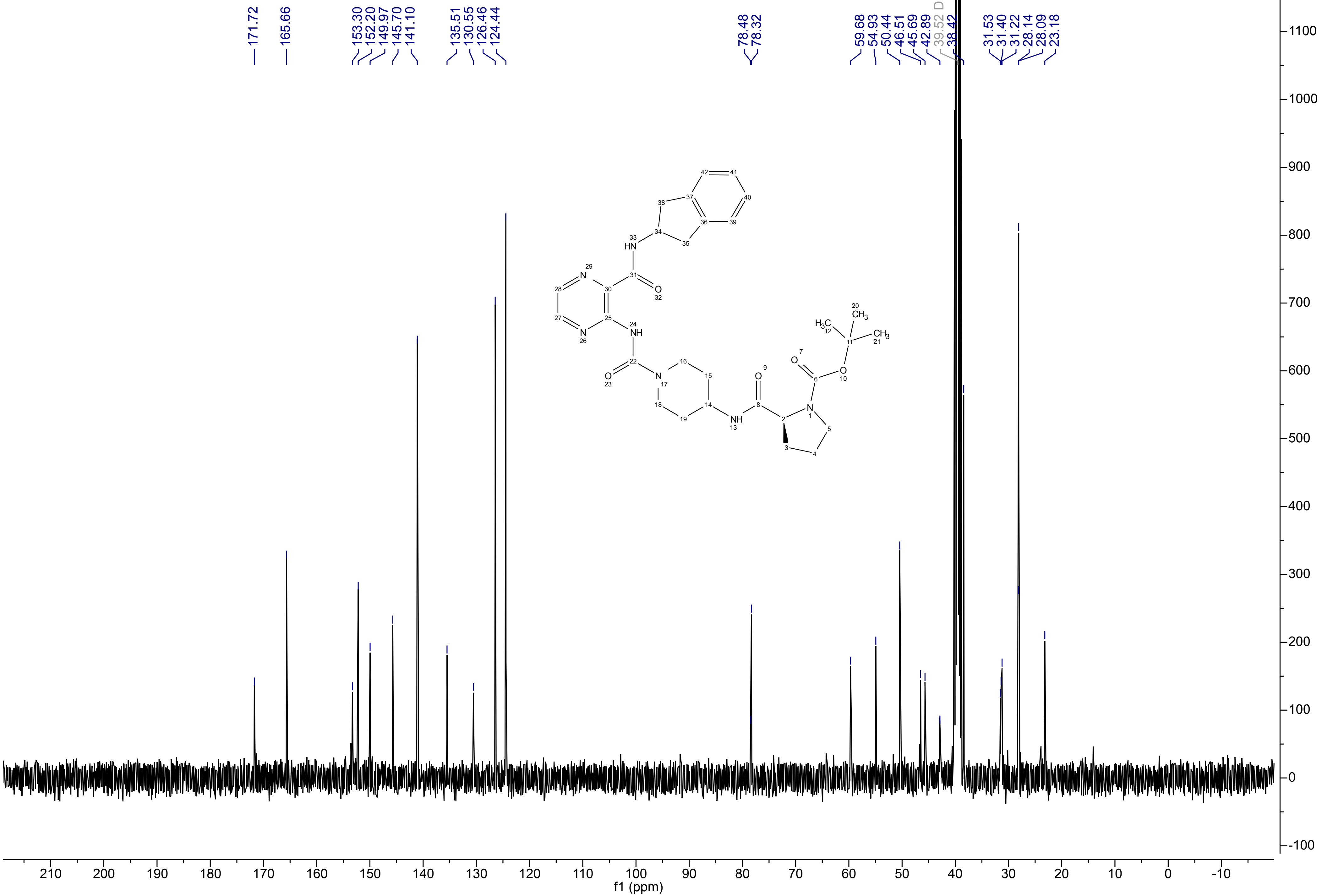

Supplementary Fig. 89. ^{1}H NMR of compound MAT334.

d_6DMSO

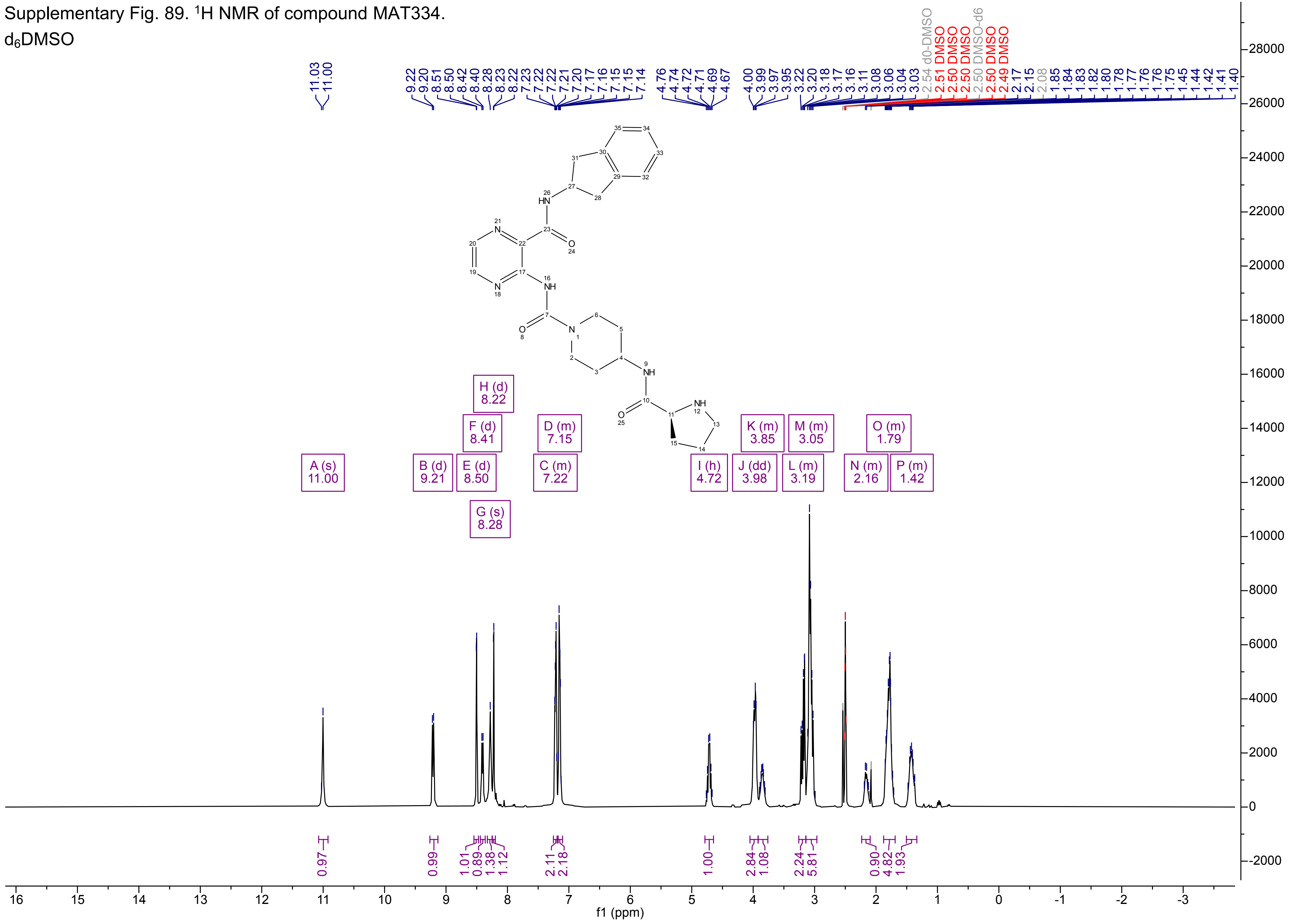

Supplementary Fig. 90. ^{13}C NMR of compound MAT334.
d_6DMSO

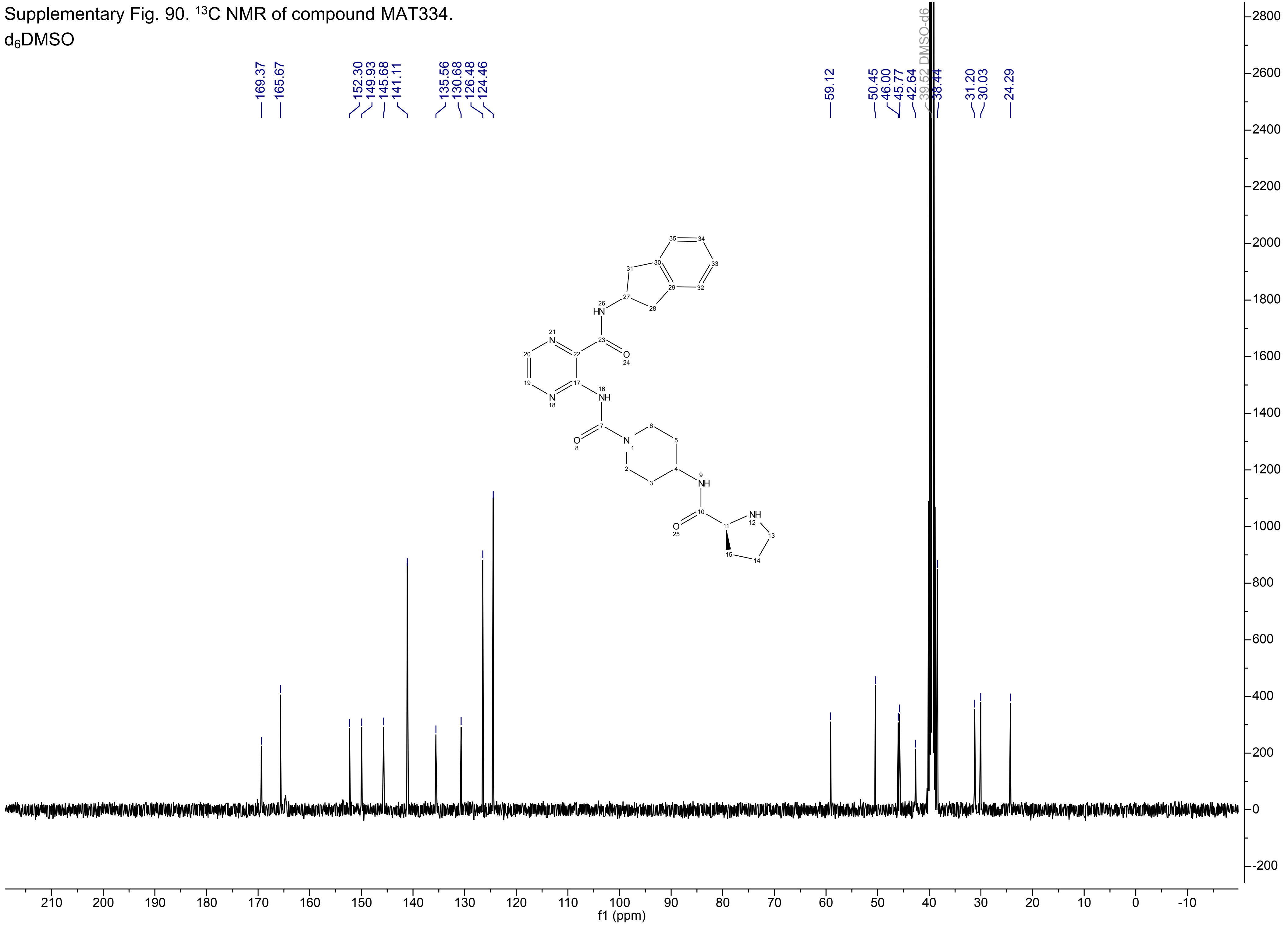

Supplementary Fig. 91. ^{1}H NMR of compound **32**.

d$_6$DMSO

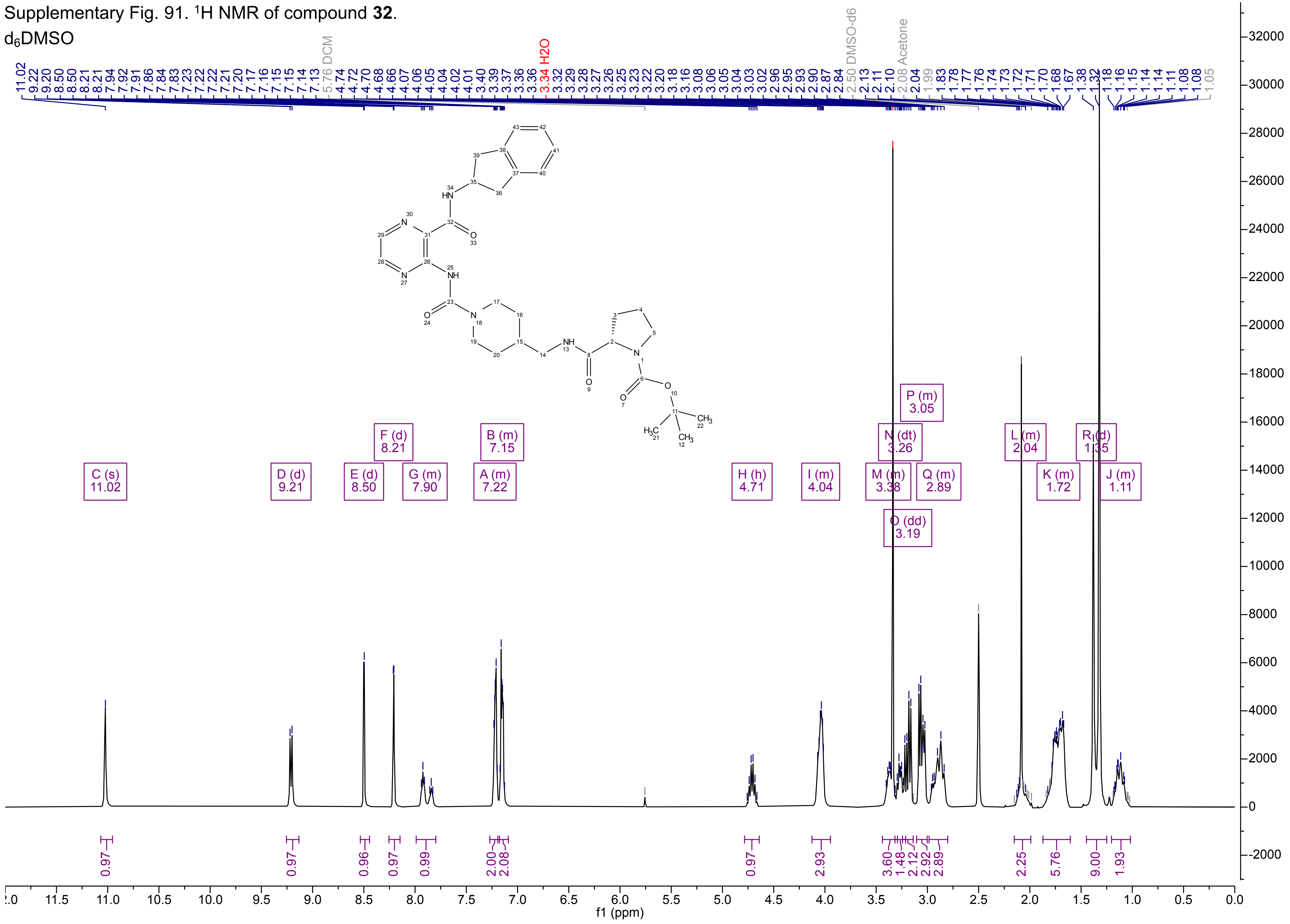

Supplementary Fig. 92. ^{13}C NMR of compound **32**.
d_6DMSO

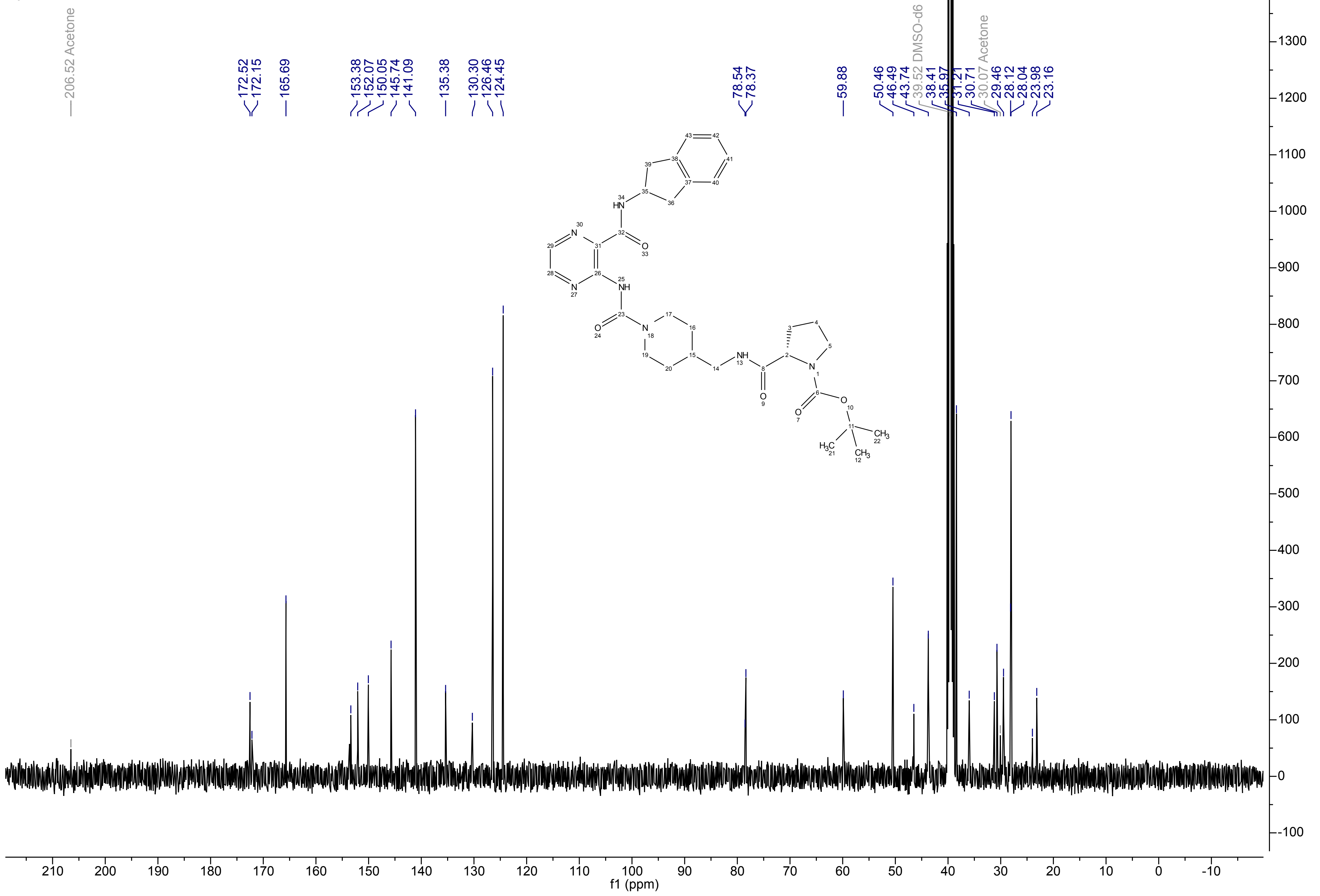

Supplementary Fig. 93. ^{1}H NMR of compound MAT345.
d_6DMSO

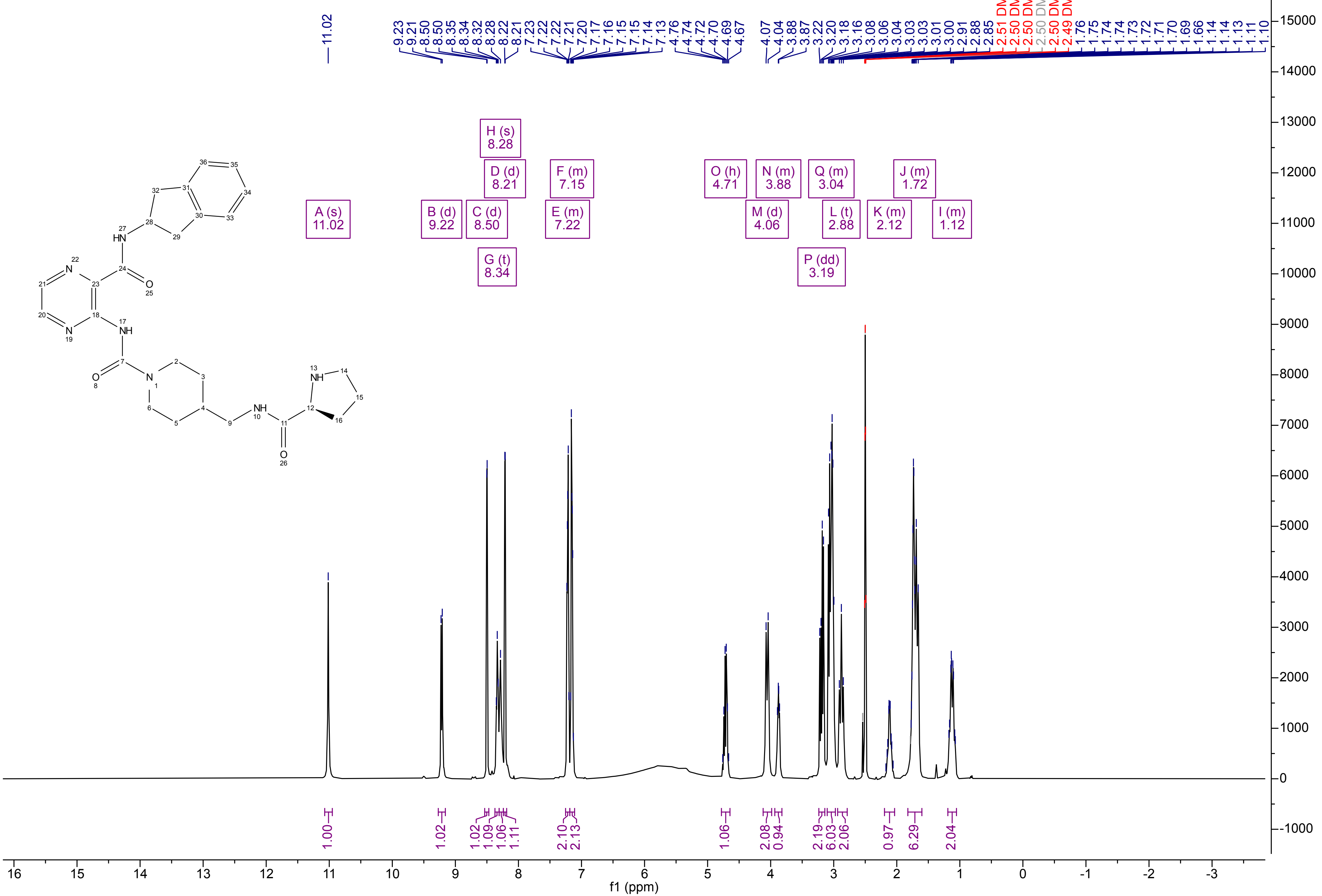

Supplementary Fig. 94. ^{13}C NMR of compound MAT345.
d_6DMSO

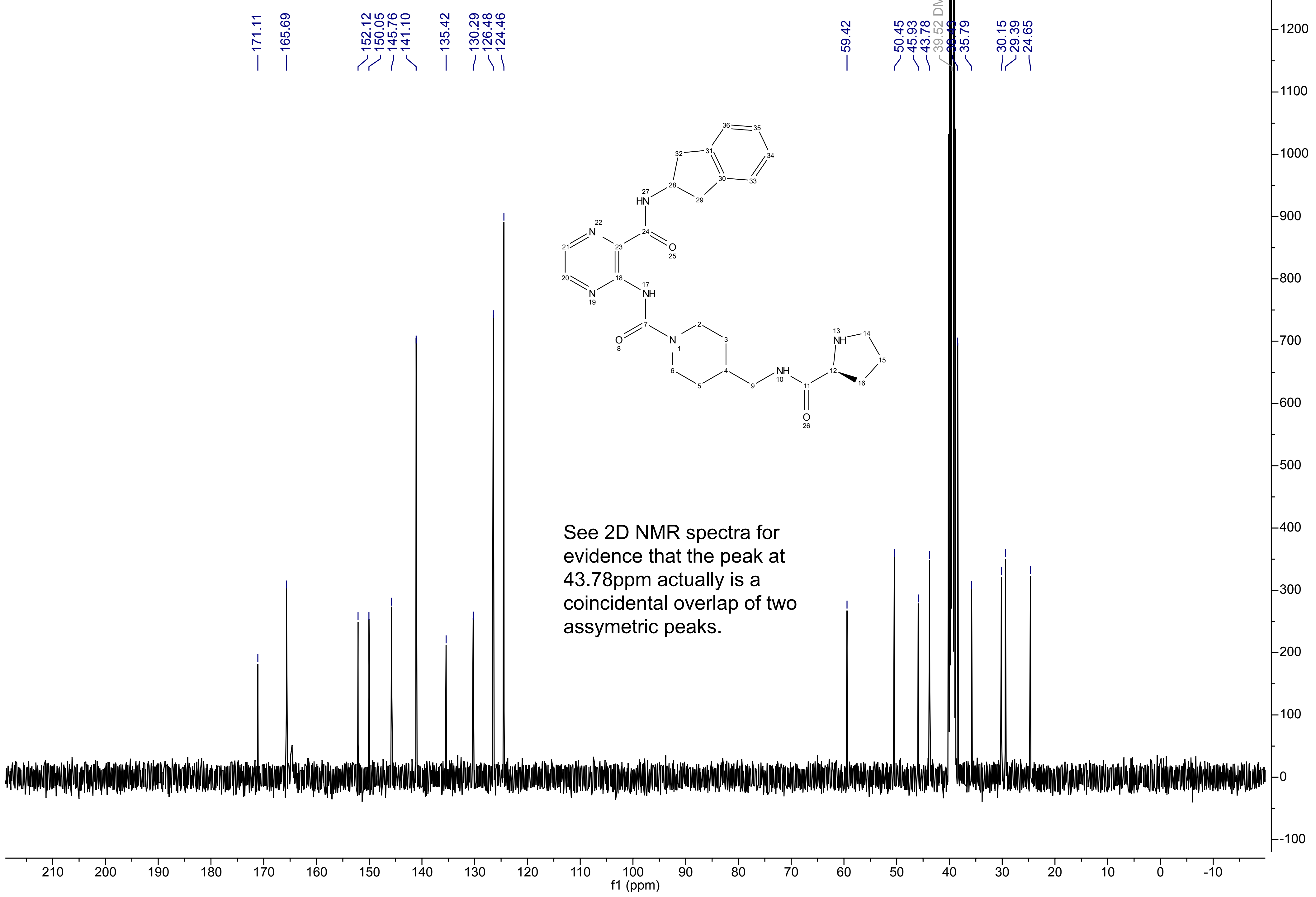

Supplementary Fig. 95. DEPT-135 NMR of compound MAT345.

d_6DMSO

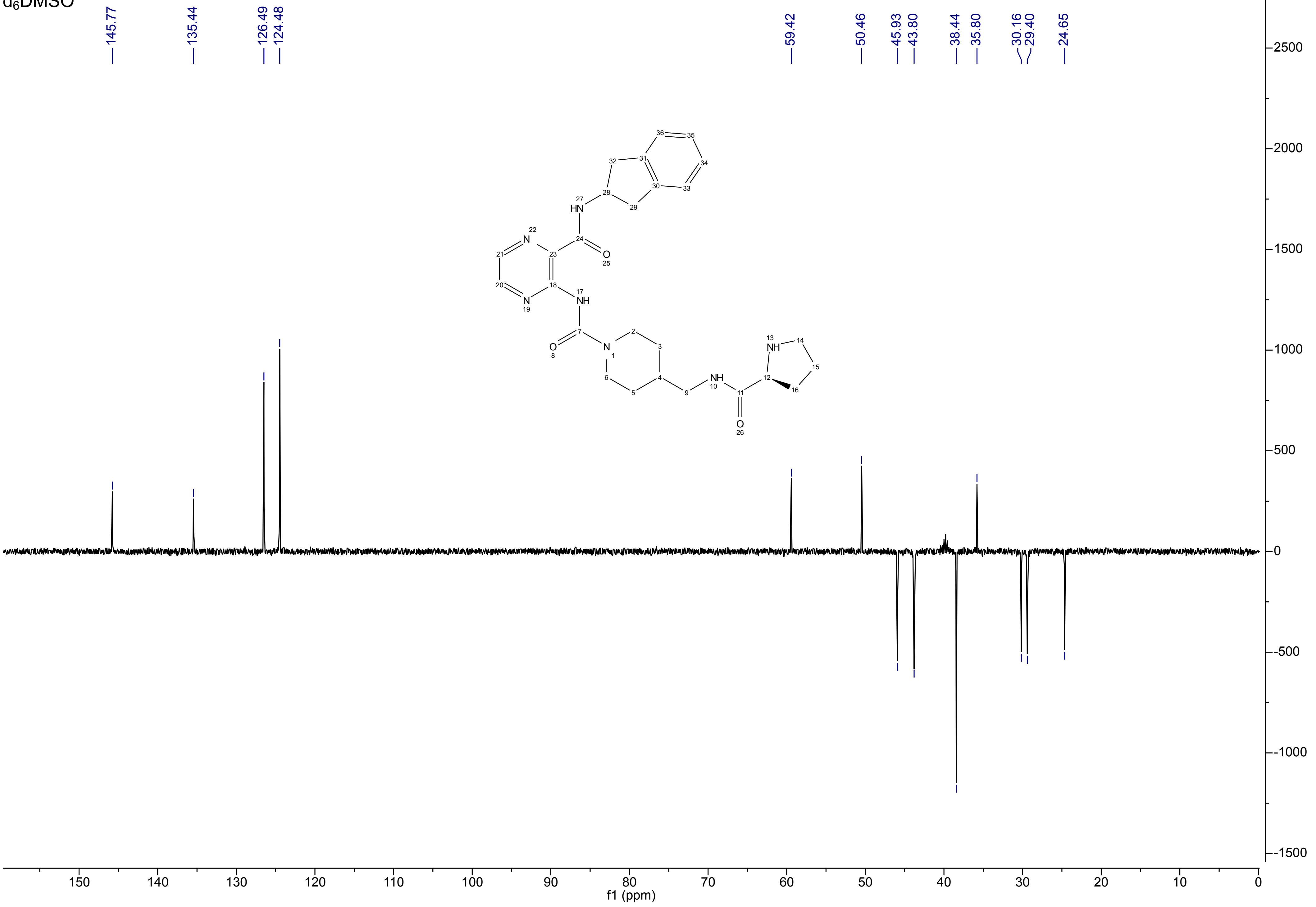

Supplementary Fig. 96. ^1H-^1H COSY NMR of compound MAT345.
d_6DMSO

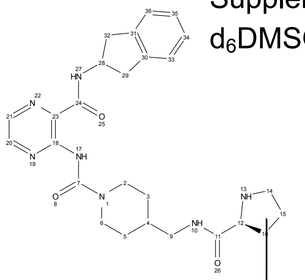

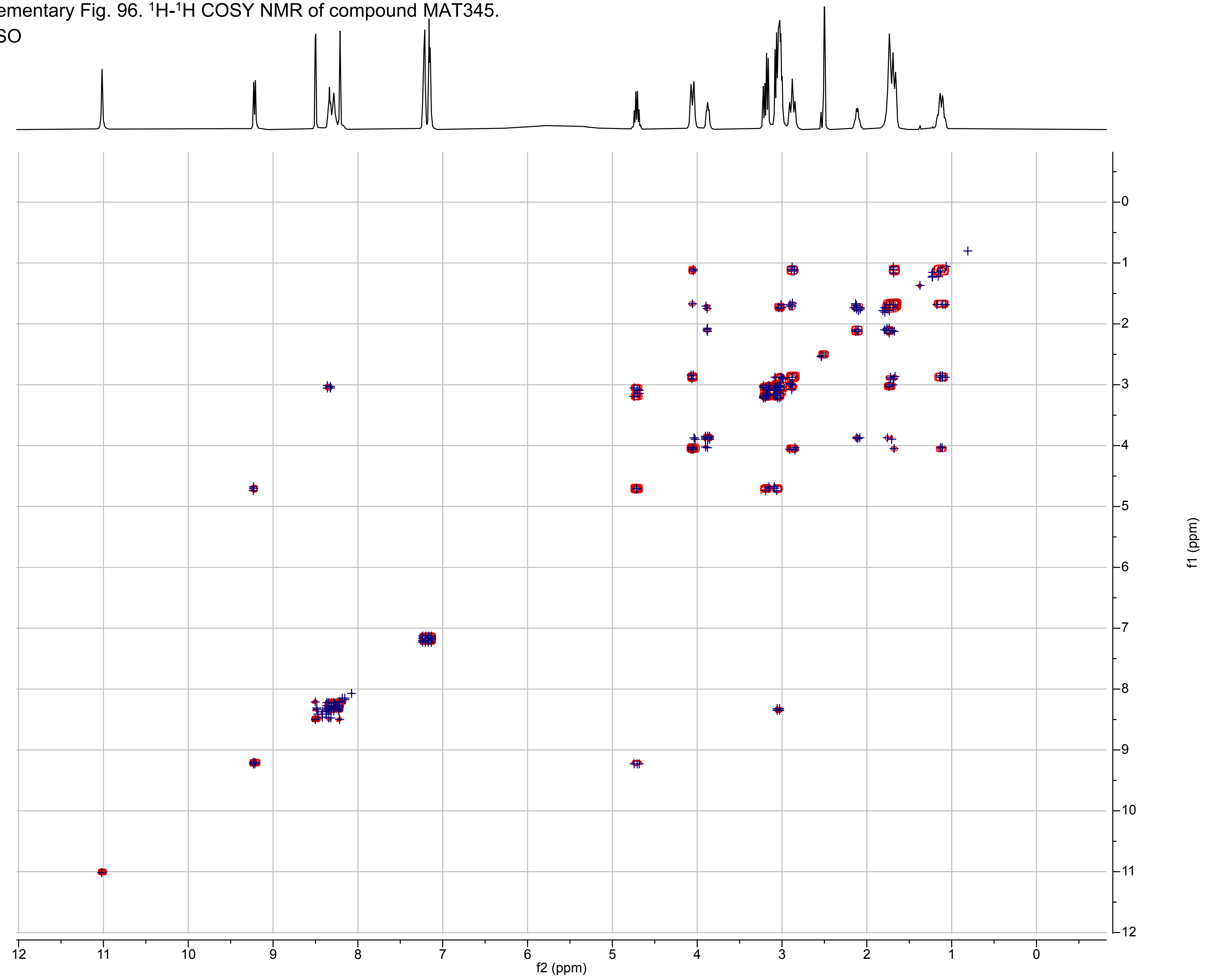

Supplementary Fig. 97. ^1H-^{13}C HMBC NMR of compound MAT345.
d_6DMSO

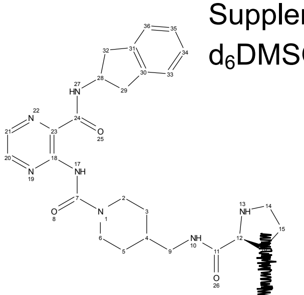

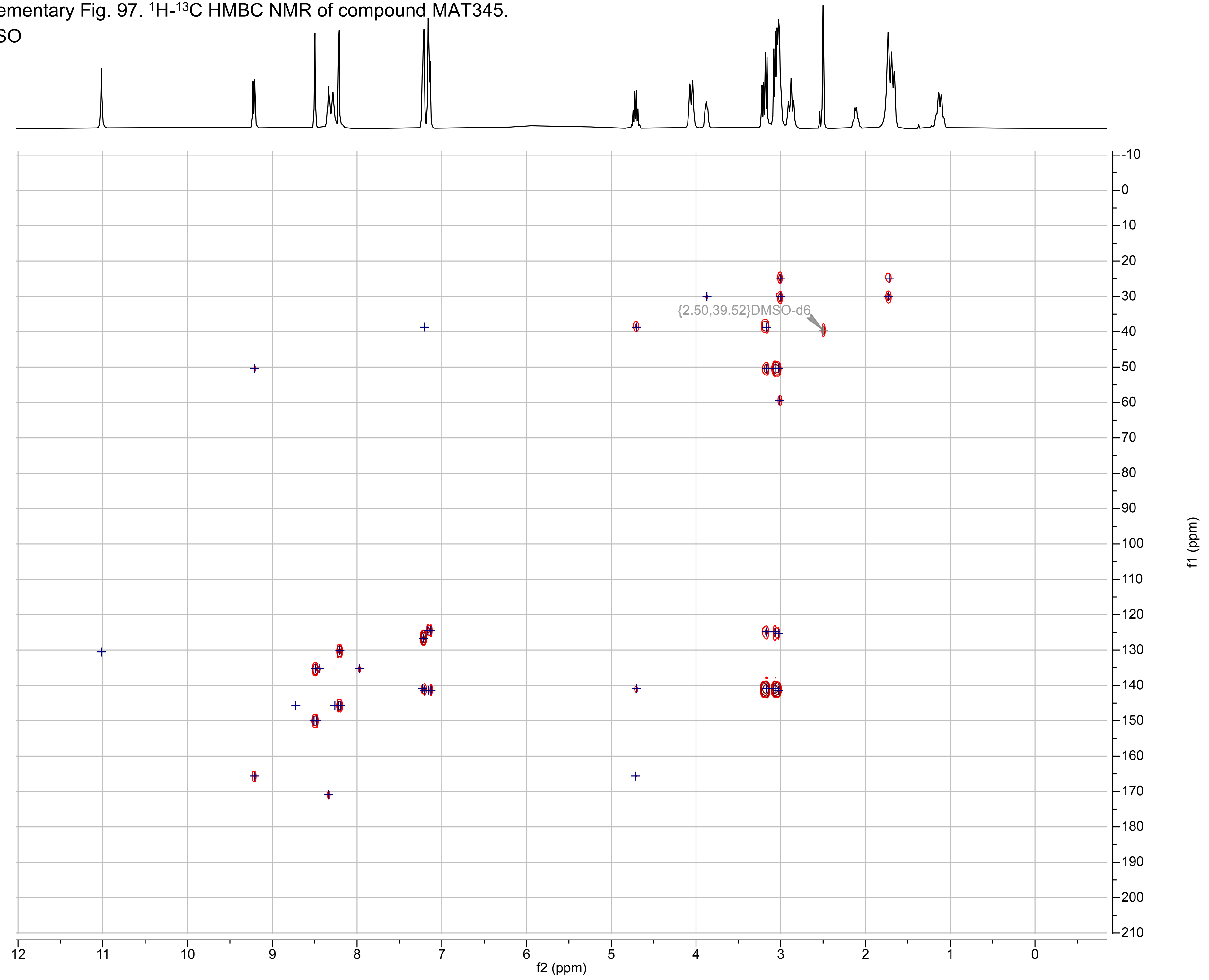

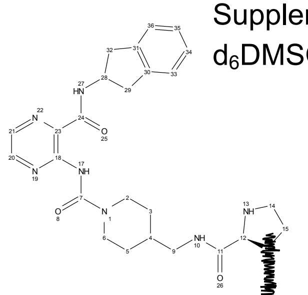

Supplementary Fig. 98. ^1H-^{13}C HSQC NMR of compound MAT345.
d_6DMSO

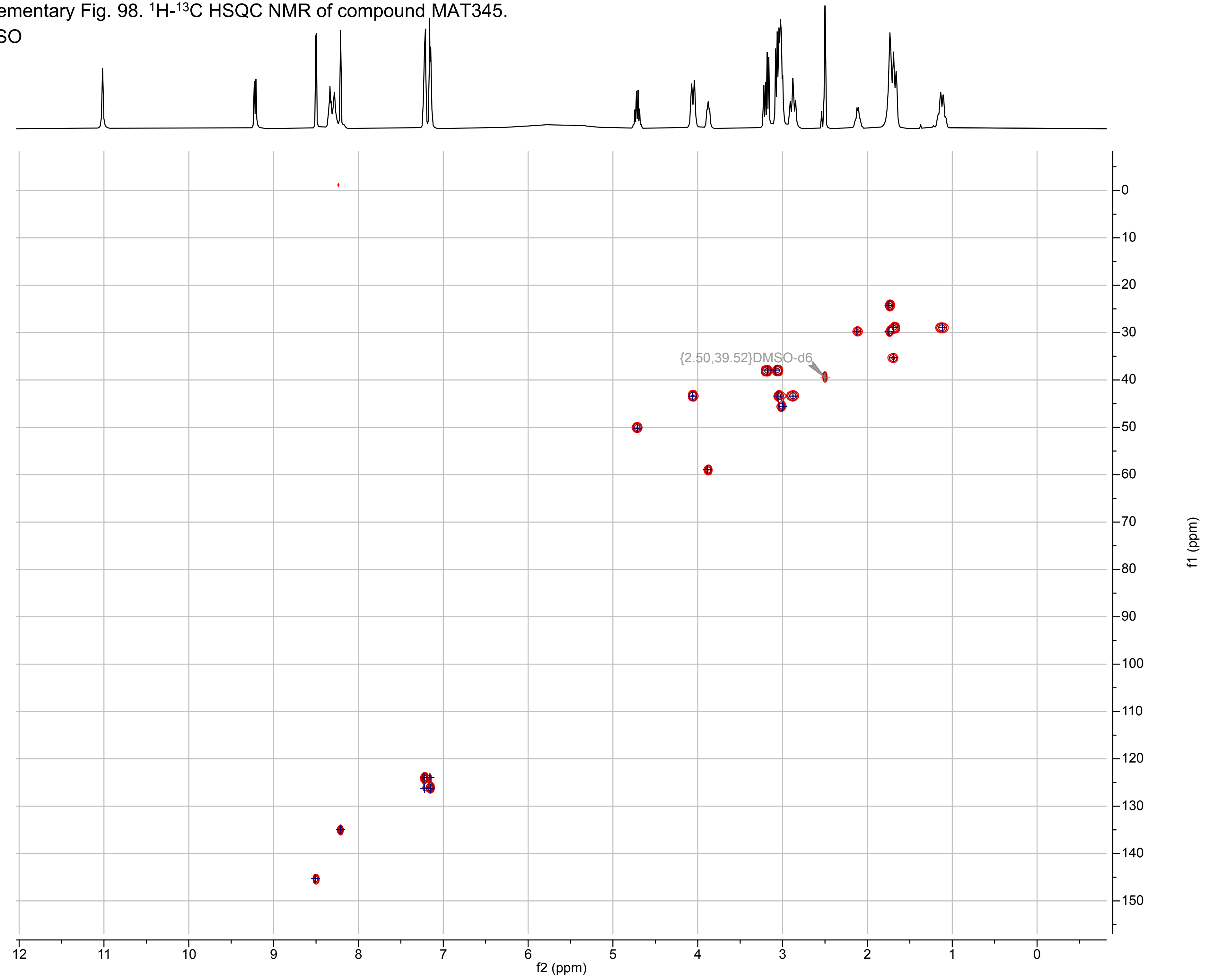

Supplementary Fig. 99. ^{1}H NMR of compound **54**.
$CDCl_3$

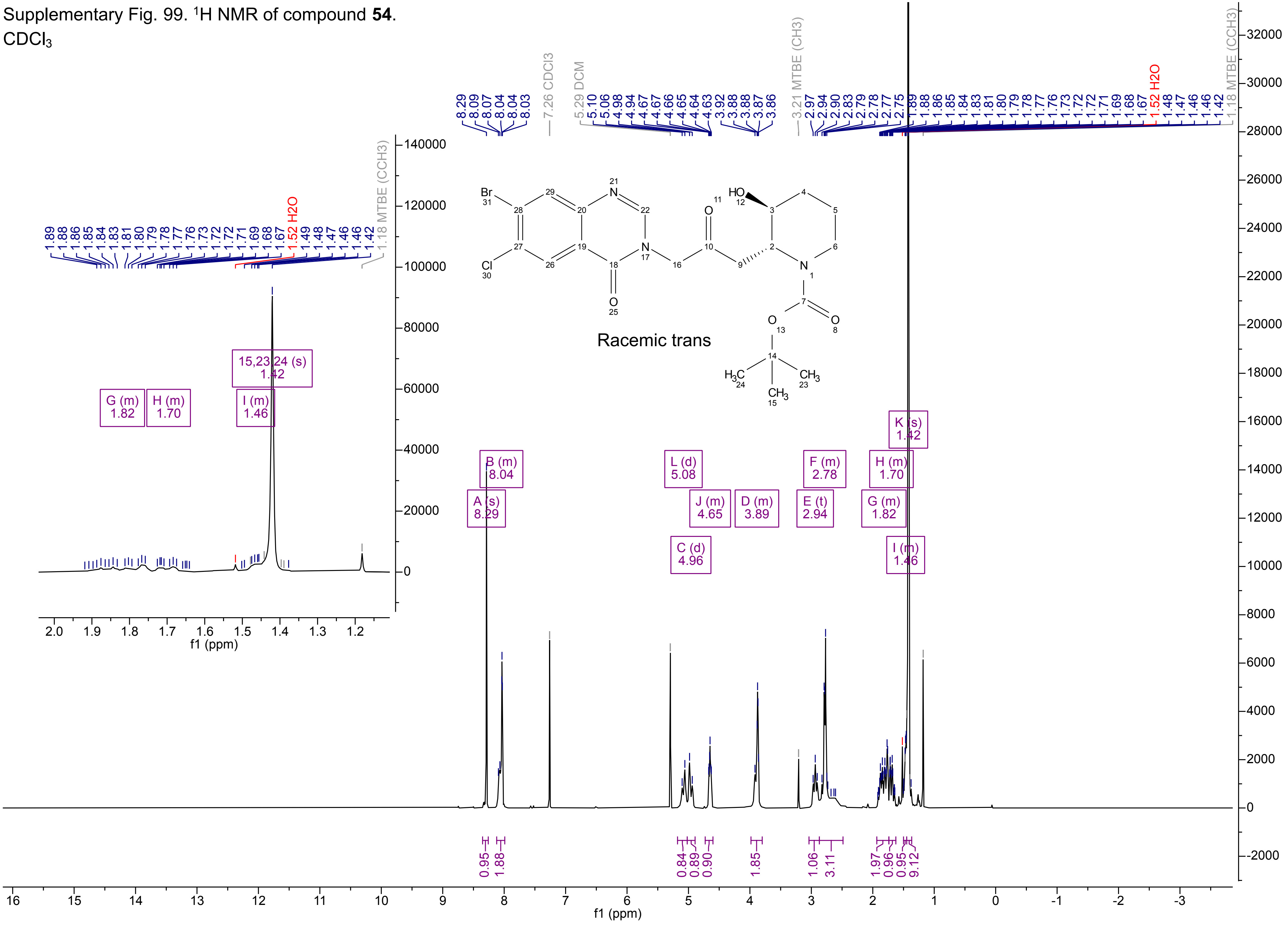

Supplementary Fig. 100. ^{13}C NMR of compound **54**.
$CDCl_3$

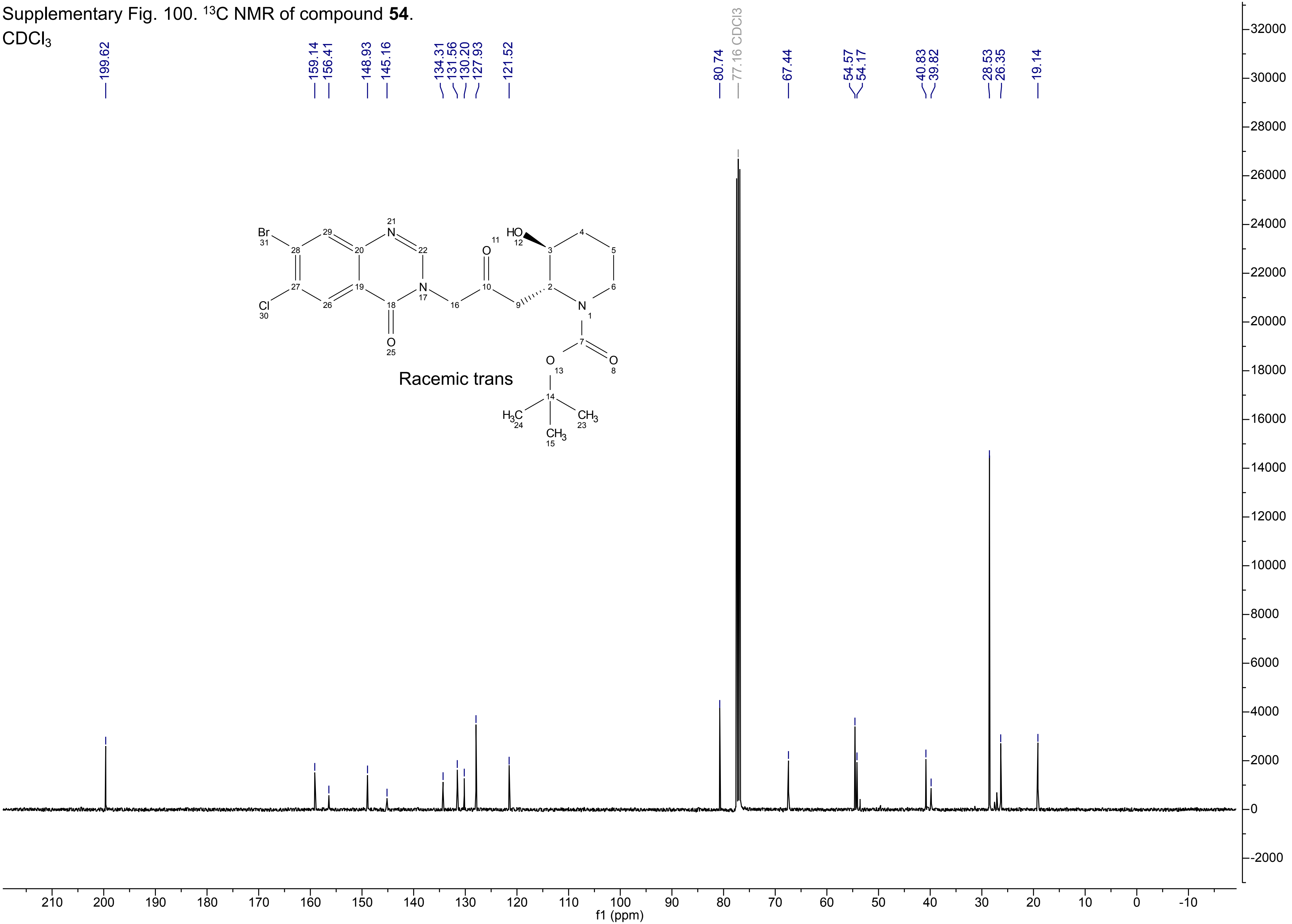

Supplementary Fig. 101. ^{1}H NMR of compound **37**.

d$_6$DMSO

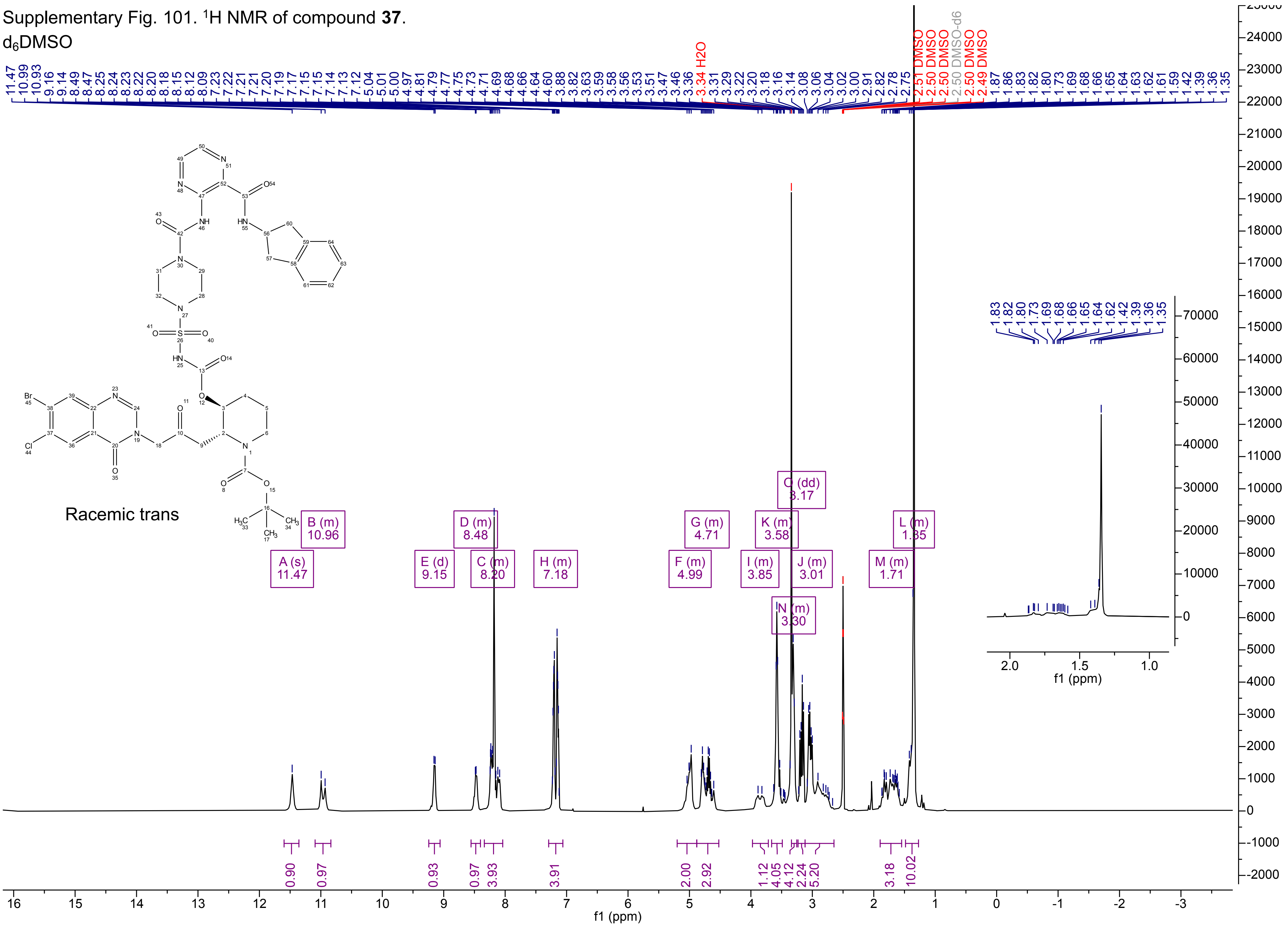

Supplementary Fig. 102. ^{13}C NMR of compound **37**.
d_6DMSO

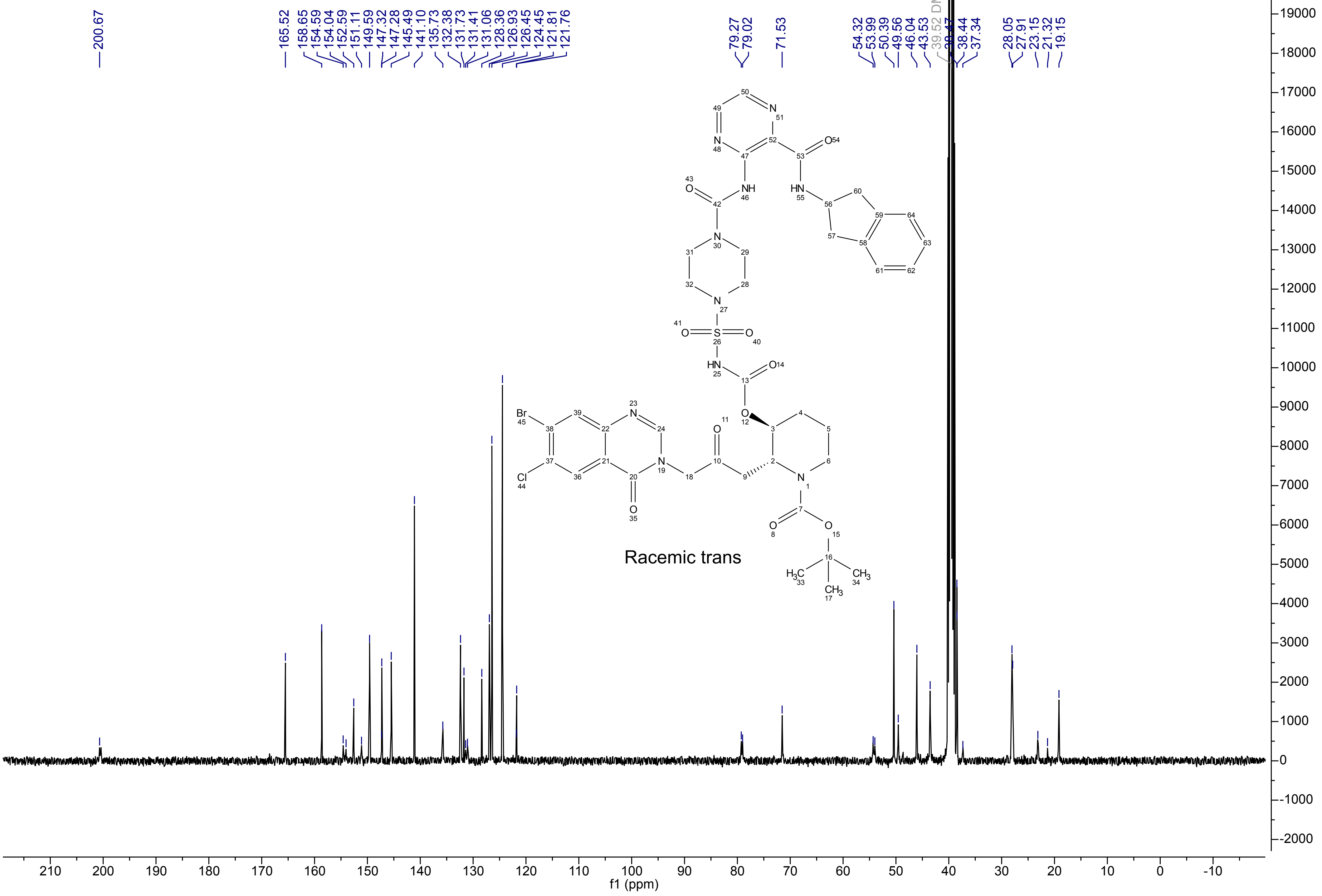

Supplementary Fig. 103. DEPT-135 NMR of compound **37**.

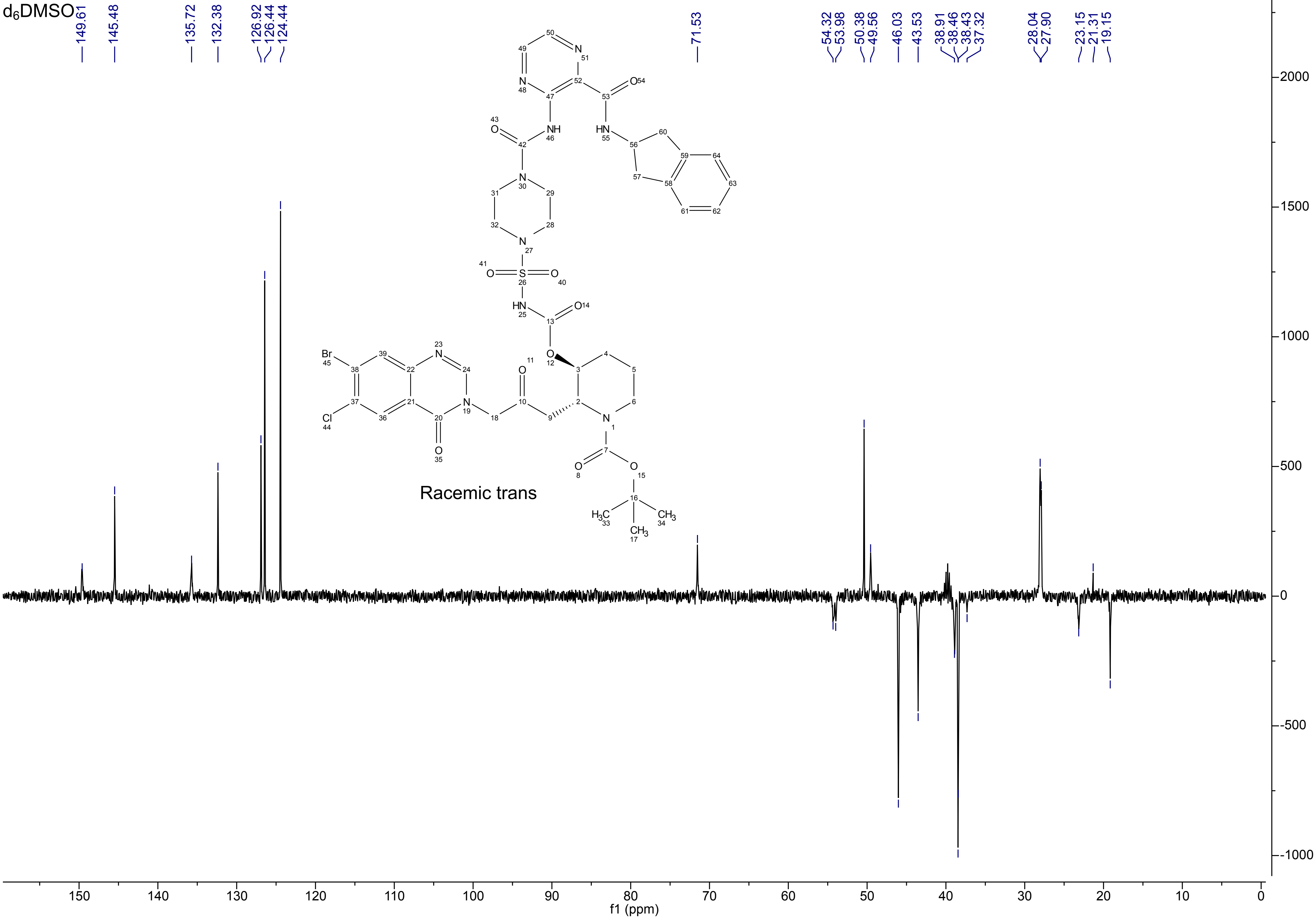

Supplementary Fig. 104. ^{1}H-^{1}H COSY NMR of compound **37**.

d_6DMSO

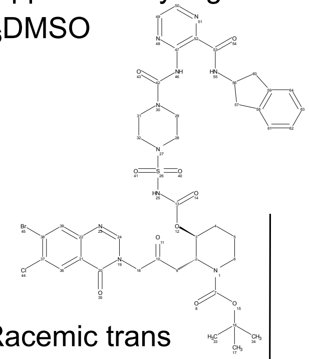

Racemic trans

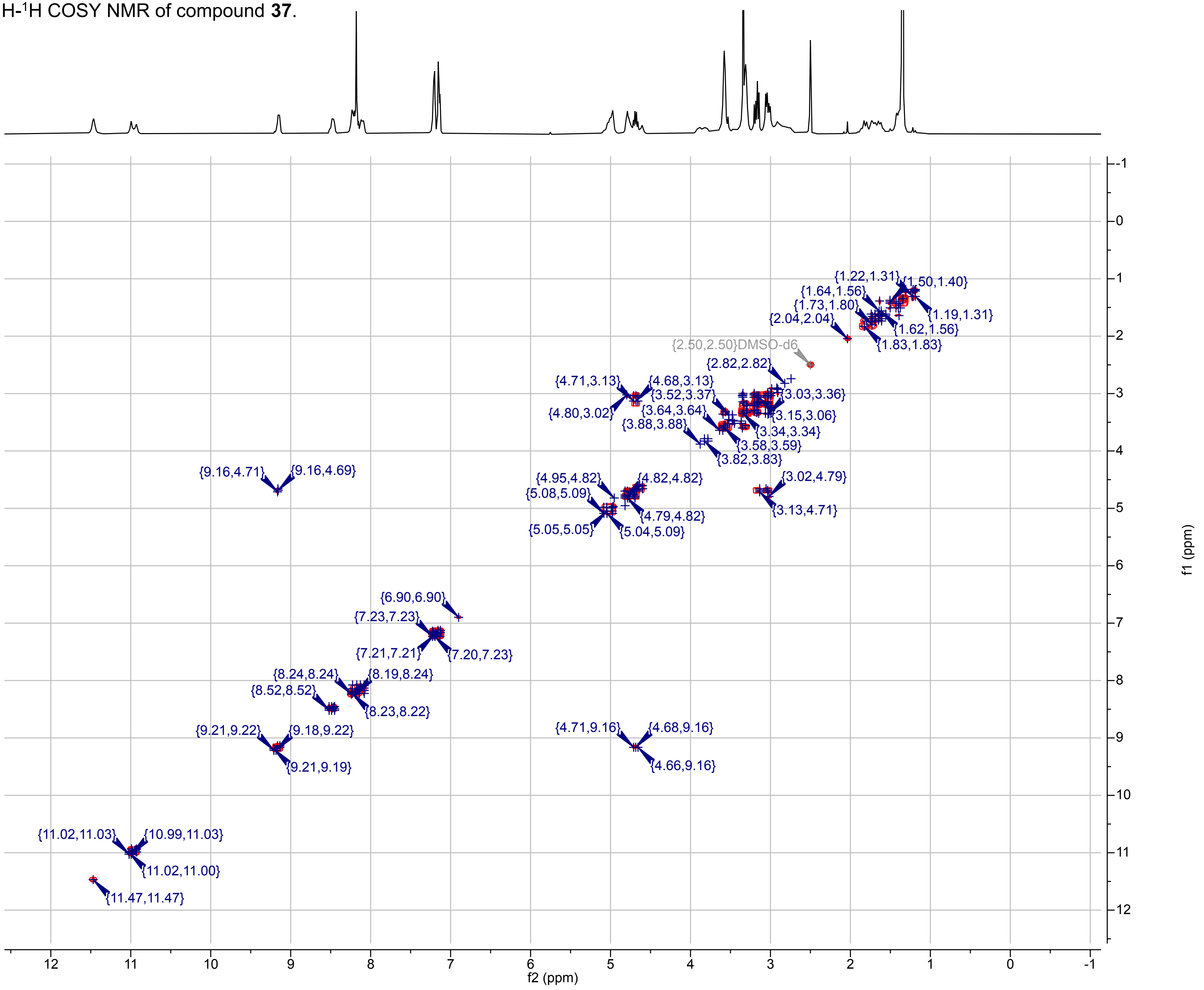

Supplementary Fig. 105. ^1H-^{13}C HMBC NMR of compound **37**.

d_6DMSO

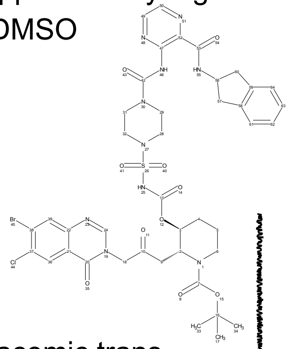

Racemic trans

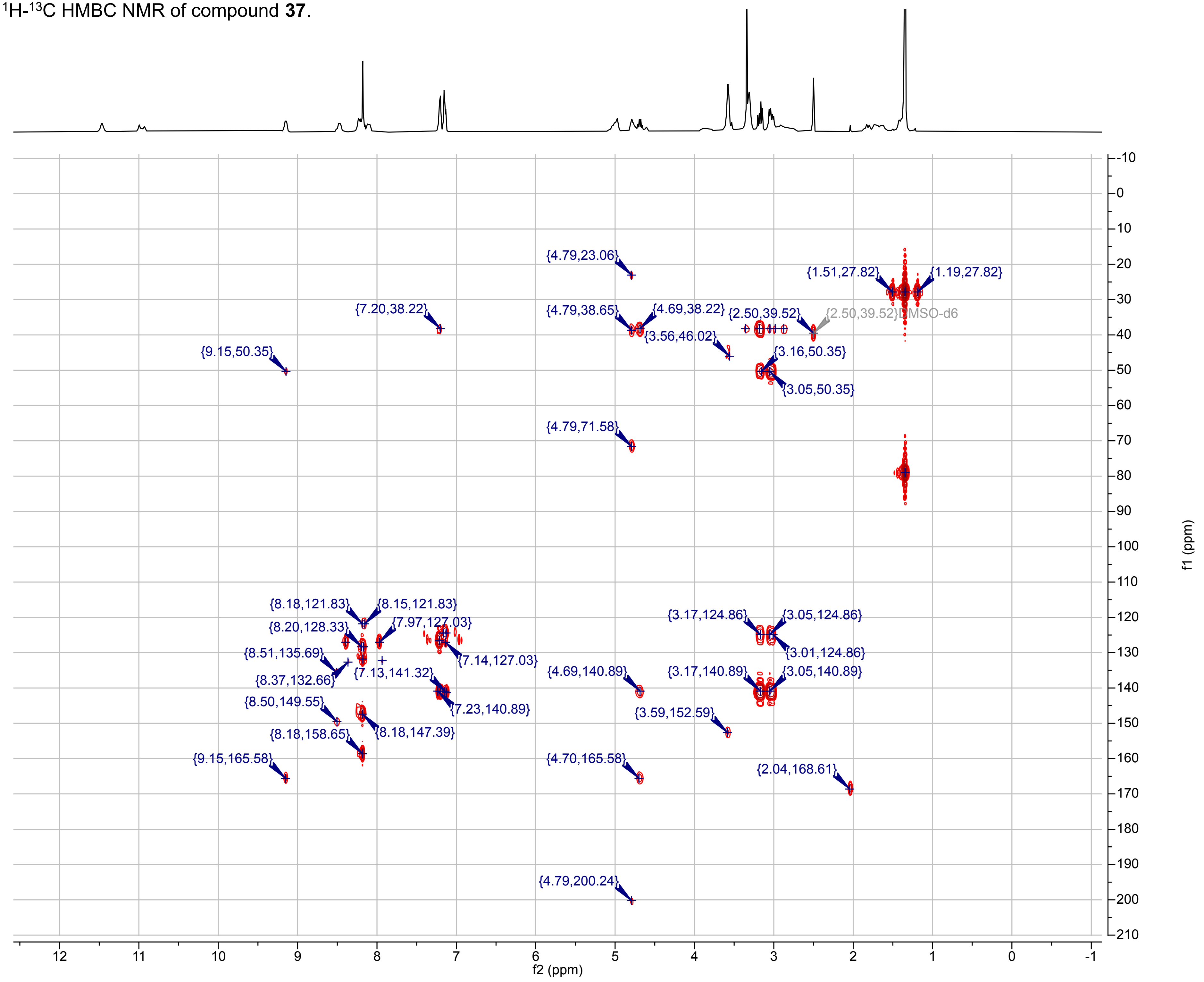

Supplementary Fig. 106. ^{1}H-^{13}C HSQC NMR of compound **37**.
d_6DMSO

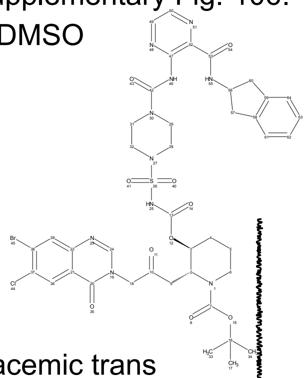

Racemic trans

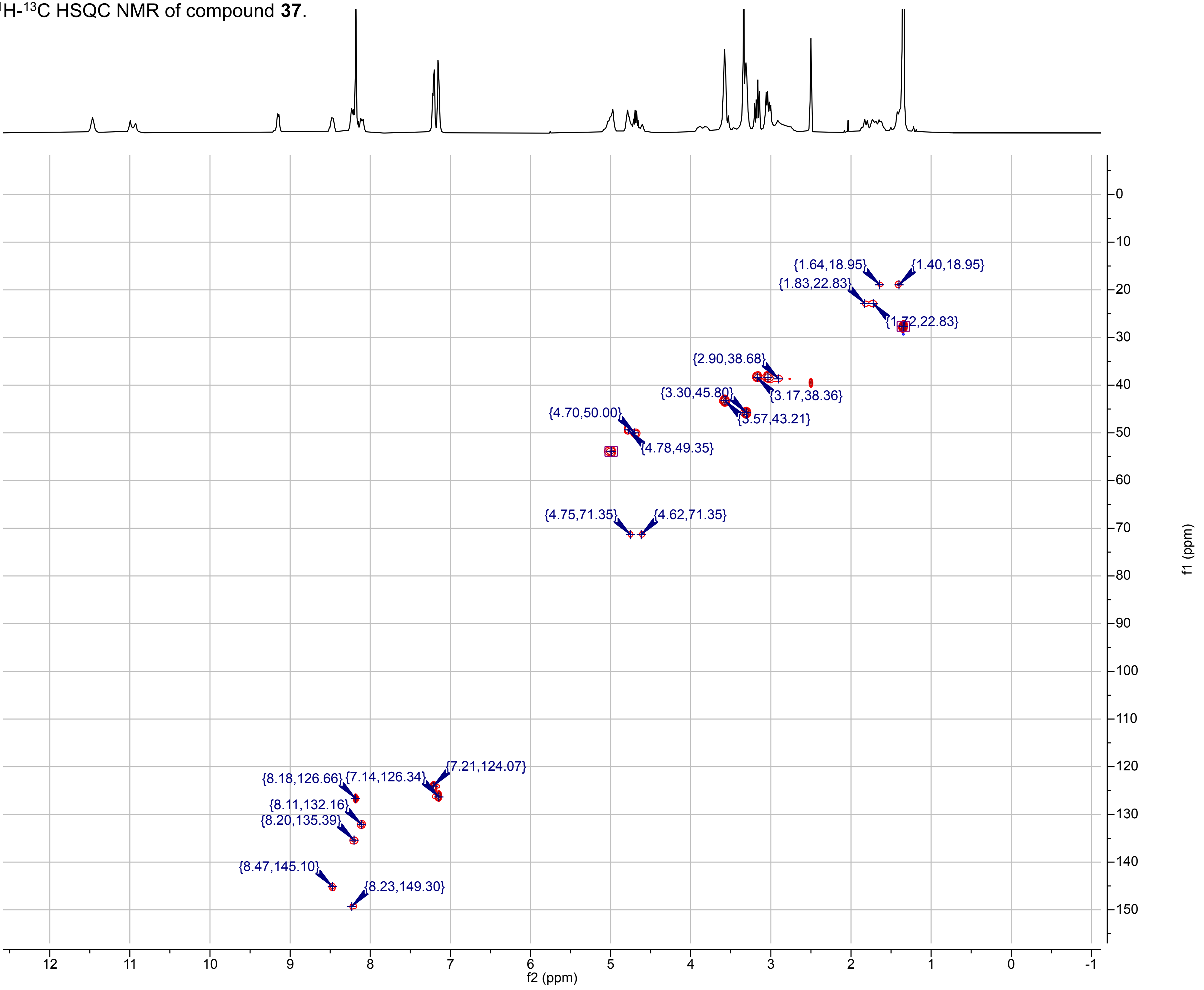

Supplementary Fig. 107. ^1H NMR of compound MAT436.
d_6DMSO

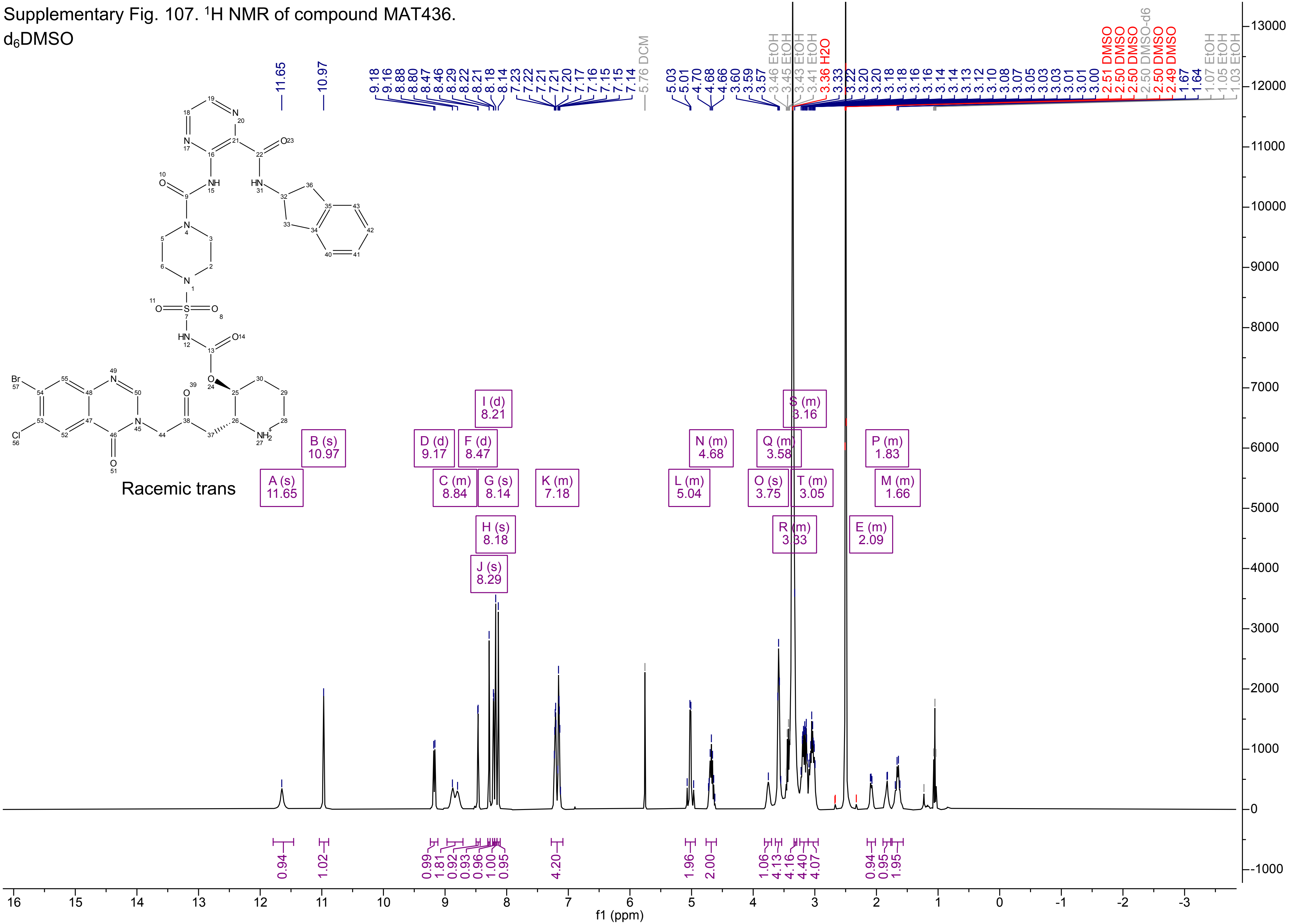

Supplementary Fig. 108. ^{13}C NMR of compound MAT436.
d_6DMSO

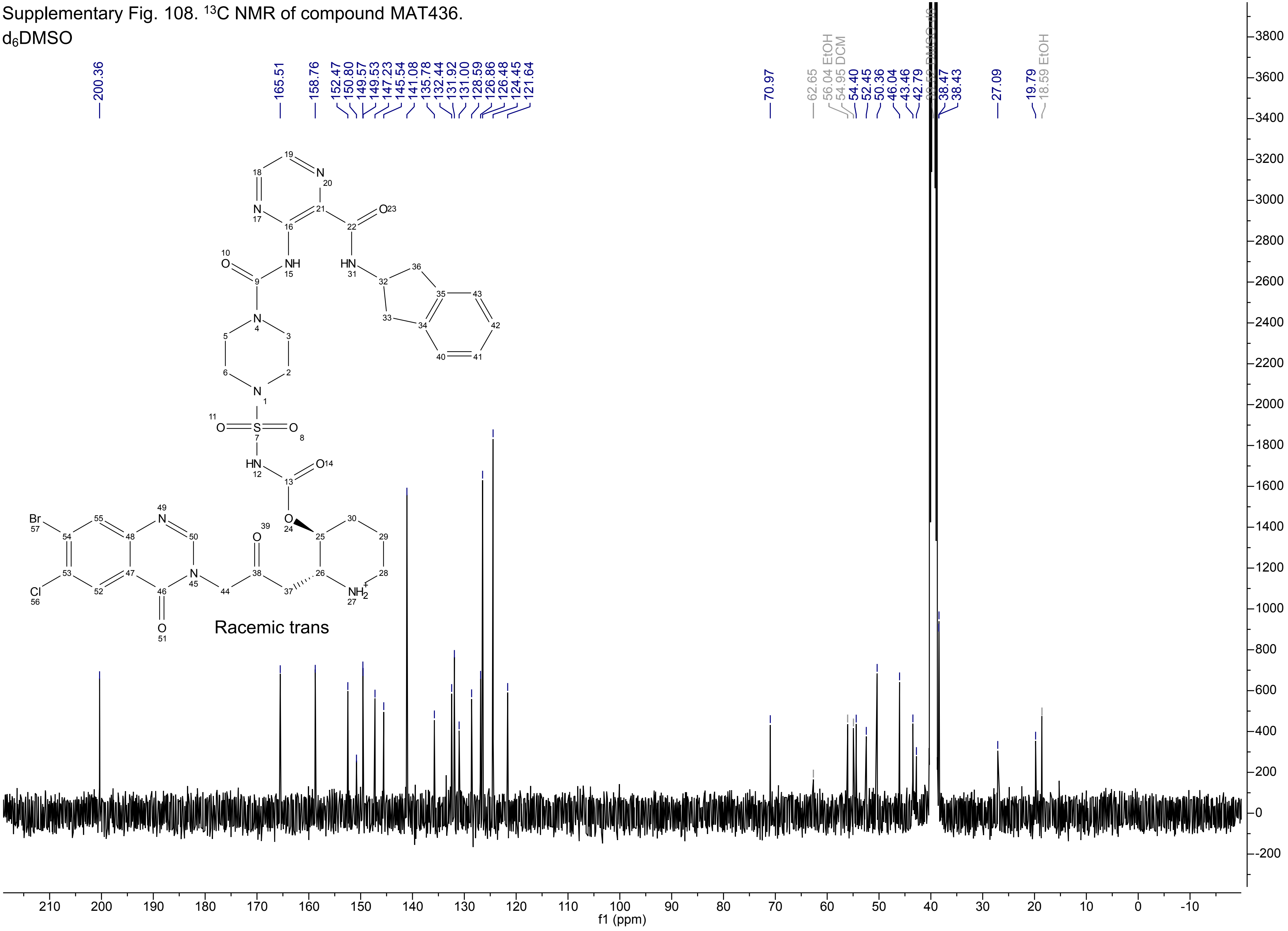

Supplementary Fig. 109. DEPT-135 NMR of compound MAT436.

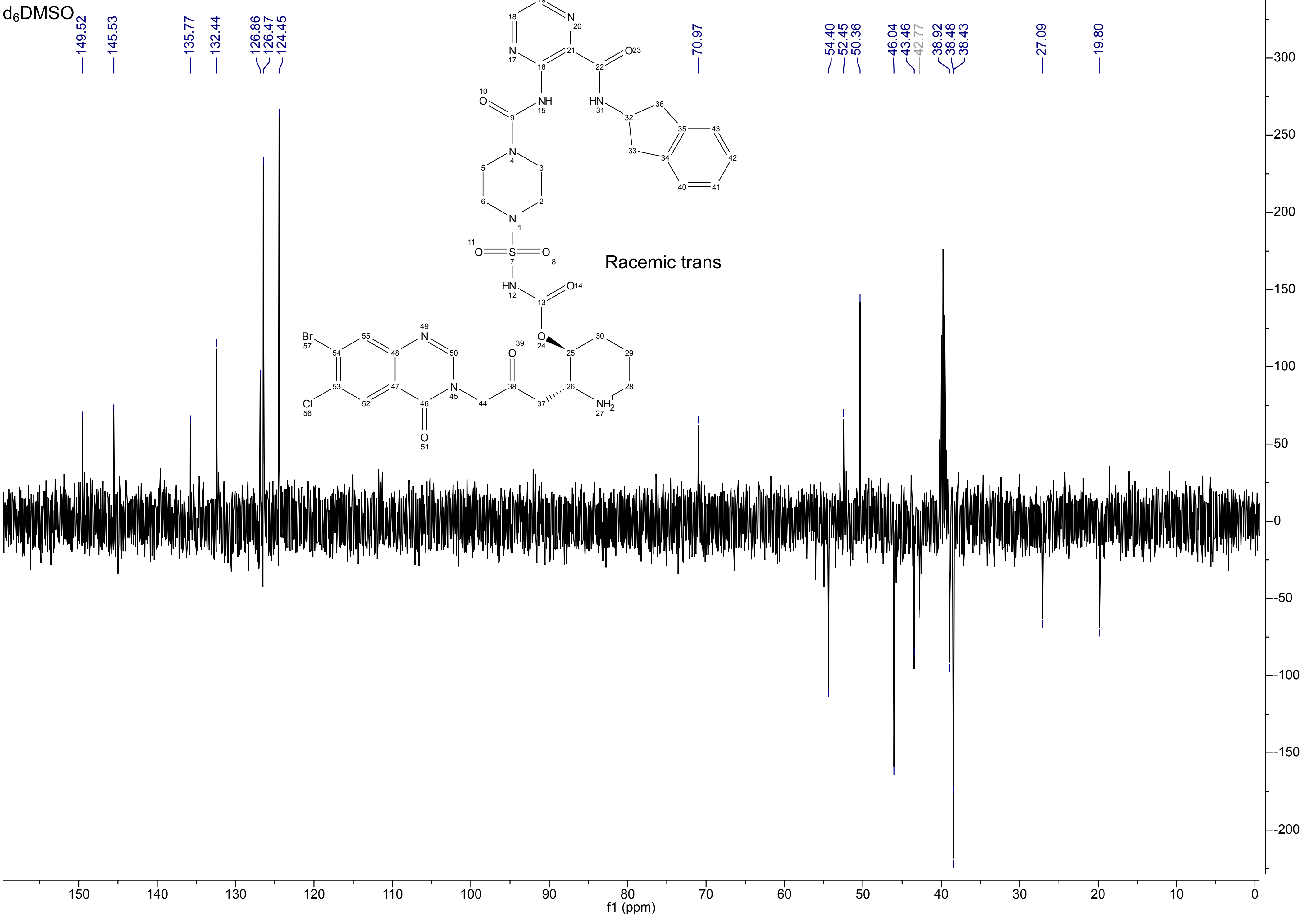

Supplementary Fig. 110. ^{1}H-^{1}H COSY NMR of compound MAT436.
d_6DMSO

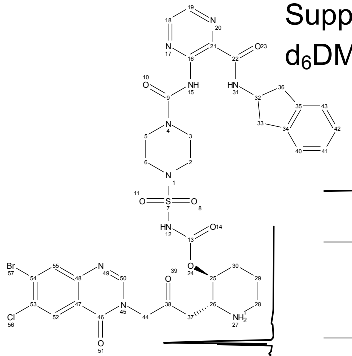

Racemic trans

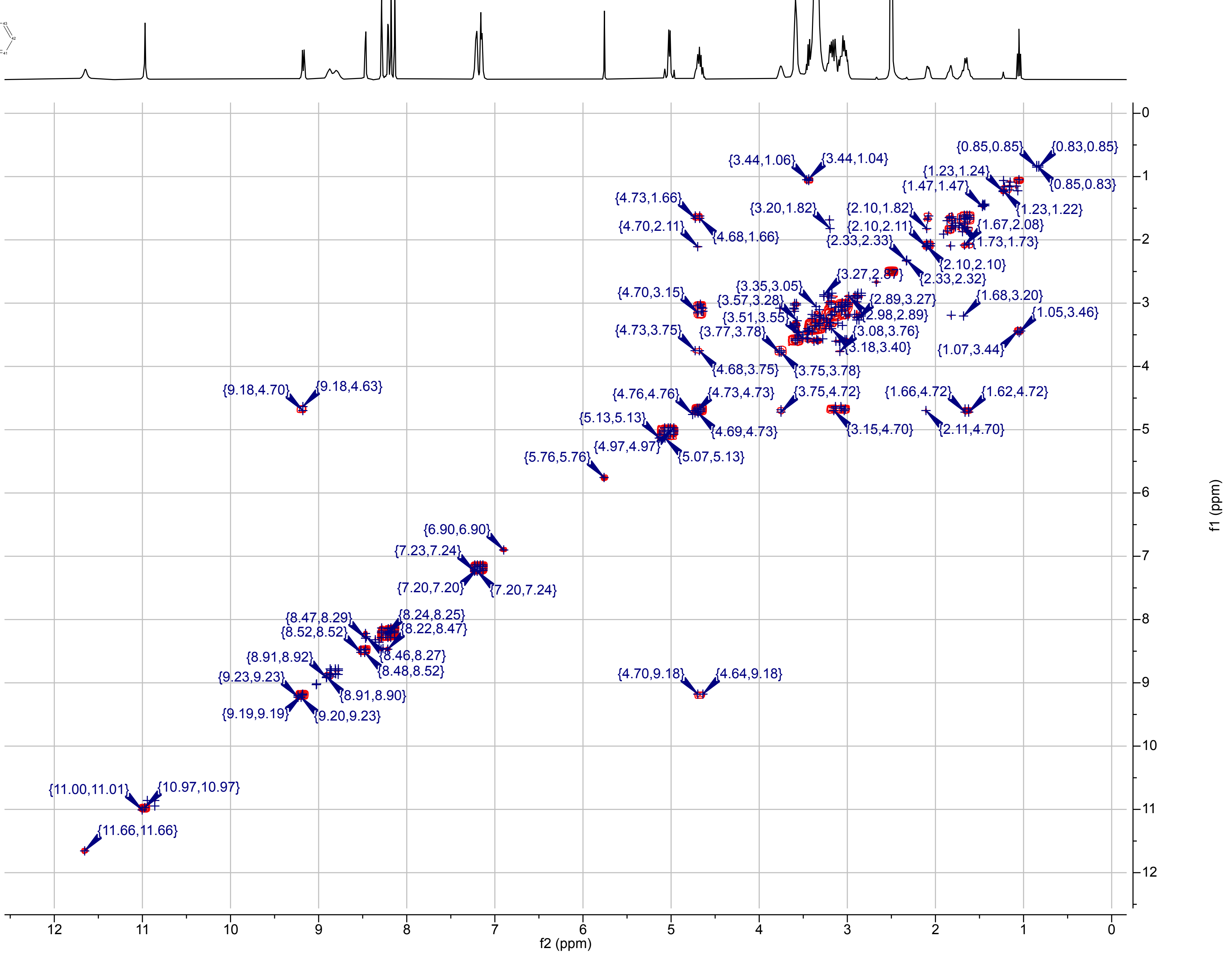

Supplementary Fig. 111. ^1H-^{13}C HMBC NMR of compound MAT436.
d_6DMSO

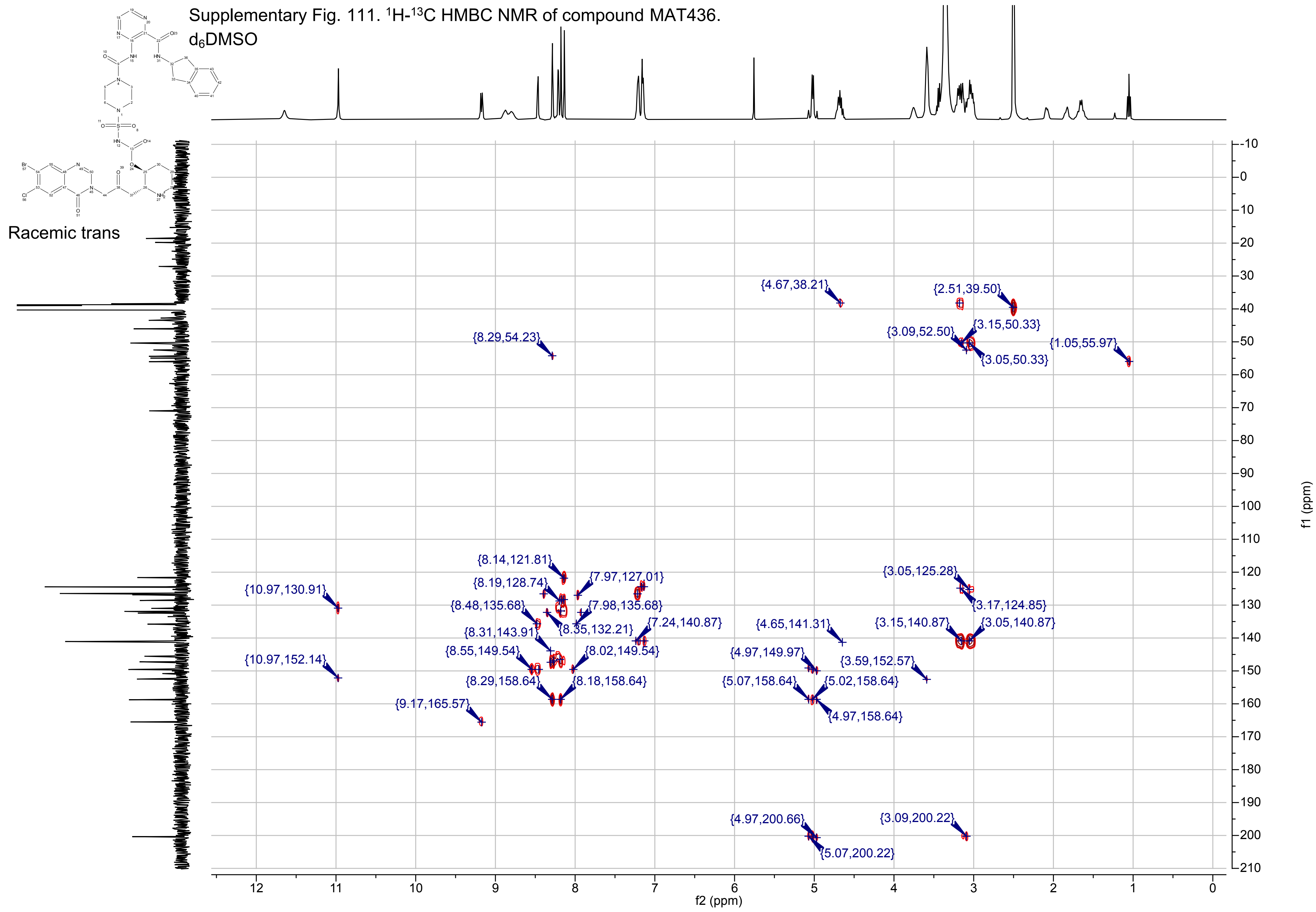

Supplementary Fig. 112. ^{1}H-^{13}C HSQC NMR of compound MAT436.
d_6DMSO

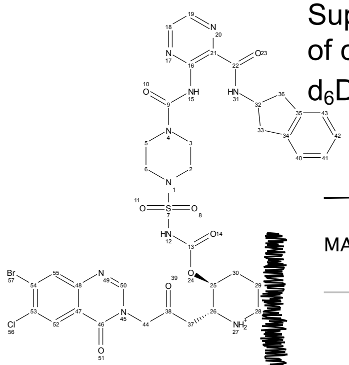

Racemic trans

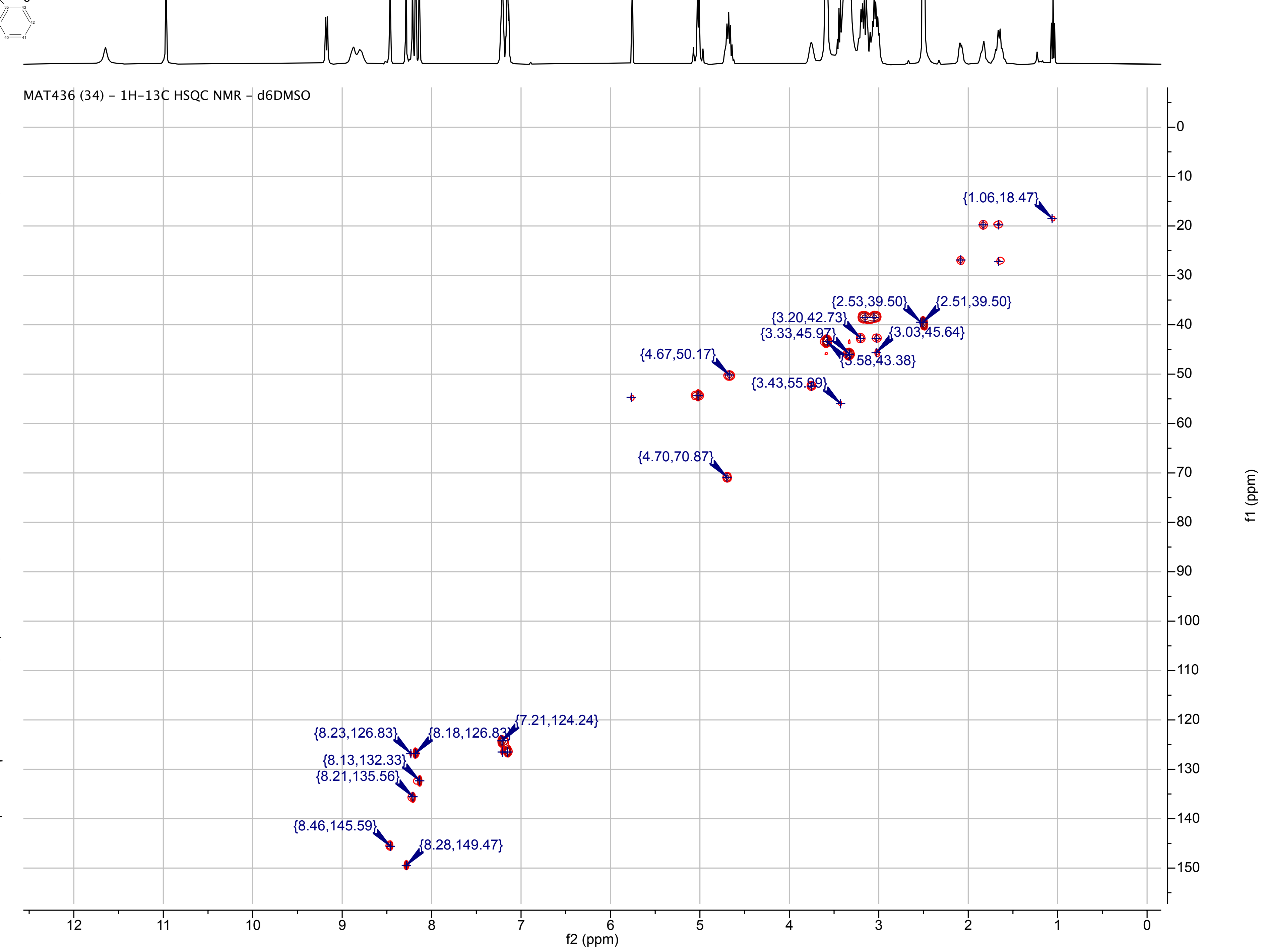

Supplementary Fig. 113. ^{1}H NMR of compound iso-MAT436.
d_6DMSO

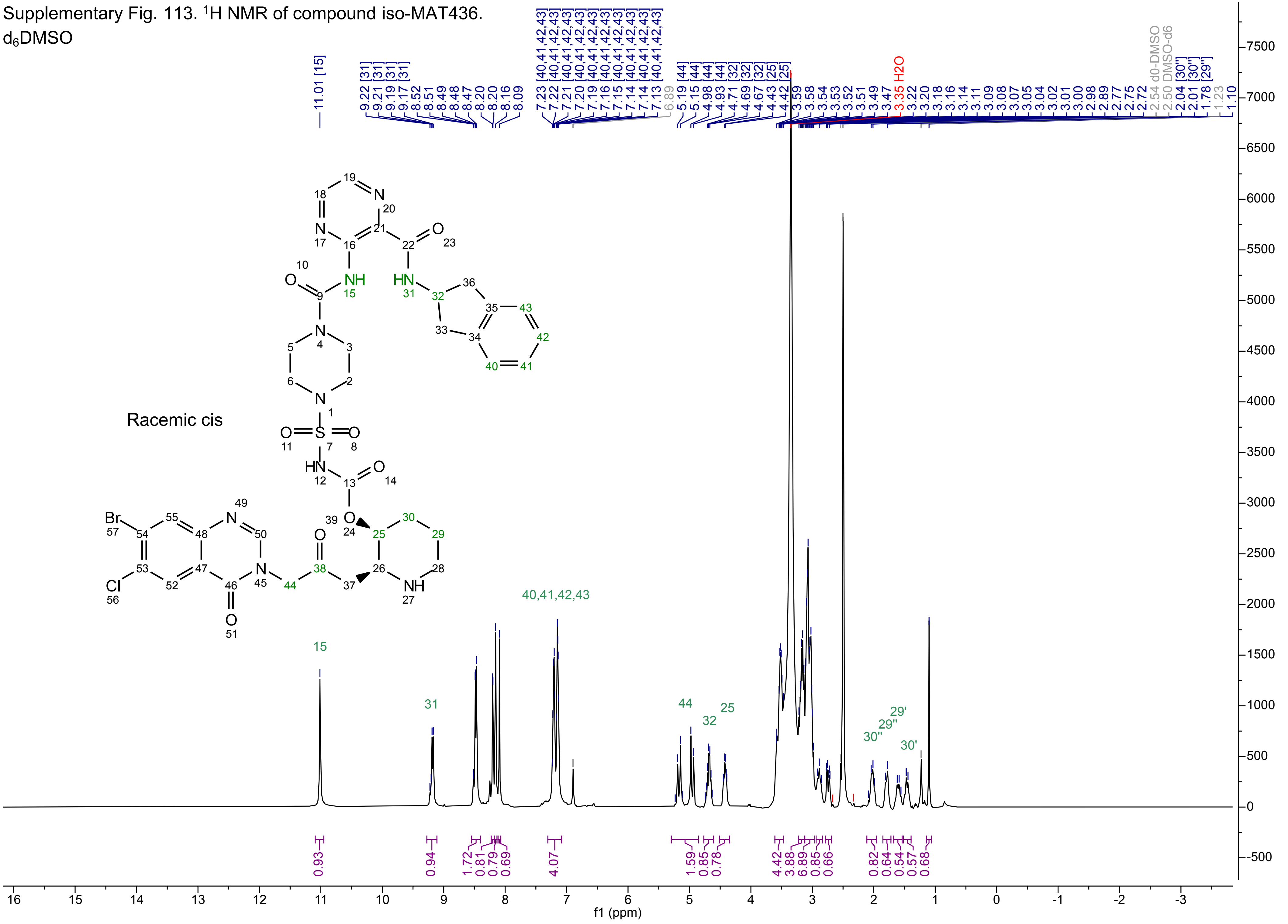

Supplementary Fig. 114. ^{13}C NMR of compound iso-MAT436.

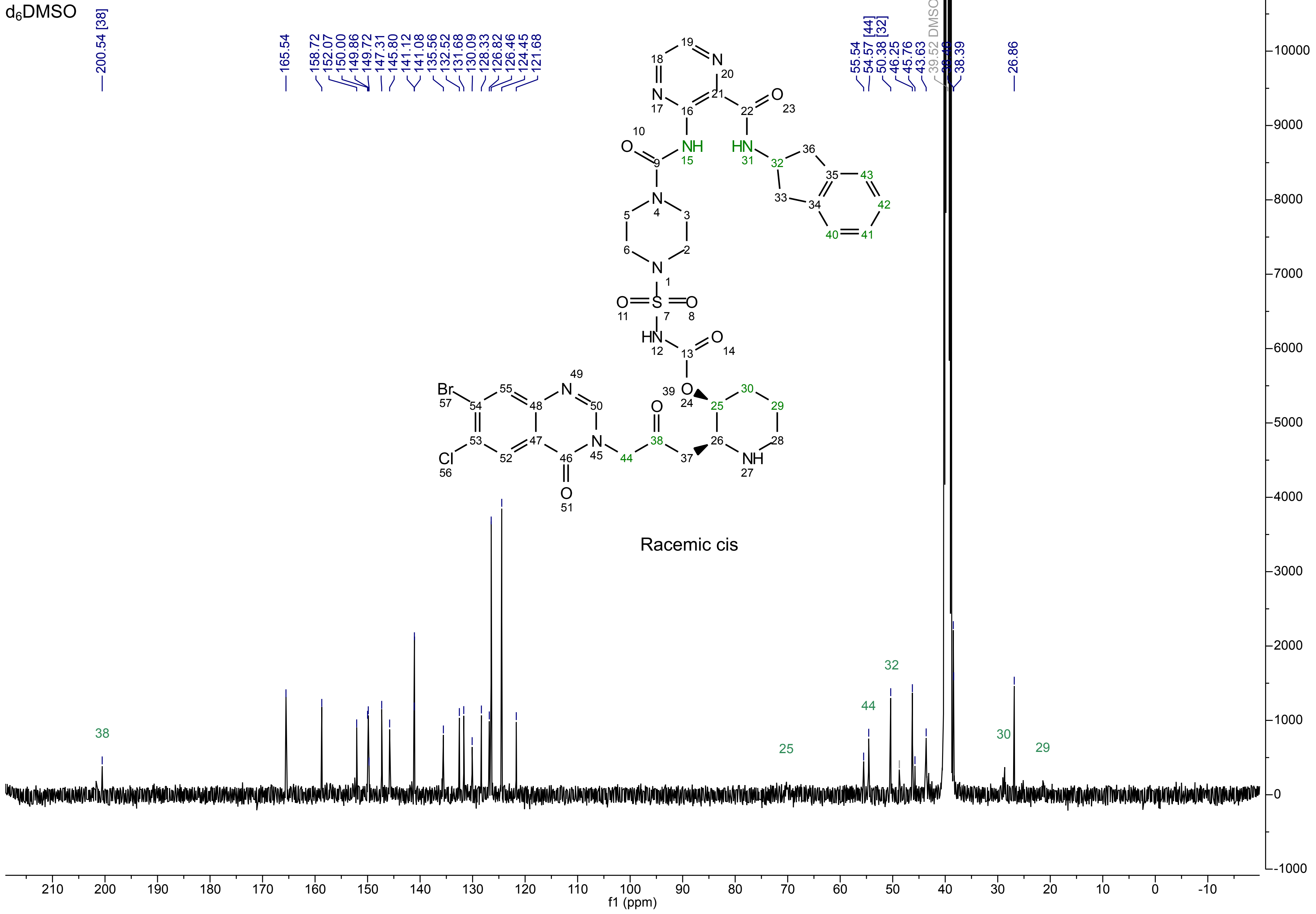

Supplementary Fig. 115. DEPT-135 NMR of compound iso-MAT436.
d_6DMSO

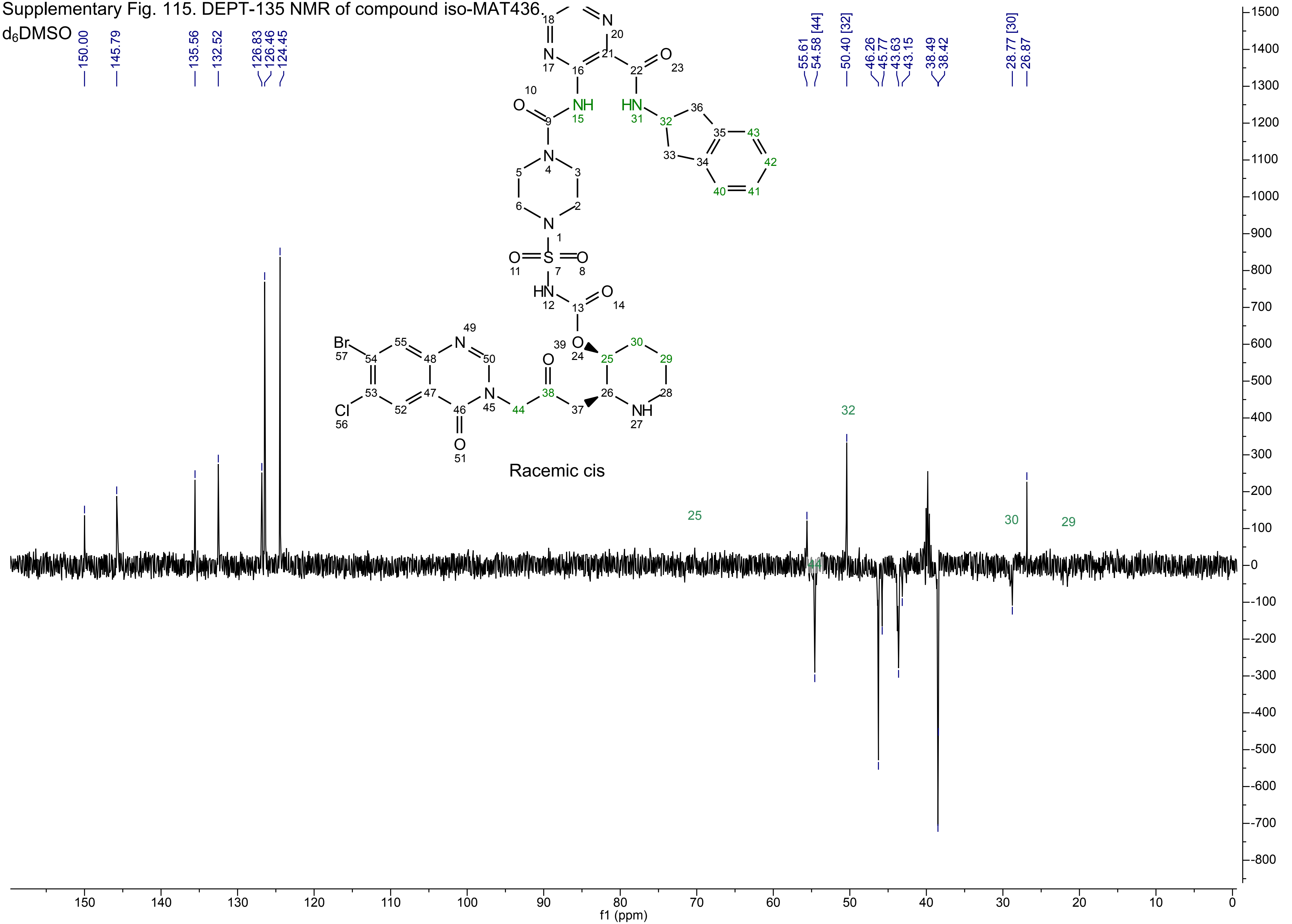

Supplementary Fig. 116. ^1H-^1H COSY NMR of compound iso-MAT436.
d_6DMSO

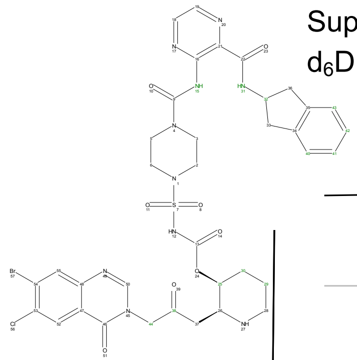

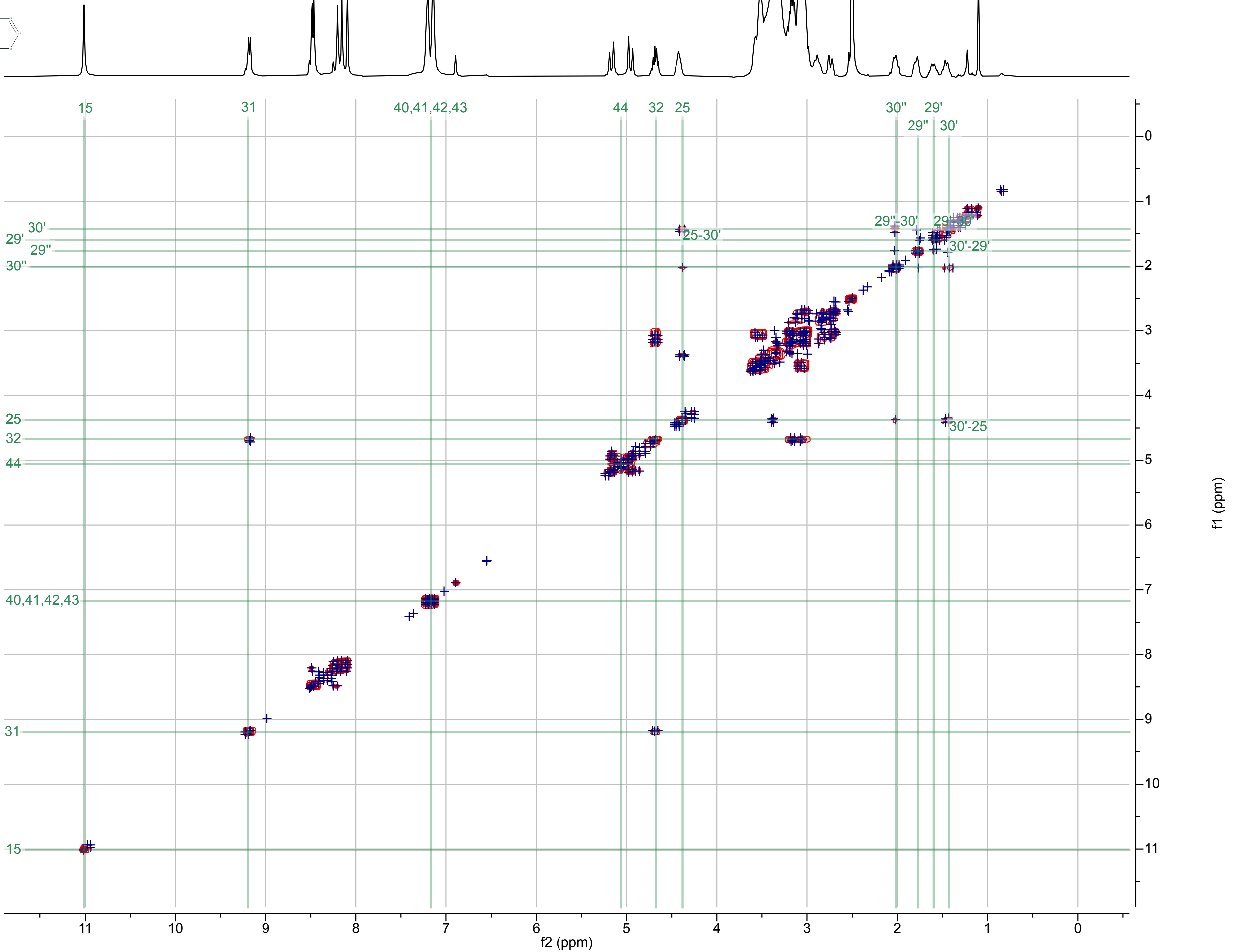

Supplementary Fig. 117. ^1H-^{13}C HMBC NMR of compound iso-MAT436.
d_6DMSO

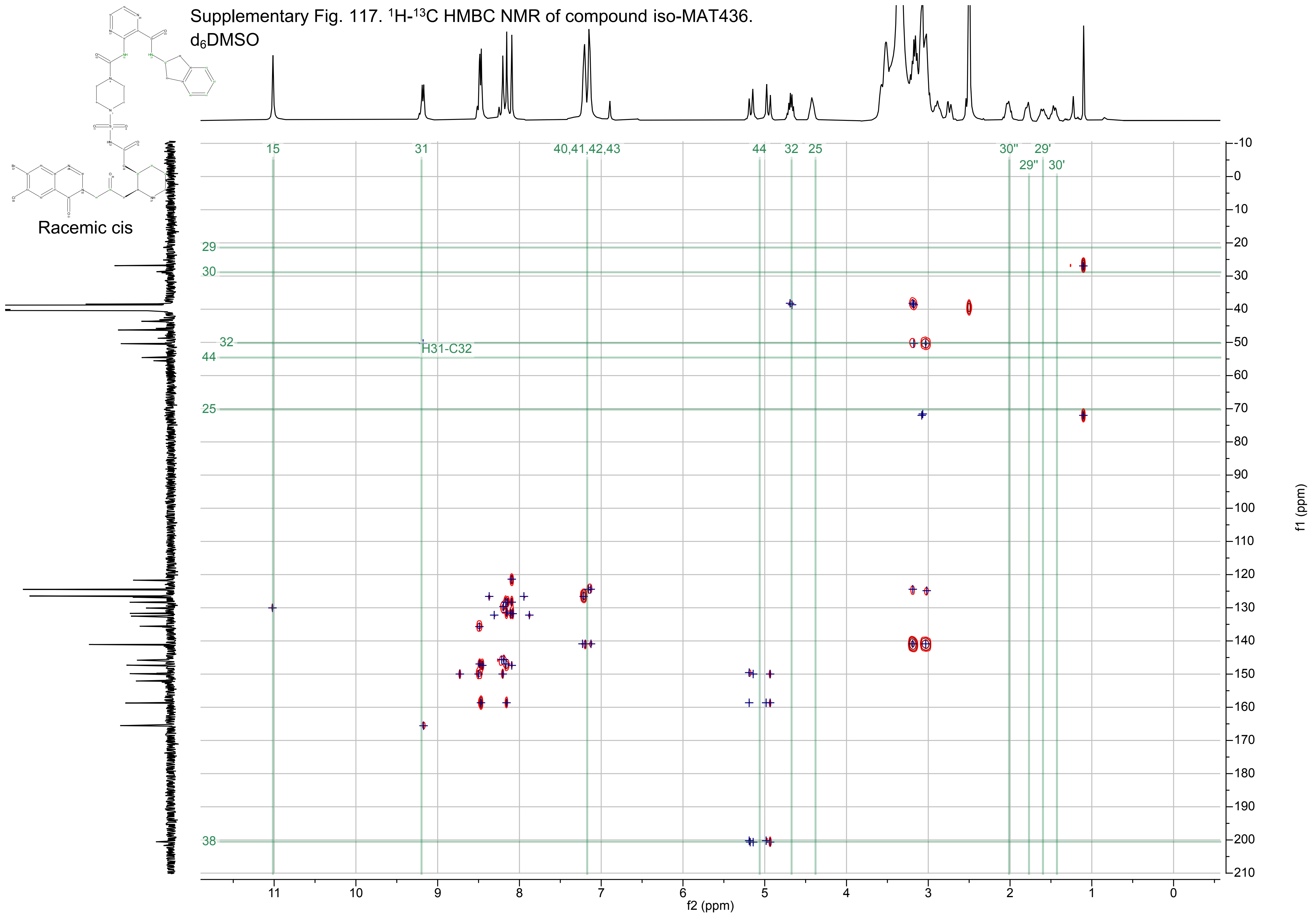

Supplementary Fig. 118. ^{1}H-^{13}C HSQC NMR of compound iso-MAT436.
d$_6$DMSO

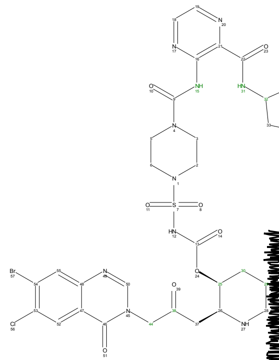

Racemic cis

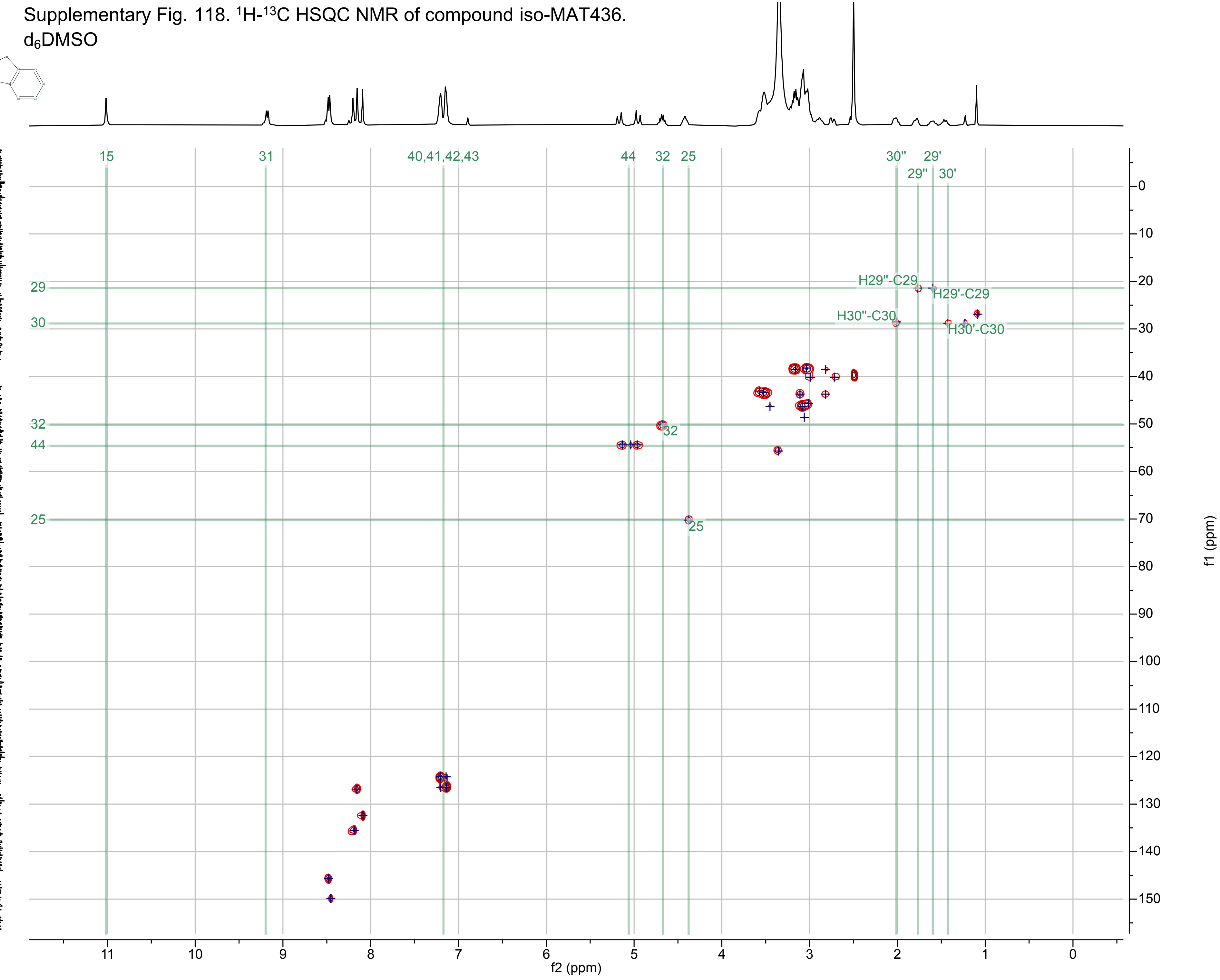

Supplementary Fig. 119. ^{1}H NMR of compound **38**.
d_6DMSO

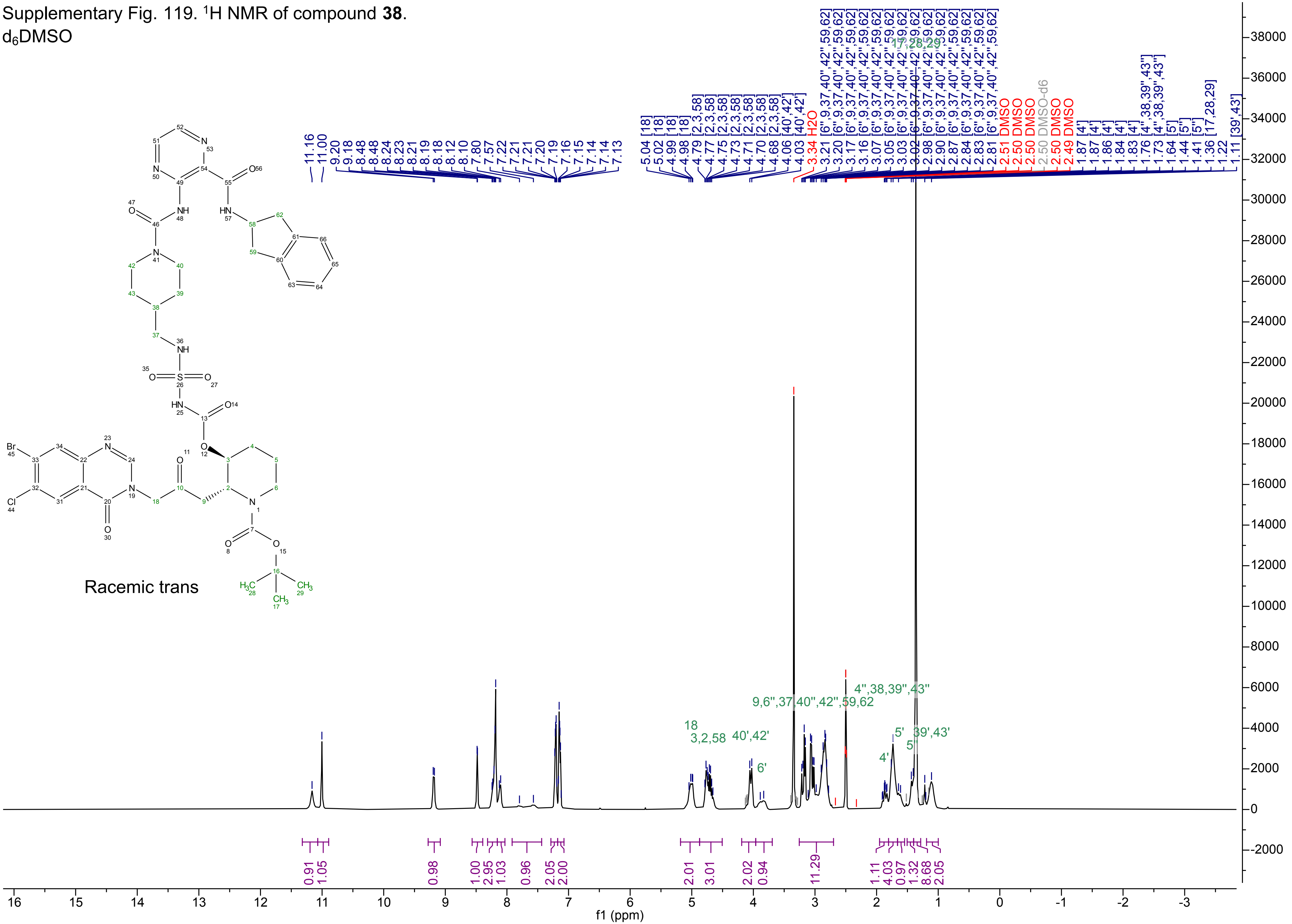

Supplementary Fig. 120. ^{13}C NMR of compound **38**.
d_6DMSO

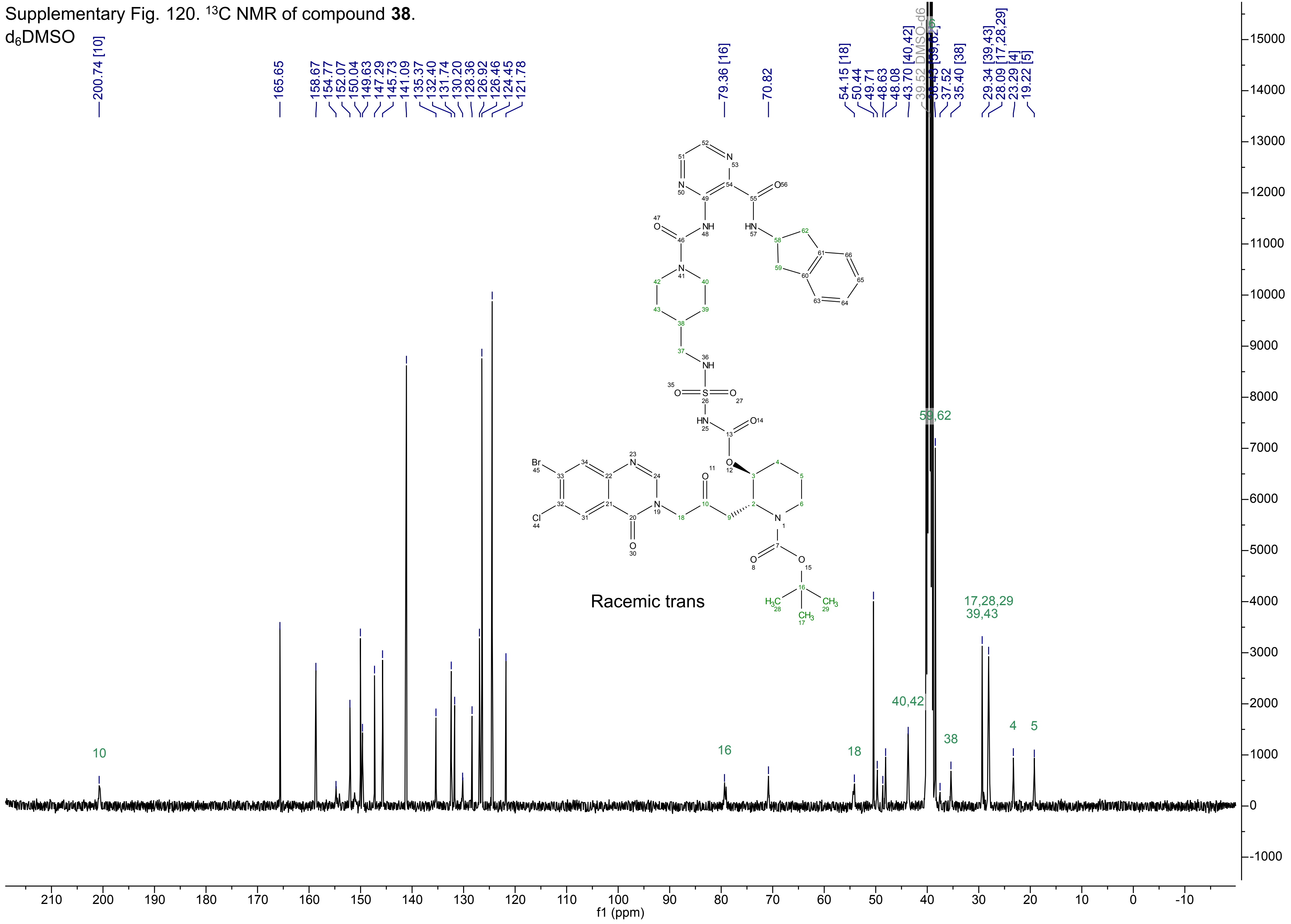

Supplementary Fig. 121. DEPT-135 NMR of compound **38**.
d_6DMSO

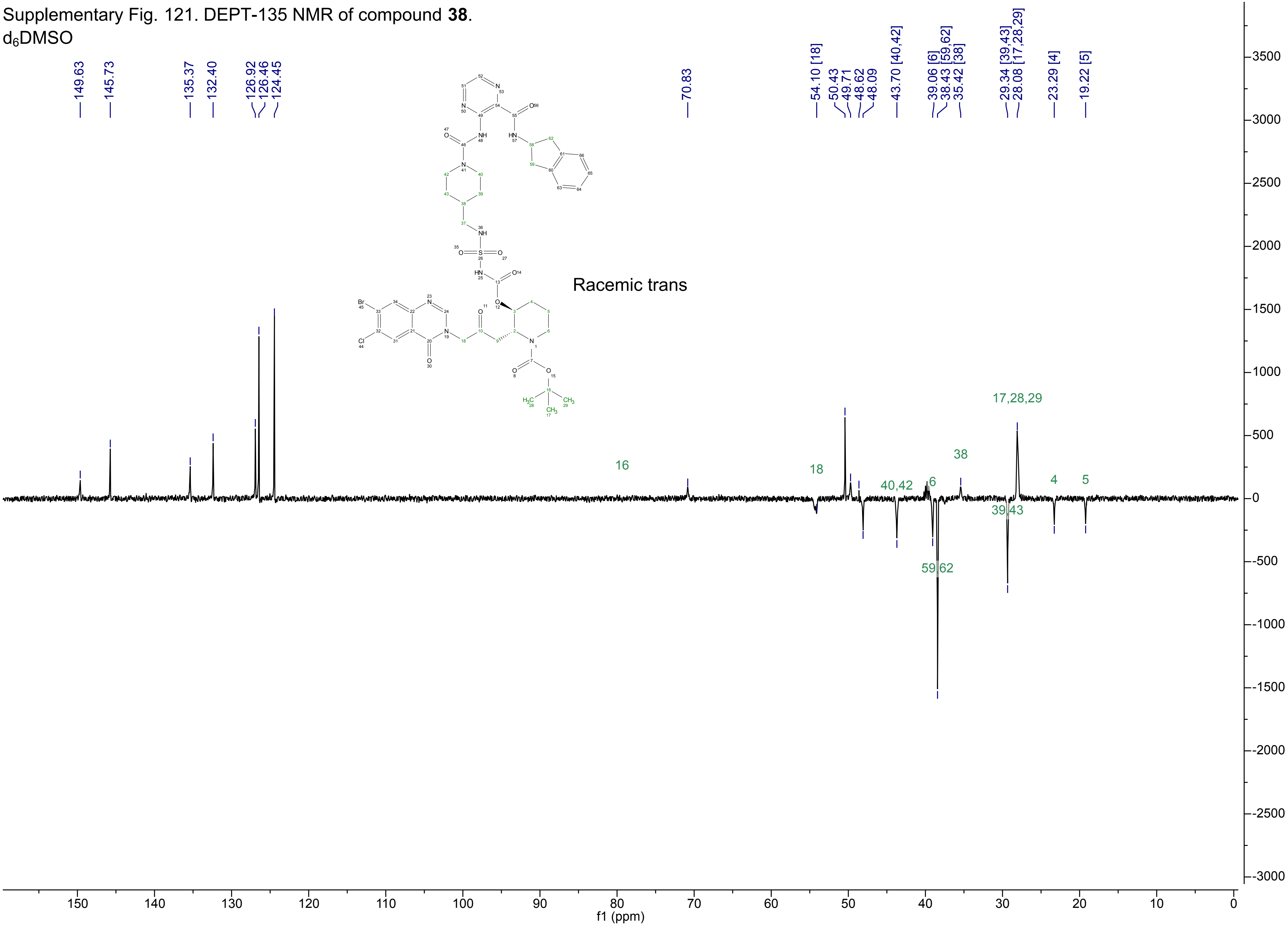

Supplementary Fig. 122. ^1H-^1H COSY NMR of compound **38**.
d_6DMSO

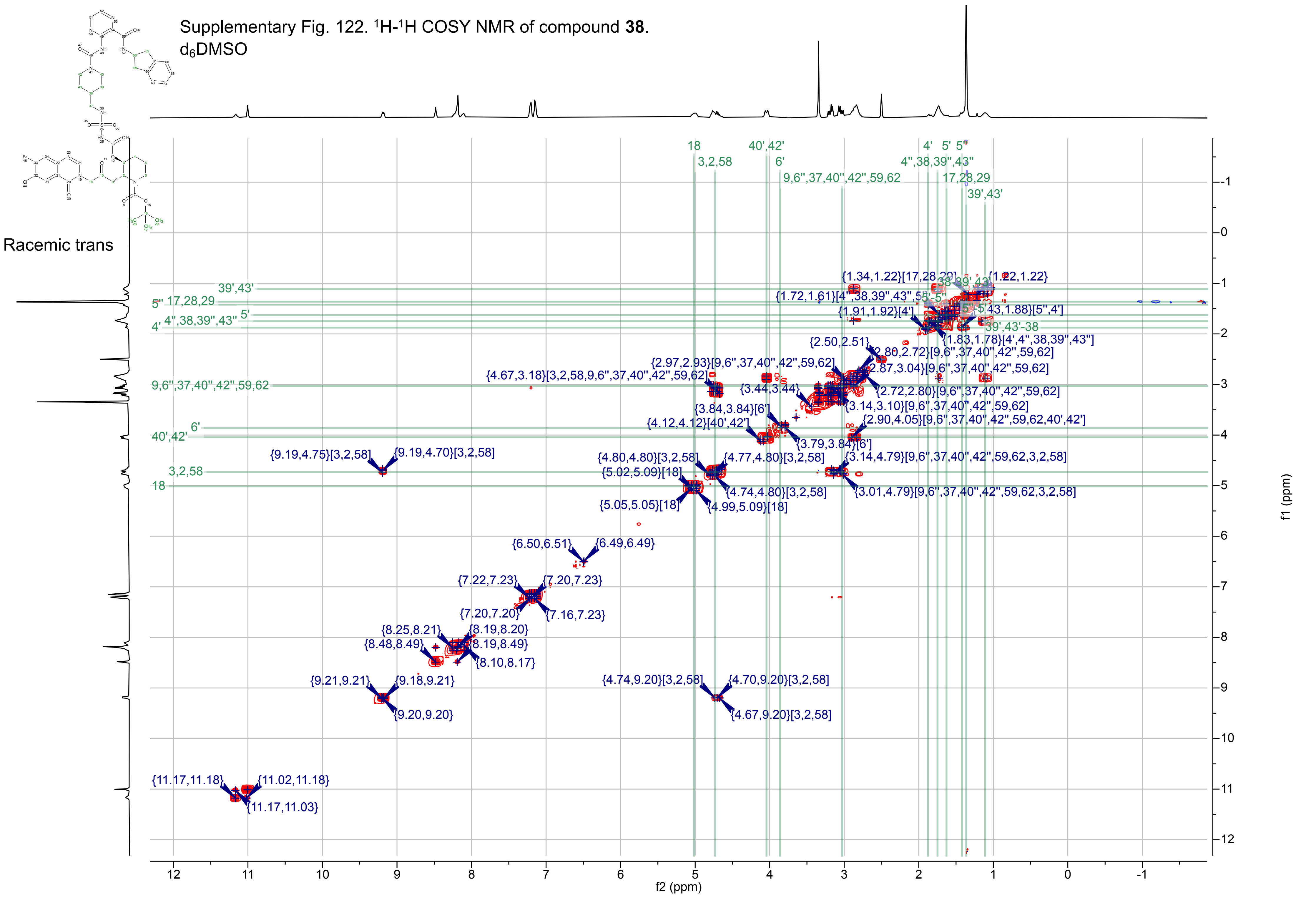

Supplementary Fig. 123. ^{1}H-^{13}C HMBC NMR of compound **38**.
d$_6$DMSO

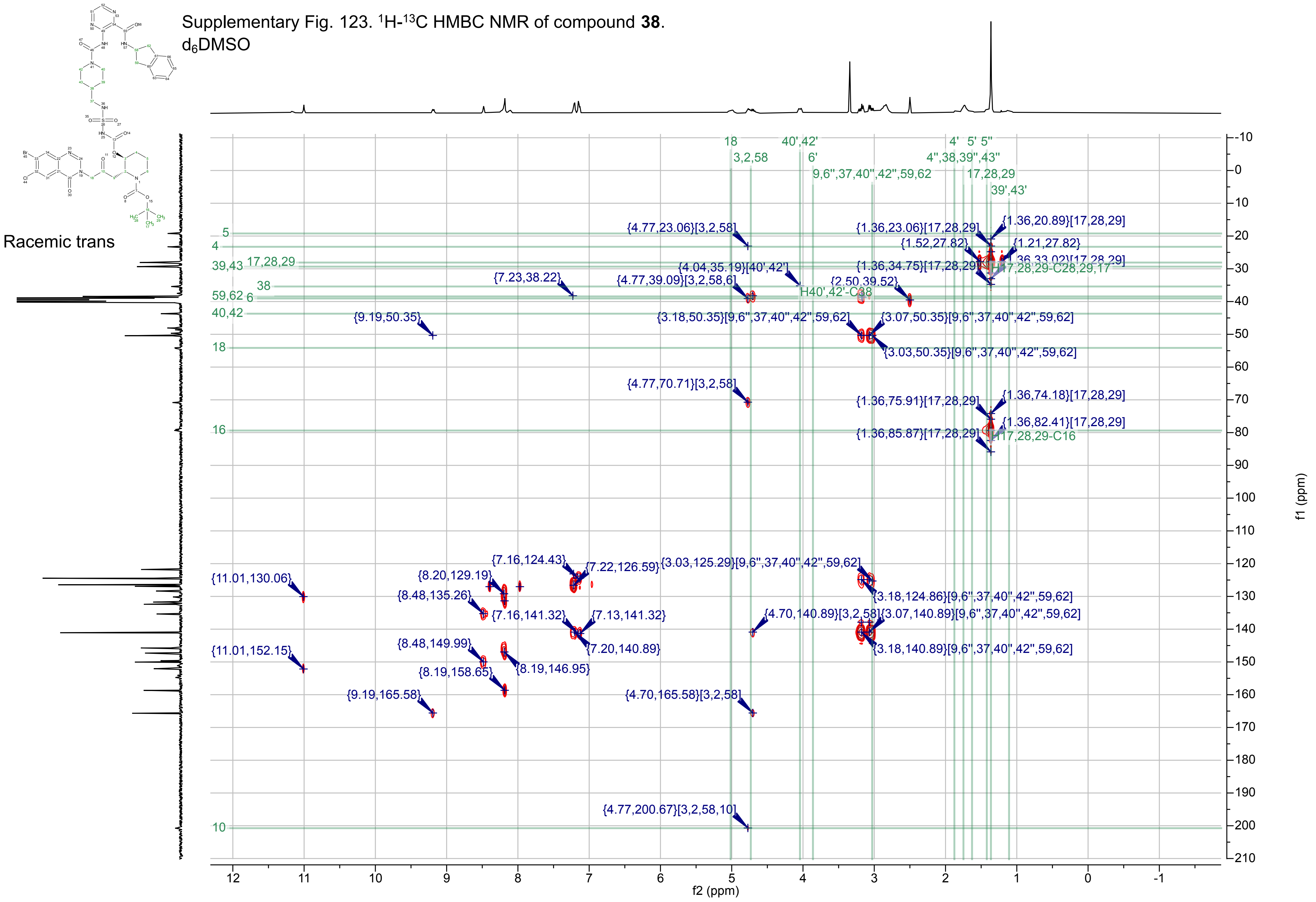

Supplementary Fig. 124. ^{1}H-^{13}C HSQC NMR of compound **38**.
d$_6$DMSO

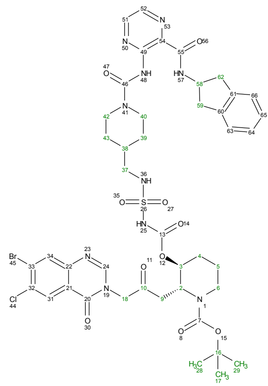

Racemic trans

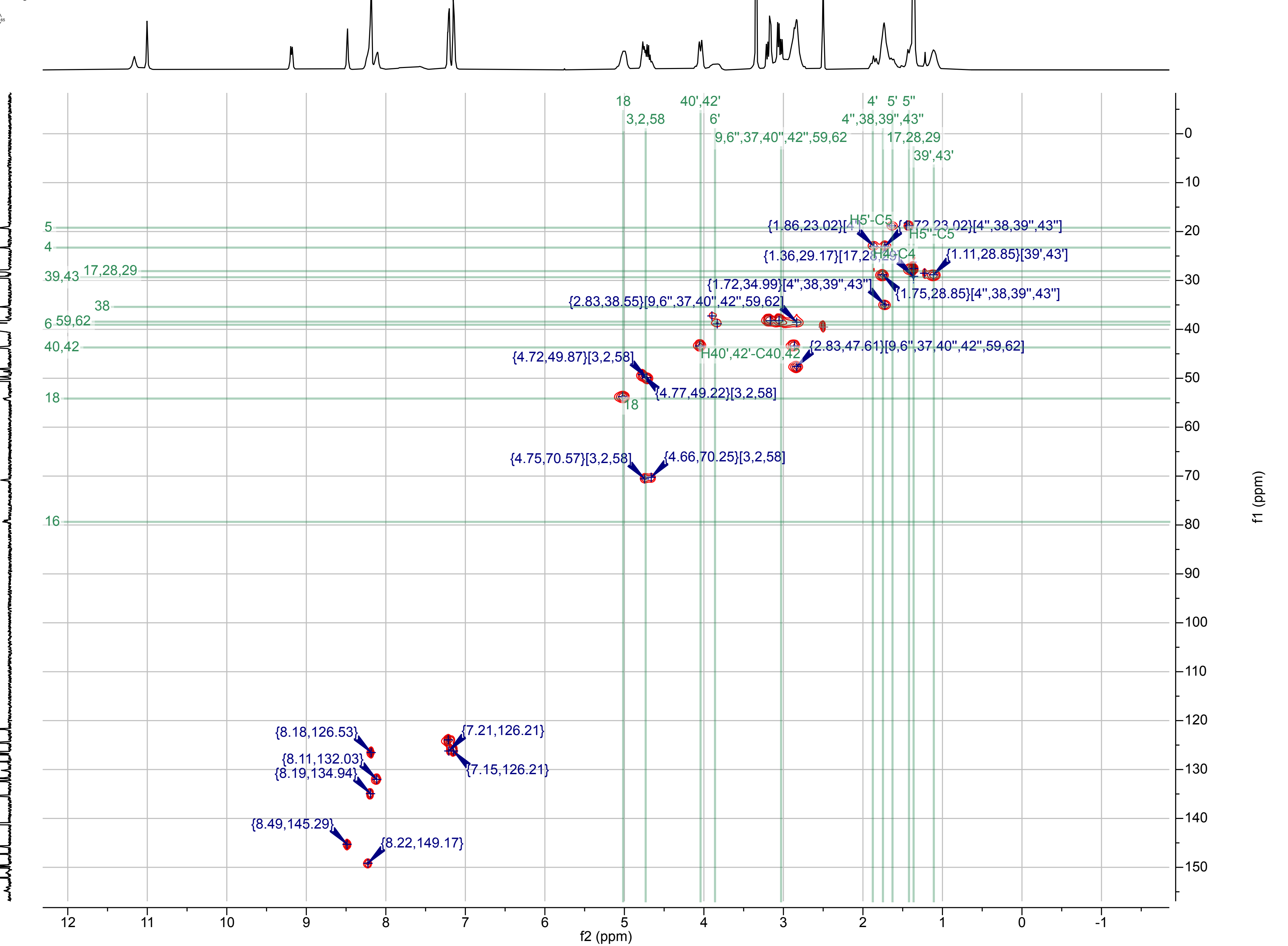

Supplementary Fig. 125. ^{1}H NMR of compound **35**.

d_6DMSO

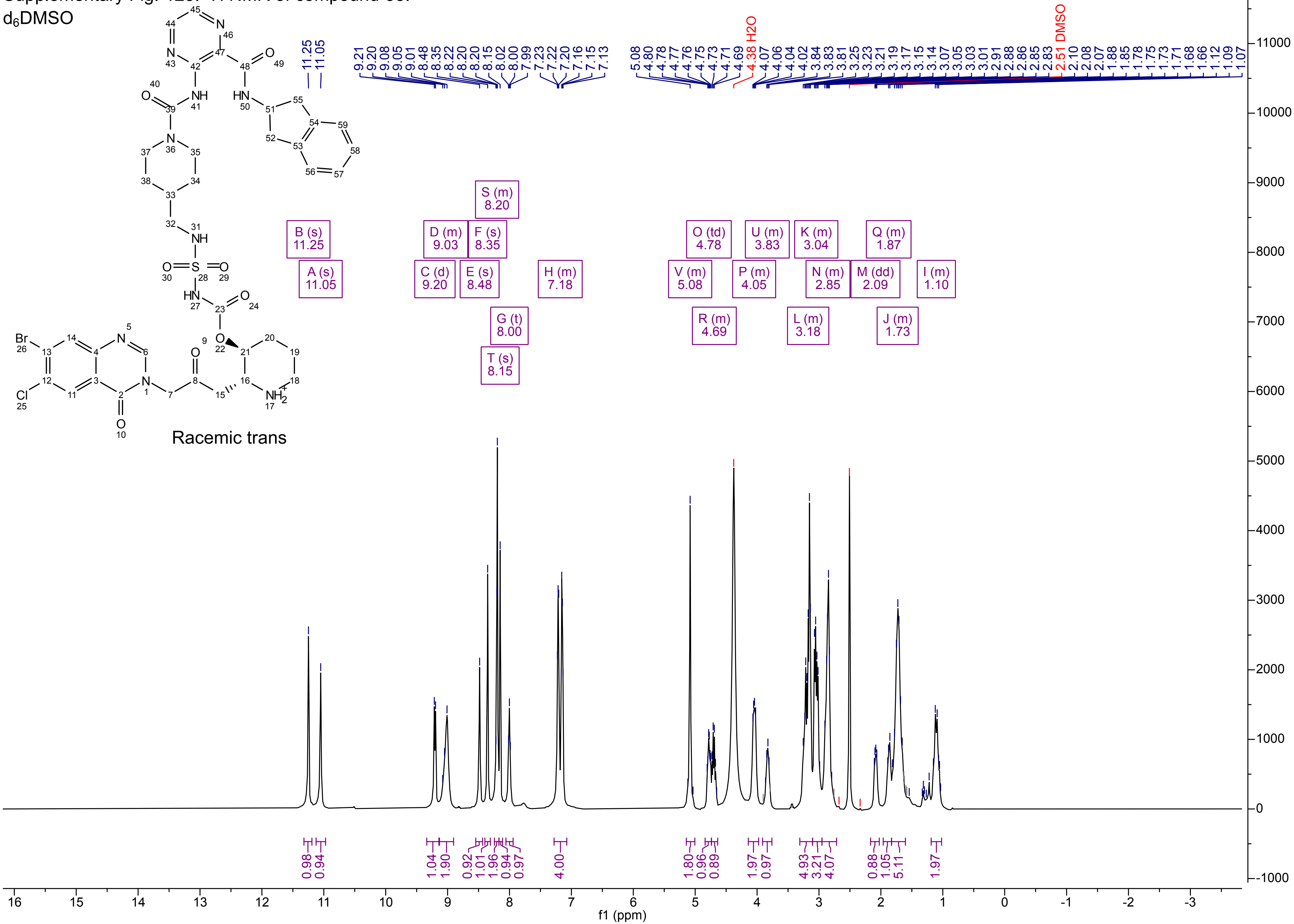

Supplementary Fig. 126. ^{13}C NMR of compound **35**.
d_6DMSO

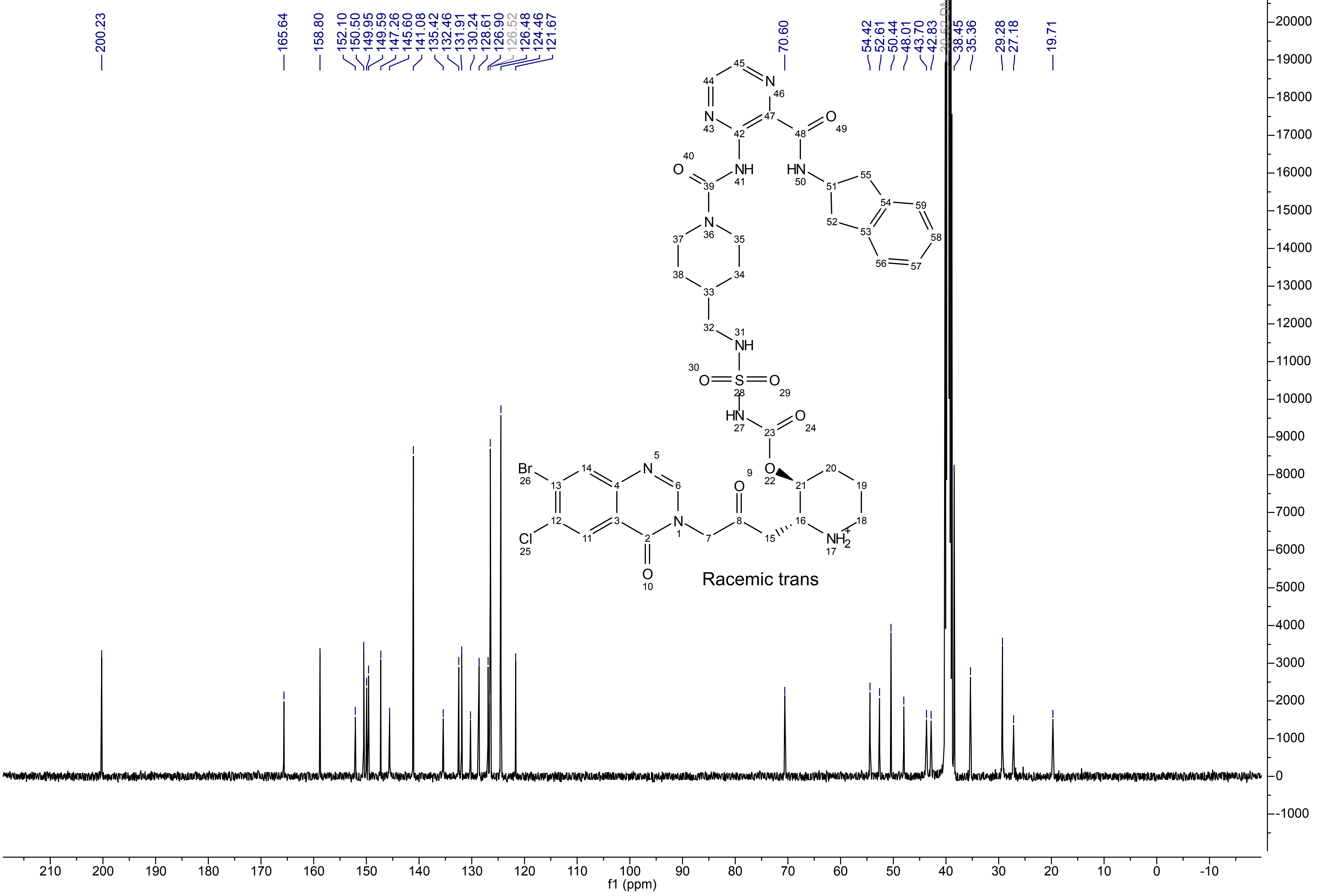

Supplementary Fig. 127. DEPT-135 NMR of compound **35**.

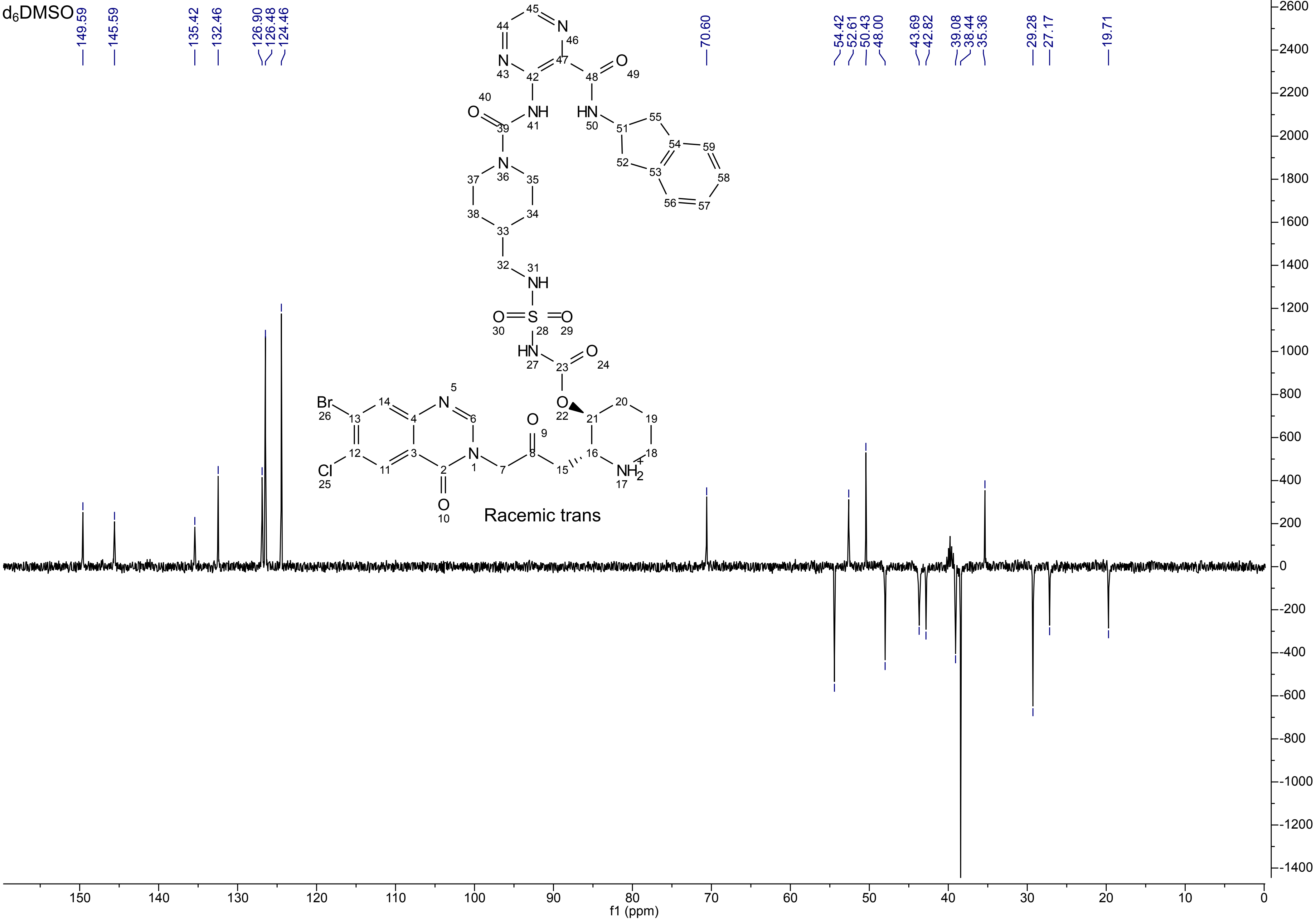

Supplementary Fig. 128. ^1H-^1H COSY NMR of compound **35**.
d_6DMSO

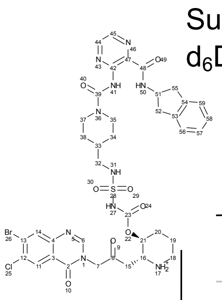

Racemic trans

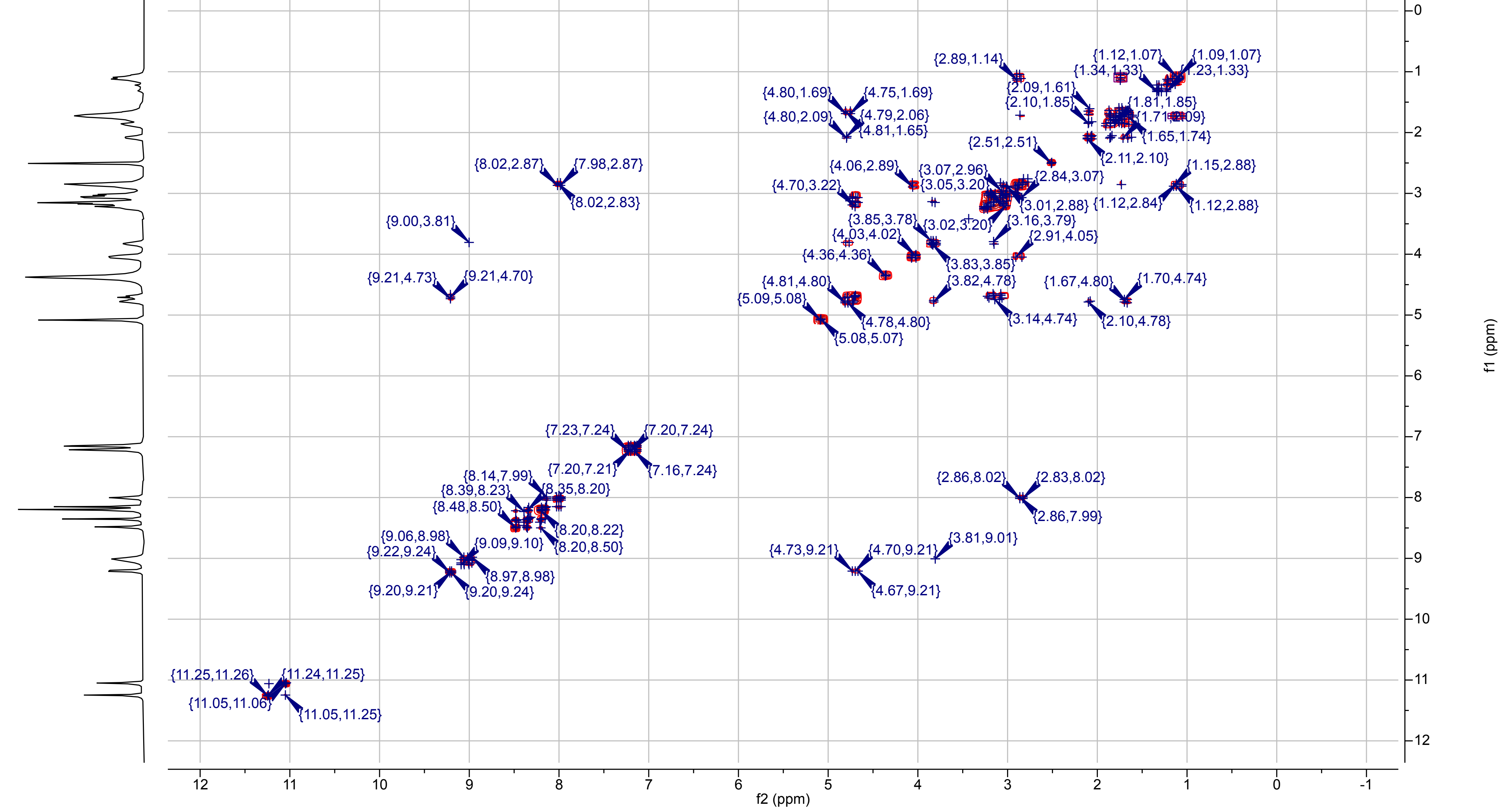

Supplementary Fig. 129. ^{1}H-^{13}C HMBC NMR of compound **35**.
d$_6$DMSO

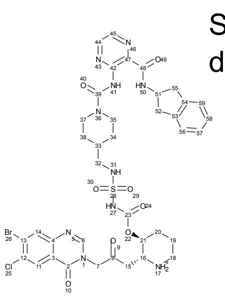

Racemic trans

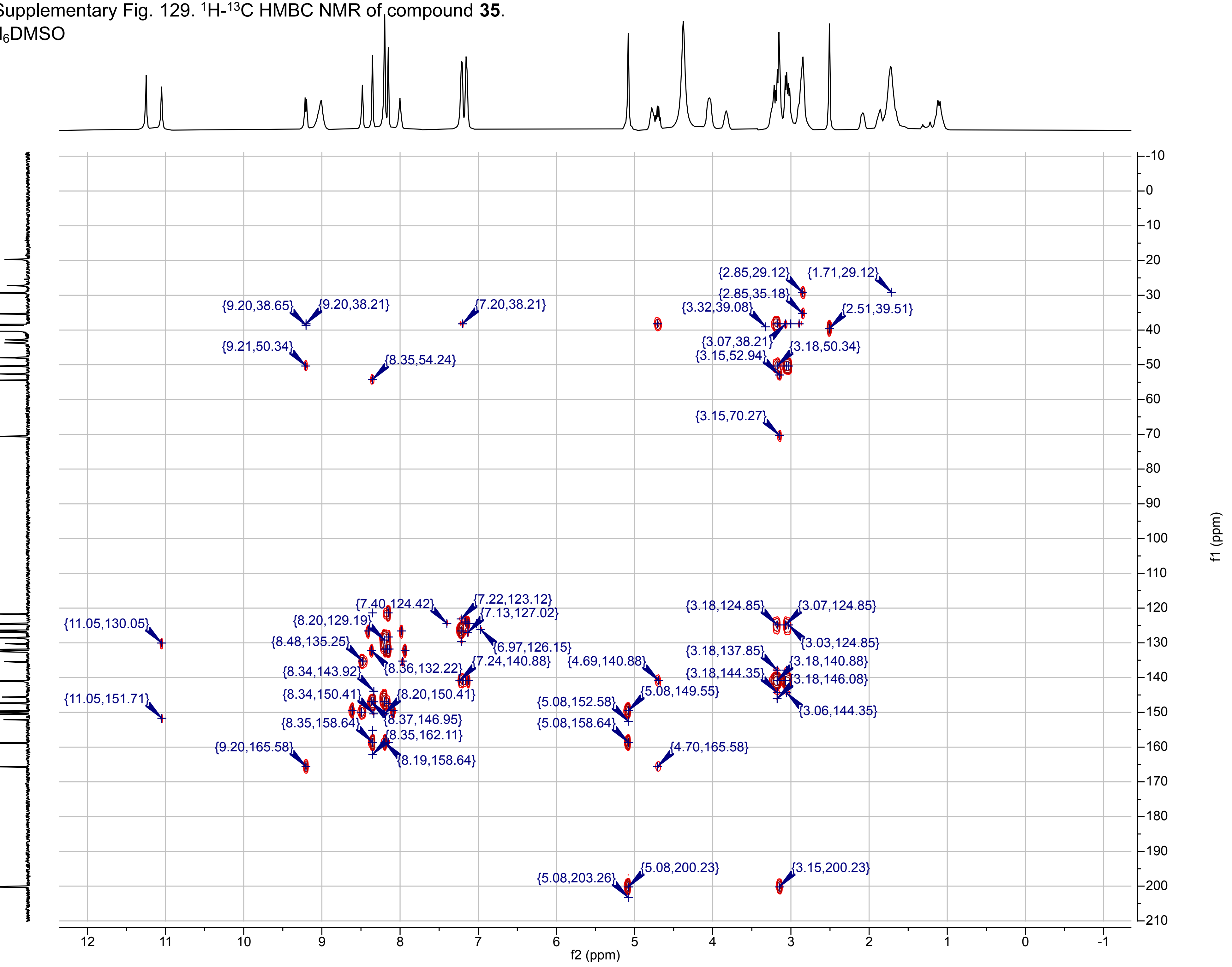

Supplementary Fig. 130. ^{1}H-^{13}C HSQC NMR of compound **35**.
d_6DMSO

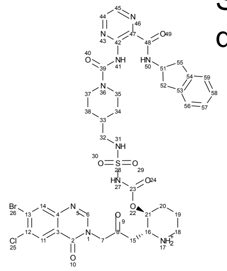

Racemic trans

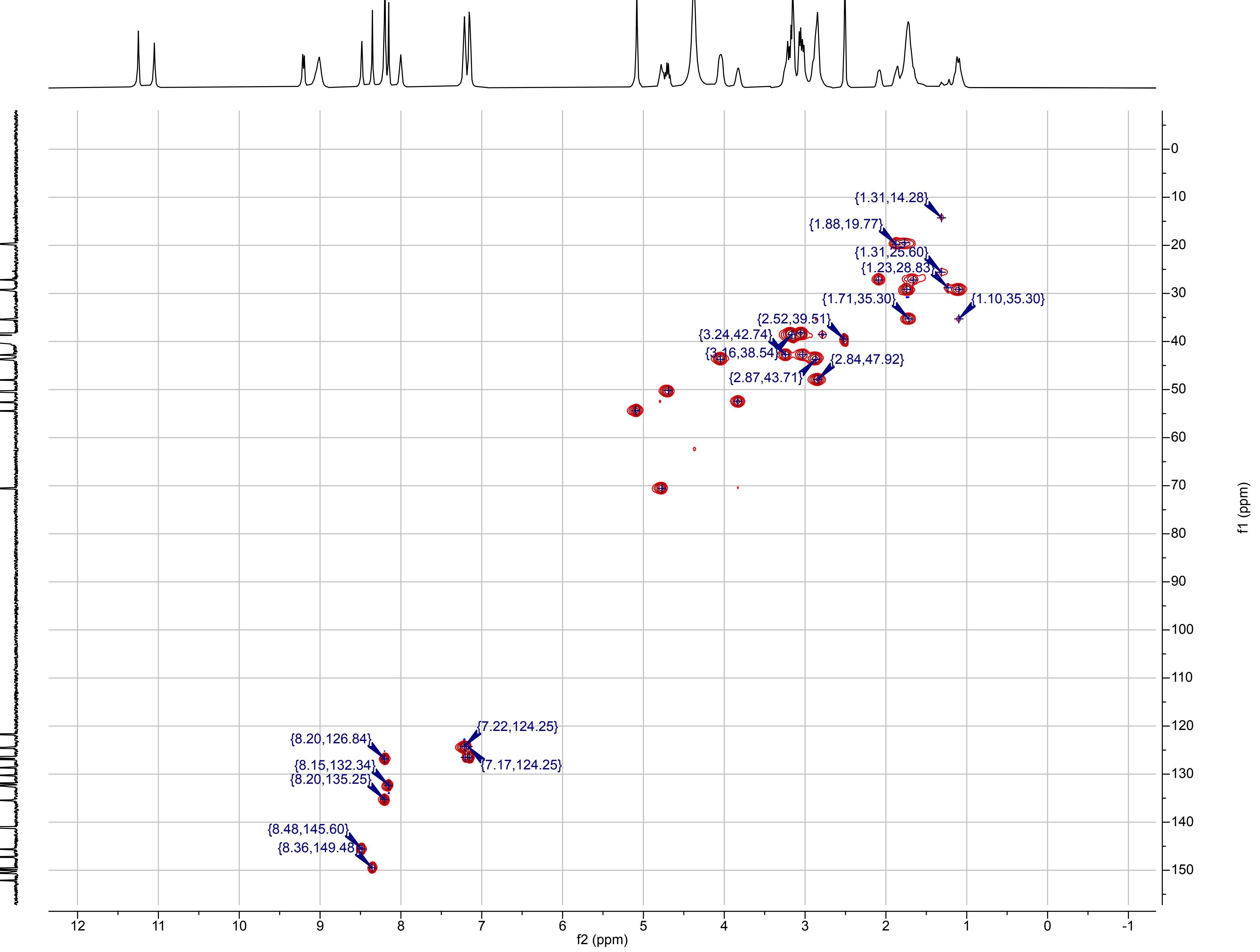

Supplementary Fig. 131. ^{1}H NMR of compound **39**.

d$_6$DMSO

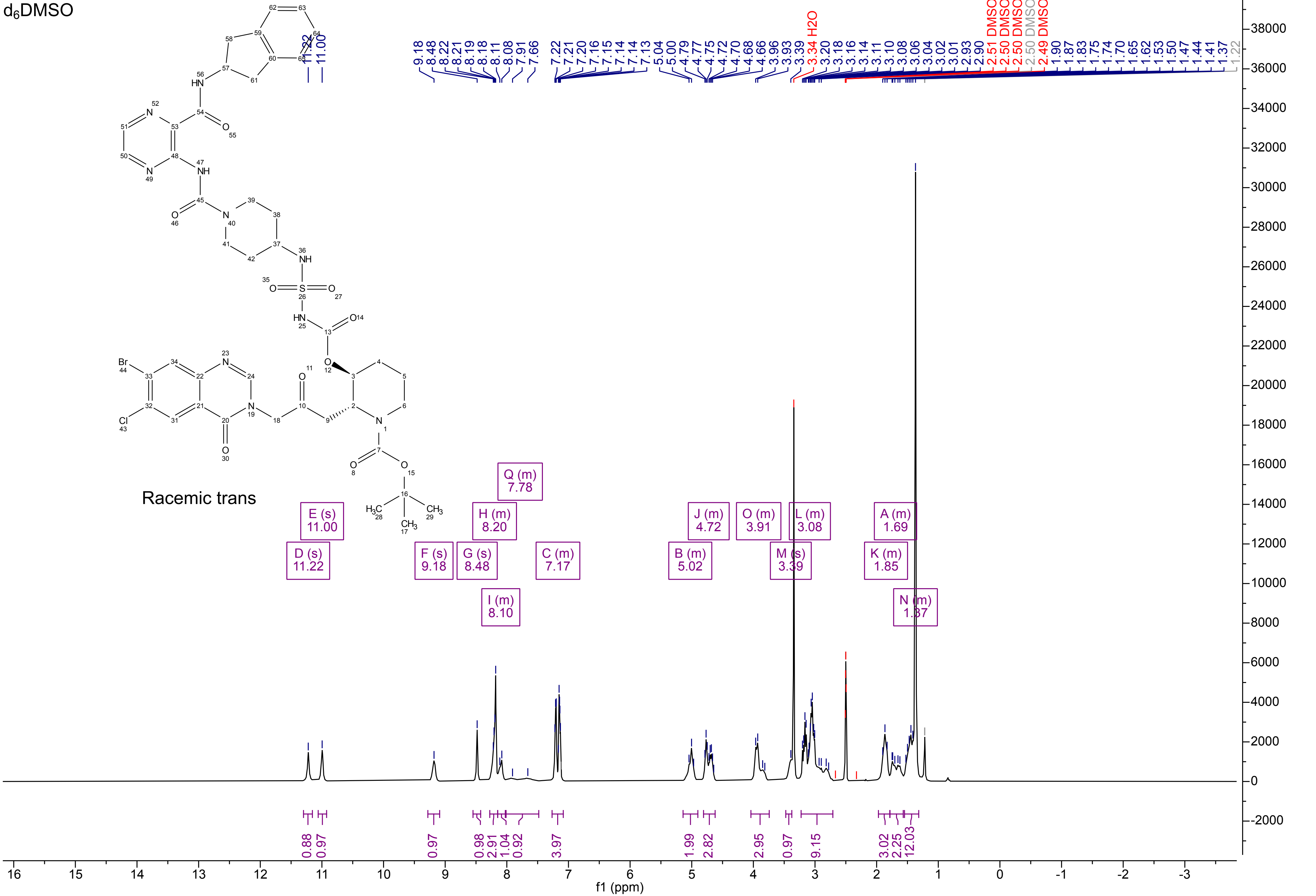

Supplementary Fig. 132. ^{13}C NMR of compound **39**.
d_6DMSO

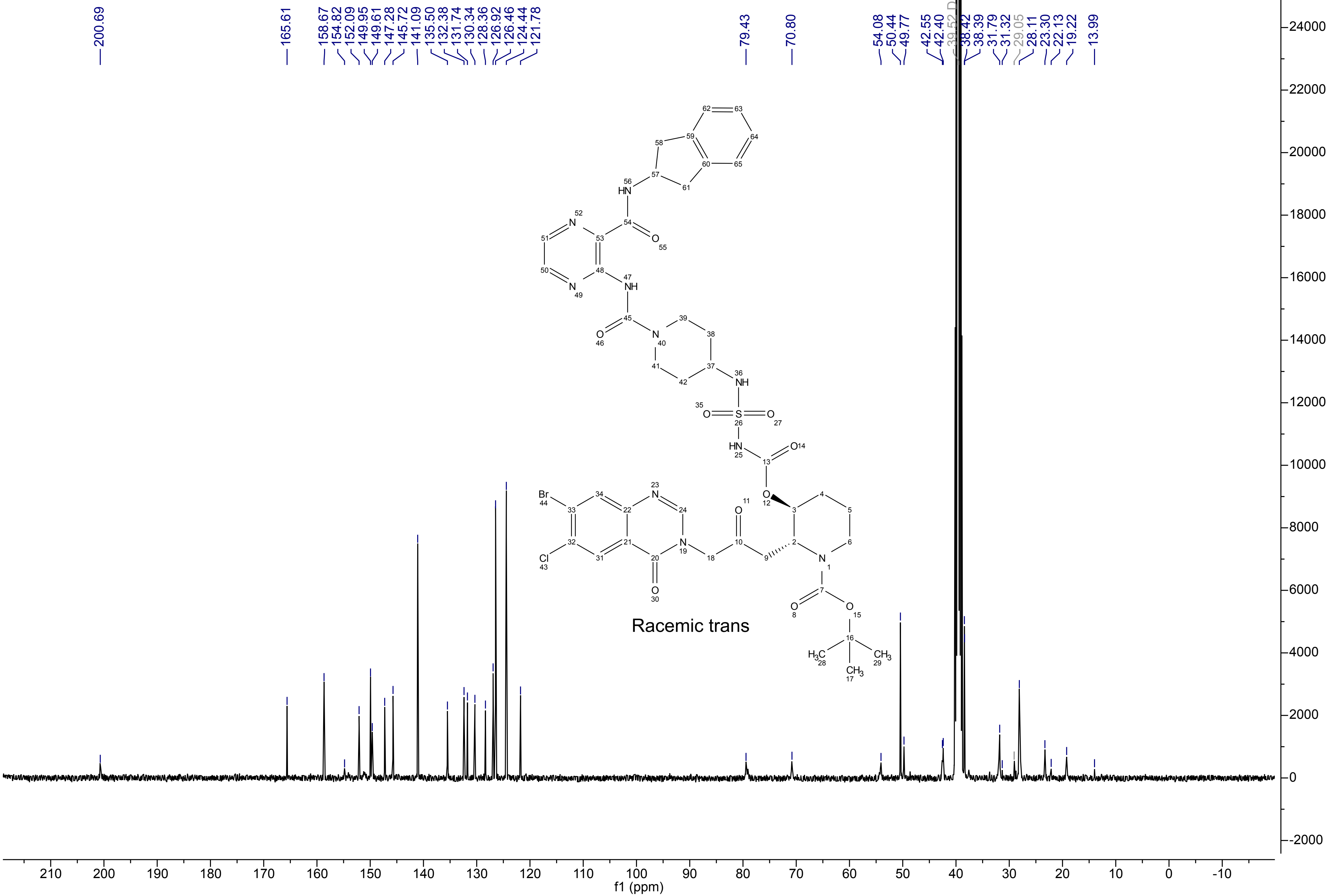

Supplementary Fig. 133. DEPT-135 NMR of compound **39**.

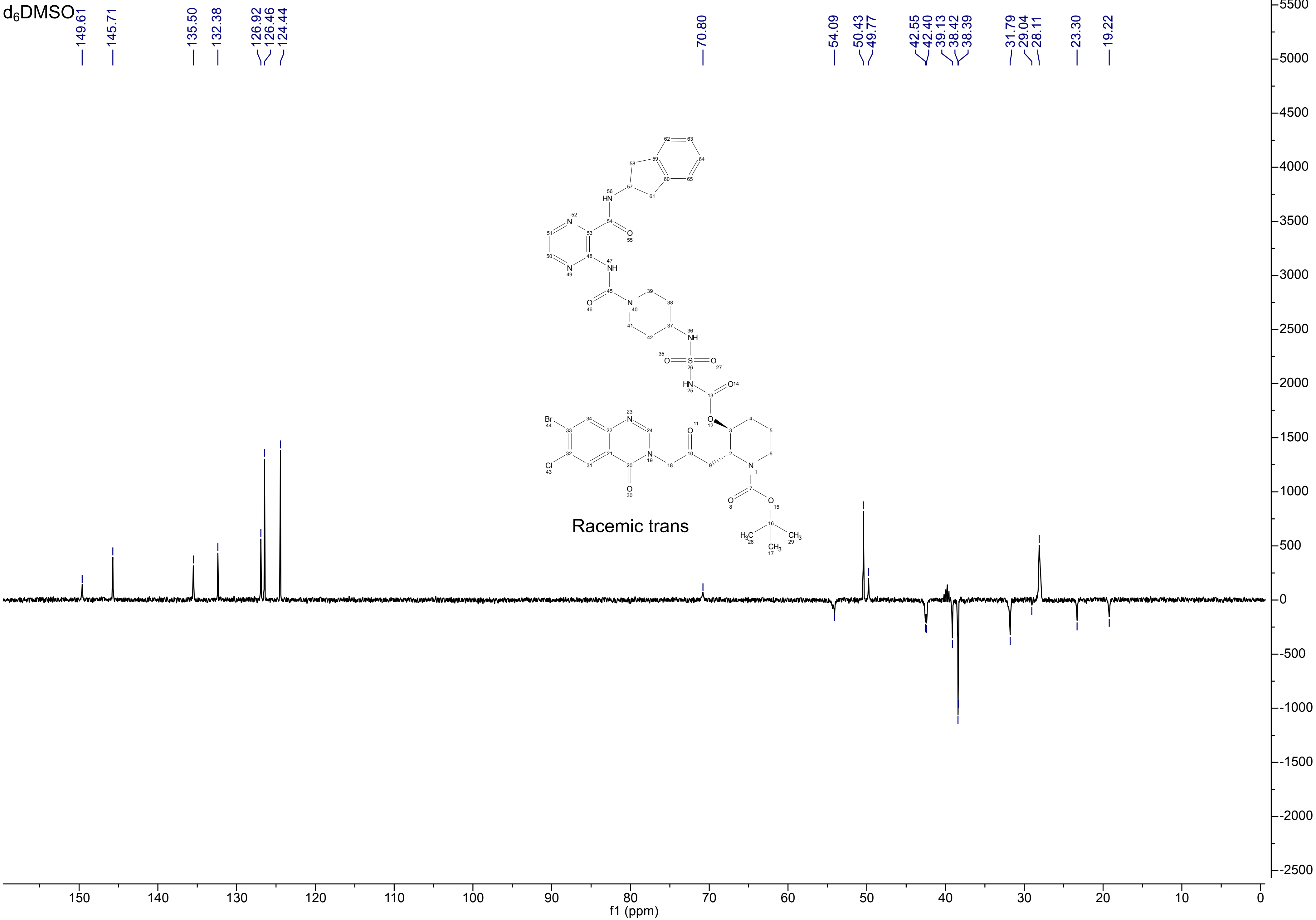

Supplementary Fig. 134. ^1H-^1H COSY NMR of compound **39**.
d_6DMSO

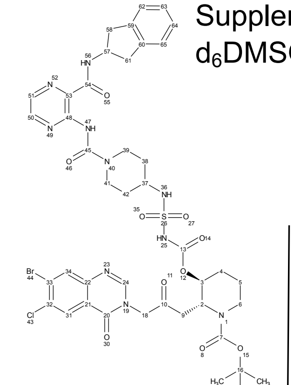

Racemic trans

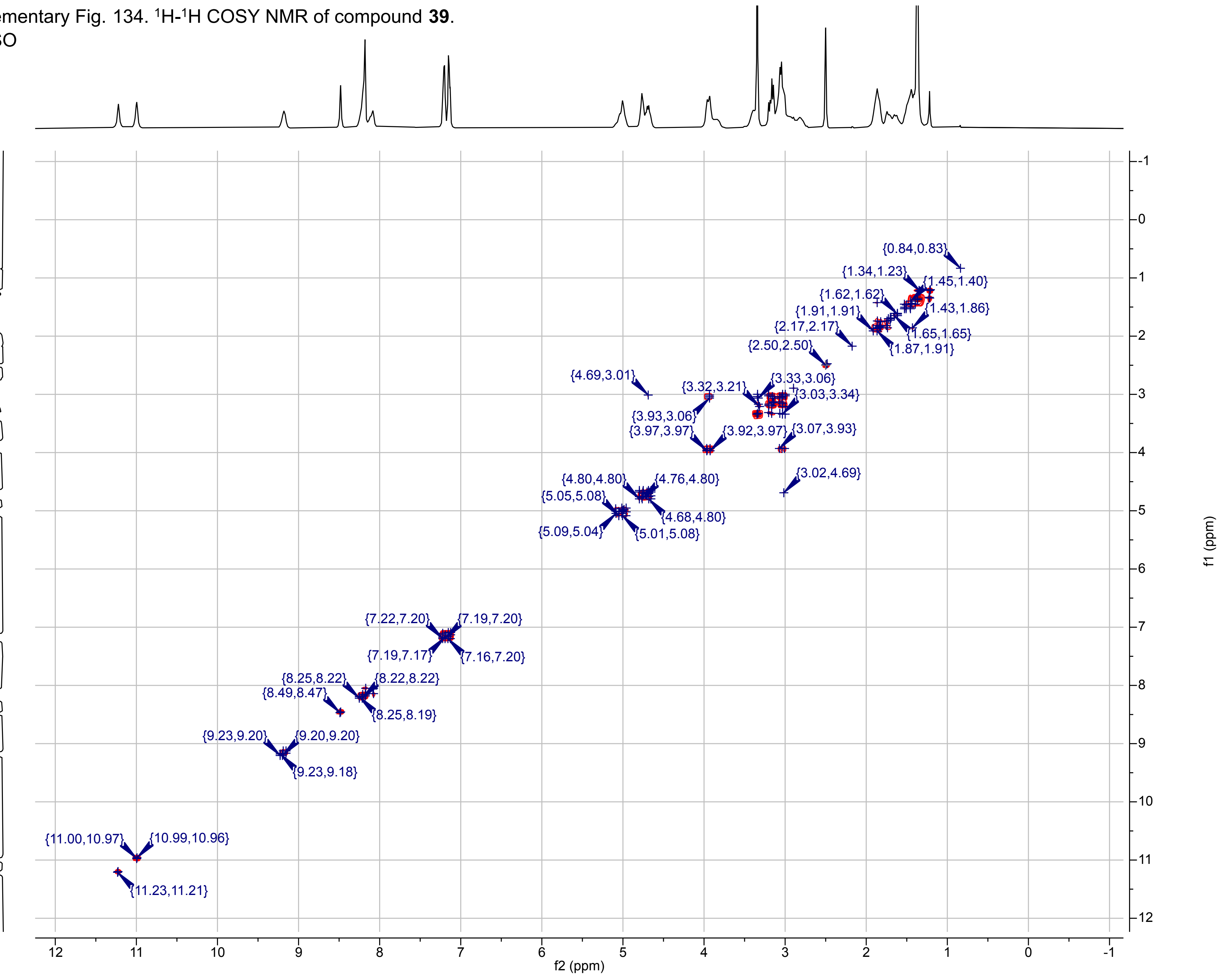

Supplementary Fig. 135. ^{1}H-^{13}C HMBC NMR of compound **39**.
d_6DMSO

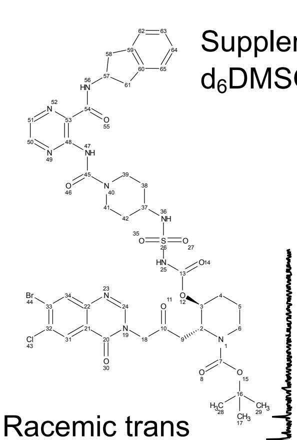

Racemic trans

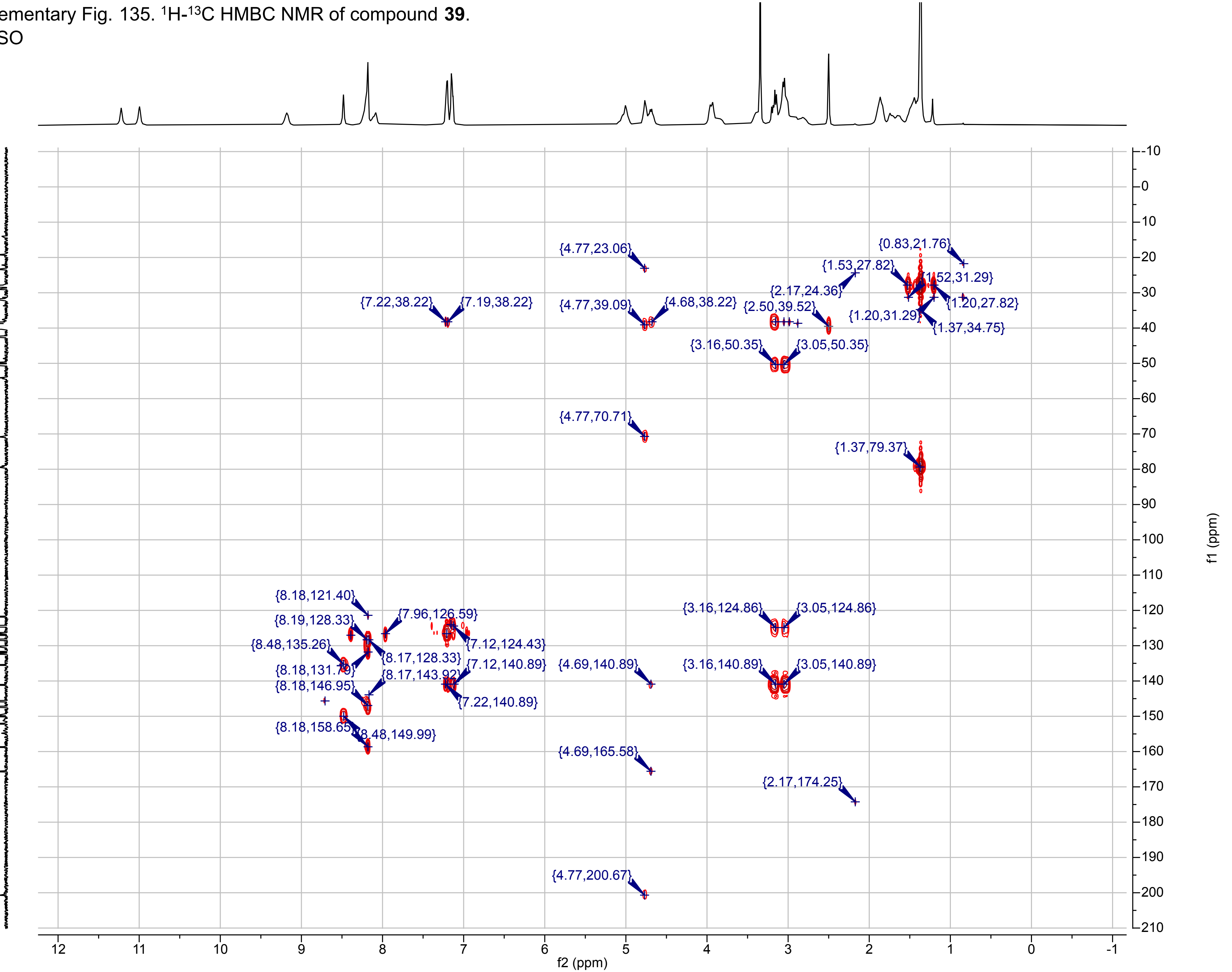

Supplementary Fig. 136. ^{1}H-^{13}C HSQC NMR of compound **39**.
d$_6$DMSO

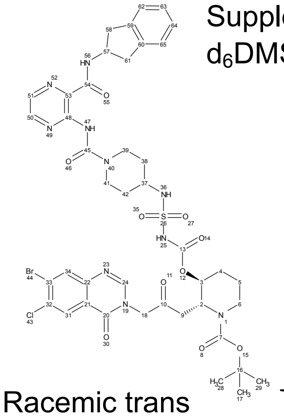

Racemic trans

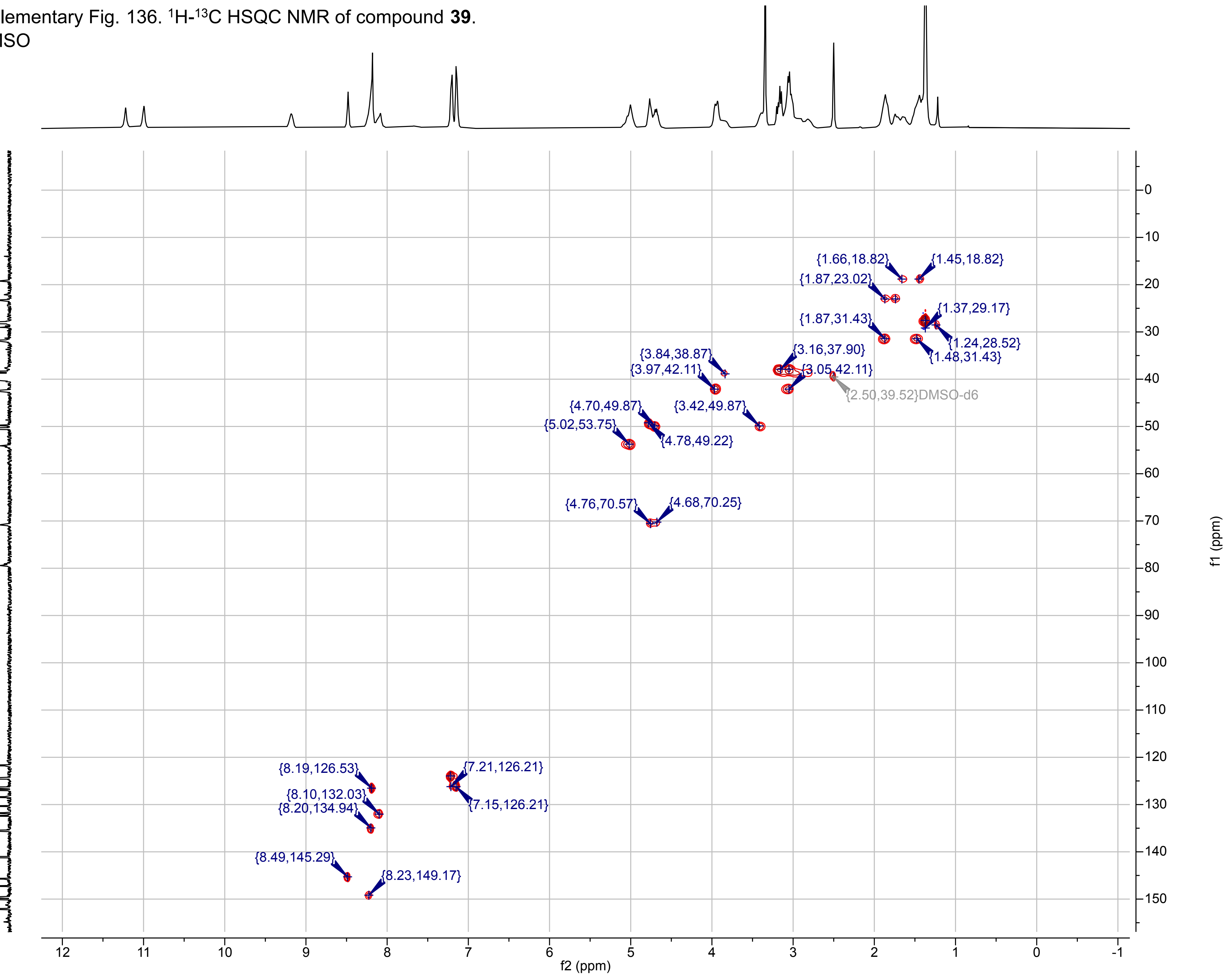

Supplementary Fig. 137. ^1H NMR of compound **36**.
d_6DMSO

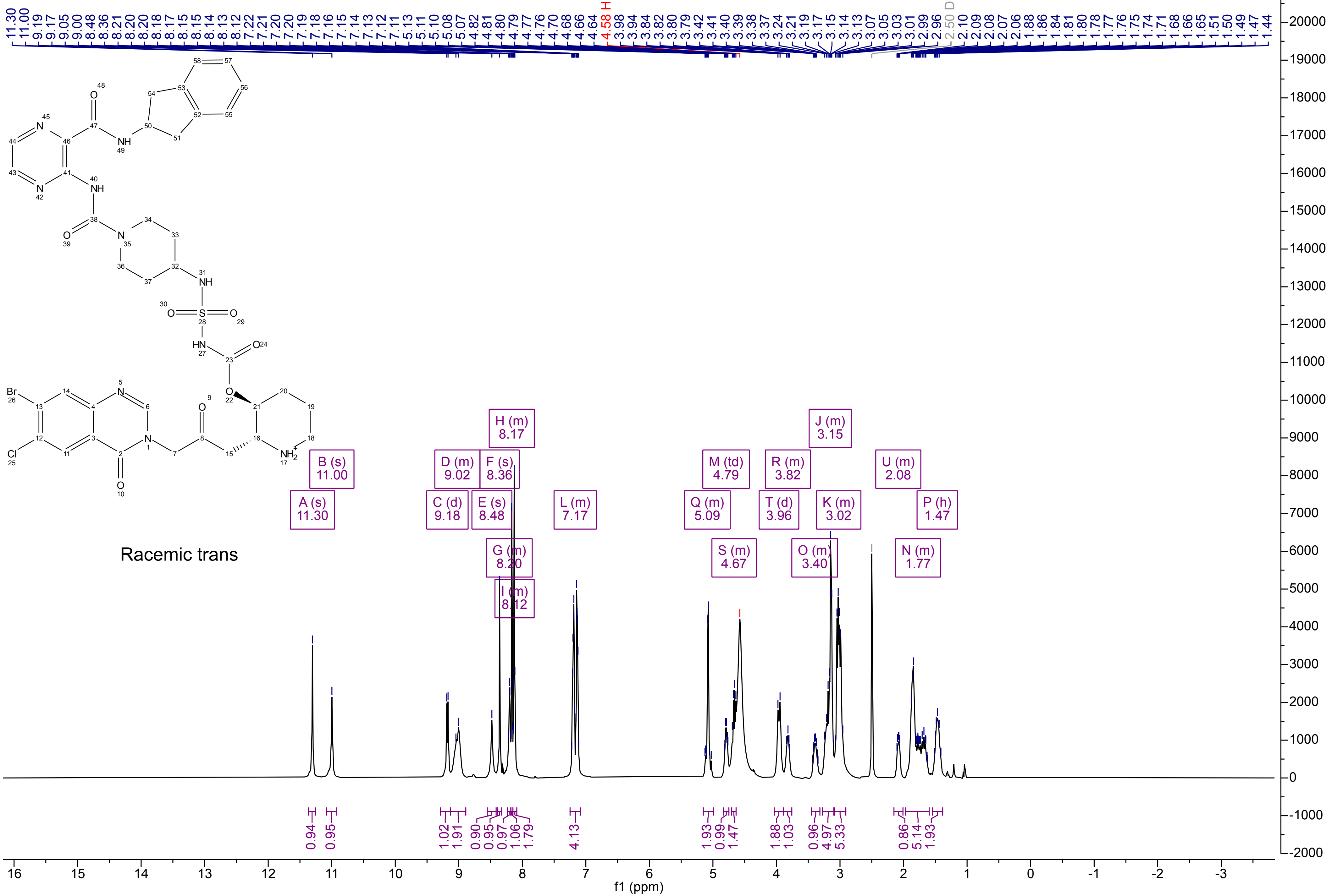

Supplementary Fig. 138. ^{13}C NMR of compound **36**.
d$_6$DMSO

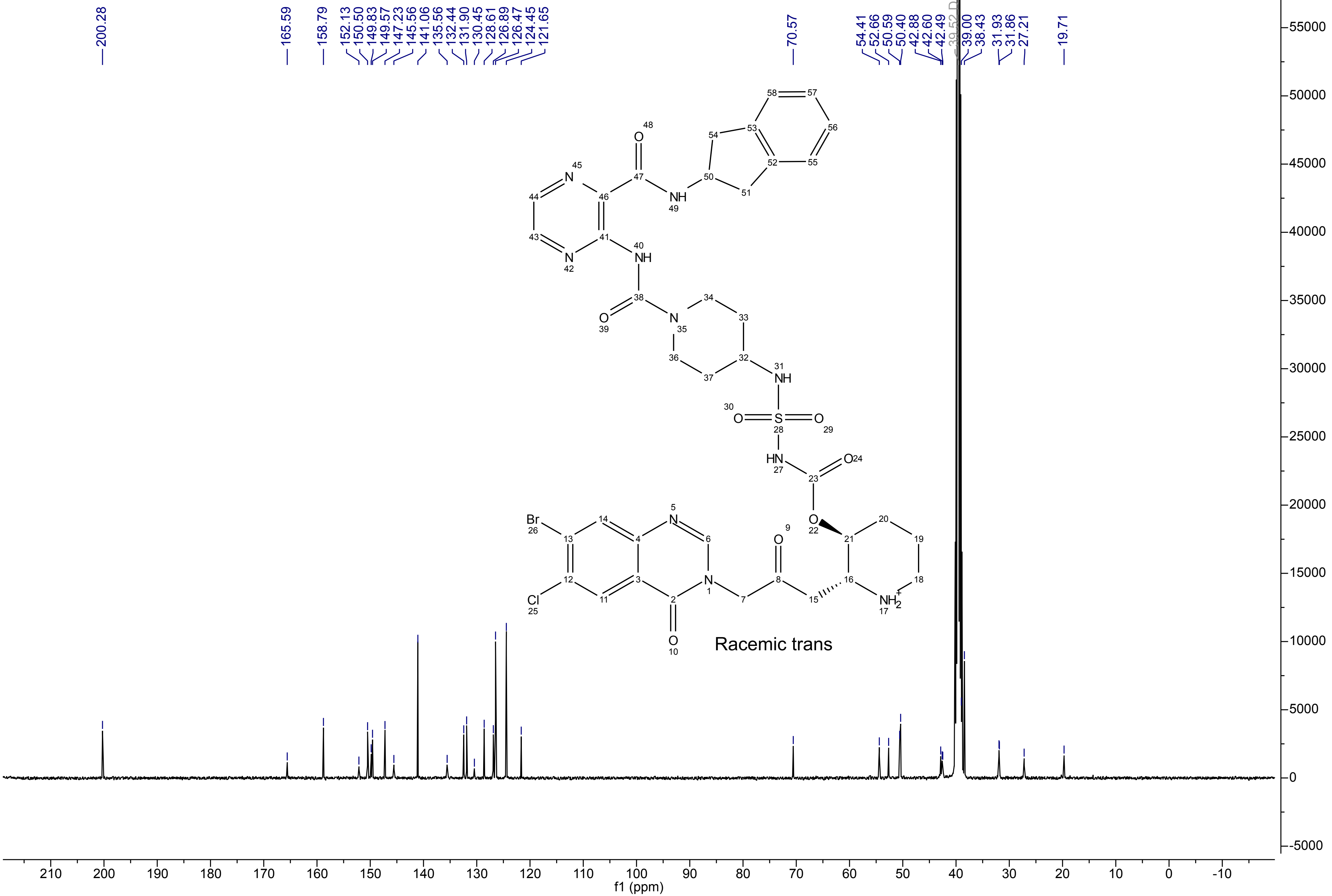

Supplementary Fig. 139. DEPT-135 NMR of compound **36**.

d$_6$DMSO

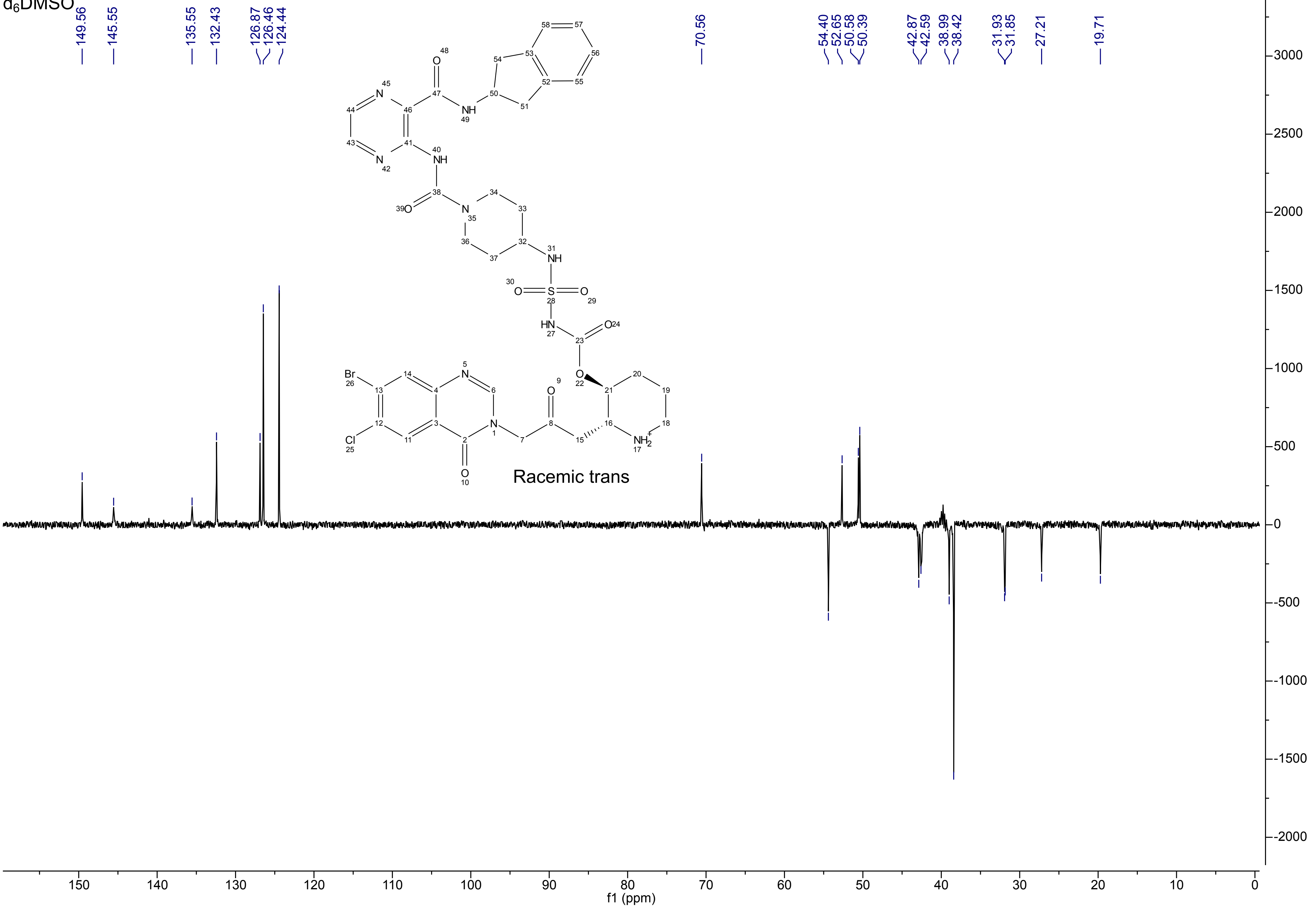

Supplementary Fig. 140. ^{1}H-^{1}H COSY NMR of compound **36**.
d_6DMSO

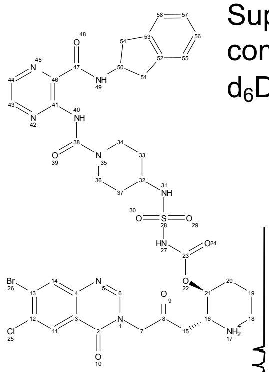

Racemic trans

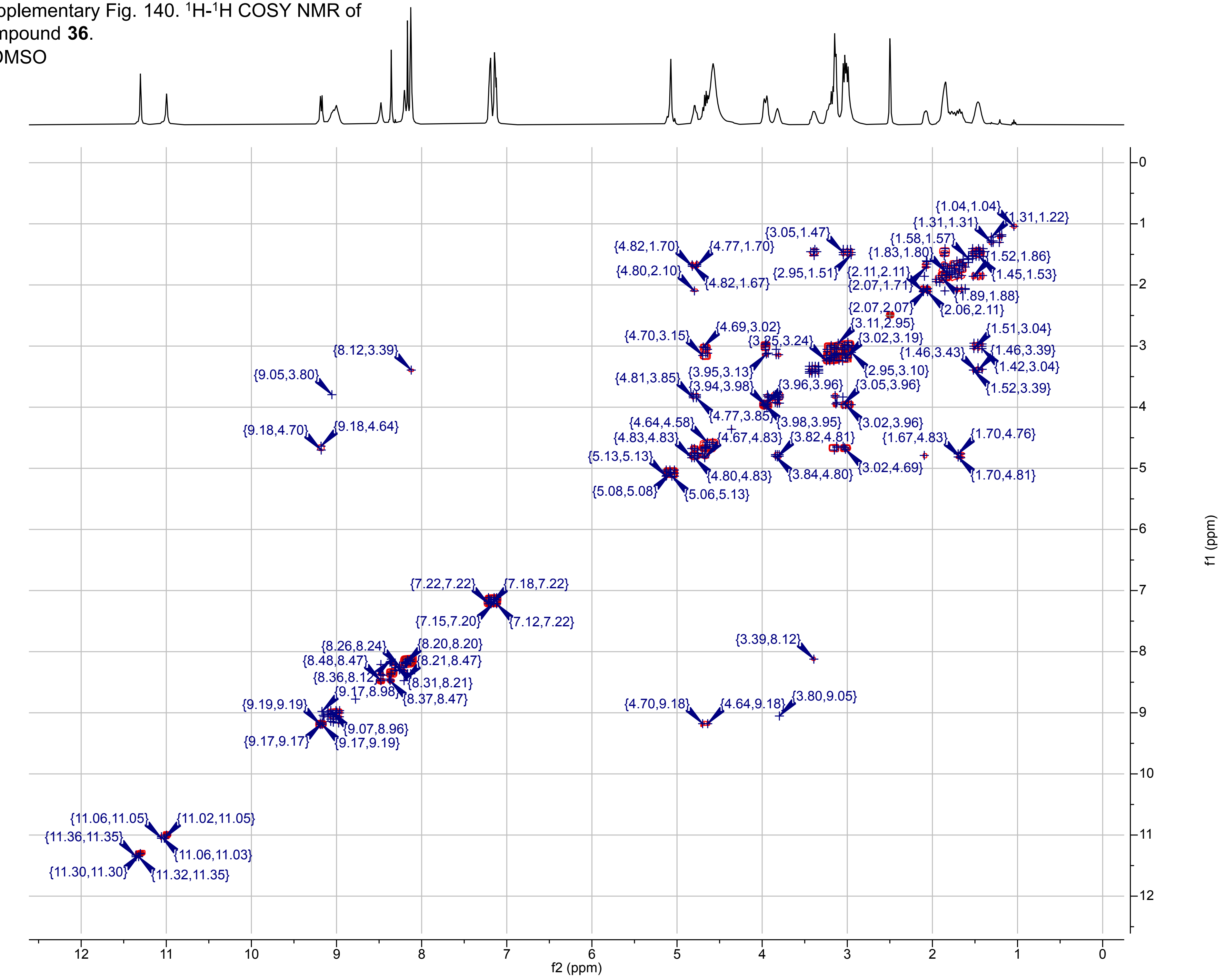

Supplementary Fig. 141. ^{1}H-^{13}C HMBC NMR of compound **36**.
d_6DMSO

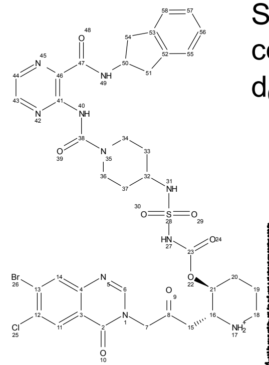

Racemic trans

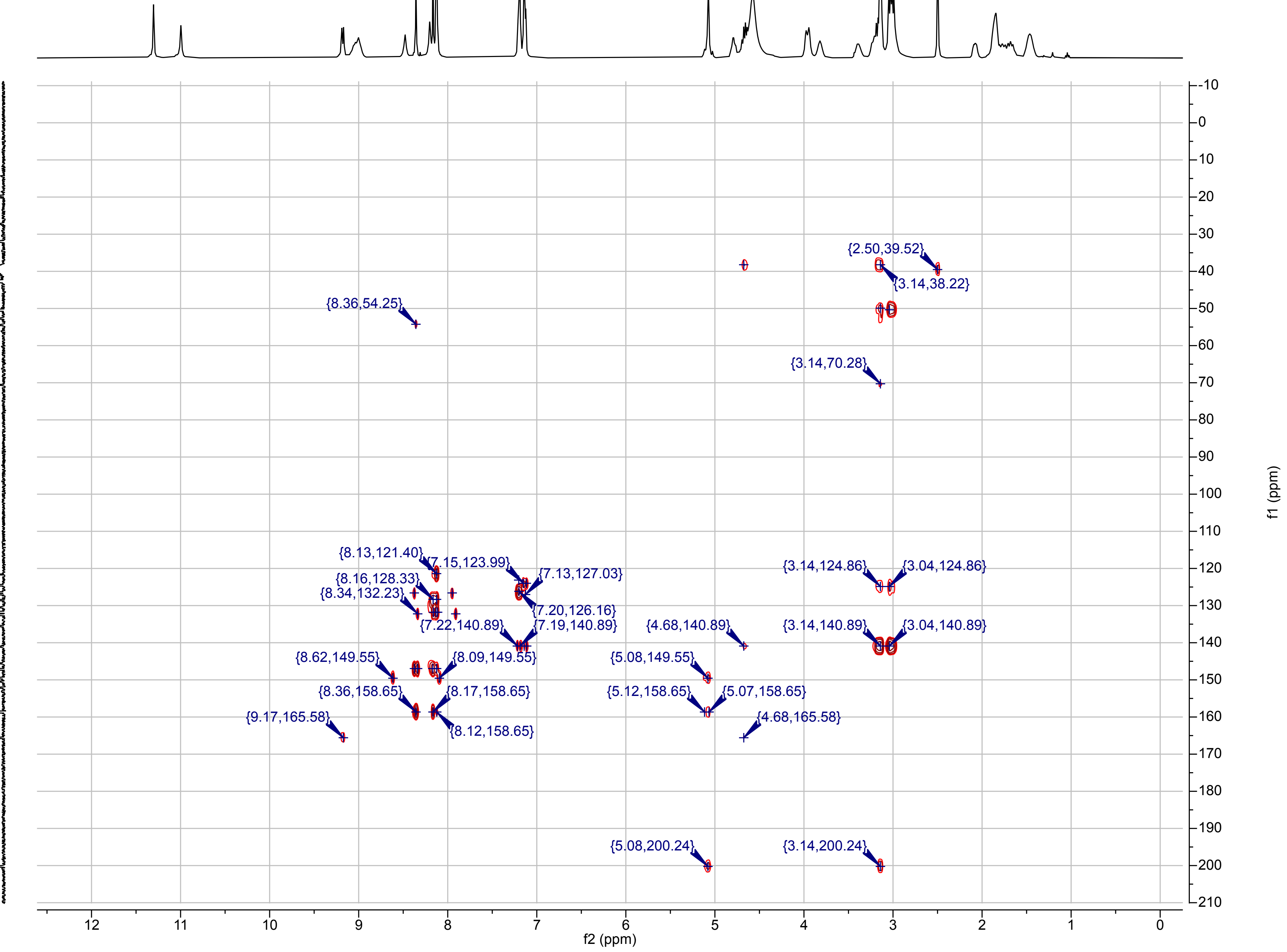

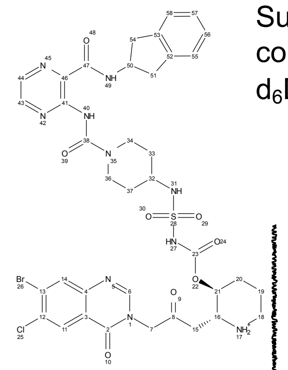

Racemic trans

Supplementary Fig. 142. ^{1}H-^{13}C HSQC NMR of compound **36**.
d_6DMSO

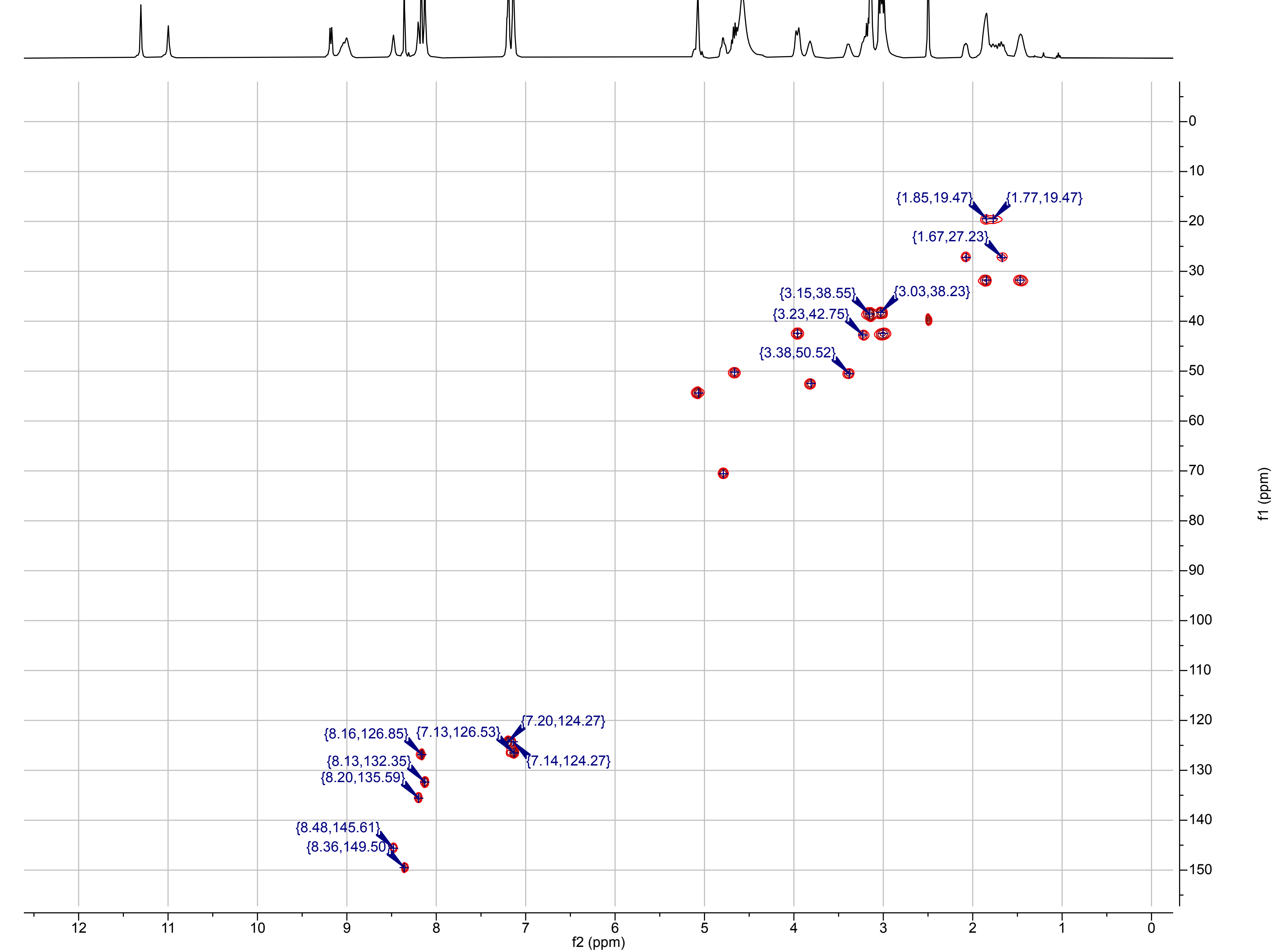

Supplementary Fig. 143. ^{1}H NMR of compound **46**.
$CDCl_3$

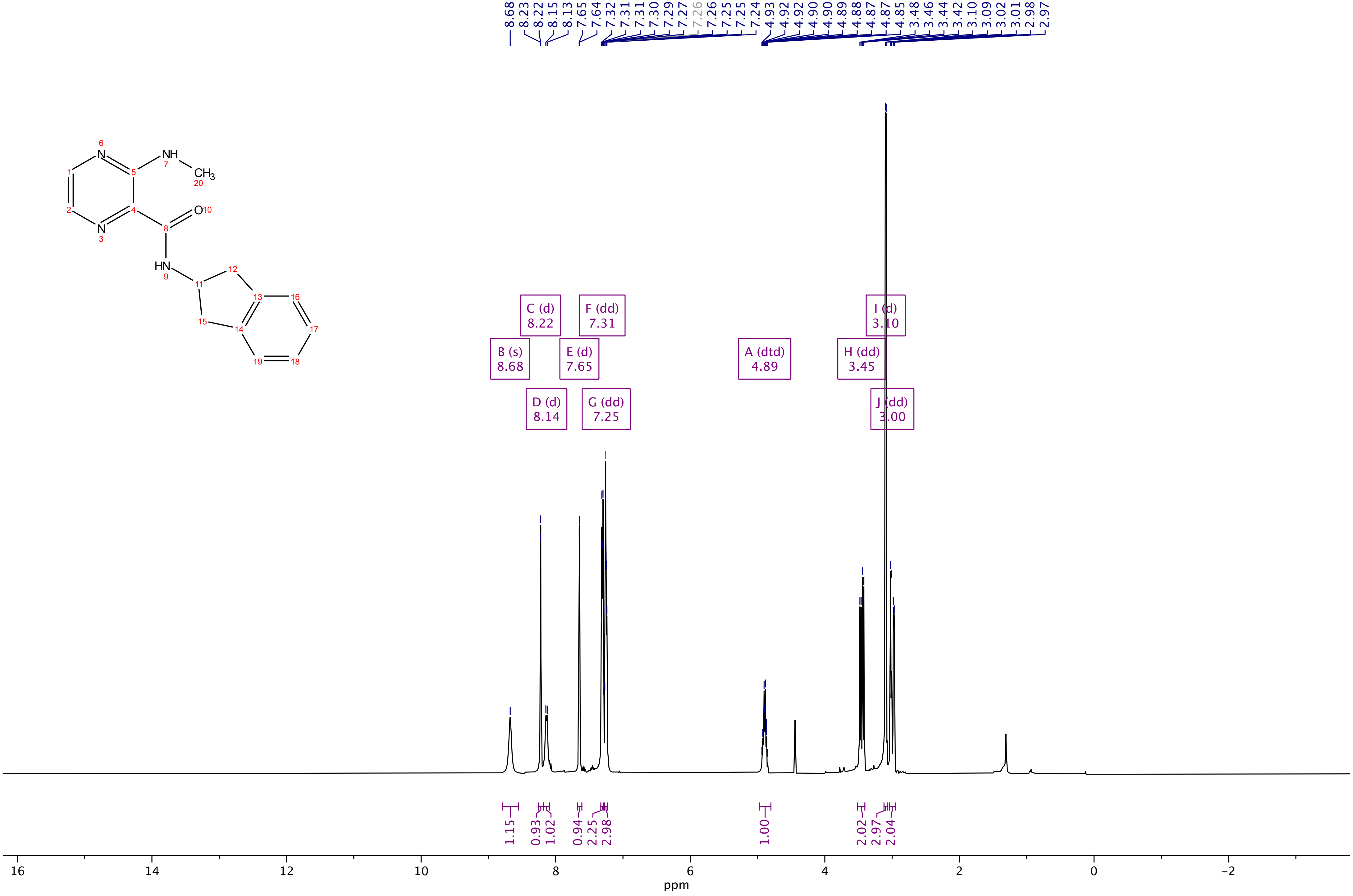

Supplementary Fig. 144. ^{13}C NMR of compound **46**.
$CDCl_3$

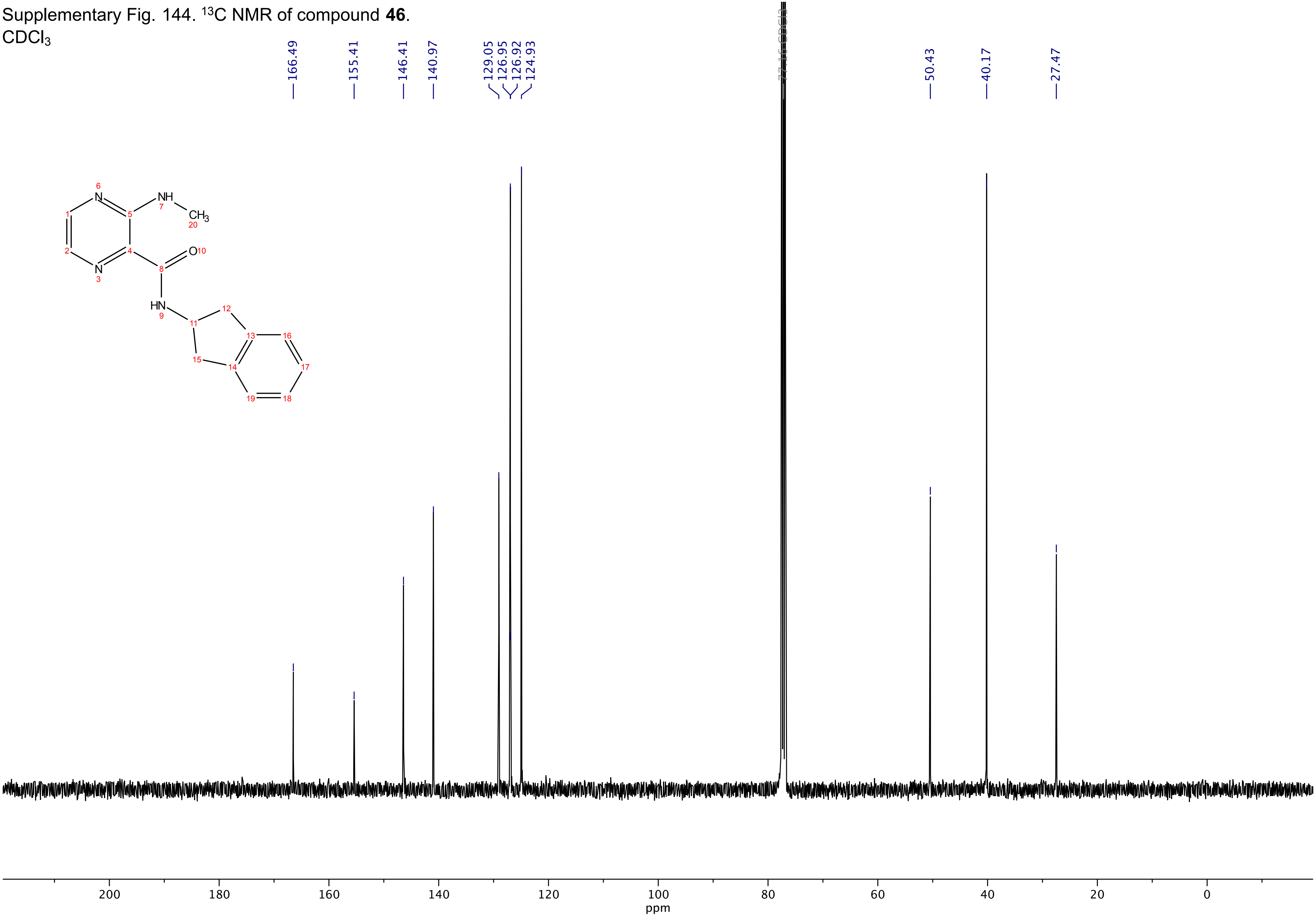

Supplementary Fig. 145. ^{1}H NMR of compound **47**.
d_6DMSO

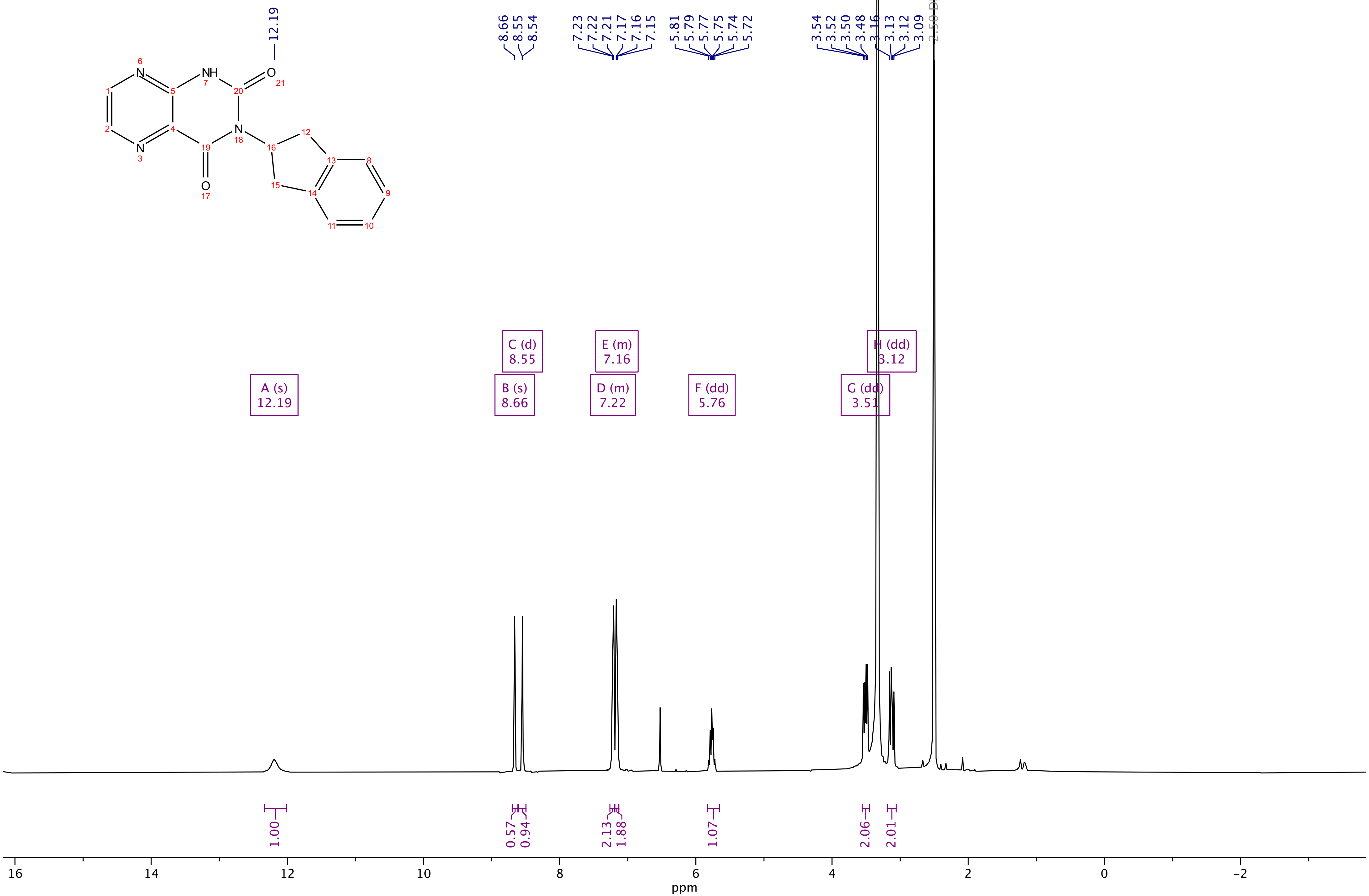

Supplementary Fig. 146. ^{13}C NMR of compound **47**.
d_6DMSO

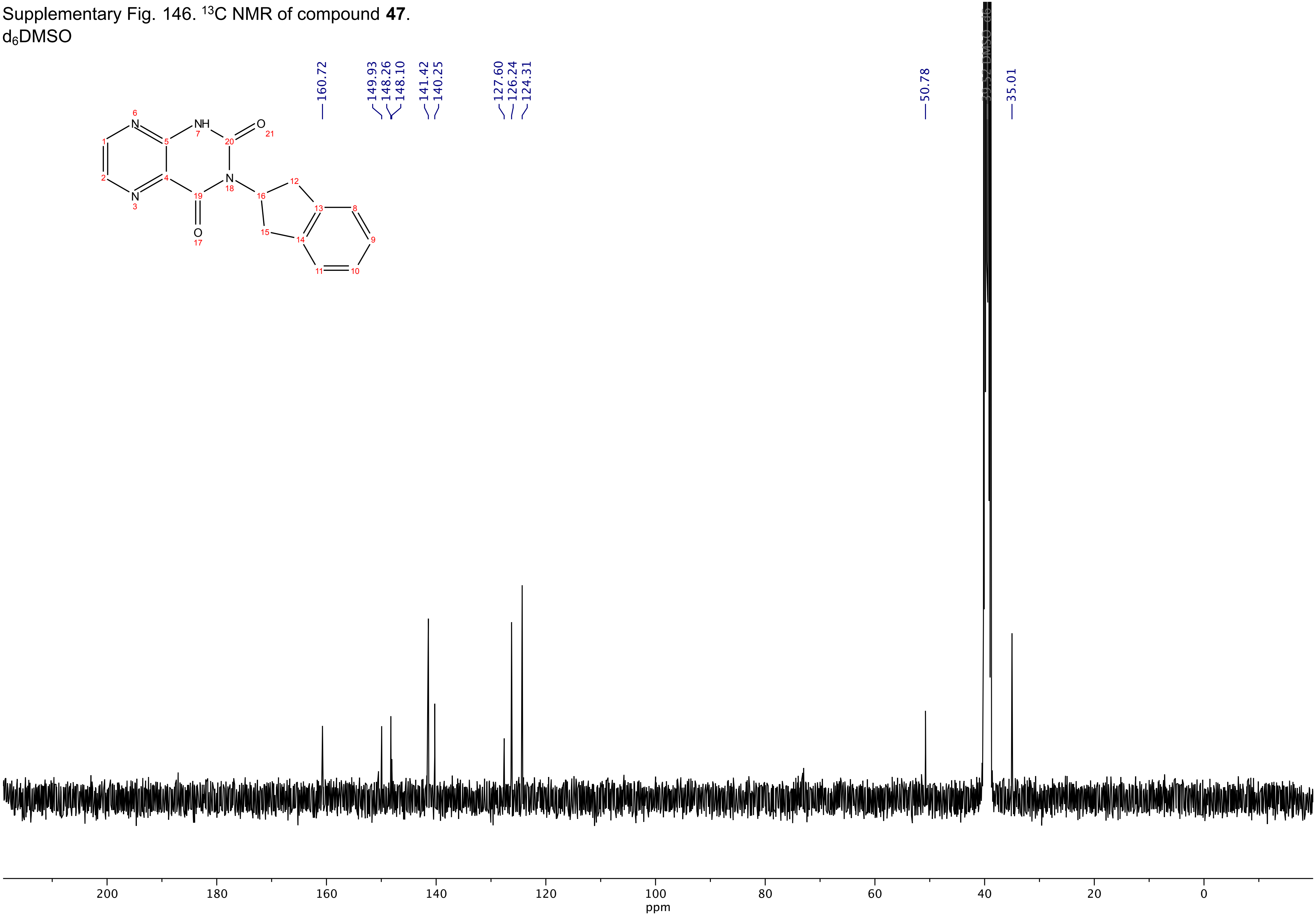

Supplementary Fig. 147. ^{1}H NMR of compound **48**.
$CDCl_3$

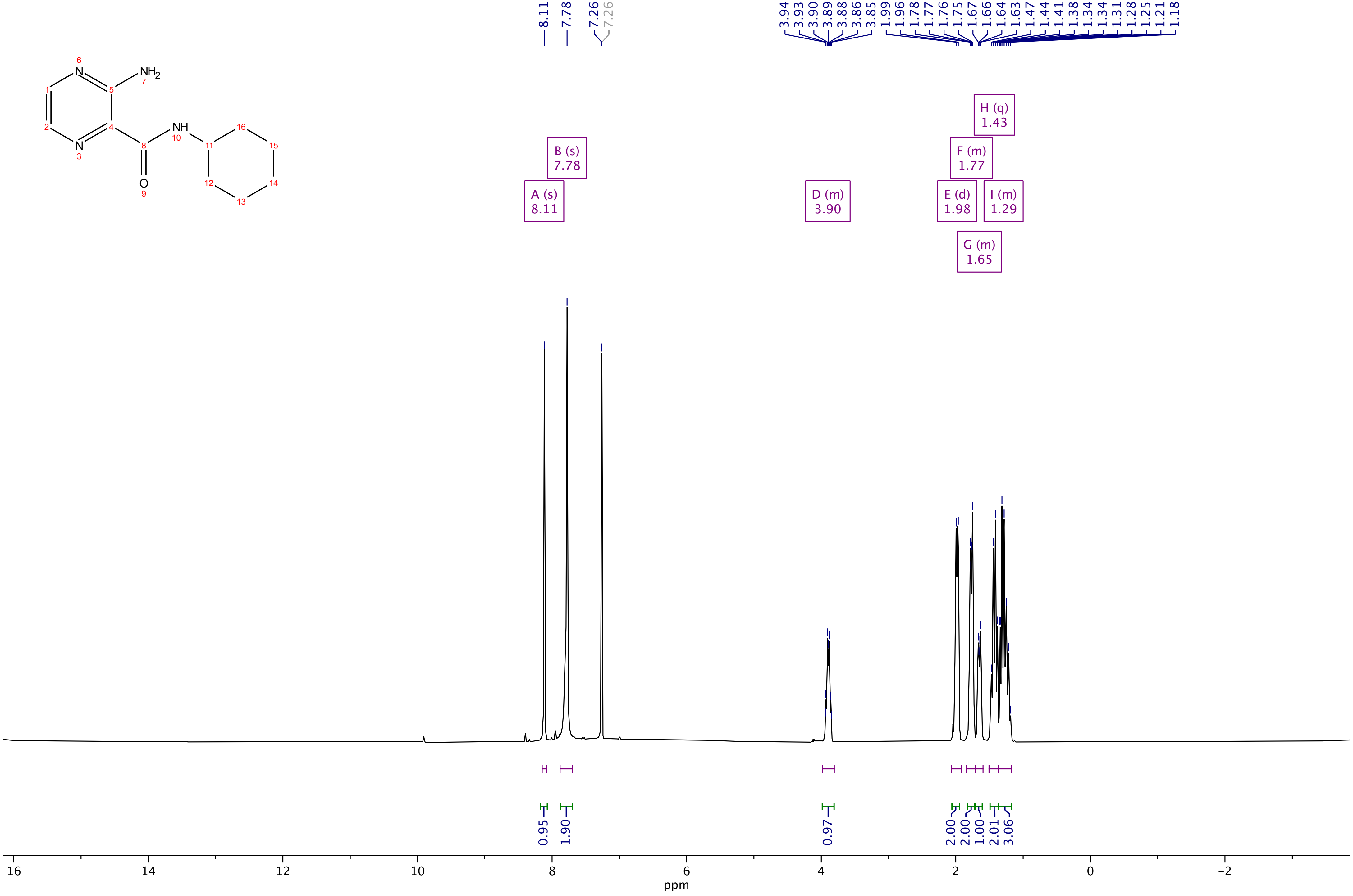

Supplementary Fig. 148. ^{13}C NMR of compound **48**.
$CDCl_3$

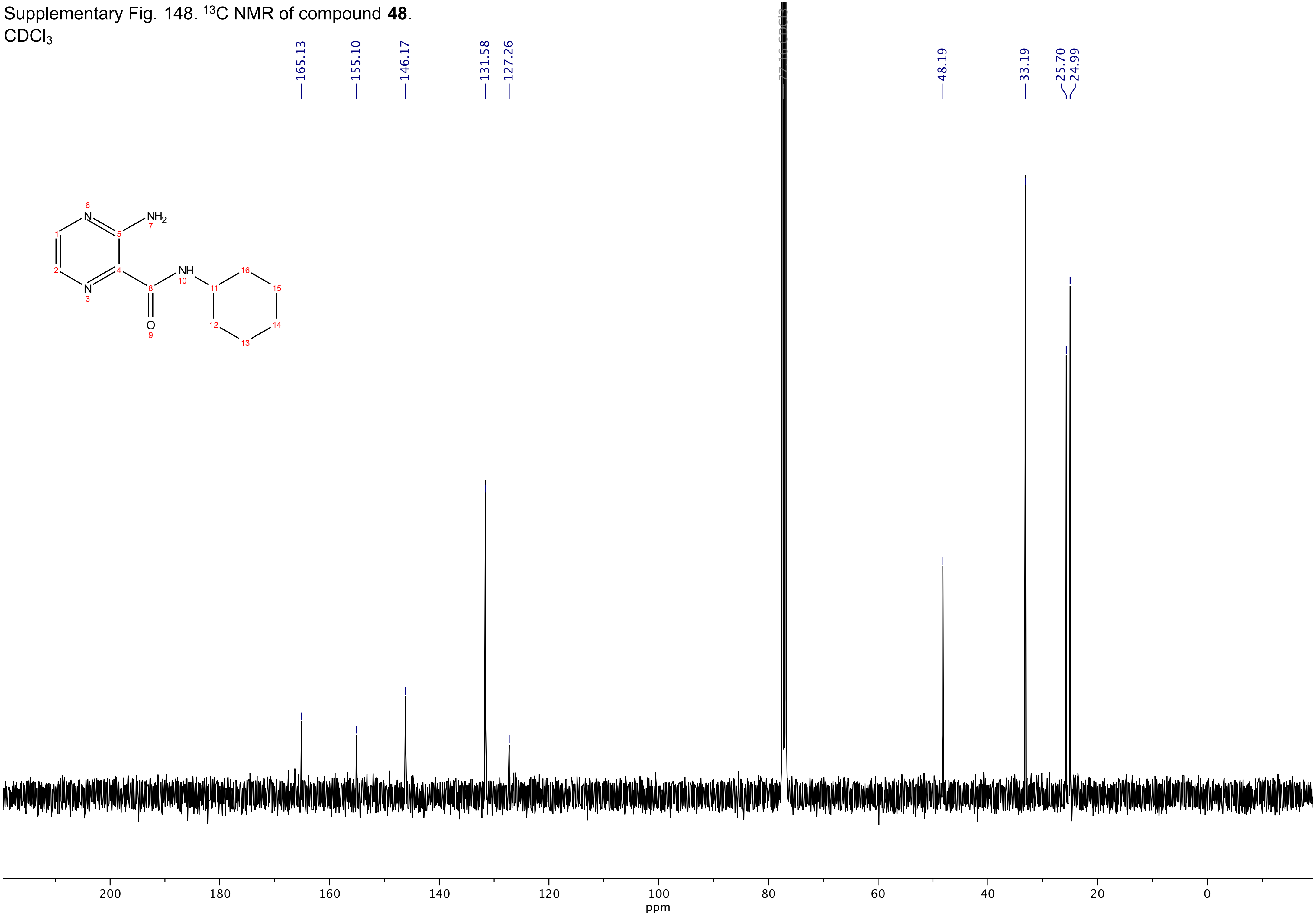

Supplementary Fig. 149. ^1H NMR of compound **49**.
$CDCl_3$

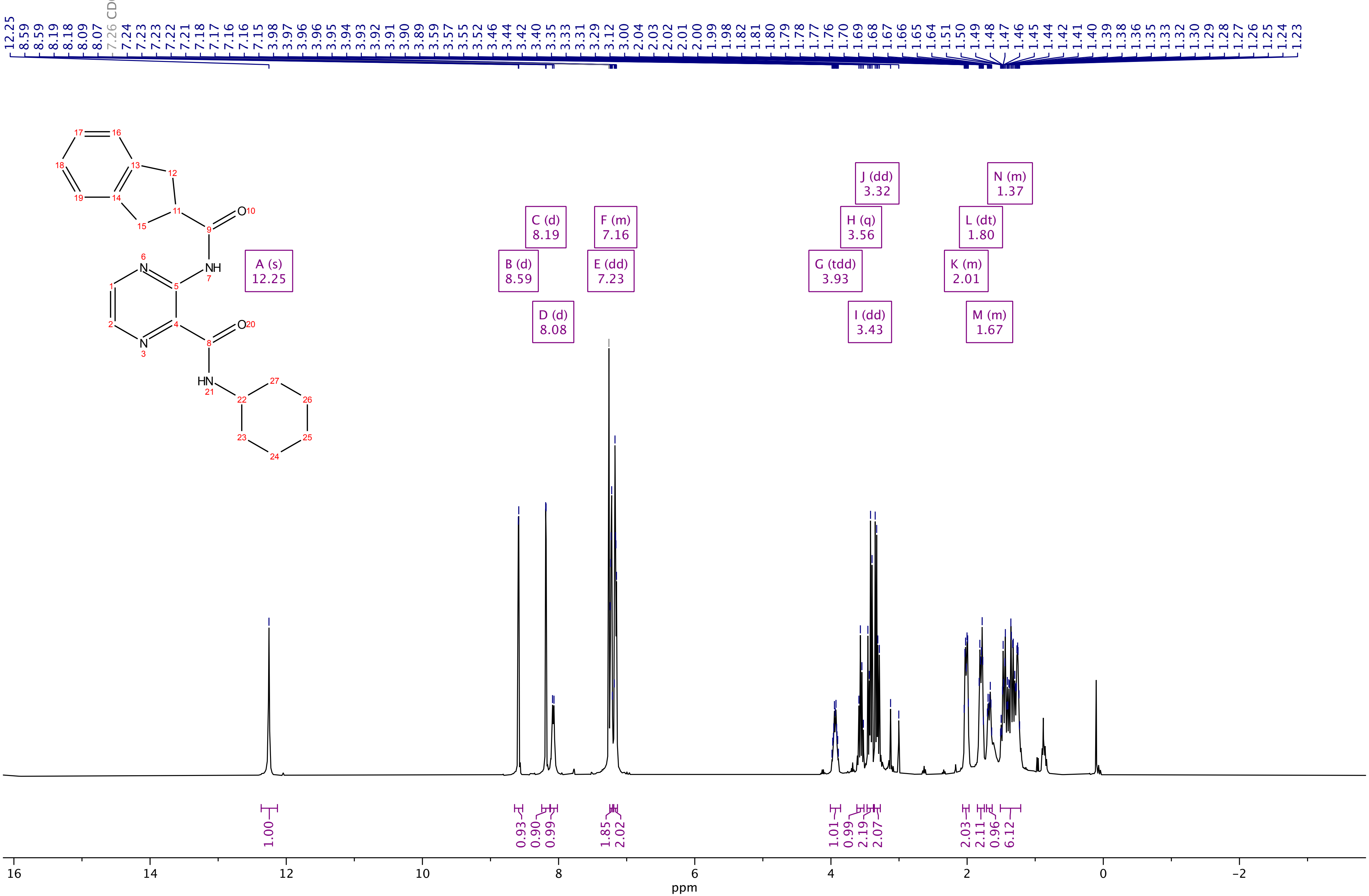

Supplementary Fig. 150. ^{13}C NMR of compound **49**.
$CDCl_3$

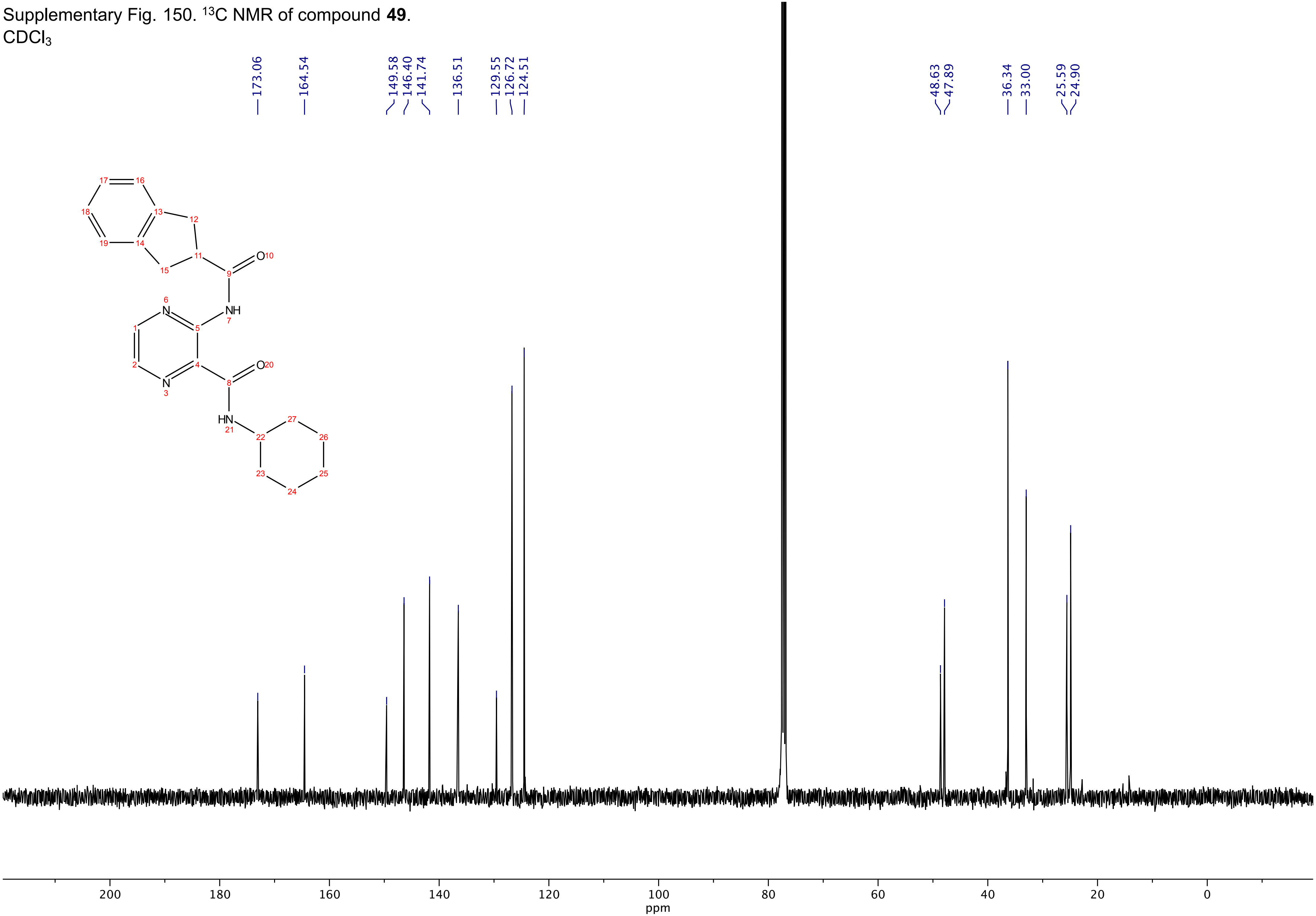

Supplementary Fig. 151. ^{1}H NMR of compound **50**.
$CDCl_3$

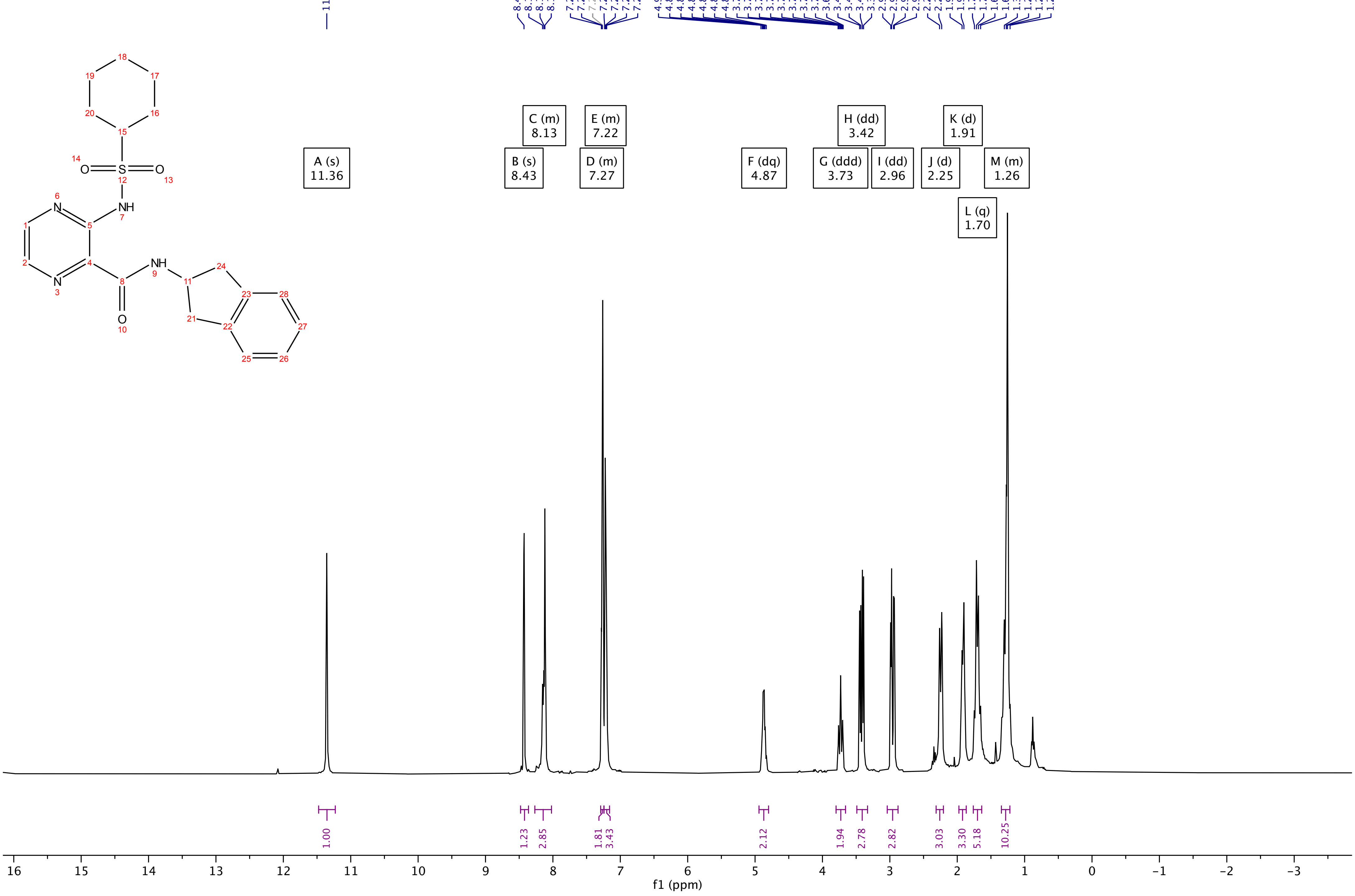

Supplementary Fig. 152. ^{13}C NMR of compound **50**.
$CDCl_3$

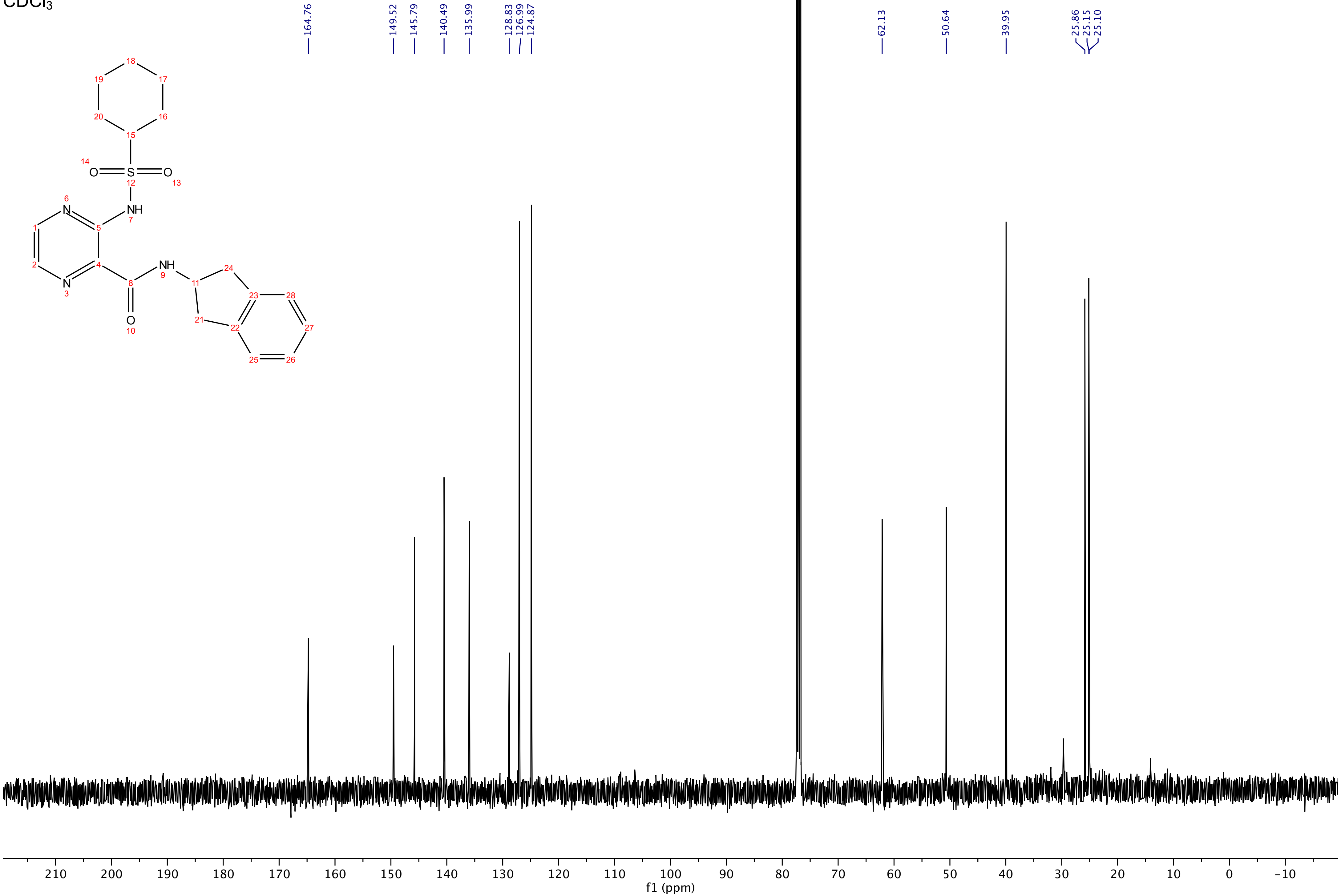

Supplementary Table

Supplementary Table 1. Data collection and refinement statistics for *Pf*cProRS ligand complexes. Data were collected from one crystal for each structure. Statistics for the highest-resolution shell are shown in parentheses.

Compound (PDB id)	NCP26 and proline (6T7K)	MAT334 and proline (7QC2)	MAT345 and proline (7QB7)	MAT436 (7QC1)
**Data collection**				
Space group	P 3_2 2 1	P 3_2 2 1	P 3_2 2 1	P 4_3 2_1 2
Cell dimensions				
a, b, c (Å)	103.0, 103.0,126.83	103.5, 103.5, 127.51	102.96, 102.96, 127.12	207.21, 207.21, 115.85
α, β, γ (°)	90, 90, 120	90, 90, 120	90, 90, 120	90, 90, 90
Resolution (Å)	89.20 - 1.79	51.95 - 2.28	51.76 - 1.93	65.53 - 2.51
Unique reflections	73725 (3643)	36445 (1805)	61850 (3016)	86283 (4261)
R_{merge}	0.077 (1.72)	0.062 (1.68)	0.073 (2.64)	0.261 (5.9)
$I / \sigma I$	18.1 (1.2)	18.4 (1.2)	21.4 (0.8)	11.5 (0.6)
CC-half	1.0 (0.84)	1.0 (0.69)	1.0 (0.58)	1.0 (0.36)
Completeness (%)	100 (99.70)	99. 5 (99.3)	100 (99.44)	99.92 (99.69)
Wilson B-factor	34.29	56.86	38.45	54.55
**Refinement**				
Resolution (Å)	51.68-1.79	51.95-2.28	44.58-1.90	65.52-2.51
No. reflections	73610 (7254)	36394 (3617)	61755 (6053)	86161 (8481)
R_{work} / R_{free}	0.172/0.195	0.227/0.253	0.183/0.210	0.194/0.233
Macromolecules (atoms)	4039	3865	3937	11641
Ligands (atoms)	111	47	88	211
Solvent (atoms)	239	45	184	73
RMS (bonds)	0.014	0.002	0.011	0.005
RMS (angles)	1.18	0.44	1.11	0.69
Ramachandran favored (%)	98.97	98.55	97.71	97.84
Ramachandran allowed (%)	1.03	1.45	2.29	2.16
Ramachandran outliers (%)	0.00	0.00	0.00	0.00
Rotamer outliers (%)	1.15	0.26	0.95	0.41
Clashscore	3.65	3.06	4.03	3.07
**Average B-factor**	46.4	84.5	50.3	75.5
macromolecules	46.0	84.9	50.1	75.7
ligands	53.2	67.2	57.0	68.1
solvent	49.7	70.2	52.1	66.8

Supplementary Methods:
General Methods:

Unless otherwise noted, all reagents were used as received from vendors.

Column purifications were performed on a Biotage Isolera 4 Purification System equipped with a 200- 400 nm diode array detector. For normal phase flash column chromatography purifications, Sorbtech Purity Flash Cartridges were used (CFC-52300-012-18 and CFC-52500-025-12). For reverse phase flash column chromatography purifications, Biotage SNAP KP-C18-HS (FSL0-1118-0012 and FSL0-1118-0030) and Biotage Sfär Bio C18 Duo 300 Å, 20 µm cartridges were used (FSBD-0411-0010 and FSBD-0411-0025).
Analytical LC-MS was performed on a Waters 2545 HPLC equipped with a 2998 diode array detector, a 2424 evaporative light scattering detector, a 2475 multichannel fluorescence detector, and a Waters 3100 ESI-MS module, using a XTerraMS C18, 5 µm, 4.6 x 50 mm column at a flow rate of 5 mL/min with a linear gradient (95% A: 5% B to 100% B over 90 sec and 30 sec hold at 100% B; solvent A = water + 0.1% formic acid, solvent B = acetonitrile + 0.1% formic acid). LC-MS data analysis was performed using Waters Masslynx V4.1 SCN 846 software.
Proton and carbon nuclear magnetic resonance (^{1}H and ^{13}C NMR spectra) were recorded on a Bruker Avance III 400 spectrometer using Topspin 3.2 software and data were analyzed using MestreNova (version 12.0.1-20560, Mestrelab Research). Chemical shifts for NMR spectra are reported in parts per million (ppm) and are referenced to residual solvent peaks (except for ^{13}C NMR in D_2O). Data is reported as follows: chemical shift, multiplicity (s = singlet, br s, = broad singlet, d = doublet, t = triplet, q = quartet, p = pentet, m = multiplet), proton coupling constants (*J*, Hz), and integration.

Abbreviations:

DCC (*N,N'*-Dicyclohexylcarbodiimide),

DCM (dichloromethane),

DIPEA (ethyldiisopropylamine),

DMAc (*N,N*-dimethylacetamide),

DMF (*N,N*-dimethylformamide),

DMSO (dimethylsulfoxide),

EtOAc (ethyl acetate),

LC-MS (Liquid Chromatography – Mass Spectrometry),

MeCN (acetonitrile),

MeOH (methanol),

MTBE (methyl *tert*-butyl ether),

NHS (*N*-hydroxysuccinimide),

THF (tetrahydrofuran).

3-amino-*N*-(2,3-dihydro-1*H*-inden-2-yl)pyrazine-2-carboxamide (45):

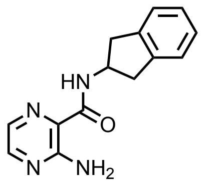

7.8 mL isobutyl chloroformate (8.1 g, 59 mmol, 1.1 eq) was added to a solution of 7.5 g 3-aminopyrazine-2-carboxylic acid (54 mmol, 1 eq) and 28 mL DIPEA (21 g, 160 mmol, 3 eq) in 600 mL DCM under argon and stirred for 16 h, followed by the addition of 9.2 g 2,3-dihydro-1H-inden-2-amine (54 mmol, 1 eq). The reaction mixture was stirred for an additional 24 h, diluted with 150 mL DCM and 5 mL MeOH. The organic layer was then washed twice with 1:1 saturated $NaHCO_{3(aq)}/H_2O$ (300 mL each) and washed once with 1:1 saturated $NaCl_{(aq)}/H_2O$ (300 mL), keeping the emulsion with the organic layer each time. Insoluble material (largely product, but not pure) was collected by filtration. The organic layer was dried over anhydrous Na_2SO_4, filtered, and concentrated *in vacuo*. Recombined the concentrated material with the filtrand from before. Triturated the combined solids with MeCN (50 mL) (note: sonicated vigorously until the solid was visually homogeneous beige, the MeCN was orange, and no black/brown spots were observed; typically ~10-15 min) and filtered to obtain **45**.

Yield: 11.1 g, 80.7%. Beige solid.

^{1}H NMR (400 MHz, DMSO-d_6) δ 8.81 (d, J = 7.9 Hz, 1H), 8.20 (d, J = 2.1 Hz, 1H), 7.80 (d, J = 2.1 Hz, 1H), 7.55 (s, 2H), 7.22 (dd, J = 5.5, 3.3 Hz, 2H), 7.15 (dd, J = 5.4, 3.2 Hz, 2H), 4.70 (h, J = 7.5 Hz, 1H), 3.17 (dd, J = 15.7, 7.6 Hz, 2H), 3.01 (dd, J = 15.7, 7.3 Hz, 2H).

^{13}C NMR (101 MHz, DMSO-d_6) δ 165.8, 155.2, 146.8, 141.2, 130.8, 126.5, 125.7, 124.5, 50.2, 38.6.

LC-MS ($C_{14}H_{14}N_4O$): Calculated $[M+H]^+$ m/z = 255.12. Observed $[M+H]^+$ m/z 255.33.

3-(cyclohexanecarboxamido)-*N*-(2,3-dihydro-1*H*-inden-2-yl)pyrazine-2-carboxamide (T-3767758, 2):

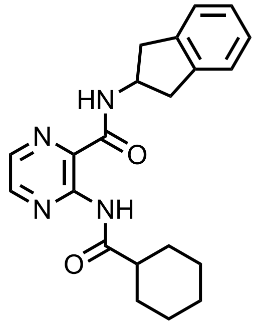

Cyclohexanecarbonyl chloride (92.6 µL, 101 mg, 693 µmol, 1.5 eq) was added to a solution of **45** (117 mg, 462 µmol, 1 eq) and DIPEA (0.24 mL, 1.4 mmol, 3.0 eq) in DCM (5 mL) under an argon atmosphere. After the reaction was complete as determined by LC-MS, the reaction

mixture was diluted with DCM (25 mL) and quenched with H_2O (20 mL). The organic layer was washed twice with 1 M $HCl_{(aq)}$ (20 mL each), washed twice with 1:1 saturated $NaHCO_{3(aq)}/H_2O$ (20 mL each), washed twice with 1:1 saturated $NaCl_{(aq)}/H_2O$ (20 mL each), dried over anhydrous Na_2SO_4, filtered, and concentrated *in vacuo*. Purified by normal phase flash column chromatography (20%-60% EtOAc in hexanes gradient) to obtain **2**.

Yield: 45.5 mg, 27%.

^{1}H NMR (400 MHz, Chloroform-*d*) δ 11.98 (s, 1H), 8.57 (d, *J* = 2.3 Hz, 1H), 8.33 (d, *J* = 8.2 Hz, 1H), 8.11 (s, 1H), 7.29 – 7.25 (m, 2H), 7.24 – 7.19 (m, 2H), 4.88 (tt, *J* = 12.3, 8.1, 4.8 Hz, 1H), 3.43 (dd, *J* = 16.2, 7.2 Hz, 2H), 2.97 (dd, *J* = 16.2, 4.8 Hz, 2H), 2.44 (tt, *J* = 11.7, 3.6 Hz, 1H), 2.05 (d, *J* = 12.9 Hz, 2H), 1.91 – 1.80 (m, 2H), 1.71 (d, *J* = 10.0 Hz, 1H), 1.59 (q, *J* = 12.2 Hz, 2H), 1.42 – 1.24 (m, 3H).

^{13}C NMR (101 MHz, $CDCl_3$) δ 174.7, 165.3, 149.7, 146.6, 140.6, 136.3, 129.2, 127.1, 124.9, 50.7, 47.5, 40.1, 29.5, 25.8, 25.8.

LC-MS ($C_{21}H_{24}N_4O_2$): Calculated $[M+H]^+$ m/z = 365.19, $[M-H]^-$ m/z = 363.19. Observed $[M+H]^+$ m/z = 365.28, $[M-H]^-$ m/z = 363.09.

***N*-(2,3-dihydro-1*H*-inden-2-yl)-3-(piperidine-1-carboxamido)pyrazine-2-carboxamide (NCP26, 3)**

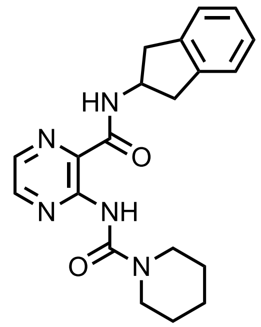

To a solution of **45** (3.00 g, 11.8 mmol, 1 eq) in anhydrous DCM (300 mL), added anhydrous lutidine (5.5 mL, 47 mmol, 4 eq) and cooled to 0°C. Over the course of 10 min, added 15%w/v $phosgene_{(toluene)}$ (11.8 mL, 16.5 mmol, 1.4 eq) slowly along the walls and then stirred 40 min at 0°C. In parallel, charged a second flask with piperidine (2.33 mL, 23.6 mmol, 2 eq) and DCM (150 mL). Over the course of 3 min, added the phosgene solution to the piperidine solution and stirred at room temperature overnight. Then, the reaction was quenched by addition of MeOH (30 mL) and stirred at room temperature for 30 min. Added granular silica (~20 g) directly to the reaction mixture, concentrated reaction *in vacuo*, and purified by normal phase flash column chromatography (20-100% EtOAc in hexanes gradient) to obtain NCP26.

Yield: 2.08 g, 48.2%. White solid.

^{1}H NMR (400 MHz, Chloroform-*d*) δ 11.37 (s, 1H), 8.47 (s, 1H), 8.28 (d, *J* = 8.1 Hz, 1H), 7.96 (s, 1H), 7.26 – 7.22 (m, 2H), 7.20 – 7.16 (m, 2H), 4.84 (h, *J* = 7.5 Hz, 1H), 3.57 (s, 4H), 3.39 (dd, *J* = 16.1, 7.2 Hz, 2H), 2.94 (dd, *J* = 16.1, 4.8 Hz, 2H), 1.63 (s, 6H).

^{13}C NMR (101 MHz, $CDCl_3$) δ 165.8, 152.5, 151.3, 146.6, 140.5, 134.6, 128.5, 127.0, 124.8, 50.5, 45.2, 40.0, 25.8, 24.5.

LC-MS ($C_{20}H_{23}N_5O_2$): Calculated $[M+H]^+$ m/z = 366.19, $[M-H]^-$ m/z = 364.19. Observed $[M+H]^+$ m/z = 366.40, $[M-H]^-$ m/z = 364.13.

_N_-(2,3-dihydro-1_H_-inden-2-yl)-3-(1-methylcyclohexane-1-carboxamido)pyrazine-2-carboxamide (4):

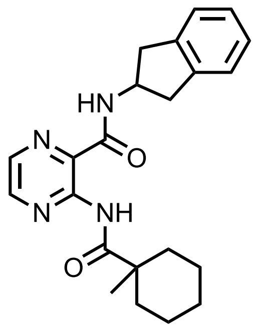

To a solution of 1-methylcyclohexane-1-carboxylic acid (55.2 mg, 388 µmol, 2 eq) in THF (2 mL), successively added a drop of DMF and then oxalyl chloride (25.8 µL, 37.4 mg, 295 µmol, 1.5 eq). Stirred the reaction mixture for 15 min. During this time, separately dissolved **45** (50.1 mg, 197 µmol, 1 eq) in THF (4.6 mL) and added 1.0 M $NaHMDS_{(THF)}$ (982 µL, 982 µmol, 5 eq) to produce a cloudy, yellow suspension. This suspension was added dropwise to the first solution dropwise, resulting in a cloudy orange suspension. The reaction proceed to completion within 15 min as determined by LC-MS. Concentrated the reaction mixture *in vacuo* to remove THF, diluted with DCM (30 mL), washed twice with 1 M $HCl_{(aq)}$ (30 mL), washed twice with water (30 mL), washed once with saturated $NaCl_{(aq)}$ (30 mL), dried over anhydrous Na_2SO_4, filtered, and concentrated *in vacuo*. Purified by normal phase flash column chromatography (hexanes/EtOAc) to obtain **4**.

Yield: 15.1 mg, 20.3%.

^1H NMR (400 MHz, Chloroform-*d*) δ 12.18 (s, 1H), 8.57 (s, 1H), 8.33 (d, *J* = 6.8 Hz, 1H), 8.10 (s, 1H), 7.28 – 7.24 (m, 2H), 7.23 – 7.19 (m, 2H), 4.97 – 4.85 (m, 1H), 3.42 (dd, *J* = 16.1, 7.1 Hz, 2H), 2.96 (dd, *J* = 16.1, 4.3 Hz, 2H), 2.16 (d, *J* = 9.7 Hz, 2H), 1.66 – 1.42 (m, 8H), 1.32 (s, 3H).

^{13}C NMR (101 MHz, $CDCl_3$) δ 176.6, 165.3, 150.1, 146.6, 140.6, 136.1, 129.3, 127.1, 125.0, 50.7, 45.0, 40.1, 35.6, 26.5, 25.9, 23.1.

LC-MS ($C_{22}H_{26}N_4O_2$): Calculated $[M+H]^+$ m/z = 379.21, $[M-H]^-$ m/z = 377.21. Observed $[M+H]^+$ m/z = 379.41, $[M-H]^-$ m/z = 377.25.

N-(2,3-dihydro-1H-inden-2-yl)-3-(3,3-diisopropylureido)pyrazine-2-carboxamide (MAT107, 5):

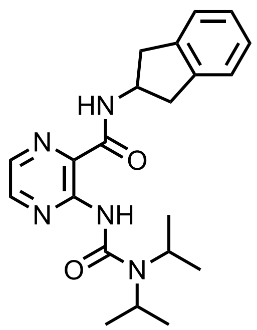

To a solution of **45** (50 mg, 197 μmol, 1 eq) in anhydrous DCM (12 mL), added anhydrous lutidine (114 μL, 983 μmol, 5 eq) and cooled to 0°C. To this, added 15%w/v phosgene$_{(toluene)}$ (196 μL, 275 μmol, 1.4 eq), removed from 0°C bath, and stirred for 1 h. In parallel, charged a second vial with diisopropylamine (200 μL, 143 mg, 1.42 mmol, 7.2 eq) and DCM (2 mL). Added the contents of the phosgene solution to this vial and stirred at room temperature overnight. Concentrated the reaction mixture *in vacuo* to dryness and purified by reverse phase flash column chromatography (water/MeCN; both with 0.1% formic acid) to obtain **5**.

Yield: 38.4 mg, 51.2%. White solid.

^{1}H NMR (400 MHz, DMSO-d_6) δ 10.79 (s, 1H), 9.20 (d, *J* = 7.9 Hz, 1H), 8.49 (d, *J* = 2.3 Hz, 1H), 8.18 (d, *J* = 2.5 Hz, 1H), 7.25 – 7.18 (m, 3H), 7.18 – 7.11 (m, 3H), 4.73 (hept, *J* = 7.3 Hz, 1H), 3.91 (hept, *J* = 6.7 Hz, 2H), 3.25 – 3.12 (m, 3H), 3.05 (dd, *J* = 15.9, 7.3 Hz, 2H), 1.98 (s, 1H), 1.28 (d, *J* = 6.7 Hz, 12H).

^{13}C NMR (101 MHz, DMSO-d_6) δ 166.1, 152.1, 150.9, 146.3, 141.5, 135.5, 130.1, 126.9 (d, *J* = 2.7 Hz), 124.9, 50.9, 46.6, 38.9, 21.3.

LC-MS ($C_{21}H_{27}N_5O_2$): Calculated $[M+H]^+$ m/z = 382.22, $[M-H]^-$ m/z = 380.22. Observed $[M+H]^+$ m/z = 382.45, $[M-H]^-$ m/z = 380.25.

***tert*-butyl ((1*s*,4*s*)-4-((3-((2,3-dihydro-1*H*-inden-2-yl)carbamoyl)pyrazin-2-yl)carbamoyl)cyclohexyl)carbamate (6):**

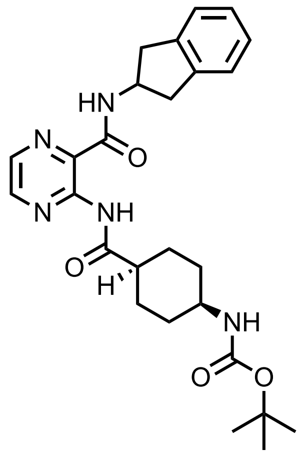

Mixed (1*s*,4*s*)-4-((*tert*-butoxycarbonyl)amino)cyclohexane-1-carboxylic acid (232 mg, 952 μmol, 1.18 eq), *N*-hydroxysuccinimide (111 mg, 966 μmol, 1.20 eq), MeCN (10 mL), and DCM (5 mL) to produce a cloudy, white suspension which was stirred for 5 min. To this, added DCC (197 mg, 955 μmol, 1.18 eq) and stirred overnight. Filtered off the insoluble material and concentrated *in vacuo* to obtain a white solid containing 2,5-dioxopyrrolidin-1-yl (1*s*,4*s*)-4-((*tert*-butoxycarbonyl)amino)cyclohexane-1-carboxylate that was used without further purification or characterization. Dissolved this crude material in THF (4 mL).

Separately dissolved **45** (205 mg, 806 μmol, 1 eq) in THF (10.0 mL) and added 1.0 M $NaHMDS_{(THF)}$ (1.6 mL, 1.60 mmol, 1.98 eq) dropwise to produce a cloudy, yellow suspension that was stirred 2.67 h. Added this solution dropwise to the *N*-hydroxysuccinimide solution and stirred for 2 h. Added more 1.0 M $NaHMDS_{(THF)}$ (1.0 mL, 1.00 mmol, 1.25 eq) and stirred 5 min. Again, added more 1.0 M $NaHMDS_{(THF)}$ (1.0 mL, 1.00 mmol, 1.25 eq) and stirred 5 min. Diluted the reaction mixture with DCM (100 mL), washed twice with 3:1 water / saturated $NH_4Cl_{(aq)}$ (100 mL), dried over anhydrous Na_2SO_4, filtered, and concentrated *in vacuo*. Purified by normal phase flash column chromatography (hexanes/EtOAc) to obtain **6**.

Yield: 217.0 mg, 56.1%. Off-white solid.

^{1}H NMR (400 MHz, Chloroform-*d*) δ 12.09 (s, 1H), 8.57 (d, *J* = 2.3 Hz, 1H), 8.32 (d, *J* = 8.2 Hz, 1H), 8.13 (d, *J* = 2.4 Hz, 1H), 7.30 – 7.25 (m, 2H), 7.24 – 7.19 (m, 2H), 4.88 (dt, *J* = 12.1, 6.1 Hz, 1H), 4.68 (d, *J* = 7.2 Hz, 1H), 3.43 (dd, *J* = 16.2, 7.2 Hz, 2H), 2.97 (dd, *J* = 16.4, 4.4 Hz, 2H), 2.59 (tt, *J* = 8.6, 4.2 Hz, 1H), 1.94 (dt, *J* = 9.0, 4.7 Hz, 2H), 1.88 (p, *J* = 5.1, 4.6 Hz, 2H), 1.81 – 1.68 (m, 5H), 1.44 (s, 9H).

^{13}C NMR (101 MHz, $CDCl_3$) δ 173.8, 165.3, 149.6, 146.6, 140.6, 136.5, 129.2, 127.2, 125.0, 79.3, 77.4, 50.8, 46.5, 44.4, 40.1, 29.8, 28.6, 25.0.

LC-MS ($C_{26}H_{33}N_5O_4$): Calculated $[M+H]^+$ m/z = 480.25, $[M-H]^-$ m/z = 478.25. Observed $[M+H]^+$ m/z = 480.38, $[M-H]^-$ m/z = 478.26.

tert-butyl (1-((3-((2,3-dihydro-1_H_-inden-2-yl)carbamoyl)pyrazin-2-yl)carbamoyl)piperidin-4-yl)carbamate (7):

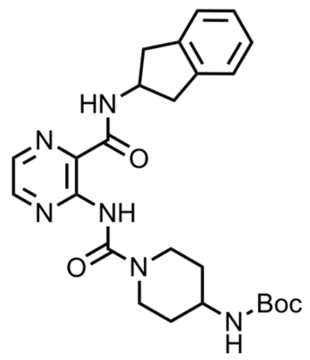

To a solution of **45** (2.00 g, 7.87 mmol, 1 eq) in anhydrous DCM (300 mL), added anhydrous lutidine (3.64 mL, 3.37 g, 31.5 mmol, 4 eq). Cooled this solution to 0°C and then added 15%w/v phosgene$_{(toluene)}$ (7.86 mL, 11.0 mmol, 1.4 eq) slowly along the walls over 5 min. Removed reaction mixture flask from 0°C bath and stirred for 1 h at room temperature. To this, added a solution of tert-butyl piperidin-4-ylcarbamate (3.15 g, 15.7 mmol, 2 eq) in DCM (125 mL) and stirred at room temperature overnight. Quenched reaction mixture with MeOH and concentrated reaction mixture *in vacuo* to dryness. Purified by normal phase flash column chromatography (hexanes/EtOAc) to obtain **7**.

Yield: 776 mg, 20.5%. White solid.

^{1}H NMR (400 MHz, $CDCl_3$) δ 11.49 (s, 1H), 8.52 (d, *J* = 2.2 Hz, 1H), 8.28 (d, *J* = 8.2 Hz, 1H), 8.01 (d, *J* = 2.3 Hz, 1H), 7.31 – 7.24 (m, 2H), 7.24 – 7.18 (m, 2H), 4.93 – 4.79 (m, 1H), 4.46 (s, 1H), 4.23 (d, *J* = 13.6 Hz, 2H), 3.70 (s, 1H), 3.42 (dd, *J* = 16.2, 7.2 Hz, 2H), 3.08 (t, *J* = 12.6 Hz, 2H), 2.96 (dd, *J* = 16.1, 4.9 Hz, 2H), 2.04 (d, *J* = 11.3 Hz, 2H), 1.45 (d, *J* = 7.6 Hz, 9H), 1.39 (dd, *J* = 12.1, 4.2 Hz, 2H).

^{13}C NMR (101 MHz, $CDCl_3$) δ 165.9, 155.2, 152.6, 151.2, 146.8, 140.6, 135.1, 128.7, 127.1, 125.0, 79.7, 50.7, 48.0, 43.3, 40.1, 32.6, 28.6.

LC-MS ($C_{25}H_{32}N_6O_4$): Calculated $[M+H]^+$ m/z = 481.26, $[M-H]^-$ m/z = 479.24. Observed $[M+H]^+$ m/z = 481.39, $[M-H]^-$ m/z = 479.40.

3-(4-aminopiperidine-1-carboxamido)-*N*-(2,3-dihydro-1*H*-inden-2-yl)pyrazine-2-carboxamide (8):

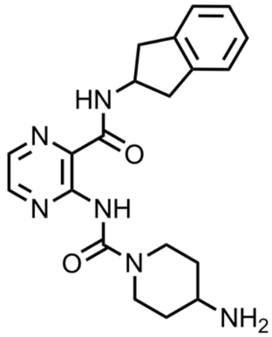

10 mL of 4.0 M $HCl_{(1,4\text{-dioxane})}$ was added to a solution of **7** (2.00 g, 4.16 mmol, 1 eq) in 10 mL methanol and stirred vigorously for 1.75 h before concentrating *in vacuo* to obtain **8** as the dihydrochloride salt without further purification.

Yield: 1.85 g, >95%. White solid.

^{1}H NMR (400 MHz, DMSO) δ 11.04 (s, 1H), 9.21 (d, *J* = 7.8 Hz, 1H), 8.51 (d, *J* = 2.4 Hz, 1H), 8.43 (d, *J* = 5.2 Hz, 3H), 8.23 (d, *J* = 2.4 Hz, 1H), 7.27 – 7.16 (m, 2H), 7.18 – 7.05 (m, 2H), 4.71 (h, *J* = 7.6 Hz, 1H), 4.08 (d, *J* = 13.8 Hz, 2H), 3.28 (tt, *J* = 10.6, 5.0 Hz, 1H), 3.19 (dd, *J* = 15.8, 7.8 Hz, 2H), 3.09 – 2.94 (m, 4H), 2.08 – 1.93 (m, 2H), 1.55 (qd, *J* = 12.3, 4.2 Hz, 2H).

^{13}C NMR (101 MHz, DMSO) δ 165.7, 152.3, 149.8, 145.6, 141.1, 135.7, 130.8, 126.5, 124.5, 50.5, 47.5, 42.1, 38.5, 29.6.

LC-MS ($C_{20}H_{24}N_6O_2$): Calculated $[M+H]^+$ m/z = 381.20, $[M-H]^-$ m/z = 379.19. Observed $[M+H]^+$ m/z = 381.44, $[M-H]^-$ m/z = 379.43.

***tert*-butyl ((1-((3-((2,3-dihydro-1*H*-inden-2-yl)carbamoyl)pyrazin-2-yl)carbamoyl)piperidin-4-yl)methyl)carbamate (9):**

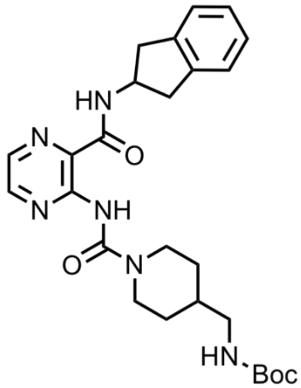

12 mL of 15%w/v $phosgene_{(toluene)}$ (16.8 mmol, 1.4 eq) was added to a solution of **45** (2.99 g, 11.8 mmol, 1 eq) and 2,6-lutidine (6.83 mL, 6.32 g, 59.0 mmol, 5 eq) in 400 mL DCM at 0°C. The reaction mixture was allowed to warm to room temperature while stirring for 1 h and then added to a solution of 4.00 g *tert*-butyl (piperidin-4-ylmethyl)carbamate (18.7 mmol, 1.59 eq) in

100 mL DCM. After vigorously stirring for 20 h, the reaction mixture was quenched with methanol, followed by the addition of granular silica (~15 g) and removal of the solvent under reduced pressure. Purification by normal phase flash column chromatography (hexanes/EtOAc) yielded **9** as a white solid.

Yield: 1.14 g, 19.7%. White solid.

^{1}H NMR (400 MHz, $CDCl_3$) δ 11.46 (s, 1H), 8.52 (d, *J* = 2.3 Hz, 1H), 8.28 (d, *J* = 8.1 Hz, 1H), 8.00 (d, *J* = 2.3 Hz, 1H), 7.30 – 7.24 (m, 2H), 7.24 – 7.15 (m, 2H), 4.92 – 4.80 (m, 1H), 4.65 (t, *J* = 6.1 Hz, 1H), 4.32 (d, *J* = 13.3 Hz, 2H), 3.42 (dd, *J* = 16.2, 7.2 Hz, 2H), 3.05 (t, *J* = 6.3 Hz, 2H), 3.01 – 2.86 (m, 4H), 1.79 (d, *J* = 13.3 Hz, 2H), 1.75 – 1.64 (m, 1H), 1.44 (s, 9H), 1.25 (qd, *J* = 12.2, 4.3 Hz, 2H).

^{13}C NMR (101 MHz, $CDCl_3$) δ 165.9, 156.2, 152.6, 151.2, 146.8, 140.7, 134.9, 128.6, 127.1, 125.0, 79.5, 53.6, 51.0, 50.7, 46.1, 44.3, 41.1, 40.1, 36.9, 29.8, 28.5.

LC-MS ($C_{26}H_{34}N_6O_4$): Calculated $[M+H]^+$ m/z = 495.27, $[M-H]^-$ m/z = 493.26. Observed $[M+H]^+$ m/z = 495.67, $[M-H]^-$ m/z = 493.45.

3-(4-(aminomethyl)piperidine-1-carboxamido)-*N*-(2,3-dihydro-1*H*-inden-2-yl)pyrazine-2-carboxamide (10):

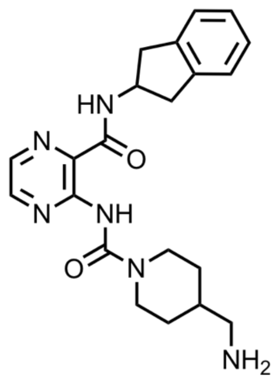

Compound **9** (799 mg, 1.62 mmol, 1 eq) was added to a room temperature solution of 1.0 M HCl in 3:1 methanol / 1,4-dioxane (16 mL) and stirred vigorously for 2 h before concentrating *in vacuo* to yield amine **10** as the dihydrochloride salt without further purification.

Yield: 797.9 mg, 93.2%. White solid.

^{1}H NMR (400 MHz, DMSO) δ 11.01 (s, 1H), 9.22 (d, *J* = 7.8 Hz, 1H), 8.51 (d, *J* = 2.4 Hz, 1H), 8.22 (d, *J* = 2.3 Hz, 1H), 8.03 (s, 3H), 7.27 – 7.19 (m, 2H), 7.19 – 7.09 (m, 2H), 4.71 (h, *J* = 7.5 Hz, 1H), 4.07 (d, *J* = 13.4 Hz, 2H), 3.24 – 3.14 (m, 2H), 3.06 (dd, *J* = 15.8, 7.3 Hz, 2H), 2.91 (t, *J* = 12.7 Hz, 2H), 2.73 (h, *J* = 6.0 Hz, 2H), 1.92 – 1.83 (m, 1H), 1.83 – 1.73 (m, 2H), 1.19 (qd, *J* = 12.3, 4.0 Hz, 2H).

^{13}C NMR (101 MHz, DMSO) δ 165.7, 152.2, 145.0, 145.7, 141.1, 135.5, 130.5, 126.5, 124.5, 50.5, 43.5, 38.4, 33.9, 28.9.

LC-MS ($C_{21}H_{26}N_6O_2$): Calculated $[M+H]^+$ m/z = 395.22, $[M-H]^-$ m/z = 393.20. Observed $[M+H]^+$ m/z = 395.04, $[M-H]^-$ m/z = 393.36.

N-(2,3-dihydro-1H-inden-2-yl)-3-(4-(hydroxymethyl)piperidine-1-carboxamido)pyrazine-2-carboxamide (11):

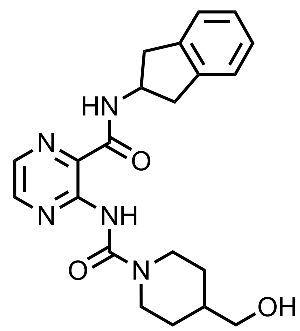

A mixture of **45** (500 mg, 1.97 mmol, 1 eq) and 2,6-lutidine (1.14 mL, 1.05 g, 9.83 mmol, 5 eq) in 80 mL DCM was cooled to 0°C. To this, 1.96 mL 15%w/v $phosgene_{(toluene)}$ (2.75 mmol, 1.4 eq) was added dropwise and then stirred for 15 min at 0°C. The reaction mixture was then added to a solution of 454 mg piperidin-4-ylmethanol (3.94 mmol, 2 eq) in 20 mL DCM. After stirring overnight, the reaction mixture was diluted with DCM, washed twice with 1 M $HCl_{(aq)}$, washed once with saturated $NaCl_{(aq)}$, dried over anhydrous Na_2SO_4, filtered, and concentrated *in vacuo*. The crude product was purified by reverse phase flash column chromatography (water + 0.1% formic acid / MeCN + 0.1% formic acid) to obtain impure product fractions, which were concentrated *in vacuo*. The resulting solid was extracted with MeOH, centrifuged to pellet the insoluble, and decanted. The MeOH-soluble material was concentrated *in vacuo* and then purified by normal phase flash column chromatography (loaded with DCM and eluted with EtOAc → 3:1 EtOAc/EtOH) to obtain **11**.

Yield: 138 mg, 17.7%. White solid.

^{1}H NMR (400 MHz, $CDCl_3$) δ 11.84 (d, 1H), 8.69 (s, 1H), 8.29 (d, *J* = 8.1 Hz, 1H), 8.12 (d, *J* = 2.7 Hz, 1H), 7.30 – 7.26 (m, 1H), 7.26 – 7.19 (m, 3H), 4.87 (dtd, *J* = 12.4, 7.4, 4.8 Hz, 1H), 4.40 – 4.26 (m, 2H), 3.55 (d, *J* = 6.1 Hz, 2H), 3.42 (dd, *J* = 16.1, 7.3 Hz, 4H), 2.98 (td, *J* = 10.3, 4.4 Hz, 4H), 1.86 (d, *J* = 13.7 Hz, 2H), 1.82 – 1.73 (m, 1H), 1.45 – 1.22 (m, 2H).

^{13}C NMR (101 MHz, $CDCl_3$) δ 165.9, 152.6, 151.1, 146.6, 146.5, 140.6, 140.6, 135.1, 134.9, 128.8, 127.1, 125.0, 75.5, 67.6, 50.7, 50.7, 44.3, 43.9, 40.1, 38.9, 35.6, 28.8, 28.4.

LC-MS ($C_{21}H_{25}N_5O_3$): Calculated $[M+H]^+$ m/z = 396.20, $[M-H]^-$ m/z = 394.19. Observed $[M+H]^+$ m/z = 396.38, $[M-H]^-$ m/z = 394.37.

***N*-(2,3-dihydro-1*H*-inden-2-yl)-3-(4-(2-hydroxyethyl)piperidine-1-carboxamido)pyrazine-2-carboxamide (12):**

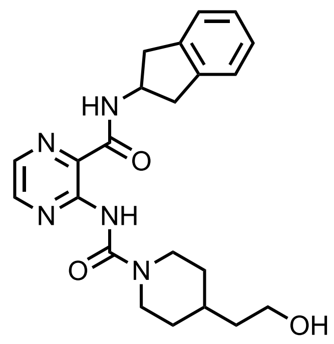

Cooled a mixture of **45** (500 mg, 1.97 mmol, 1 eq), DCM (80 mL), and 2,6-lutidine (1.14 mL, 1.05 g, 9.83 mmol, 5 eq) to 0°C. To this, added 15%w/v $phosgene_{(toluene)}$ (1.96 mL, 2.75 mmol, 1.4 eq) dropwise and then stirred 15 min at 0°C. Added this solution to a flask containing 2-(piperidin-4-yl)ethan-1-ol (508 mg, 3.93 mmol, 2 eq) and DCM (20 mL). After stirring overnight, the reaction mixture was diluted with DCM, washed twice with 1 M $HCl_{(aq)}$, washed once with saturated $NaCl_{(aq)}$, dried over anhydrous Na_2SO_4, filtered, and concentrated *in vacuo*. Purified by reverse phase flash column chromatography (water + 0.1% formic acid / MeCN + 0.1% formic acid) to obtain impure product fractions, which were concentrated *in vacuo*. The resulting solid was extracted with MeOH, centrifuged to pellet the insoluble, and decanted. The MeOH-soluble material was concentrated *in vacuo* and then purified by normal phase flash column chromatography (loaded with DCM and eluted with EtOAc → 3:1 EtOAc/EtOH) to obtain **12**.

Yield: 160 mg, 19.9%. White solid.

^{1}H NMR (400 MHz, $CDCl_3$) δ 11.74 (d, 1H), 8.72 – 8.58 (m, 1H), 8.29 (d, *J* = 8.1 Hz, 1H), 8.12 – 8.06 (m, 1H), 7.30 – 7.26 (m, 1H), 7.26 – 7.24 (m, 1H), 7.24 – 7.17 (m, 2H), 4.93 – 4.81 (m, 1H), 4.27 (d, *J* = 13.4 Hz, 2H), 3.81 (s, 2H), 3.74 (t, *J* = 6.5 Hz, 2H), 3.42 (dd, *J* = 16.2, 7.2 Hz, 2H), 2.96 (dt, *J* = 14.9, 5.3 Hz, 4H), 1.82 (d, *J* = 13.1 Hz, 2H), 1.78 – 1.68 (m, 1H), 1.56 (q, *J* = 6.6 Hz, 2H), 1.29 (qd, *J* = 12.5, 4.2 Hz, 2H).

^{13}C NMR (101 MHz, $CDCl_3$) δ 165.8, 152.5, 151.0, 146.2, 140.6, 134.8, 128.8, 127.1, 125.0, 60.3, 50.7, 44.7, 40.1, 39.2, 32.7, 32.2.

LC-MS ($C_{22}H_{27}N_5O_3$): Calculated $[M+H]^+$ m/z = 410.22, $[M-H]^-$ m/z = 408.20. Observed $[M+H]^+$ m/z = 410.41, $[M-H]^-$ m/z = 408.48.

tert-butyl 4-((3-((2,3-dihydro-1H-inden-2-yl)carbamoyl)pyrazin-2-yl)carbamoyl)piperazine-1-carboxylate (13):

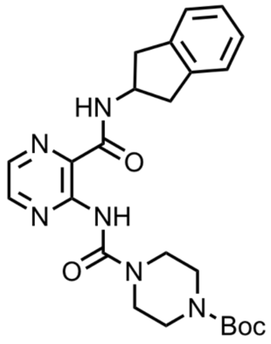

Charged a flask with **45** (500 mg, 197 mmol, 1 eq), DCM (75 mL), and 2,6-lutidine (911 µL, 843 mg, 7.87 mmol, 4 eq) and cooled to 0°C. To this, added 15%w/v $phosgene_{(toluene)}$ (2.00 mL, 2.75 mmol, 1.4 eq), removed reaction from 0°C bath, and let stir at room temperature for 1 h. To the reaction mixture, added *tert*-butyl piperazine-1-carboxylate (394 mg, 3.93 mmol, 2 eq) and stirred 24 h. The reaction mixture was quenched with methanol and concentrated *in vacuo* to dryness. Purified by normal phase flash column chromatography (hexanes/EtOAc) to obtain **13**.

Yield: 156.2 mg, 17.0%. White solid.

^{1}H NMR (400 MHz, $CDCl_3$) δ 11.57 (s, 1H), 8.52 (d, *J* = 2.3 Hz, 1H), 8.30 (d, *J* = 8.1 Hz, 1H), 8.06 – 8.01 (m, 1H), 7.28 (d, *J* = 4.3 Hz, 2H), 7.26 – 7.18 (m, 5H), 4.86 (tq, *J* = 12.6, 6.0 Hz, 1H), 3.67 – 3.60 (m, 4H), 3.53 (t, *J* = 5.1 Hz, 4H), 3.43 (dd, *J* = 16.2, 7.3 Hz, 2H), 2.97 (dd, *J* = 16.2, 4.8 Hz, 2H), 1.48 (s, 9H).

^{13}C NMR (101 MHz, $CDCl_3$) δ 165.9, 154.7, 152.7, 151.1, 146.8, 140.6, 135.3, 128.7, 127.1, 125.0, 80.4, 50.7, 44.0, 40.1, 28.6.

LC-MS ($C_{24}H_{30}N_6O_4$): Calculated $[M+H]^+$ m/z = 467.24, $[M-H]^-$ m/z = 465.23. Observed $[M+H]^+$ m/z = 467.50, $[M-H]^-$ m/z = 465.50.

***N*-(2,3-dihydro-1*H*-inden-2-yl)-3-(piperazine-1-carboxamido)pyrazine-2-carboxamide (14):**

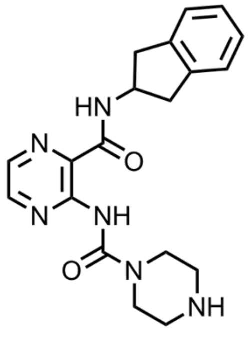

93.0 mg **13** (199 µmol, 1 eq) was dissolved in a solution of 4.0 M $HCl_{(1,4\text{-dioxane})}$ (1.50 mL) in methanol (1.50 mL) and stirred vigorously for 3 h. The reaction mixture was concentrated *in vacuo* to obtain **14** as the dihydrochloride salt without further purification.

Yield: >95%. White solid.

^{1}H NMR (400 MHz, DMSO) δ 11.04 (s, 1H), 9.21 (d, *J* = 7.8 Hz, 1H), 8.50 (d, *J* = 2.4 Hz, 1H), 8.21 (d, *J* = 2.4 Hz, 1H), 7.21 (dt, *J* = 7.3, 3.6 Hz, 2H), 7.19 – 7.10 (m, 2H), 4.72 (h, *J* = 7.5 Hz, 1H), 3.68 – 3.45 (m, 2H), 3.41 (t, *J* = 5.0 Hz, 4H), 3.19 (dd, *J* = 15.8, 7.7 Hz, 2H), 3.05 (dd, *J* = 15.8, 7.2 Hz, 2H), 2.74 (t, *J* = 5.0 Hz, 4H).

^{13}C NMR (101 MHz, DMSO) δ 165.7, 152.3, 150.0, 145.7, 141.1, 135.4, 130.2, 126.4, 124.4, 50.5, 45.4, 44.8, 38.4.

LC-MS ($C_{19}H_{22}N_6O_2$): Calculated $[M+H]^+$ m/z = 367.19, $[M-H]^-$ m/z = 365.17. Observed $[M+H]^+$ m/z = 367.38, $[M-H]^-$ m/z = 365.41.

N-(2,3-dihydro-1*H*-inden-2-yl)-3-(1-hydroxycyclohexane-1-carboxamido)pyrazine-2-carboxamide (15):

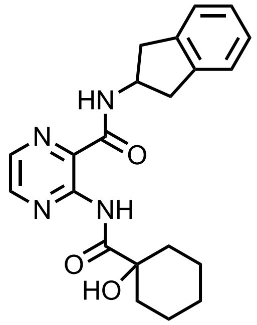

Mixed 1-hydroxycyclohexane-1-carboxylic acid (100 mg, 0.694 mmol), *N*-hydroxysuccinimide (95.4 mg, 829 µmol), and MeCN (3.5 mL). To this, added solution of *N*,*N*'-dicyclohexylcarbodiimide (147 mg, 0.713 mmol) in DCM (1 mL) and observed the formation of a white precipitate. Stirred the reaction mixture for 1.25 h, filtered with cotton to remove the insoluble material, dried over anhydrous Na_2SO_4, filtered with cotton, and concentrated *in vacuo* to obtain crude 1-hydroxycyclohexane-1-carboxylate *N*-hydroxysuccinimide ester, which was used without further purification or characterization.

To a solution of 1-hydroxycyclohexane-1-carboxylate *N*-hydroxysuccinimide ester (50 mg, 0.21 mmol) in DCM (500 µL), successively added chlorotrimethylsilane (TMSCl; 53 µL, 45 mg, 0.42 mmol) and imidazole (57 mg, 0.84 mmol). After 1 h, the reaction was complete as determined by LC-MS and a white precipitate was observed. Filtered the reaction mixture with cotton and concentrated the filtrate *in vacuo* to obtain crude 2,5-dioxopyrrolidin-1-yl 1-((trimethylsilyl)oxy)cyclohexane-1-carboxylate, which was used without further purification or characterization. Resuspended the crude intermediate in anhydrous THF (1.0 mL).

In a separate flask, dissolved **45** (30.0 mg, 0.118 mmol, 1 eq) in THF (3.0 mL) and added 1.0 M $NaHMDS_{(THF)}$ (590 µL, 0.590 mmol) to produce a cloudy, orange suspension. Added the slurry of 2,5-dioxopyrrolidin-1-yl 1-((trimethylsilyl)oxy)cyclohexane-1-carboxylate in THF. After 5 min, reaction was complete as determined by LC-MS. Diluted with DCM (30 mL) and quenched with 1.0 M $HCl_{(aq)}$ (30 mL). Stirred overnight. Discarded $HCl_{(aq)}$ quench and washed organic layer with 1.0 M $HCl_{(aq)}$ (30 mL). Dried over anhydrous Na_2SO_4, filtered, and concentrated *in vacuo*.

To remove the TMS protecting group, dissolved in mixture of MeOH (2.5 mL), DCM (2.5 mL), and acetic acid (20 µL). After stirring for 4 h, the reaction was complete as determined by LC-MS. Diluted the reaction mixture with DCM (30 mL), washed twice with saturated $NaHCO_{3(aq)}$ (30 mL), and washed once with saturated $NaCl_{(aq)}$ (30 mL). Dried over anhydrous Na_2SO_4, filtered, and concentrated *in vacuo*. Purified by normal phase flash column chromatography (hexanes/EtOAc) to obtain **15**.

Yield: 5.1 mg, 11.3%. White solid.

^{1}H NMR (400 MHz, Chloroform-*d*) δ 12.85 (s, 1H), 8.60 (s, 1H), 8.31 (d, *J* = 7.9 Hz, 1H), 8.15 (s, 1H), 7.28 – 7.24 (m, 2H), 7.24 – 7.18 (m, 2H), 4.93 (h, *J* = 7.7 Hz, 1H), 3.42 (dd, *J* = 16.2, 7.2 Hz, 2H), 2.96 (dd, *J* = 16.2, 4.6 Hz, 2H), 2.74 (s, 1H), 2.02 (td, *J* = 13.7, 3.8 Hz, 2H), 1.81 – 1.69 (m, 5H), 1.67 – 1.58 (m, 2H), 1.44 – 1.35 (m, 1H).

^{13}C NMR (101 MHz, $CDCl_3$) δ 175.5, 164.9, 149.3, 146.5, 140.7, 136.7, 129.9, 127.1, 125.0, 75.9, 50.7, 40.2, 34.6, 25.1, 21.4.

LC-MS ($C_{21}H_{24}N_4O_3$): Calculated $[M-H]^-$ m/z = 379.18. Observed $[M-H]^-$ m/z = 379.21.

tert-butyl (1-((3-((2,3-dihydro-1*H*-inden-2-yl)carbamoyl)pyrazin-2-yl)carbamoyl)cyclohexyl)carbamate (16):

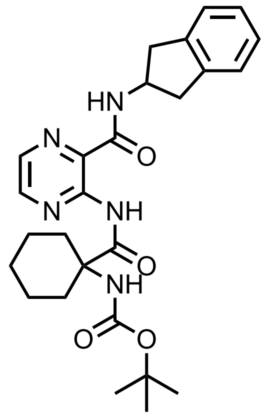

To a solution of 1-((tert-butoxycarbonyl)amino)cyclohexane-1-carboxylic acid (86.1 mg, 354 µmol, 0.9 eq) in 1:1 MeCN/DCM, added *N*-hydroxysuccinimide (40.7 mg, 354 µmol, 0.9 eq) and then a solution of N,N'-dicyclohexylcarbodiimide (73.0 mg, 354 µmol, 0.9 eq) in MeCN (0.71 mL). Stirred 15 min, filtered, concentrated *in vacuo*, and resuspended in DMF (2 mL). Separately combined **45** (100 mg, 393 µmol, 1 eq), DMF (5 mL), and 1.0 M $NaHMDS_{(THF)}$ (0.79 mL, 787 µmol, 2 eq) and added this dropwise to the reaction mixture. After stirring for 15 min, diluted the reaction mixture with EtOAc (100 mL), washed twice with saturated $NH_4Cl_{(aq)}$, dried over anhydrous Na_2SO_4, filtered, and concentrated *in vacuo*. Purified by normal phase flash column chromatography (hexanes/EtOAc) to obtain **16**.

Yield: 50.0 mg, 26.5%.

^{1}H NMR (400 MHz, Chloroform-*d*) δ 12.58 (s, 1H), 8.58 (d, *J* = 2.4 Hz, 1H), 8.29 (d, *J* = 8.3 Hz, 1H), 8.09 (s, 1H), 7.27 (s, 2H), 7.24 – 7.19 (m, 2H), 5.10 (s, 1H), 4.96 – 4.84 (m, 1H), 3.40 (dd,

J = 16.2, 7.3 Hz, 2H), 2.95 (dd, J = 16.0, 5.1 Hz, 2H), 2.07 (s, 2H), 1.99 (td, J = 13.4, 12.9, 3.8 Hz, 2H), 1.79 – 1.64 (m, 3H), 1.44 (d, J = 14.0 Hz, 12H).

^{13}C NMR (101 MHz, $CDCl_3$) δ 173.3, 165.0, 164.7, 154.3, 149.8, 146.5, 145.5, 140.5, 136.0, 129.3, 127.0, 124.9, 50.4, 40.1, 32.0, 28.4, 25.2, 21.4.

LC-MS ($C_{26}H_{33}N_5O_4$): Calculated $[M+H]^+$ m/z = 480.25, $[M-H]^-$ m/z = 478.25. Observed $[M+H]^+$ m/z = 480.44, $[M-H]^-$ m/z = 478.33.

3-(1-aminocyclohexane-1-carboxamido)-*N*-(2,3-dihydro-1*H*-inden-2-yl)pyrazine-2-carboxamide (17):

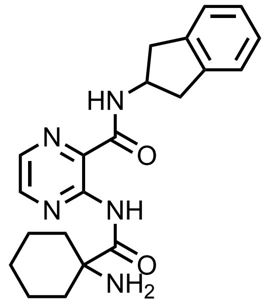

To a solution of **16** (10.0 mg, 20.9 µmol, 1 eq) in DCM (1 mL), added trifluoroacetic acid (TFA; 1 mL) and stirred vigorously for 30 min. Concentrated the reaction mixture *in vacuo* and purified by reverse phase flash column chromatography (water + 0.1% formic acid / MeCN + 0.1% formic acid) to yield **17** as the formic acid salt.

Yield: 8.5 mg, >95%.

^{1}H NMR (400 MHz, DMF-d_7) δ 9.15 (d, J = 7.8 Hz, 1H), 8.60 (d, J = 2.3 Hz, 1H), 8.32 (d, J = 2.3 Hz, 1H), 7.35 – 7.22 (m, 2H), 7.19 (dd, J = 5.5, 3.2 Hz, 2H), 4.86 (h, J = 7.4 Hz, 1H), 3.32 (dd, J = 15.8, 7.6 Hz, 2H), 3.17 (dd, J = 15.7, 6.9 Hz, 2H), 1.94 (dd, J = 12.2, 9.3 Hz, 2H), 1.77 – 1.54 (m, 7H), 1.36 – 1.18 (m, 1H).

^{13}C NMR (101 MHz, DMF-d_7) δ 177.4, 166.2, 163.9, 150.0, 146.9, 142.4, 138.0, 132.4, 127.6, 125.6, 59.6, 52.0, 39.9, 26.5, 22.1.

^{13}C NMR (101 MHz, $CDCl_3$) δ 172.8, 165.3, 149.6, 146.6, 140.5, 136.6, 129.2, 127.2, 125.0, 67.3, 50.8, 44.0, 40.1, 29.0.

LC-MS ($C_{21}H_{25}N_5O_2$): Calculated $[M+H]^+$ m/z = 380.20, $[M-H]^-$ m/z = 378.20. Observed $[M+H]^+$ m/z = 380.40, $[M-H]^-$ m/z = 378.20.

***N*-(2,3-dihydro-1*H*-inden-2-yl)-3-(tetrahydro-2*H*-pyran-4-carboxamido)pyrazine-2-carboxamide (18):**

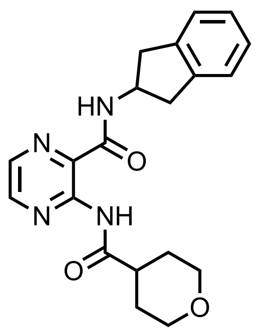

Dissolved **45** (50.0 mg, 197 µmol, 1 eq) in THF (5.6 mL) and added 1.0 M $NaHMDS_{(THF)}$ (983 µL, 983 µmol, 5 eq) to produce a cloudy, yellow suspension. In a second vial, dissolved tetrahydro-2*H*-pyran-4-carboxylic acid (39.0 mg, 300 µmol, 1.5 eq) in THF (1.0 mL) and successively added a drop of DMF and then oxalyl chloride (20.0 µL, 29.0 mg, 228 µmol, 1.15 eq) (note: this mixture is defined as being 1.5 equivalents of tetrahydro-2*H*-pyran-4-carbonyl chloride solution). Added the oxalyl chloride solution dropwise to the **45** vial to produce a cloudy, orange solution. LC-MS analysis of the reaction mixture showed only partial conversion. Added another one equivalent of tetrahydro-2*H*-pyran-4-carbonyl chloride solution that was prepared as described above to the reaction after 40 min. LC-MS analysis showed only a minor increase in yield so after 1.67 h, added 1.0 M $NaHMDS_{(THF)}$ (383 µL, 0.383 mmol, 2 eq) and an additional two equivalents of tetrahydro-2*H*-pyran-4-carbonyl chloride solution. Stirred reaction for 15 h. Then, concentrated the reaction mixture *in vacuo* to remove THF, diluted with EtOAc (30 mL), washed twice with 3:1 water/saturated $NH_4Cl_{(aq)}$ (30 mL), washed twice with water (30 mL), washed once with saturated $NaCl_{(aq)}$ (30 mL), dried over anhydrous Na_2SO_4, filtered, and concentrated *in vacuo*. Purified by normal phase flash column chromatography (1% EtOH in EtOAc). Product fractions were concentrated *in vacuo* and repurified by normal phase flash column chromatography (hexanes/EtOAc) to obtain **18**.

Yield: 9.3 mg, 12.9%.

^{1}H NMR (400 MHz, Chloroform-*d*) δ 12.11 (s, 1H), 8.58 (s, 1H), 8.33 (d, *J* = 8.2 Hz, 1H), 8.14 (s, 1H), 7.29 – 7.26 (m, 2H), 7.24 – 7.19 (m, 2H), 4.94 – 4.82 (m, 1H), 4.07 (d, *J* = 11.5 Hz, 2H), 3.58 – 3.47 (m, 2H), 3.43 (dd, *J* = 16.2, 7.3 Hz, 2H), 2.97 (dd, *J* = 16.2, 4.8 Hz, 2H), 2.70 (p, *J* = 7.8 Hz, 1H), 2.01 – 1.92 (m, 4H).

^{13}C NMR (101 MHz, $CDCl_3$) δ 172.8, 165.3, 149.6, 146.6, 140.5, 136.6, 129.2, 127.2, 125.0, 67.3, 50.8, 44.0, 40.1, 29.0.

LC-MS ($C_{20}H_{22}N_4O_3$): Calculated $[M+H]^+$ m/z = 367.17, $[M-H]^-$ m/z = 365.17. Observed $[M+H]^+$ m/z = 367.38, $[M-H]^-$ m/z = 365.20.

***N*-(2,3-dihydro-1*H*-inden-2-yl)-3-(cis-2,6-dimethylpiperidine-1-carboxamido)pyrazine-2-carboxamide (19):**

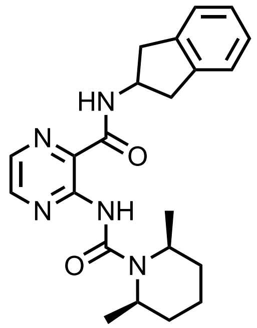

To a solution of **45** (100 mg, 0.393 mmol, 1 eq) in anhydrous DCM (15 mL), added anhydrous lutidine (184 µL, 170 mg, 1.57 mmol, 4 eq) and cooled to 0°C. To this, added 15%w/v phosgene$_{(toluene)}$ (7.9 mL, 11 mmol, 1.4 eq) and stirred 20 min at 0°C. To this, added cis-2,6-dimethylpiperidine (178 mg, 1.57 mmol, 4 eq), warmed to room temperature, and stirred reaction mixture overnight. Concentrated the reaction mixture *in vacuo*, resuspended in DCM (3 mL), filtered off insoluble materials, and concentrated the filtrate *in vacuo*. Purified the crude product by normal phase flash column chromatography (hexanes/EtOAc) to obtain **19**.

Yield: 80.6 mg, 52.0%. White solid.

^{1}H NMR (400 MHz, DMSO-d_6) δ 11.00 (s, 1H), 9.21 (d, *J* = 7.9 Hz, 1H), 8.51 (d, *J* = 2.0 Hz, 1H), 8.21 (d, *J* = 2.2 Hz, 1H), 7.22 (d, *J* = 4.5 Hz, 2H), 7.15 (t, *J* = 4.5 Hz, 2H), 4.72 (q, *J* = 7.6 Hz, 1H), 4.35 (s, 2H), 3.17 (d, *J* = 7.7 Hz, 2H), 3.05 (dd, *J* = 15.7, 7.3 Hz, 2H), 1.79 (dq, *J* = 13.5, 7.0 Hz, 1H), 1.68 – 1.57 (m, 4H), 1.46 (d, *J* = 13.0 Hz, 1H), 1.26 (d, *J* = 6.9 Hz, 6H).

^{1}H NMR (400 MHz, Chloroform-*d*) δ 11.38 (s, 1H), 8.50 (t, *J* = 1.7 Hz, 1H), 8.28 (d, *J* = 8.3 Hz, 1H), 7.97 (t, *J* = 1.7 Hz, 1H), 7.25 (q, *J* = 4.1 Hz, 2H), 7.22 – 7.16 (m, 2H), 4.87 (ddt, *J* = 12.8, 7.9, 4.2 Hz, 1H), 4.53 (p, *J* = 6.7 Hz, 2H), 3.40 (dd, *J* = 16.1, 7.3 Hz, 2H), 2.94 (dd, *J* = 16.2, 5.0 Hz, 2H), 1.91 – 1.75 (m, 1H), 1.77 – 1.61 (m, 4H), 1.52 (dq, *J* = 12.3, 3.7 Hz, 1H), 1.36 (d, *J* = 7.0 Hz, 6H).

^{13}C NMR (101 MHz, $CDCl_3$) δ 165.8, 153.0, 151.4, 146.6, 140.7, 134.6, 128.8, 127.0, 125.0, 50.5, 46.1, 40.1, 30.4, 21.1, 13.9.

LC-MS ($C_{22}H_{27}N_5O_2$): Calculated $[M+H]^+$ m/z = 394.22, $[M-H]^-$ m/z = 392.22. Observed $[M+H]^+$ m/z = 394.48, $[M-H]^-$ m/z = 392.35.

3-(cyclobutanecarboxamido)-*N*-(2,3-dihydro-1*H*-inden-2-yl)pyrazine-2-carboxamide (20):

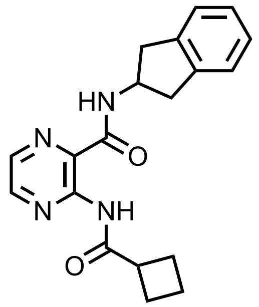

Combined **45** (100 mg, 0.20 mmol, 1 eq), THF (10 mL), and 1.0 M $NaHMDS_{(THF)}$ (1.97 mL, 1.97 mmol, 5 eq) and stirred vigorously for 5 min. The reaction mix was added to a solution of cyclobutanecarbonyl chloride (93.2 mg, 0.787 mmol, 2 eq) in THF (5 mL) and stirred vigorously. After 2 h, diluted reaction mixture with EtOAc, washed twice with 1:1 water / saturated $NH_4Cl_{(aq)}$, dried over anhydrous Na_2SO_4, filtered, and concentrated *in vacuo*. Purified by reverse phase flash column chromatography (water + 0.1% formic acid / MeCN + 0.1% formic acid) and then further purified by normal phase flash column chromatography (hexanes/EtOAc) to obtain **20**.

Yield: 35.7 mg, 27.0%.

^{1}H NMR (400 MHz, Chloroform-*d*) δ 11.92 (s, 1H), 8.60 (s, 1H), 8.35 (d, *J* = 8.1 Hz, 1H), 8.15 (s, 1H), 7.30 (q, *J* = 4.1 Hz, 2H), 7.28 – 7.22 (m, 2H), 4.91 (tq, *J* = 7.5, 4.8, 3.8 Hz, 1H), 3.48 (d, *J* = 7.3 Hz, 1H), 3.46 – 3.37 (m, 2H), 3.01 (dd, *J* = 16.2, 4.8 Hz, 2H), 2.49 (dq, *J* = 11.7, 9.1 Hz, 2H), 2.35 (qd, *J* = 8.8, 4.4 Hz, 2H), 2.03 (dddd, *J* = 25.1, 20.5, 12.4, 7.9 Hz, 2H).

^{13}C NMR (101 MHz, $CDCl_3$) δ 173.6, 165.2, 149.5, 146.5, 140.5, 136.2, 129.0, 127.0, 124.9, 50.7, 41.9, 40.0, 25.3, 18.1.

LC-MS ($C_{19}H_{20}N_4O_2$): Calculated $[M+H]^+$ m/z = 337.16, $[M-H]^-$ m/z = 335.16. Observed $[M+H]^+$ m/z = 337.39, $[M-H]^-$ m/z = 335.20.

3-benzamido-*N*-(2,3-dihydro-1*H*-inden-2-yl)pyrazine-2-carboxamide (21):

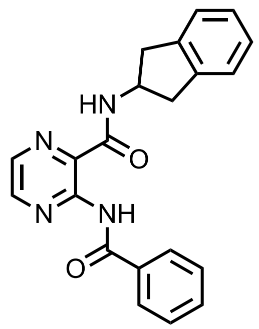

Under argon atmosphere, dissolved **45** (20 mg, 78.7 μmol; 1 eq) in anhydrous THF (2.0 mL) and added 1 M $NaHMDS_{(THF)}$ (0.20 mL, 197 μmol, 2.5 eq). Separately dissolved benzoyl chloride (18.3 μL, 22.2 mg, 157 μmol, 2 eq) and THF (0.6 mL). Added the benzoyl chloride solution dropwise to the suspension of **45** and stirred overnight. Concentrated reaction mixture

in vacuo to dryness, diluted with EtOAc (30 mL), washed twice with 3:1 water / saturated $NH_4Cl_{(aq)}$ (30 mL), washed twice with water (30 mL), washed once with saturated $NaCl_{(aq)}$, dried over anhydrous Na_2SO_4, filtered, and concentrated *in vacuo*. Purified by normal phase flash column chromatography (1% EtOH in EtOAc) and then repurified by normal phase flash column chromatography (hexanes/EtOAc) to obtain **21**.

Yield: 12.9 mg, 45.7%.

^{1}H NMR (400 MHz, Chloroform-*d*) δ 12.94 (s, 1H), 8.64 (s, 1H), 8.37 (d, *J* = 8.0 Hz, 1H), 8.16 (s, 1H), 8.12 (d, *J* = 7.2 Hz, 2H), 7.60 – 7.50 (m, 3H), 7.28 – 7.24 (m, 2H), 7.23 – 7.19 (m, 2H), 4.99 – 4.84 (m, 1H), 3.44 (dd, *J* = 16.3, 7.0 Hz, 2H), 2.99 (dd, *J* = 16.3, 4.7 Hz, 2H).

^{13}C NMR (101 MHz, $CDCl_3$) δ 165.5, 164.8, 145.0, 146.7, 140.6, 136.6, 134.5, 132.5, 129.5, 129.0, 127.9, 127.1, 125.0, 50.8, 40.1.

LC-MS ($C_{21}H_{18}N_4O_2$): Calculated $[M+H]^+$ m/z = 359.14, $[M-H]^-$ m/z = 357.14. Observed $[M+H]^+$ m/z = 359.34, $[M-H]^-$ m/z = 357.14.

5-((3-((2,3-dihydro-1*H*-inden-2-yl)carbamoyl)pyrazin-2-yl)amino)-5-oxopentanoic acid (22):

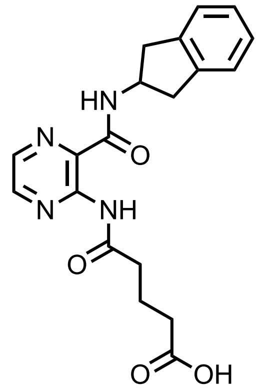

Combined **45** (50 mg, 0.20 mmol, 1 eq), glutaric anhydride (22 mg, 0.20 mmol, 1 eq), DCM (5 mL), and 1.0 M $NaHMDS_{(THF)}$ (0.6 mL, 0.59 mmol, 3 eq). After 2 h, diluted reaction mixture with DCM, washed with 1 M $HCl_{(aq)}$, washed with saturated $NaCl_{(aq)}$, dried over anhydrous Na_2SO_4, filtered, and concentrated *in vacuo*. Purified by ion exchange chromatography on a 500 mg Biotage Evolute AX column (successively pre-washed with MeOH, water, and then 5% $NH_4OH_{(aq)}$; loaded in 5% $NH_4OH_{(aq)}$; successively washed with water, MeOH, DCM, and then again with MeOH; and eluted with 0.25->0.5% formic $acid_{(MeOH)}$). Concentrated *in vacuo* to obtain **22**.

Yield: 5.8 mg, 8%. Brown solid.

^{1}H NMR (400 MHz, Methanol-*d*$_4$) δ 8.47 (s, 1H), 8.30 (s, 1H), 7.26 – 7.19 (m, 2H), 7.15 (dd, *J* = 5.5, 3.3 Hz, 2H), 4.81 (q, *J* = 7.0 Hz, 1H), 3.66 (s, 1H), 3.35 (d, *J* = 7.6 Hz, 1H), 3.04 (dd, *J* = 15.8, 6.6 Hz, 2H), 2.69 (t, *J* = 7.3 Hz, 2H), 2.44 (t, *J* = 7.3 Hz, 2H), 2.39 (t, *J* = 7.3 Hz, 1H), 2.34 (t, *J* = 7.2 Hz, 1H), 2.03 (p, *J* = 7.4 Hz, 2H), 1.88 (p, *J* = 7.3 Hz, 1H).

^{13}C NMR (101 MHz, MeOD) δ 175.3, 173.7, 166.9, 149.5, 146.6, 142.1, 138.7, 132.1, 127.8, 125.6, 52.2, 52.0, 40.1, 38.1, 34.1, 33.9, 21.5, 21.4.

LC-MS ($C_{19}H_{20}N_4O_4$): Calculated $[M+H]^+$ m/z = 369.15, $[M-H]^-$ m/z = 367.15. Observed $[M+H]^+$ m/z = 369.30, $[M-H]^-$ m/z = 367.15.

_N_-(2,3-dihydro-1_H_-inden-2-yl)-3-ureidopyrazine-2-carboxamide (23):

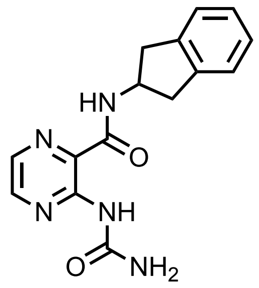

To a solution of **45** (20.0 mg, 78.7 µmol, 1 eq) in anhydrous DCM (2 mL), added anhydrous lutidine (37 µL, 34 mg, 0.32 mmol, 4 eq) and cooled to 0°C. Added 15%w/v $phosgene_{(toluene)}$ (63 µL, 0.94 mmol, 1.2 eq) dropwise and then stirred 10 min at 0°C. Added *N*-hydroxysuccinimide (30 mg, 0.26 mmol) and stirred overnight. Diluted the reaction mixture with DCM (30 mL), washed twice with 1:1 water / saturated $NH_4Cl_{(aq)}$, washed twice with 1:1 water / saturated $NaHCO_{3(aq)}$, washed once with saturated $NaCl_{(aq)}$, dried over anhydrous Na_2SO_4, filtered, and concentrated *in vacuo*. Triturated with MeCN, filtered, and successively washed with MeCN and then DCM to obtain **23**.

Yield: 6.1 mg, 26%. White solid.

^{1}H NMR (400 MHz, DMSO-d_6) δ 10.89 (s, 1H), 9.27 (d, *J* = 7.9 Hz, 1H), 8.48 (s, 1H), 8.29 (s, 1H), 8.25 (s, 1H), 7.33 (s, 1H), 7.22 (d, *J* = 4.4 Hz, 2H), 7.16 (d, *J* = 4.4 Hz, 2H), 4.73 (h, *J* = 7.6 Hz, 1H), 3.20 (dd, *J* = 15.7, 7.7 Hz, 2H), 3.05 (dd, *J* = 15.7, 7.4 Hz, 2H).

^{13}C NMR (101 MHz, DMSO-d_6) δ 165.0, 153.9, 149.1, 144.4, 141.0, 134.9, 128.5, 126.5, 124.4, 50.5, 38.3.

LC-MS ($C_{15}H_{15}N_5O_2$): Calculated $[M+H]^+$ m/z = 298.12, $[M-H]^-$ m/z = 296.12. Observed $[M+H]^+$ m/z = 298.38, $[M-H]^-$ m/z = 296.19.

perfluorophenyl 6-((tert-butoxycarbonyl)amino)hexanoate (51):

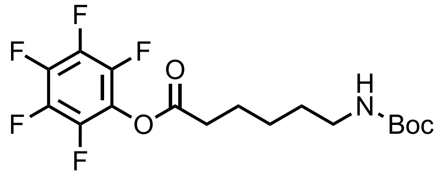

Dissolved 6-((tert-butoxycarbonyl)amino)hexanoic acid (500 mg, 2.16 mmol, 1 eq) in DMF (4 mL) and added bis(perfluorophenyl) carbonate (1.02 g, 2.59 mmol, 1.2 eq) and DIPEA

(1.51 mL, 1.12 g, 8.65 mmol, 4 eq). Stirred reaction for 25 min. Then, diluted the reaction mixture with EtOAc (150 mL), washed twice with 0.2 M $HCl_{(aq)}$ (100 mL), washed three times with 4:1 mixture of water and saturated $NaHCO_{3(aq)}$ (100 mL), washed once with 3:1 mixture of water and saturated $NaCl_{(aq)}$ (100 mL), dried over anhydrous Na_2SO_4, filtered, and concentrated *in vacuo* to obtain **51**.

Yield: 424 mg, 49.3%. White solid.

MS and NMR data matched that from Pourcelle et al. [1]

tert-butyl (6-((1-((3-((2,3-dihydro-1_H_-inden-2-yl)carbamoyl)pyrazin-2-yl)carbamoyl)piperidin-4-yl)amino)-6-oxohexyl)carbamate (41):

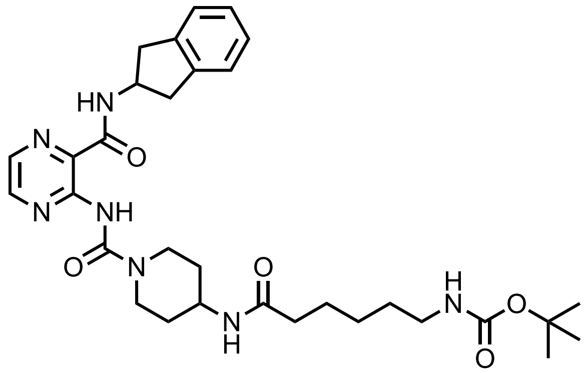

To a stirred solution of **8·2HCl** (213 mg, 470 µmol, 1 eq), DCM (5 mL), and DIPEA (307 µL, 228 mg, 1.76 mmol, 3.47 eq), added a solution of **51** (214.5 mg, 540 µmol, 1.15 eq) in DCM (5 mL) and stirred 4 h. Diluted reaction mixture with EtOAc (300 mL) + MeOH (3 mL), washed twice with 1:1 mixture of water and saturated $NaHCO_{3(aq)}$ (200 mL), washed twice with 0.2 M $HCl_{(aq)}$ (50 mL), and washed once with 3:1 mixture of water and saturated $NaCl_{(aq)}$ (100 mL). Dried over anhydrous Na_2SO_4, filtered, and concentrated *in vacuo*. Purified by reverse phase flash column chromatography (water + 0.1% formic acid / MeCN + 0.1% formic acid) to obtain **41**.

Yield: 254 mg, 88.9%. White solid.

^{1}H NMR (400 MHz, $CDCl_3$) δ 11.51 (s, 1H), 8.49 (d, *J* = 2.4 Hz, 1H), 8.28 (d, *J* = 8.2 Hz, 1H), 8.02 (d, *J* = 2.4 Hz, 1H), 7.29 – 7.16 (m, 4H), 5.63 (d, *J* = 8.0 Hz, 1H), 4.85 (dtd, *J* = 12.3, 7.4, 4.9 Hz, 1H), 4.56 (s, 1H), 4.25 (d, *J* = 13.6 Hz, 2H), 4.03 (dtt, *J* = 11.6, 7.9, 3.9 Hz, 1H), 3.41 (dd, *J* = 16.1, 7.2 Hz, 2H), 3.14 – 3.02 (m, 4H), 2.95 (dd, *J* = 16.1, 4.8 Hz, 2H), 2.17 (t, *J* = 7.6 Hz, 2H), 2.02 (dd, *J* = 13.2, 3.9 Hz, 2H), 1.65 (p, *J* = 7.6 Hz, 2H), 1.55 – 1.25 (m, 15H).

^{13}C NMR (101 MHz, $CDCl_3$) δ 172.7, 165.7, 156.2, 152.6, 150.9, 146.2, 140.6, 135.1, 128.9, 127.1, 125.0, 79.3, 50.7, 46.7, 43.4, 40.6, 40.1, 36.5, 32.1, 29.8, 28.6, 26.4, 25.4.

LC-MS ($C_{31}H_{43}N_7O_5$): Calculated $[M+H]^+$ m/z = 594.73, $[M-H]^-$ m/z = 592.73. Observed $[M+H]^+$ m/z = 594.66, $[M-H]^-$ m/z = 592.56.

3-(4-(6-aminohexanamido)piperidine-1-carboxamido)-*N*-(2,3-dihydro-1*H*-inden-2-yl)pyrazine-2-carboxamide (42):

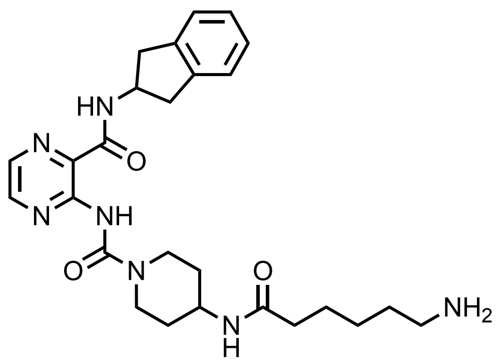

To a vigorously stirred solution of MeOH (6 mL) at 0°C, added $SOCl_2$ (500 µL, 815 mg, 6.85 mmol, 35.5 eq) dropwise over 5 min and stirred for 20 min. Added the entire MeOH/$SOCl_2$ solution to dry **41** (115 mg, 193 µmol, 1 eq) and stirred for 45 min. Concentrated reaction mixture *in vacuo* to a dry, white solid to obtain **42** as the dihydrochloride salt.

Yield: 108.3 mg, >95%. White solid.

^{1}H NMR (400 MHz, MeOD) δ 8.43 (s, 2H), 7.29 – 7.11 (m, 4H), 4.85 (t, *J* = 6.6 Hz, 1H), 4.18 (d, *J* = 13.0 Hz, 2H), 3.99 (s, 1H), 3.39 – 3.32 (m, 2H), 3.22 (t, *J* = 12.1 Hz, 2H), 3.07 (dd, *J* = 15.8, 6.4 Hz, 2H), 2.93 (t, *J* = 7.2 Hz, 2H), 2.25 (t, *J* = 7.1 Hz, 2H), 1.99 (d, *J* = 11.4 Hz, 2H), 1.78 – 1.62 (m, 4H), 1.62 – 1.48 (m, 2H), 1.43 (q, *J* = 7.5 Hz, 2H).

^{13}C NMR (101 MHz, MeOD) δ 175.3, 166.2, 154.0, 148.7, 141.9, 139.4, 137.0, 134.3, 127.9, 125.6, 52.4, 47.6, 44.3, 40.6, 39.9, 36.6, 32.4, 28.2, 26.9, 26.3.

LC-MS ($C_{26}H_{35}N_7O_3$): Calculated $[M+H]^+$ m/z = 494.29, $[M-H]^-$ m/z = 492.27. Observed $[M+H]^+$ m/z = 494.48, $[M-H]^-$ m/z = 492.44.

4-(3-(6-((1-((3-((2,3-dihydro-1*H*-inden-2-yl)carbamoyl)pyrazin-2-yl)carbamoyl)piperidin-4-yl)amino)-6-oxohexyl)thioureido)-2-(6-hydroxy-3-oxo-3*H*-xanthen-9-yl)benzoic acid

5-(3-(6-((1-((3-((2,3-dihydro-1*H*-inden-2-yl)carbamoyl)pyrazin-2-yl)carbamoyl)piperidin-4-yl)amino)-6-oxohexyl)thioureido)-2-(6-hydroxy-3-oxo-3*H*-xanthen-9-yl)benzoic acid (MAT379, 24):

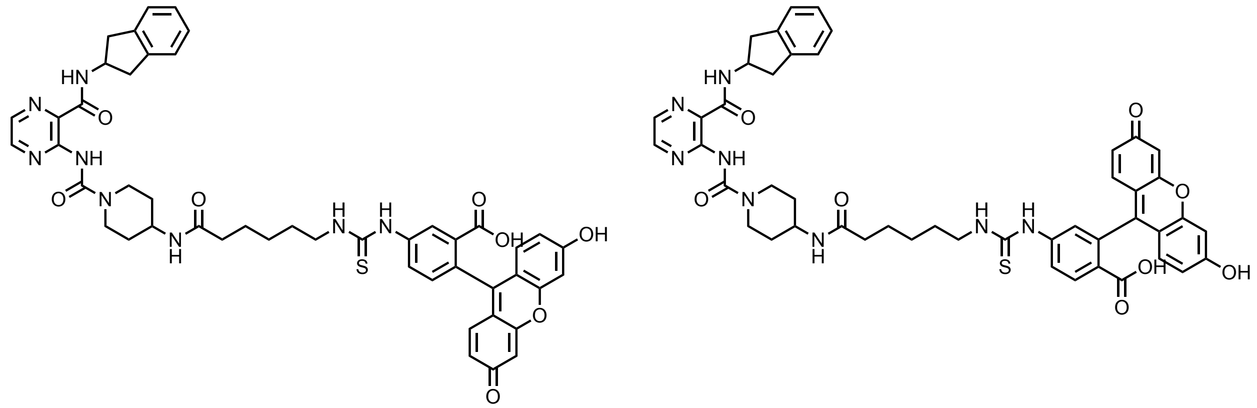

Combined **42·2HCl** (21.6 mg, 40.7 µmol, 1 eq), DMF (1 mL), and DIPEA (30 µL, 22 mg, 0.17 mmol, 4.2 eq). To this, added fluorescein 5/6-isothiocyanate (20.0 mg, 51.4 µmol, 1.25 eq) and stirred for 20 h. The reaction mixture was directly purified by reverse phase flash column chromatography (water + 0.1% formic acid / MeCN + 0.1% formic acid) to obtain MAT379.

Yield: 12.3 mg, 34.2%. Orange solid.

^{1}H NMR (400 MHz, DMSO) δ 11.00 (s, 1H), 10.13 (s, 2H), 9.98 (s, 1H), 9.21 (d, *J* = 7.7 Hz, 1H), 8.50 (d, *J* = 2.7 Hz, 1H), 8.25 (s, 1H), 8.22 (d, *J* = 2.6 Hz, 1H), 8.14 (s, 1H), 7.83 (d, *J* = 7.8 Hz, 1H), 7.80 – 7.65 (m, 1H), 7.27 – 7.05 (m, 5H), 6.67 (d, *J* = 2.6 Hz, 2H), 6.64 – 6.52 (m, 4H), 4.80 – 4.62 (m, 1H), 3.97 (d, *J* = 13.3 Hz, 2H), 3.88 – 3.72 (m, 1H), 3.49 (s, 1H), 3.19 (dd, *J* = 15.8, 7.6 Hz, 2H), 3.05 (dd, *J* = 15.7, 6.9 Hz, 4H), 2.14 – 2.01 (m, 1H), 1.87 – 1.69 (m, 2H), 1.63 – 1.45 (m, 4H), 1.45 – 1.15 (m, 6H).

^{13}C NMR (101 MHz, DMSO) δ 171.3, 168.6, 165.7, 163.1, 159.5, 152.2, 151.9, 145.0, 145.7, 141.4, 141.1, 135.5, 130.5, 129.1, 126.5, 124.5, 124.0, 112.6, 109.7, 102.2, 83.0, 50.4, 45.5, 43.7, 42.8, 38.4, 35.4, 31.5, 28.2, 26.1, 25.1.

LC-MS ($C_{47}H_{46}N_8O_8S$): Calculated $[M+H]^+$ m/z = 883.32, $[M-H]^-$ m/z = 881.31. Observed $[M+H]^+$ m/z = 883.74, $[M-H]^-$ m/z = 881.61.

tert-butyl (6-(((1-((3-((2,3-dihydro-1_H_-inden-2-yl)carbamoyl)pyrazin-2-yl)carbamoyl)piperidin-4-yl)methyl)amino)-6-oxohexyl)carbamate (43):

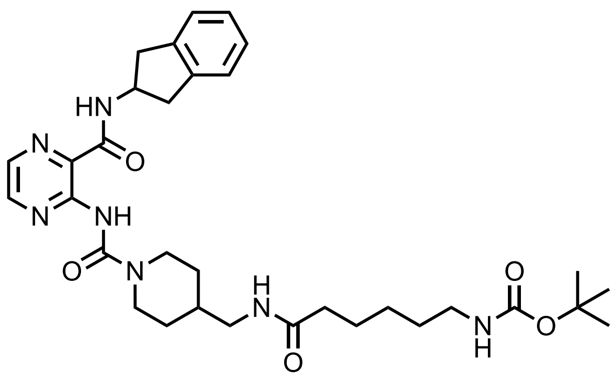

Charged a vial with **10·2HCl** (204 mg, 437 μmol, 1 eq), DCM (5 mL), and DIPEA (298 μL, 221 mg, 1.71 mmol, 3.92 eq) and stirred vigorously. Over the course of 1 min, slowly added a solution of **51** (215 mg, 540 μmol, 1.24 eq) and DCM (5 mL). After stirring for 3 h, diluted reaction mixture with EtOAc (300 mL) and MeOH (3 mL), washed twice with 1:1 water / saturated $NaHCO_{3(aq)}$ (200 mL), washed twice with 0.2 M $HCl_{(aq)}$ (50 mL), washed once with 3:1 water / saturated $NaCl_{(aq)}$ (100 mL), dried over anhydrous Na_2SO_4, filtered, and concentrated *in vacuo*. Purified by reverse phase flash column chromatography (water + 0.1% formic acid / MeCN + 0.1% formic acid) to obtain **43**.

Yield: 247 mg, 93%. Light yellow solid.

^{1}H NMR (400 MHz, $CDCl_3$) δ 11.45 (s, 1H), 8.49 (d, J = 2.3 Hz, 1H), 8.28 (d, J = 8.1 Hz, 1H), 7.99 (d, J = 2.3 Hz, 1H), 7.29 – 7.16 (m, 4H), 5.79 (s, 1H), 4.85 (dtd, J = 12.3, 7.5, 4.9 Hz, 1H), 4.58 (s, 1H), 4.30 (d, J = 13.3 Hz, 2H), 3.41 (dd, J = 16.2, 7.2 Hz, 2H), 3.17 (t, J = 6.1 Hz, 2H), 3.09 (t, J = 7.1 Hz, 2H), 3.01 – 2.81 (m, 4H), 2.18 (t, J = 7.6 Hz, 2H), 1.85 – 1.72 (m, 3H), 1.65 (p, J = 7.6 Hz, 2H), 1.53 – 1.18 (m, 15H).

^{13}C NMR (101 MHz, $CDCl_3$) δ 173.4, 165.8, 156.2, 152.6, 151.1, 146.5, 140.6, 134.9, 128.7, 127.1, 125.0, 79.3, 50.7, 44.9, 44.3, 40.5, 40.1, 36.6, 36.4, 29.9, 28.6, 26.5, 25.5.

LC-MS ($C_{32}H_{45}N_7O_5$): Calculated $[M+H]^+$ m/z = 608.76, $[M-H]^-$ m/z = 606.76. Observed $[M+H]^+$ m/z = 608.37, $[M-H]^-$ m/z = 606.49.

3-(4-((6-aminohexanamido)methyl)piperidine-1-carboxamido)-*N*-(2,3-dihydro-1*H*-inden-2-yl)pyrazine-2-carboxamide (44):

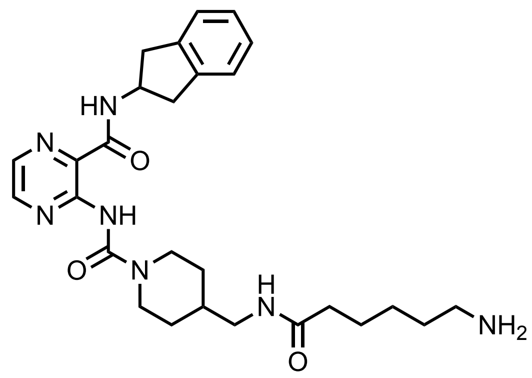

To a vigorously stirred solution of MeOH (6 mL) at 0°C, added $SOCl_2$ (500 µL, 815 mg, 6.85 mmol, 49.2 eq) dropwise over 5 min and stirred for 20 min. Added the entire MeOH/$SOCl_2$ solution to dry **43** (84.6 mg, 139 µmol, 1 eq) and stirred for 45 min. Concentrated the reaction mixture *in vacuo* to obtain **44** as the dihydrochloride salt.

Yield: 69.1 mg, 85.5%. Light yellow solid.

^{1}H NMR (400 MHz, MeOD) δ 8.50 (s, 1H), 8.41 (s, 1H), 7.28 – 7.10 (m, 4H), 4.86 (d, *J* = 9.1 Hz, 1H), 4.23 (s, 2H), 3.40 – 3.32 (m, 2H), 3.19 – 3.04 (m, 6H), 2.92 (d, *J* = 7.4 Hz, 2H), 2.27 (d, *J* = 7.1 Hz, 2H), 1.87 (d, *J* = 11.1 Hz, 3H), 1.68 (q, *J* = 7.8 Hz, 4H), 1.43 (t, *J* = 7.4 Hz, 2H), 1.37 – 1.18 (m, 2H).

^{13}C NMR (101 MHz, MeOD) δ 176.2, 165.8, 153.9, 147.9, 141.9, 136.8, 135.4, 127.9, 127.8, 125.6, 52.4, 45.5, 40.6, 39.8 (s, 2C), 37.2, 36.6, 30.7, 28.2, 27.0, 26.4.

LC-MS ($C_{27}H_{39}Cl_2N_7O_3$): Calculated $[M+H]^+$ m/z = 581.56, $[M-H]^-$ m/z = 579.56. Observed $[M+H]^+$ m/z = 508.47, $[M-H]^-$ m/z = 506.46.

5-(3-(6-(((1-((3-((2,3-dihydro-1*H*-inden-2-yl)carbamoyl)pyrazin-2-yl)carbamoyl)piperidin-4-yl)methyl)amino)-6-oxohexyl)thioureido)-2-(6-hydroxy-3-oxo-3*H*-xanthen-9-yl)benzoic acid

4-(3-(6-(((1-((3-((2,3-dihydro-1*H*-inden-2-yl)carbamoyl)pyrazin-2-yl)carbamoyl)piperidin-4-yl)methyl)amino)-6-oxohexyl)thioureido)-2-(6-hydroxy-3-oxo-3*H*-xanthen-9-yl)benzoic acid

MAT425:

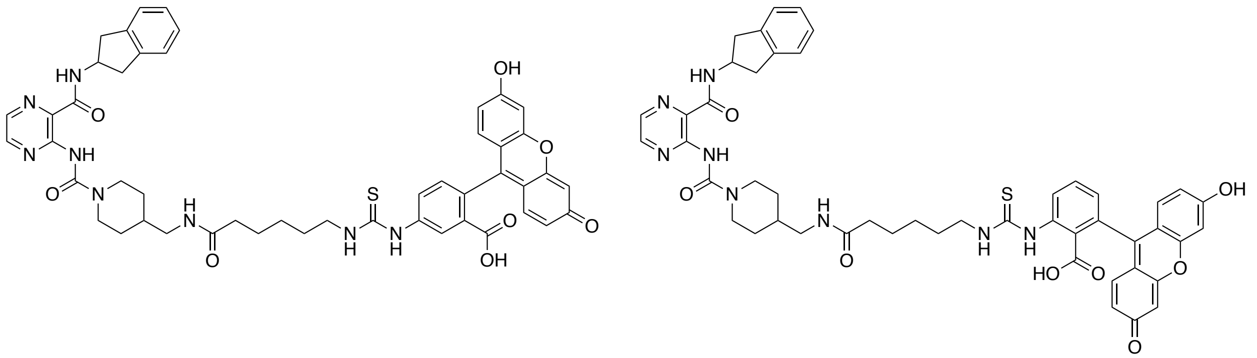

Combined **44·2HCl** (5.60 mg, 9.65 µmol, 1 eq), DIPEA (8.40 µL, 6.23 mg, 48.2 µmol, 5 eq), and DMSO (250 µL) and stirred 1 min. To this, added a solution of 5/6-FITC (3.76 mg, 9.65 µmol, 1 eq) in DMSO (250 µL) and stirred vigorously. Monitored by LCMS until reaction was complete. Purified reaction mixture directly by reverse phase flash column chromatography (water + 0.1% formic acid / MeCN + 0.1% formic acid) to obtain MAT425.

Yield: 3.2 mg, 37%. Yellow solid.

^{1}H NMR (400 MHz, DMSO) δ 11.00 (s, 1H), 10.23 (s, 2H), 9.20 (d, *J* = 7.8 Hz, 1H), 8.49 (d, *J* = 2.4 Hz, 1H), 8.41 (s, 1H), 8.31 – 8.17 (m, 2H), 7.86 (t, *J* = 5.9 Hz, 1H), 7.75 (d, *J* = 8.2 Hz, 1H), 7.27 – 7.18 (m, 2H), 7.18 – 7.11 (m, 2H), 6.68 – 6.52 (m, 5H), 4.72 (dt, *J* = 15.1, 8.7 Hz, 1H), 4.05 (d, *J* = 13.7 Hz, 2H), 3.51 – 3.42 (m, 2H), 3.19 (dd, *J* = 15.7, 7.5 Hz, 2H), 3.11 – 3.00 (m, 2H), 2.97 (t, *J* = 6.0 Hz, 1H), 2.92 – 2.76 (m, 2H), 2.25 (s, 1H), 2.14 – 2.04 (m, 2H), 1.68 (d, *J* = 12.5 Hz, 2H), 1.61 – 1.48 (m, 4H), 1.40 – 1.04 (m, 12H), 0.84 (d, *J* = 6.2 Hz, 1H).

LC-MS ($C_{48}H_{48}N_8O_8S$): Calculated $[M+H]^+$ m/z = 897.34, $[M-H]^-$ m/z = 895.32. Observed $[M+H]^+$ m/z = 897.64, $[M-H]^-$ m/z = 895.54.

Sulfamoyl chloride (52):

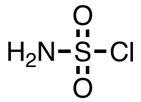

Synthesized as described by Kato et al.,[2] except that the benzene used for precipitation was replaced by toluene. In short, a vial was charged with chlorosulfonyl isocyanate (3.00 mL, 4.89 g, 34.6 mmol, 1.02 eq), flushed with argon, and cooled to 0°C. Added formic acid (1.27 mL, 1.55 g, 33.7 mmol, 1.00 eq) dropwise. Reaction bubbled vigorously before solidifying within 1 min. After 1 h, added toluene (12 mL) and stirred at room temperature overnight. Removed insoluble material by filtration and concentrated *in vacuo* to obtain **52**.

Yield: 3.54 g, 88.6%. White solid.

^{1}H NMR (400 MHz, $CDCl_3$) δ 6.07 (s, 2H).

^{13}C NMR – *does not contain any carbons*.

LC-MS (ClH_2NO_2S): Calculated $[M+H]^+$ m/z = 116.53, $[M+H]^-$ m/z = 114.53. Did not observe by LC-MS. Product of the hydrolysis of the S-Cl bond would be below our LC-MS limit of detection.

Note: when the reaction solidified, the stir bar ceased functioning, but we found that adding solvent (DCM or toluene) during the initial reaction step significantly reduced yields in subsequent couplings.

***2',3'-O*-Isopropylidene-*5'-O*-sulfamoyladenosine (53):**

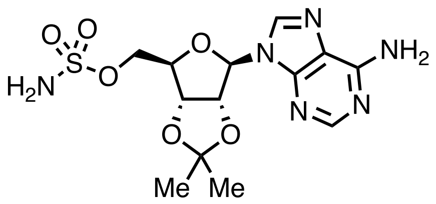

Synthesized as described by Van de Vijver et al.[3] In short, dissolved ((3aR,4R,6R,6aR)-6-(6-amino-9H-purin-9-yl)-2,2-dimethyltetrahydrofuro[3,4-d][1,3]dioxol-4-yl)methanol (880 mg, 2.86 mmol, 1 eq) in dimethylacetamide (DMAc; 4.4 mL) and cooled to 0°C. Separately dissolved sulfamoyl chloride (761 mg, 6.59 mmol, 2.3 eq) in MeCN (3 mL) and cooled to 0°C. Slowly added the MeCN solution to the DMAc solution. After 45 min, quenched the reaction by successive addition of triethylamine (3 mL) and MeOH (6 mL). Concentrated the reaction mixture *in vacuo* to ~4-5 mL. Diluted with EtOAc (100 mL), washed three times with 5% $LiCl_{(aq)}$ (50 mL), washed twice with half saturated $NaHCO_{3(aq)}$ (50 mL), washed twice with quarter saturated $NaCl_{(aq)}$ (50 mL), dried over anhydrous sodium sulfate, and concentrated *in vacuo* without heating to obtain **53**.

Yield: 522 mg, 47.2%. Flaky yellow-white solid.

^{1}H NMR, ^{13}C NMR, and LC-MS data all matched those from Heacock et al. [4]

Notes: adding DMAc or DMF to the sulfamoyl chloride solution in the absence of the alcohol will cause it to rapidly decompose.

5'-O-[N-(L-Prolyl)-sulfamoyl]adenosine (ProSA, 25):

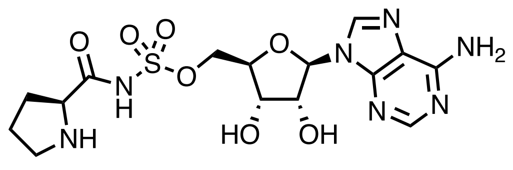

Synthesized as described by Van de Vijver et al. for similar molecules with minor modifications.[3] In short, a flask was charged with **53** (522 mg, 1.35 mmol, 1 eq), L-Boc-Pro-OSu (507 mg, 1.62 mmol, 1.2 eq), and DMF (13.5 mL). To this, added DBU (489 µL, 494 mg, 3.25 mmol, 2.4 eq) and stirred 25 min. The reaction mixture was successively diluted with water (27 mL) and then quarter saturated aqueous sodium citrate until pH = 9 before extracting five times with EtOAc. Washed pooled EtOAc twice with 5% $LiCl_{(aq)}$, twice with saturated $NH_4Cl_{(aq)}$, twice with half saturated $NaHCO_{3(aq)}$, twice with quarter saturated $NaCl_{(aq)}$, and dried over anhydrous sodium sulfate. LC-MS analysis indicated that much of the product remained in the sodium citrate and LiCl washes so these were pooled, and then anhydrous $Na_2SO_{4(s)}$ was added until saturated. Unsuccessfully tried to extract three times with EtOAc. To the pooled sodium citrate / LiCl fraction, added $NaCl_{(s)}$ until saturated. Extracted three times with iPrOH. Pooled iPrOH with both sets of EtOAc fractions and concentrated *in vacuo*. Purified by normal phase flash column chromatography and eluted with EtOAc/EtOH to obtain crude ***2',3'-O-Isopropylidene-5'-O-[N-(Boc-L-prolyl)-sulfamoyl]adenosine*** (160 mg, 20.3%) which was used without further purification or characterization.

To crude ***2',3'-O-Isopropylidene-5'-O-[N-(Boc-L-prolyl)-sulfamoyl]adenosine*** (160 mg, 0.275 mmol), added 5:1 TFA/water (2.4 mL) and stirred 30 min. Concentrated *in vacuo* and azeotroped three times with EtOH. Purified by reverse phase flash column chromatography and eluted with water/MeCN (no additives) to obtain **25** as a white solid.

^{1}H NMR (d_6-DMSO) and MS data all matched those from Heacock *et al.*[4]

^{13}C NMR (101 MHz, DMSO) δ 172.1*, 156.0, 152.7, 149.6, 139.4, 119.0, 87.1, 82.4, 73.4, 70.7, 67.7, 62.0, 45.4, 29.2, 23.4. *Resonance assigned based upon ^{1}H-^{13}C HMBC.

Note: Our ^{13}C NMR spectra (d_6-DMSO) matched the spectra of ProSA·Et_3N reported by Konno et al.[5], except our spectra did not include the Et_3N ^{13}C resonances (10.2 ppm and 44 ppm).

5'-O-[N-(D-Prolyl)-sulfamoyl]adenosine (D-ProSA, 27):

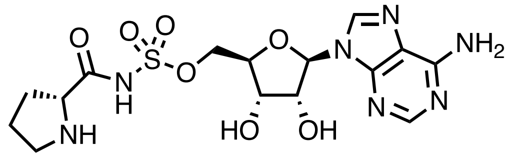

Synthesized as described by Heacock et al. from **53**.[4]

^{1}H NMR (D_2O) and LC-MS data matched what was reported Heacock et al. The ^{13}C NMR data was not reported by Heacock et al.

^{13}C NMR (101 MHz, D_2O) δ 175.3, 155.5, 152.9, 148.9, 139.7, 118.6, 87.4, 82.2, 74.0, 70.1, 68.5, 62.2, 46.2, 29.5, 23.6.

tert-butyl (S)-2-((1-((3-((2,3-dihydro-1H-inden-2-yl)carbamoyl)pyrazin-2-yl)carbamoyl)piperidin-4-yl)carbamoyl)pyrrolidine-1-carboxylate (31):

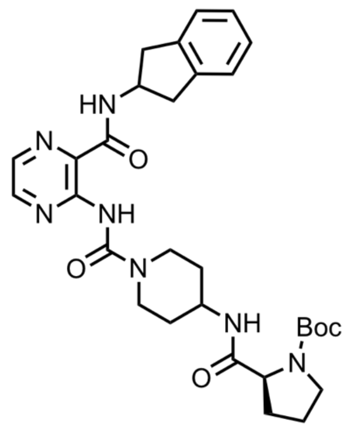

Charged a vial with **8·2HCl** (185 mg, 408 µmol, 1 eq), Boc-L-proline *N*-hydroxysuccinimide ester (199 mg, 636 µmol, 1.56 eq), DCM (5 mL), and DIPEA (400 µL, 297 mg, 2.30 mmol, 5.6 eq). Stirred 3 h. Concentrated reaction mixture *in vacuo* to a white solid. Purified by normal phase flash column chromatography (DCM/MeOH) to obtain **31**.

Yield: 216 mg, 91.4%. White solid.

^{1}H NMR (400 MHz, DMSO) δ 11.01 (s, 1H), 9.21 (d, *J* = 7.8 Hz, 1H), 8.50 (d, *J* = 2.4 Hz, 1H), 8.22 (d, *J* = 2.4 Hz, 1H), 7.91 – 7.74 (m, 1H), 7.28 – 7.18 (m, 2H), 7.19 – 7.09 (m, 2H), 4.72 (h, *J* = 7.6 Hz, 1H), 4.08 – 3.90 (m, 3H), 3.90 – 3.73 (m, 1H), 3.43 – 3.29 (m, 1H), 3.31 – 3.21 (m, 1H), 3.19 (dd, *J* = 15.8, 7.7 Hz, 2H), 3.04 (s, 4H), 2.16 – 2.00 (m, 1H), 1.87 – 1.66 (m, 5H), 1.50 – 1.24 (m, 11H).

^{13}C NMR (101 MHz, DMSO) δ 171.7, 165.7, 153.3, 152.2, 145.0, 145.7, 141.1, 135.5, 130.6, 126.5, 124.4, 78.5, 78.3, 59.7, 54.9, 50.4, 46.5, 45.7, 42.9, 38.4, 31.5, 31.4, 31.2, 28.1, 28.1, 23.2.

LC-MS ($C_{30}H_{39}N_7O_5$): Calculated $[M+H]^+$ m/z = 578.31, $[M-H]^-$ m/z = 576.29. Observed $[M+H]^+$ m/z = 578.63, $[M-H]^-$ m/z = 576.58.

(*S*)-*N*-(2,3-dihydro-1*H*-inden-2-yl)-3-(4-(pyrrolidine-2-carboxamido)piperidine-1-carboxamido)pyrazine-2-carboxamide (MAT334, 29):

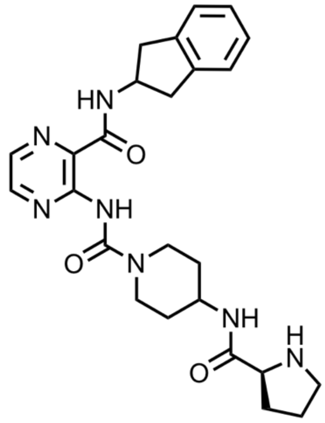

Diluted 4.0 M $HCl_{(1,4\text{-dioxane})}$ (1 mL) with methanol (3 mL) and stirred until mixture cooled to room temperature. Added this to a vial containing **31** (77.5 mg, 134 µmol, 1 eq) and stirred vigorously for 2.5 h. Concentrated the reaction mixture *in vacuo* to a light brown oil and purified by reverse phase flash column chromatography (water + 0.1% formic acid / MeCN + 0.1% formic acid). Concentrated product fractions *in vacuo* to dryness and azeotroped with methanol (2 mL) to obtain **MAT334**.

Yield: 57.1 mg, 89.1%. White solid.

^{1}H NMR (400 MHz, DMSO) δ 11.00 (s, 1H), 9.21 (d, *J* = 7.8 Hz, 1H), 8.50 (d, *J* = 2.5 Hz, 1H), 8.41 (d, *J* = 7.7 Hz, 1H), 8.28 (s, 1H), 8.22 (d, *J* = 2.4 Hz, 1H), 7.25 – 7.17 (m, 2H), 7.19 – 7.10 (m, 2H), 4.72 (h, *J* = 7.6 Hz, 1H), 3.98 (dd, *J* = 12.9, 4.8 Hz, 3H), 3.92 – 3.76 (m, 1H), 3.26 – 3.14 (m, 2H), 3.14 – 2.96 (m, 6H), 2.23 – 2.10 (m, 1H), 1.88 – 1.69 (m, 5H), 1.51 – 1.34 (m, 2H).

^{13}C NMR (101 MHz, DMSO) δ 169.4, 165.7, 152.3, 149.9, 145.7, 141.1, 135.6, 130.7, 126.5, 124.5, 59.1, 50.5, 46.0, 45.8, 42.6, 38.4, 31.2, 30.0, 24.3.

LC-MS ($C_{25}H_{31}N_7O_3$): Calculated $[M+H]^+$ m/z = 478.26, $[M-H]^-$ m/z = 476.24. Observed $[M+H]^+$ m/z = 478.55, $[M-H]^-$ m/z = 476.55.

***tert*-butyl (*S*)-2-(((1-((3-((2,3-dihydro-1*H*-inden-2-yl)carbamoyl)pyrazin-2-yl)carbamoyl)piperidin-4-yl)methyl)carbamoyl)pyrrolidine-1-carboxylate (32):**

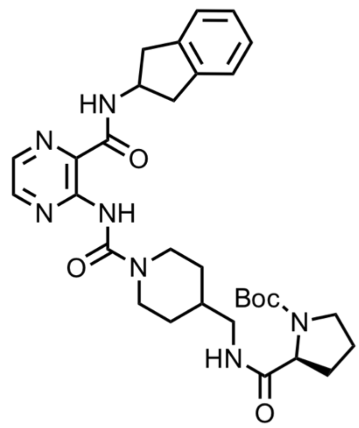

Charged a vial with **10** (141 mg, 356 μmol, 1 eq), Boc-L-proline *N*-hydroxysuccinimide ester (152 mg, 488 μmol, 1.37 eq), DCM (5 mL), and DIPEA (400 μL, 297 mg, 2.30 mmol, 6.45 eq). Stirred reaction mixture for 3 h, concentrated *in vacuo* to a white solid, and purified by normal phase flash column chromatography (DCM/MeOH) to obtain **32**.

Yield: 153 mg, 72.5%. White solid.

^{1}H NMR (400 MHz, DMSO) δ 11.02 (s, 1H), 9.21 (d, *J* = 7.8 Hz, 1H), 8.50 (d, *J* = 2.4 Hz, 1H), 8.21 (d, *J* = 2.3 Hz, 1H), 7.99 – 7.80 (m, 1H), 7.27 – 7.18 (m, 2H), 7.19 – 7.09 (m, 2H), 4.71 (h, *J* = 7.6 Hz, 1H), 4.13 – 3.95 (m, 3H), 3.44 – 3.29 (m, 1H), 3.26 (dt, *J* = 10.2, 6.8 Hz, 1H), 3.19 (dd, *J* = 15.8, 7.7 Hz, 2H), 3.11 – 3.00 (m, 3H), 2.98 – 2.80 (m, 3H), 2.15 – 1.99 (m, 1H), 1.87 – 1.61 (m, 6H), 1.35 (d, 9H), 1.20 – 1.02 (m, 2H).

^{13}C NMR (101 MHz, DMSO) δ 172.5, 172.2, 165.7, 153.4, 152.1, 150.1, 145.7, 141.1, 135.4, 130.3, 126.5, 124.5, 78.5, 78.4, 59.9, 50.5, 46.5, 43.7, 38.4, 36.0, 31.2, 30.7, 29.5, 28.1, 28.0, 24.0, 23.2.

LC-MS ($C_{31}H_{41}N_7O_5$): Calculated $[M+H]^+$ m/z = 592.32, $[M-H]^-$ m/z = 590.31. Observed $[M+H]^+$ m/z = 592.65, $[M-H]^-$ m/z = 590.60.

(*S*)-*N*-(2,3-dihydro-1*H*-inden-2-yl)-3-(4-((pyrrolidine-2-carboxamido)methyl)piperidine-1-carboxamido)pyrazine-2-carboxamide (MAT345, 30):

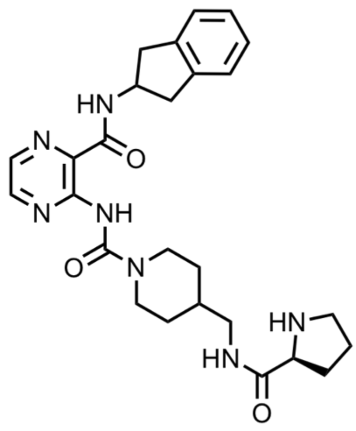

Diluted 4.0 M $HCl_{(1,4\text{-dioxane})}$ (1 mL) with methanol (3 mL) and stirred until mixture cooled to room temperature. Added this to a vial containing **32** (78.1 mg, 132 µmol, 1 eq) and stirred vigorously for 2.5 h. Concentrated reaction mixture *in vacuo* to a light brown oil and purified by reverse phase flash column chromatography (water + 0.1% formic acid / MeCN + 0.1% formic acid). Concentrated product fractions *in vacuo* to dryness and azeotroped with methanol (2 mL) to obtain **MAT345**.

Yield: 60.8 mg, 93.7%. White solid.

^{1}H NMR (400 MHz, DMSO) δ 11.02 (s, 1H), 9.22 (d, *J* = 7.8 Hz, 1H), 8.50 (d, *J* = 2.4 Hz, 1H), 8.34 (t, *J* = 5.9 Hz, 1H), 8.28 (s, 1H), 8.21 (d, *J* = 2.4 Hz, 1H), 7.25 – 7.18 (m, 2H), 7.18 – 7.11 (m, 2H), 4.71 (h, *J* = 7.6 Hz, 1H), 4.06 (d, *J* = 13.1 Hz, 2H), 3.93 – 3.82 (m, 1H), 3.19 (dd, *J* = 15.8, 7.7 Hz, 2H), 3.10 – 2.97 (m, 6H), 2.88 (t, *J* = 12.6 Hz, 2H), 2.19 – 2.03 (m, 1H), 1.82 – 1.60 (m, 6H), 1.19 – 1.05 (m, 2H).

^{13}C NMR (101 MHz, DMSO) δ 171.1, 165.7, 152.1, 150.1, 145.8, 141.1, 135.4, 130.3, 126.5, 124.5, 59.4, 50.5, 45.9, 43.8, 38.4, 35.8, 30.2, 29.4, 24.7.

LC-MS ($C_{26}H_{33}N_7O_3$): Calculated $[M+H]^+$ m/z = 492.27, $[M-H]^-$ m/z = 490.26. Observed $[M+H]^+$ m/z = 492.14, $[M-H]^-$ m/z = 490.56.

tert-butyl trans-2-(3-(7-bromo-6-chloro-4-oxoquinazolin-3(4*H*)-yl)-2-oxopropyl)-3-hydroxypiperidine-1-carboxylate (54):

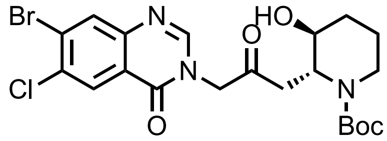

(Racemic trans)

To a suspension of trans-halofuginone (2.06 g, 4.96 mmol, 1 eq) in DMF (90 mL), successively added Boc_2O (1.44 g, 6.59 mmol, 1.32 eq) and DIPEA (2.50 mL, 1.86 g, 14.4 mmol, 2.90 eq). After 2 h, concentrated reaction mixture *in vacuo* to ~0.5 mL, diluted with EtOAc (600 mL),

washed twice with 0.2 M $HCl_{(aq)}$ (300 mL), washed twice with 5% $LiCl_{(aq)}$ (150 mL), and washed once with saturated $NaCl_{(aq)}$ (100 mL). Dried the organic fraction over anhydrous sodium sulfate, filtered, and concentrated *in vacuo*. Resuspended in methyl tert-butyl ether (MTBE; 5 mL), sonicated vigorously, and filtered to obtain **54**.

Yield: 2.48 g, >95%. White solid.

^{1}H NMR, ^{13}C NMR, and LC-MS data match the previously reported data from Linder et al.[6]

tert-butyl trans-2-(3-(7-bromo-6-chloro-4-oxoquinazolin-3(4*H*)-yl)-2-oxopropyl)-3-((((4-((3-((2,3-dihydro-1*H*-inden-2-yl)carbamoyl)pyrazin-2-yl)carbamoyl)piperazin-1-yl)sulfonyl)carbamoyl)oxy)piperidine-1-carboxylate (37):

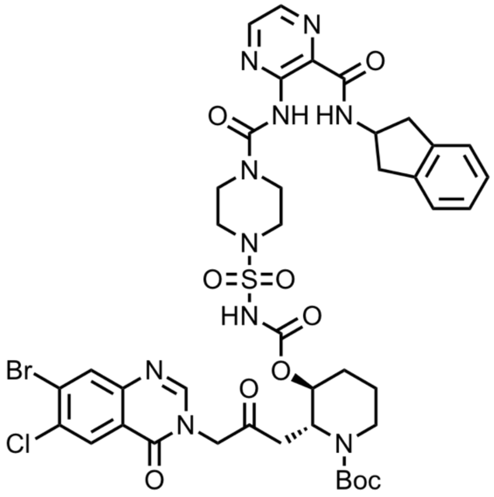

+en

To a stirred solution of **54** (206 mg, 401 μmol, 1 eq) in DCM (5 mL), added chlorosulfonyl isocyanate (37.1 μL, 60.5 mg, 427 μmol, 1.1 eq). After stirring reaction mixture for 10 min, added solution of **14·2HCl** (269 mg, 613 μmol, 1.53 eq), DIPEA (540 μL, 401 mg, 3.10 mmol, 7.74 eq), and DCM (5 mL). After reaction was complete by LC-MS (typically ~5 min), diluted reaction mixture with 0.2 M $HCl_{(aq)}$ (200 mL) and extracted twice with DCM (200 mL). Pooled DCM fractions were dried with anhydrous sodium sulfate, filtered, and concentrated *in vacuo*. Purified by normal phase flash column chromatography (DCM/MeOH) to obtain **37**.

Yield: 244 mg, 61.6%. White solid.

^{1}H NMR (400 MHz, DMSO) δ 11.47 (s, 1H), 11.09 – 10.84 (m, 1H), 9.15 (d, *J* = 7.6 Hz, 1H), 8.56 – 8.40 (m, 1H), 8.34 – 8.04 (m, 4H), 7.29 – 7.06 (m, 4H), 5.20 – 4.88 (m, 2H), 4.88 – 4.52 (m, 3H), 3.97 – 3.72 (m, 1H), 3.66 – 3.49 (m, 4H), 3.34 – 3.25 (m, 4H), 3.17 (dd, *J* = 15.8, 7.8 Hz, 2H), 3.12 – 2.65 (m, 5H), 1.90 – 1.55 (m, 3H), 1.48 – 1.27 (m, 10H).

^{13}C NMR (101 MHz, DMSO) δ 200.7, 165.5, 158.7, 154.6, 154.0, 152.6, 151.1, 149.6, 147.3, 147.3, 145.5, 141.1, 135.7, 132.4, 131.7, 131.4, 131.1, 128.4, 126.9, 126.5, 124.5, 121.8, 121.8, 79.3, 79.0, 71.5, 54.3, 54.0, 50.4, 49.6, 46.0, 43.5, 38.9*, 38.5, 38.4, 37.3, 28.1, 27.9, 23.2, 21.3, 19.2. *Assigned based upon DEPT-135 and ^{1}H-^{13}C HSQC.

LC-MS ($C_{41}H_{46}BrClN_{10}O_{10}S$): Calculated $[M+H]^+$ m/z = 985.20, $[M-H]^-$ m/z = 983.20. Observed $[M+H]^+$ m/z = 985.43, $[M-H]^-$ m/z = 983.45.

Note: ^{1}H and ^{13}C NMR spectra for this compound shows rotomers. All resonances observed are reported.

trans-2-(3-(7-bromo-6-chloro-4-oxoquinazolin-3(4*H*)-yl)-2-oxopropyl)piperidin-3-yl ((4-((3-((2,3-dihydro-1*H*-inden-2-yl)carbamoyl)pyrazin-2-yl)carbamoyl)piperazin-1-yl)sulfonyl)carbamate (MAT436, 34):

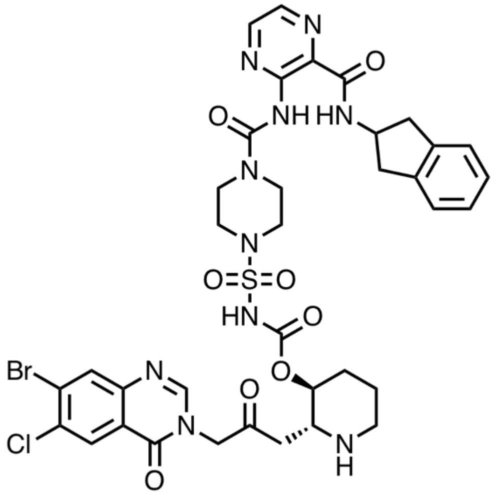

+en

To EtOH (792 µL), added 48% $HBr_{(aq)}$ (208 µL, 1.84 mmol, 160 eq) and stirred for 5 min. Added this to a vial of **37** (11.3 mg, 11.5 µmol, 1 eq) and stirred vigorously for 20 h. Concentrated reaction mixture *in vacuo,* azeotroped once with EtOH (2 mL), azeotroped once with 1:1 DCM/EtOH (4 mL), and then azeotroped twice with EtOH (2 mL). Triturated the resulting solid with EtOH (1 mL), sonicated vigorously, centrifuged to pellet the product, and decanted the supernatant. Dried the insoluble pellet *in vacuo* to obtain MAT436 as the monohydrobromide salt.

Yield: 7.2 mg, 71%. White solid.

^{1}H NMR (400 MHz, DMSO) δ 11.65 (s, 1H), 10.97 (s, 1H), 9.17 (d, *J* = 7.8 Hz, 1H), 8.97 – 8.71 (m, 2H), 8.47 (d, *J* = 2.4 Hz, 1H), 8.29 (s, 1H), 8.21 (d, *J* = 2.4 Hz, 1H), 8.18 (s, 1H), 8.14 (s, 1H), 7.28 – 7.09 (m, 4H), 5.10 – 4.94 (m, 2H), 4.76 – 4.60 (m, 2H), 3.75 (s, 1H), 3.64 – 3.54 (m, 4H), 3.34 – 3.29 (m, 4H), 3.24 – 3.11 (m, 4H), 3.11 – 2.95 (m, 4H), 2.15 – 2.02 (m, 1H), 1.89 – 1.77 (m, 1H), 1.75 – 1.57 (m, 2H).

^{13}C NMR (101 MHz, DMSO) δ 200.4, 165.5, 158.8, 152.5, 150.8, 149.6, 149.5, 147.2, 145.5, 141.1, 135.8, 132.4, 131.9, 131.0, 128.6, 126.9, 126.5, 124.5, 121.6, 71.0, 54.4, 52.5, 50.4, 46.0, 43.5, 42.8, 38.9*, 38.5, 38.4, 27.1, 19.8. *Assigned based upon DEPT-135 and ^{1}H-^{13}C HSQC.

LC-MS ($C_{36}H_{38}BrClN_{10}O_8S$): Calculated $[M+H]^+$ m/z = 885.15, $[M-H]^-$ m/z = 883.15. Observed $[M+H]^+$ m/z = 885.56, $[M-H]^-$ m/z = 883.54.

*Note: neat **MAT436·HBr** appears to be stable indefinitely at 4°C, but 10 mM stock solutions of **MAT436·HBr** in DMSO are not stable indefinitely at room temperature (LC-MS analysis after 8 days at room temperature indicated ~36% hydrolyzed at carbamate carbonyl) so we recommend long-term storage of **MAT436·HBr** stock solutions at -80°C.*

cis-2-(3-(7-bromo-6-chloro-4-oxoquinazolin-3(4*H*)-yl)-2-oxopropyl)piperidin-3-yl ((4-((3-((2,3-dihydro-1*H*-inden-2-yl)carbamoyl)pyrazin-2-yl)carbamoyl)piperazin-1-yl)sulfonyl)carbamate (*iso*-MAT436, 40):

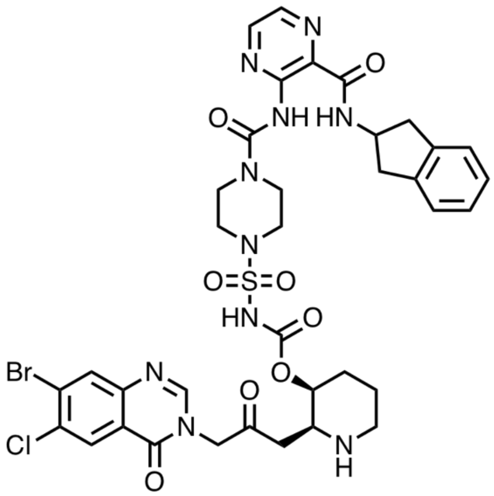

+en

Dissolved **37** (29.5 mg, 29.9 μmol) in a premixed solution of 5:1 TFA/water (1.2 mL) and stirred vigorously until reaction was complete by LC-MS (typically ~5 min). Concentrated reaction mixture *in vacuo* and then azeotroped three times with EtOH (2 mL) to obtain **MAT436·3TFA**. Purified by reverse phase flash column chromatography (water/MeCN, no buffering agent present) to obtain *iso*-MAT436.

Yield: 7.5 mg, 28.0%. White solid.

^{1}H NMR (400 MHz, DMSO) δ 11.01 (s, 1H), 9.20 (dd, *J* = 13.8, 7.8 Hz, 1H), 8.55 – 8.40 (m, 2H), 8.20 (d, *J* = 2.3 Hz, 1H), 8.16 (s, 1H), 8.09 (s, 1H), 7.31 – 7.08 (m, 4H), 5.30 – 4.85 (m, 2H), 4.77 – 4.61 (m, 1H), 4.51 – 4.35 (m, 1H), 3.61 – 3.47 (m, 4H), 3.18 (dt, *J* = 16.2, 8.3 Hz, 4H), 3.06 (dp, *J* = 16.7, 5.4 Hz, 7H), 2.89 (t, *J* = 12.1 Hz, 1H), 2.79 – 2.69 (m, 1H), 2.03 (q, *J* = 13.8 Hz, 1H), 1.79 (d, *J* = 13.5 Hz, 1H), 1.68 – 1.54 (m, 1H), 1.51 – 1.39 (m, 1H), 1.10 (s, 1H).

^{13}C NMR (101 MHz, DMSO) δ 200.6, 165.6, 158.7, 152.1, 150.0, 149.9, 149.7, 147.3, 145.8, 141.1, 141.1, 135.6, 132.5, 131.7, 130.1, 128.3, 126.8, 126.5, 124.5, 121.7, 70.3*, 55.5, 54.6, 50.4, 48.6*, 46.3, 45.8, 43.6, 43.4*, 40.2*, 38.5, 38.4, 28.9*, 26.9, 21.4*. *Assigned based upon DEPT-135 and ^{1}H-^{13}C HSQC.

LC-MS ($C_{36}H_{38}BrClN_{10}O_8S$): Calculated $[M+H]^+$ m/z = 885.15, $[M-H]^-$ m/z = 883.15. Observed $[M+H]^+$ m/z = 885.39, $[M-H]^-$ m/z = 883.31.

*Note: stereochemistry of the epimerizing carbon was assigned by comparison to NMR spectra of halofuginone and iso-halofuginone.[7] Based upon these spectra, we also ruled out the possibility that the iso-MAT436 (**40**) was isolated from deprotection of the cis-epimer of* **37***.*

tert-butyl trans-2-(3-(7-bromo-6-chloro-4-oxoquinazolin-3(4H)-yl)-2-oxopropyl)-3-(((N-((1-((3-((2,3-dihydro-1H-inden-2-yl)carbamoyl)pyrazin-2-yl)carbamoyl)piperidin-4-yl)methyl)sulfamoyl)carbamoyl)oxy)piperidine-1-carboxylate (38):

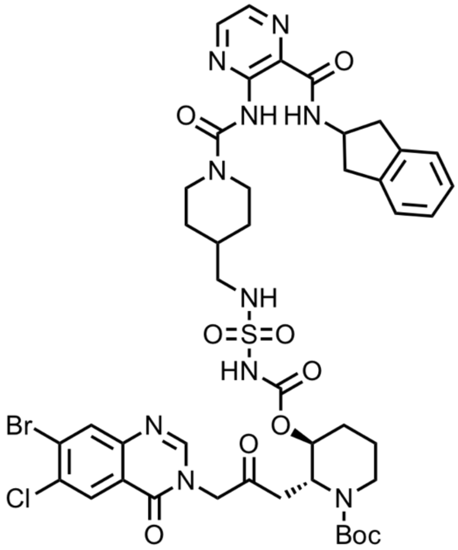

+en

To a stirred solution of **54** (100 mg, 195 μmol, 1 eq) in DCM (2.5 mL), added chlorosulfonyl isocyanate (18.6 μL, 30.3 mg, 214 μmol, 1.1 eq). After 10 min, added solution of **10** (120 mg, 303 μmol, 1.56 eq), DIPEA (540 μL, 401 mg, 3.10 mmol, 15.9 eq), and DCM (2.5 mL). After reaction was complete by LC-MS (typically ~5 min), diluted reaction mixture with 0.2 M $HCl_{(aq)}$ (100 mL) and extracted twice with DCM (100 mL). Pooled DCM fractions were dried with anhydrous sodium sulfate, filtered, and concentrated *in vacuo*. Purified by normal phase flash column chromatography (DCM/MeOH) to obtain **38**.

Yield: 69.9 mg, 35.4%. White solid.

^{1}H NMR (400 MHz, DMSO) δ 11.16 (s, 1H), 11.00 (s, 1H), 9.19 (d, *J* = 7.8 Hz, 1H), 8.48 (s, 1H), 8.31 – 8.16 (m, 3H), 8.11 (d, *J* = 8.7 Hz, 1H), 7.92 – 7.44 (m, 1H), 7.29 – 7.18 (m, 2H), 7.18 – 7.07 (m, 2H), 5.18 – 4.87 (m, 2H), 4.87 – 4.51 (m, 3H), 4.04 (d, *J* = 13.0 Hz, 2H), 3.96 – 3.69 (m, 1H), 3.26 – 2.70 (m, 11H), 1.95 – 1.81 (m, 1H), 1.81 – 1.66 (m, 4H), 1.63 (d, *J* = 13.3 Hz, 1H), 1.42 (d, *J* = 12.3 Hz, 1H), 1.36 (s, 9H), 1.11 (s, 2H).

^{13}C NMR (101 MHz, DMSO) δ 200.8, 165.7, 158.7, 154.8, 152.1, 150.0, 149.6, 147.3, 145.7, 141.1, 135.4, 132.4, 131.7, 130.2, 128.4, 126.9, 126.5, 124.5, 121.8, 79.4, 70.8, 54.2, 50.4, 49.7, 48.6, 48.1, 43.7, 39.1*, 38.4, 37.5, 35.4, 29.3, 28.1, 23.3, 19.2. *Assigned based upon DEPT-135 and ^{1}H-^{13}C HSQC.

LC-MS ($C_{43}H_{50}BrClN_{10}O_{10}S$): Calculated $[M+H]^{+}$ m/z = 1013.23, $[M-H]^{-}$ m/z = 1011.23. Observed $[M+H]^{+}$ m/z = 1013.41, $[M-H]^{-}$ m/z = 1011.51.

trans-2-(3-(7-bromo-6-chloro-4-oxoquinazolin-3(4*H*)-yl)-2-oxopropyl)piperidin-3-yl (*N*-((1-((3-((2,3-dihydro-1*H*-inden-2-yl)carbamoyl)pyrazin-2-yl)carbamoyl)piperidin-4-yl)methyl)sulfamoyl)carbamate (35):

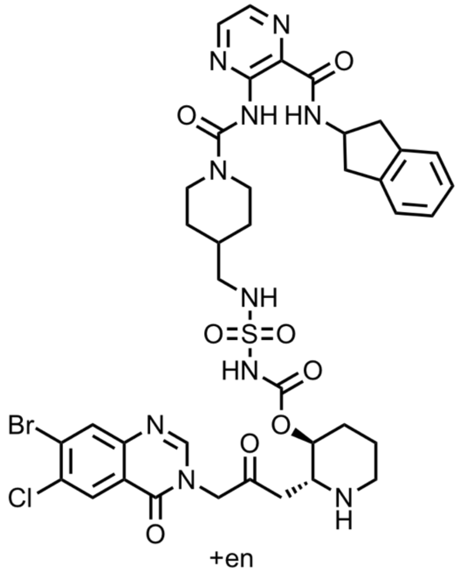

To EtOH (1.2 mL), added 48% $HBr_{(aq)}$ (312 µL, 2.76 mmol, 62.4 eq) and stirred for 5 min. Added this to a vial of **38** (44.8 mg, 44.2 µmol, 1 eq) and stirred vigorously for 24 h. Concentrated reaction mixture *in vacuo*, azeotroped once with EtOH (2 mL), azeotroped once with 1:1 DCM/EtOH (4 mL), and then azeotroped twice with EtOH (2 mL). Triturated the resulting solid with EtOH (1 mL), sonicated vigorously, centrifuged to pellet the product, and decanted supernatant. Dried the insoluble pellet *in vacuo* to obtain **35** as the monohydrobromide salt.

Yield: 36.7 mg, 83.5%. White solid.

^{1}H NMR (400 MHz, DMSO) δ 11.25 (s, 1H), 11.05 (s, 1H), 9.20 (d, *J* = 7.8 Hz, 1H), 9.13 – 8.90 (m, 2H), 8.48 (s, 1H), 8.35 (s, 1H), 8.24 – 8.17 (m, 2H), 8.15 (s, 1H), 8.00 (t, *J* = 5.9 Hz, 1H), 7.28 – 7.07 (m, 4H), 5.14 – 5.01 (m, 2H), 4.78 (td, *J* = 9.5, 4.2 Hz, 1H), 4.74 – 4.64 (m, 1H), 4.14 – 3.98 (m, 2H), 3.91 – 3.76 (m, 1H), 3.31 – 3.10 (m, 5H), 3.10 – 2.95 (m, 3H), 2.95 – 2.72 (m, 4H), 2.09 (dd, *J* = 10.7, 5.3 Hz, 1H), 1.96 – 1.83 (m, 1H), 1.83 – 1.60 (m, 5H), 1.19 – 1.02 (m, 2H).

^{13}C NMR (101 MHz, DMSO) δ 200.2, 165.6, 158.8, 152.1, 150.5, 145.0, 149.6, 147.3, 145.6, 141.1, 135.4, 132.5, 131.9, 130.2, 128.6, 126.9, 126.5, 126.5, 124.5, 121.7, 70.6, 54.4, 52.6, 50.4, 48.0, 43.7, 42.8, 39.1*,38.5, 35.4, 29.3, 27.2, 19.7. *Assigned based upon DEPT-135 and ^{1}H-^{13}C HSQC.

LC-MS ($C_{38}H_{42}BrClN_{10}O_8S$): Calculated $[M+H]^+$ m/z = 913.18, $[M-H]^-$ m/z = 911.18. Observed $[M+H]^+$ m/z = 913.58, $[M-H]^-$ m/z = 911.59.

*Note: based upon studies with the analogous **MAT436**, we suspect that this compound might be labile to epimerization if it is purified by reverse phase flash column chromatography (water / MeCN).*

tert-butyl trans-2-(3-(7-bromo-6-chloro-4-oxoquinazolin-3(4*H*)-yl)-2-oxopropyl)-3-(((*N*-(1-((3-((2,3-dihydro-1*H*-inden-2-yl)carbamoyl)pyrazin-2-yl)carbamoyl)piperidin-4-yl)sulfamoyl)carbamoyl)oxy)piperidine-1-carboxylate (39):

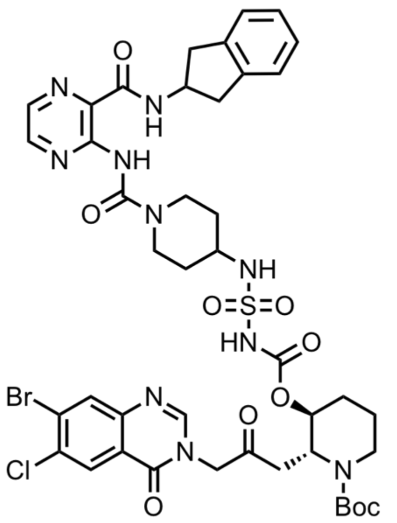

To a stirred solution of **54** (103 mg, 200 µmol, 1 eq) in DCM (2.5 mL), added chlorosulfonyl isocyanate (18.6 µL, 30.3 mg, 214 µmol, 1.1 eq). After stirring reaction mixture for 10 min, added solution of **8·2HCl** (134 mg, 296 µmol, 1.48 eq), DIPEA (540 µL, 401 mg, 3.10 mmol, 15.5 eq), and DCM (2.5 mL). After reaction was complete by LC-MS (typically ~5 min), diluted reaction mixture with 0.2 M $HCl_{(aq)}$ (100 mL) and extracted twice with DCM (100 mL). The pooled DCM fractions were dried with anhydrous sodium sulfate, filtered, and concentrated *in vacuo*. Purified by normal phase flash column chromatography (DCM/MeOH) to obtain **39**.

Yield: 82.0 mg, 41.1%. White solid.

^{1}H NMR (400 MHz, DMSO) δ 11.30 (s, 1H), 11.00 (s, 1H), 9.18 (d, *J* = 7.9 Hz, 1H), 9.13 – 8.89 (m, 2H), 8.48 (s, 1H), 8.36 (s, 1H), 8.23 – 8.18 (m, 1H), 8.18 – 8.15 (m, 1H), 8.15 – 8.09 (m, 2H), 7.25 – 7.08 (m, 4H), 5.15 – 4.99 (m, 2H), 4.79 (td, *J* = 9.4, 4.0 Hz, 1H), 4.70 – 4.64 (m, 1H), 3.96 (d, *J* = 13.2 Hz, 2H), 3.89 – 3.76 (m, 1H), 3.45 – 3.32 (m, 1H), 3.27 – 3.10 (m, 5H), 3.09 – 2.91 (m, 5H), 2.15 – 2.01 (m, 1H), 1.97 – 1.60 (m, 5H), 1.47 (h, *J* = 9.1 Hz, 2H).

^{13}C NMR (101 MHz, DMSO) δ 200.7, 165.6, 158.7, 154.8, 152.1, 145.0, 149.6, 147.3, 145.7, 141.1, 135.5, 132.4, 131.7, 130.3, 128.4, 126.9, 126.5, 124.4, 121.8, 79.4, 70.8, 54.1, 50.4, 49.8, 42.6, 42.4, 39.1*, 38.4, 38.4, 31.8, 31.3, 29.1, 28.1, 23.3, 22.1, 19.2, 14.0. *Assigned based upon DEPT-135 and ^{1}H-^{13}C HSQC.

LC-MS ($C_{42}H_{48}BrClN_{10}O_{10}S$): Calculated $[M+H]^+$ m/z = 999.21, $[M-H]^-$ m/z = 997.21. Observed $[M+H]^+$ m/z = 999.44, $[M-H]^-$ m/z = 997.27.

trans-2-(3-(7-bromo-6-chloro-4-oxoquinazolin-3(4*H*)-yl)-2-oxopropyl)piperidin-3-yl (*N*-(1-((3-((2,3-dihydro-1*H*-inden-2-yl)carbamoyl)pyrazin-2-yl)carbamoyl)piperidin-4-yl)sulfamoyl)carbamate (36):

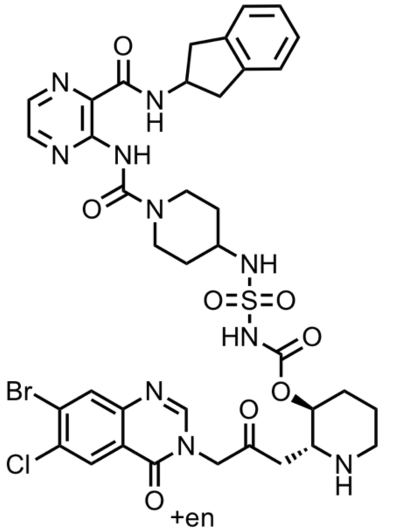

+en

To EtOH (1.2 mL), added 48% $HBr_{(aq)}$ (312 µL, 2.76 mmol, 47.0 eq) and stirred for 5 min. Added this to a vial of **39** (58.7 mg, 58.7 µmol, 1 eq) and stirred vigorously for 24 h. Concentrated the reaction mixture *in vacuo,* azeotroped once with EtOH (2 mL), azeotroped once with 1:1 DCM/EtOH (4 mL), and then azeotroped twice with EtOH (2 mL). Triturated the resulting solid with EtOH (1 mL), sonicated vigorously, centrifuged to pellet the product, and decanted supernatant. Dried the insoluble pellet *in vacuo* to obtain **36** as the monohydrobromide salt.

Yield: 54.2 mg, 94.1%. White solid.

^{1}H NMR (400 MHz, DMSO) δ 11.30 (s, 1H), 11.00 (s, 1H), 9.18 (d, *J* = 7.9 Hz, 1H), 9.13 – 8.89 (m, 2H), 8.48 (s, 1H), 8.36 (s, 1H), 8.23 – 8.18 (m, 1H), 8.18 – 8.15 (m, 1H), 8.15 – 8.09 (m, 2H), 7.25 – 7.08 (m, 4H), 5.15 – 4.99 (m, 2H), 4.79 (td, *J* = 9.4, 4.0 Hz, 1H), 4.70 – 4.64 (m, 1H), 3.96 (d, *J* = 13.2 Hz, 2H), 3.89 – 3.76 (m, 1H), 3.45 – 3.32 (m, 1H), 3.27 – 3.10 (m, 5H), 3.09 – 2.91 (m, 5H), 2.15 – 2.01 (m, 1H), 1.97 – 1.60 (m, 5H), 1.47 (h, *J* = 9.1 Hz, 2H).

^{13}C NMR (101 MHz, DMSO) δ 200.3, 165.6, 158.8, 152.1, 150.5, 149.8, 149.6, 147.2, 145.6, 141.1, 135.6, 132.4, 131.9, 130.5, 128.6, 126.9, 126.5, 124.5, 121.7, 70.6, 54.4, 52.7, 50.6, 50.4, 42.9, 42.6, 42.5, 39.0, 38.4, 31.9, 31.9, 27.2, 19.7.

LC-MS ($C_{37}H_{40}BrClN_{10}O_8S$): Calculated $[M+H]^+$ m/z = 899.16, $[M-H]^-$ m/z = 897.16. Observed $[M+H]^+$ m/z = 899.56, $[M-H]^-$ m/z = 897.56.

*Note: based upon studies with the analogous **MAT436**, we suspect that this compound might be labile to epimerization if it is purified by reverse phase flash column chromatography (water / MeCN).*

N-(2,3-dihydro-1H-inden-2-yl)-3-(methylamino)pyrazine-2-carboxamide (46):

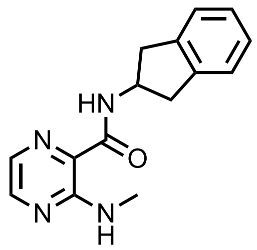

To a solution of **45** (25.0 mg, 98.3 µmol, 1 eq) in DMF (3 mL), successively added 1.0 M $NaHMDS_{(THF)}$ (200 µL, 197 µmol, 2 eq) and then a solution of methyl iodide (61.5 µL, 140 mg, 983 µmol, 10 eq) in DMF (0.5 mL). After 5 min, diluted the reaction mixture with DCM (100 mL), washed once with saturated $NH_4Cl_{(aq)}$ (100 mL), and washed twice with 1 M $HCl_{(aq)}$ (100 mL). Dried over anhydrous Na_2SO_4, filtered, and concentrated *in vacuo*. Purified by normal phase flash column chromatography (hexanes/EtOAc) to obtain **46**.

Yield: 8.6 mg, 32.6%. Yellow solid.

^{1}H NMR (400 MHz, Chloroform-*d*) δ 8.68 (s, 1H), 8.22 (d, *J* = 2.4 Hz, 1H), 8.14 (d, *J* = 8.0 Hz, 1H), 7.65 (d, *J* = 2.5 Hz, 1H), 7.31 (dd, *J* = 5.9, 3.1 Hz, 2H), 7.25 (dd, *J* = 5.4, 3.3 Hz, 2H), 4.89 (dtd, *J* = 12.7, 7.5, 5.2 Hz, 1H), 3.45 (dd, *J* = 16.1, 7.3 Hz, 2H), 3.10 (d, *J* = 5.0 Hz, 3H), 3.00 (dd, *J* = 16.1, 5.2 Hz, 2H).

^{13}C NMR (101 MHz, $CDCl_3$) δ 166.5, 155.4, 146.4, 141.0, 129.1, 127.0, 126.9, 124.9, 50.4, 40.2, 27.5.

LC-MS ($C_{15}H_{16}N_4O$): Calculated $[M+H]^+$ m/z = 269.13. Observed $[M+H]^+$ m/z = 269.37.

3-(2,3-dihydro-1H-inden-2-yl)pteridine-2,4(1H,3H)-dione (47):

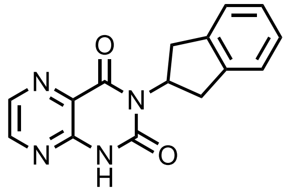

Synthesized using the general procedure described for synthesizing NCP26 modified to replace piperidine with water and all quantities scaled to 20 mg **45**. The reaction was allowed to proceed overnight before it was quenched with MeOH, concentrated *in vacuo*, and purified by reverse phase flash column chromatography (water + 0.1% formic acid / MeCN + 0.1% formic acid) to obtain **47**.

Yield: 18 mg, 83%.

^{1}H NMR (400 MHz, DMSO-d_6) δ 12.19 (s, 1H), 8.66 (s, 1H), 8.55 (d, *J* = 2.3 Hz, 1H), 7.26 – 7.19 (m, 2H), 7.19 – 7.13 (m, 2H), 5.76 (dd, *J* = 11.6, 6.5 Hz, 1H), 3.51 (dd, *J* = 15.9, 8.2 Hz, 2H), 3.12 (dd, *J* = 15.9, 9.8 Hz, 2H).

^{13}C NMR (101 MHz, DMSO-d_6) δ 160.7, 149.9, 148.3, 148.1, 141.4, 140.3, 127.6, 126.2, 124.3, 50.8, 35.0.

LC-MS ($C_{15}H_{12}N_4O_2$): Calculated $[M-H]^-$ m/z = 279.10. Observed $[M-H]^-$ m/z = 279.21.

3-amino-*N*-cyclohexylpyrazine-2-carboxamide (48):

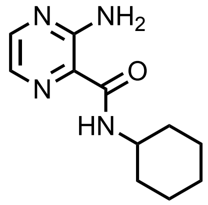

Suspended 3-aminopyrazine-2-carboxylic acid (205 mg, 1.44 mmol, 1 eq) in DCM (60 mL) and successively added cyclohexylamine (200 µL, 173 mg, 1.74 mmol, 1.2 eq), PyBOP (830 mg, 1.59 mmol, 1.1 eq), and DIPEA (750 µL, 4.31 mmol, 3 eq). After 72 h, diluted reaction mixture with DCM (200 mL), washed twice with 1 M $HCl_{(aq)}$ (200 mL), washed twice with saturated $NaHCO_{3(aq)}$ (200 mL), washed once with saturated $NaCl_{(aq)}$ (200 mL), dried over anhydrous Na_2SO_4, filtered, and concentrated *in vacuo*. Purified by normal phase flash column chromatography (1:1 hexanes/EtOAc) to obtain **48**.

Yield: 262 mg, 82.8%. Off-white solid.

^1H NMR (400 MHz, Chloroform-d) δ 8.11 (s, 1H), 7.78 (s, 2H), 3.98 – 3.81 (m, 1H), 1.98 (d, J = 12.0 Hz, 2H), 1.85 – 1.71 (m, 2H), 1.70 – 1.60 (m, 1H), 1.43 (q, J = 12.0 Hz, 2H), 1.36 – 1.17 (m, 3H).

^{13}C NMR (101 MHz, $CDCl_3$) δ 165.1, 155.1, 146.2, 131.6, 127.3, 48.2, 33.2, 25.7, 25.0.

LC-MS ($C_{11}H_{16}N_4O$): Calculated $[M+H]^+$ m/z = 221.13. Observed $[M+H]^+$ m/z = 221.37.

***N*-cyclohexyl-3-(2,3-dihydro-1*H*-indene-2-carboxamido)pyrazine-2-carboxamide (49):**

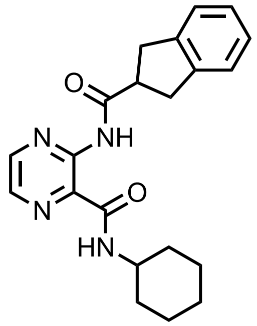

Suspended 2,3-dihydro-1*H*-indene-2-carboxylic acid (30 mg, 185 µmol, 1 eq) in THF (1 mL). Successively added a drop of DMF and then oxalyl chloride (16.2 µL, 23.5 mg, 185 µmol, 1 eq). Stirred reaction mixture for 15 min. While waiting, charged a second vial with **48** (50 mg, 220 µmol, 1.2 eq), THF (3 mL), and 1.0 M $NaHMDS_{(THF)}$ (0.55 mL, 0.56 mmol, 3 eq) to obtain a

cloudy yellow suspension. After 5 min, added the NaHMDS suspension to the first solution and stirred for 1 h. Diluted reaction mixture with DCM (100 mL), washed twice with 1 M $HCl_{(aq)}$ (100 mL), washed once with saturated $NaCl_{(aq)}$ (100 mL), dried over anhydrous Na_2SO_4, filtered, and concentrated *in vacuo*. Purified by normal phase flash column chromatography (hexanes/EtOAc) to obtain **49**.

Yield: 4.6 mg, 6.8%.

^{1}H NMR (400 MHz, Chloroform-*d*) δ 12.25 (s, 1H), 8.59 (d, *J* = 2.4 Hz, 1H), 8.19 (d, *J* = 2.4 Hz, 1H), 8.08 (d, *J* = 8.7 Hz, 1H), 7.23 (dd, *J* = 5.4, 3.4 Hz, 2H), 7.19 – 7.13 (m, 2H), 3.93 (tdd, *J* = 10.1, 7.2, 4.0 Hz, 1H), 3.56 (q, *J* = 8.7 Hz, 1H), 3.43 (dd, *J* = 15.7, 8.7 Hz, 2H), 3.32 (dd, *J* = 15.6, 8.8 Hz, 2H), 2.07 – 1.97 (m, 2H), 1.80 (dt, *J* = 13.2, 3.9 Hz, 2H), 1.72 – 1.63 (m, 1H), 1.51 – 1.21 (m, 4H).

^{13}C NMR (101 MHz, $CDCl_3$) δ 173.1, 164.5, 149.6, 146.4, 141.7, 136.5, 129.6, 126.7, 124.5, 48.6, 47.9, 36.3, 33.0, 25.6, 24.9.

LC-MS ($C_{21}H_{24}N_4O_2$): Calculated $[M+H]^+$ m/z = 365.19, $[M-H]^-$ m/z = 363.19. Observed $[M+H]^+$ m/z = 365.38, $[M-H]^-$ m/z = 363.22.

3-(cyclohexanesulfonamido)-*N*-(2,3-dihydro-1*H*-inden-2-yl)pyrazine-2-carboxamide (50):

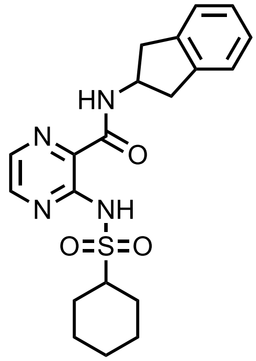

Dissolved **45** (50.5 mg, 199 μmol, 1 eq) in THF (4.6 mL) under $Ar_{(g)}$ atmosphere and added 1.0 M $NaHMDS_{(THF)}$ (600 μL, 600 μmol, 3 eq). Stirred for 15 min before adding this mixture dropwise over 5 min to a stirred solution of cyclohexanesulfonyl chloride (34.5 μL, 43.4 mg, 238 μmol, 1.2 eq) in THF (2 mL) under $Ar_{(g)}$ atmosphere. After 1 h, added more THF (2 mL) to the reaction and stirred reaction overnight under $Ar_{(g)}$ atmosphere. In the morning, the reaction was a cloudy, yellow suspension and LC-MS analysis indicated incomplete conversion to **50** (~10-15%). To the reaction mixture, added more 1.0 M $NaHMDS_{(THF)}$ (400 μL, 400 μmol, 2 eq) dropwise. The reaction mixture turned a cloudy orange but showed minimal additional conversion after 5 min by LC-MS analysis. To the reaction, added a solution of cyclohexanesulfonyl chloride (34.5 μL, 43.4 mg, 238 μmol, 1.2 eq) in THF (0.5 mL) dropwise and stirred for 3 h. Concentrated reaction mixture *in vacuo* to remove THF. The resulting solid was dissolved in DCM (30 mL), washed twice with 1 M $HCl_{(aq)}$ (30 mL), washed twice with water (30 mL), washed once with saturated $NaCl_{(aq)}$ (30 mL), dried over anhydrous Na_2SO_4, filtered,

and concentrated *in vacuo*. Purified by normal phase flash column chromatography (DCM/MeOH) to obtain **50**.

Yield: 12.1 mg, 15%.

^{1}H NMR (400 MHz, Chloroform-*d*) δ 11.36 (s, 1H), 8.43 (s, 1H), 8.27 – 8.02 (m, 2H), 7.29 – 7.24 (m, 2H), 7.24 – 7.16 (m, 2H), 4.87 (dq, *J* = 12.1, 7.6, 6.1 Hz, 1H), 3.73 (ddd, *J* = 12.1, 9.0, 3.2 Hz, 1H), 3.42 (dd, *J* = 16.2, 7.2 Hz, 2H), 2.96 (dd, *J* = 16.2, 4.7 Hz, 2H), 2.25 (d, *J* = 12.6 Hz, 2H), 1.91 (d, *J* = 10.9 Hz, 2H), 1.70 (q, *J* = 12.9, 12.4 Hz, 2H), 1.34 – 1.22 (m, 4H).

^{13}C NMR (101 MHz, $CDCl_3$) δ 164.8, 149.5, 145.8, 140.5, 136.0, 128.8, 127.0, 124.9, 62.1, 50.6, 40.0, 25.7, 25.2, 25.1.

LC-MS ($C_{20}H_{24}N_4O_3S$): Calculated $[M+H]^+$ m/z = 401.16, $[M-H]^-$ m/z = 399.16. Observed $[M+H]^+$ m/z = 401.33, $[M-H]^-$ m/z = 399.17.

Supplementary References

1. Pourcelle, V. et al. Functionalization of the PEG Corona of Nanoparticles by Clip Photochemistry in Water: Application to the Grafting of RGD Ligands on PEGylated USPIO Imaging Agent. *Bioconjug Chem* **26**, 822-829 (2015).
2. Kato, D.-i. et al. Enantiodifferentiation of ketoprofen by Japanese firefly luciferase from Luciola lateralis. *Journal of Molecular Catalysis B: Enzymatic* **69**, 140-146 (2011).
3. Van de Vijver, P. et al. Aminoacyl-tRNA synthetase inhibitors as potent and synergistic immunosuppressants. *J Med Chem* **51**, 3020-3029 (2008).
4. Heacock, D., Forsyth, C.J., Shiba, K. & Musier-Forsyth, K. Synthesis and Aminoacyl-tRNA Synthetase Inhibitory Activity of Prolyl Adenylate Analogs. *Bioorganic Chemistry* **24**, 273-289 (1996).
5. Konno, S. et al. Active site-directed proteomic probes for adenylation domains in nonribosomal peptide synthetases. *Chem Commun (Camb)* **51**, 2262-2265 (2015).
6. Linder, M.R. et al. (2R,3S)-(+)- and (2S,3R)-(-)-Halofuginone lactate: synthesis, absolute configuration, and activity against Cryptosporidium parvum. *Bioorg Med Chem Lett* **17**, 4140-4143 (2007).
7. Smullen, S. & Evans, P. An asymmetric synthesis of febrifugine, halofuginone and their hemiketal isomers. *Tetrahedron* **73**, 5493-5499 (2017).
