# Supplementary material for: Elucidating the path to Plasmodium prolyl-tRNA synthetase inhibitors that overcome halofuginone resistance
Source: Nat Commun. 2022 Aug 25;13:4976. doi: 10.1038/s41467-022-32630-4 (PMC9403976; doi:10.1038/s41467-022-32630-4)
Supplement: Supplementary file 3 — Reporting Summary [file 41467_2022_32630_MOESM3_ESM.pdf]

nature portfolio

Corresponding author(s): Ralph Mazitschek

Last updated by author(s): Jul 19, 2022

Reporting Summary

Nature Portfolio wishes to improve the reproducibility of the work that we publish. This form provides structure for consistency and transparency in reporting. For further information on Nature Portfolio policies, see our Editorial Policies and the Editorial Policy Checklist.

Statistics

For all statistical analyses, confirm that the following items are present in the figure legend, table legend, main text, or Methods section.

n/a	Confirmed	
☐	☒	The exact sample size (n) for each experimental group/condition, given as a discrete number and unit of measurement
☐	☒	A statement on whether measurements were taken from distinct samples or whether the same sample was measured repeatedly
☒	☐	The statistical test(s) used AND whether they are one- or two-sided<br>*Only common tests should be described solely by name; describe more complex techniques in the Methods section.*
☒	☐	A description of all covariates tested
☒	☐	A description of any assumptions or corrections, such as tests of normality and adjustment for multiple comparisons
☐	☒	A full description of the statistical parameters including central tendency (e.g. means) or other basic estimates (e.g. regression coefficient) AND variation (e.g. standard deviation) or associated estimates of uncertainty (e.g. confidence intervals)
☒	☐	For null hypothesis testing, the test statistic (e.g. F, t, r) with confidence intervals, effect sizes, degrees of freedom and P value noted<br>*Give P values as exact values whenever suitable.*
☒	☐	For Bayesian analysis, information on the choice of priors and Markov chain Monte Carlo settings
☒	☐	For hierarchical and complex designs, identification of the appropriate level for tests and full reporting of outcomes
☐	☒	Estimates of effect sizes (e.g. Cohen's d, Pearson's r), indicating how they were calculated

Our web collection on statistics for biologists contains articles on many of the points above.

Software and code

Policy information about availability of computer code

Data collection	NMR data were acquired on a Bruker Avance III 400 MHz spectrometer running Topspin 3.2<br>LCMS data were acquired using MassLynx V4.1 SCN 846<br>Fluorescence gel imaging data was acquired using Amersham Typhoon software (Version 1.0.0.7, Cytiva Life Sciences)<br>Protein and nucleic acid concentrations were acquired using Nanodrop 1000 software (Version 3.8.1, ThermoFisher)<br>TR-FRET microplate reader experiments were acquired using SPARKCONTROL software (Version V2.1, Tecan Group Ltd.) in Excel (Version 16.30, Microsoft)<br>Preliminary docking was done with Cresset Biomolecular Discovery Ltd's Spark v10.5.0, Forge v10.5.0, and Flare 4.0.2.<br>Asexual blood stage growth growth assay microplate reader experiments were acquired on a Molecular Devices SpectraMax iD5.<br>P. berghei liver stage assay microplate reader experiments were acquired on Envision plate reader using Envision Manager Software (Perkin Elmer)<br>Protein x-ray crystallography data was collected at the Diamond Light Source, UK.
Data analysis	NMR spectra were analyzed using Mnova (Version 12.0.1-20560 Mestrelab Research)<br>LCMS data were analyzed using MassLynx (Version 4.1 SCN 846, Waters Cooperation)<br>Fluorescent gel images were analyzed and quantified in ImageJ (Version 1.440, National Institutes of Health)<br>Data was plotted and statistically analyzed using Prism (Versions 8.4.3 - 9.1.0, GraphPad Software)<br>Protein x-ray crystallography datasets were processed, scaled, and merged at the Diamon Light Source using Xia2. Electron density maps were obtained by molecular replacement using PHASER with previously determined structures of PfcProRS as a search model. Structures were refined in an iterative process using PHENIX (1.19.2-4158) with electron density map inspections and model improvement in WinCOOT (0.9.4.1). Structural analysis and figure generation was performed with PyMOL 2.5.2.

For manuscripts utilizing custom algorithms or software that are central to the research but not yet described in published literature, software must be made available to editors and reviewers. We strongly encourage code deposition in a community repository (e.g. GitHub). See the Nature Portfolio guidelines for submitting code & software for further information.

Data

Policy information about availability of data

All manuscripts must include a data availability statement. This statement should provide the following information, where applicable:

- Accession codes, unique identifiers, or web links for publicly available datasets
- A description of any restrictions on data availability
- For clinical datasets or third party data, please ensure that the statement adheres to our policy

The authors declare that the main data supporting the findings of this study are available within the article, its Supplementary Information files, and the Source Data files. Extra data (raw plate reader data for biological experiments; LC/MS data files) are available from the corresponding author upon request.

Field-specific reporting

Please select the one below that is the best fit for your research. If you are not sure, read the appropriate sections before making your selection.

[x] Life sciences [ ] Behavioural & social sciences [ ] Ecological, evolutionary & environmental sciences

For a reference copy of the document with all sections, see nature.com/documents/nr-reporting-summary-flat.pdf

Life sciences study design

All studies must disclose on these points even when the disclosure is negative.

Sample size	Sample sizes were not predetermined using statistical analyses. The minimum sample sizes were determined by magnitude and consistency of measurable differences and selected based on prior experiences. When practical considerations permitted, e.g. for TR-FRET dissociation rate measurements, some experiments were preformed using a larger number of replicate measurements than required. Unless otherwise indicated, all presented data are representative results for at least two experiments that were performed independently on different days. Information on the number of replicates and independent experiments that were performed for each measurement is provided in the manuscript.
Data exclusions	For some dose-titration experiments all replicates for high- and low-end samples were excluded when irrelevant for analysis and/or when compounds at high concentrations caused assay interference. Furthermore, individual outliers were excluded from graphing in instances with << 0% or >> 100% activity. Finally, values were excluded in the case of obvious dispensing errors far away from the curve's inflection point. Exclusion of these outliers did not impact the EC50 or KD measurements obtained by non-linear regression analysis. Applicable figure panels: Fig. 2c, Supplementary Information Fig. 2a,e, Supplementary Information Fig. 16a, Table 1, and Supplementary Table 1.
Replication	Replicates in standard biochemical and in vitro assays were performed to monitor the performance of the assay and not for hypothesis testing. All assays were conducted with established positive and negative controls as reference standards. All conditions were tested with n ≥ 2 replicates (see individual experiments for specific number of biological replicates / independent replicate wells) and, unless otherwise noted, are representative results for at least two experiments that were performed independently on different days and that successfully replicated the presented results. The liver stage P. berghei and HuH7 data in figure 3l are presented as mean ± s.d. of 3 biological replicates. The only samples measured with one biological replicate were in the P. falciparum asexual blood stage growth assay for compound 31 in each of the three strains (Dd2-2D4, halofuginone-induced, and HFGR-I) and for compounds 8, 13, and 14 in Dd2-2D4 only. Applicable figure panels: Fig. 3c, Supplementary Information Fig. 2d, Table 1, and Supplementary Table 1. None of the compounds that were tested in only one biological replicate was meaningful for the outcome of this study.
Randomization	Randomization was not applicable to this study.<br>Biochemical and lysate assays were performed in microtiter plate format. Reagents were dispensed using automated equipment and data were acquired using a plate reader. Independent control experiments are routinely performed to ensure uniformity of all used equipment.<br>In vitro cell-based assays were performed by splitting cells from the same flask to individual wells for treatments without bias. If more than one flask/well was required, cells were combined prior to plating to ensure sample homogeneity.<br>Photophysical characterization was performed independently on cuvette based instruments with automated data acquisition. We followed the manufacturer's recommendation to ensured that the test equipment had stabilized prior to data acquisition and by repeated testing of reference samples. There is no reason to assume that the order of acquisition of test samples would be prone to bias.<br>NMR and LC/MS data collections. These experiments were performed independently on automated equipment. There is no reason to assume that these analytical methods are prone to bias.
Blinding	Blinding was not applicable to this study. All experimental data were acquired using automated equipment and analyzed using computational softwares, eliminating human error and bias.

Reporting for specific materials, systems and methods

We require information from authors about some types of materials, experimental systems and methods used in many studies. Here, indicate whether each material, system or method listed is relevant to your study. If you are not sure if a list item applies to your research, read the appropriate section before selecting a response.

Materials & experimental systems

n/a	Involved in the study
☐	☒ Antibodies
☐	☒ Eukaryotic cell lines
☒	☐ Palaeontology and archaeology
☒	☐ Animals and other organisms
☒	☐ Human research participants
☒	☐ Clinical data
☒	☐ Dual use research of concern

Methods

n/a	Involved in the study
☒	☐ ChIP-seq
☒	☐ Flow cytometry
☒	☐ MRI-based neuroimaging

Antibodies

Antibodies used

anti-6xHis; 18184, Abcam, Lot GR3349062-1

Validation

The antibody used in this study is widely used and has been validated by the manufacturer (https://www.abcam.com/primary-antibodies/how-we-validate-our-antibodies). Furthermore, as we reported previously, we have experimentally measured the binding affinity of the antibody for the epitope and verified that the absence of epitope-tagged protein did not result in signal (https://www.nature.com/articles/s41589-021-00877-5).

Eukaryotic cell lines

Policy information about cell lines

Cell line source(s)

P. falciparum Dd2-2D4 is derived from Dd2 (Bei Resources Malaria Research and Reference Reagent Resource Center MRA-156) and was previously isolated by dilutional cloning.
P. falciparum halofuginone-induced and HFGR-I are derivatives of Dd2 produced in our previous studies (PMID: 25395010 and PMID: 25995223).
Human blood was purchased from Interstate Blood Bank.
Human HuH7-D12 cells were purchased from Sigma (Cat#: 01042712, Lot:18H009).
P. berghei ANKA (University of Georgia-SporoCore)

Authentication

The original Dd2 parent line was purchased from Bei Resources Malaria Research and Reference Reagent Resource Center. Dd2 cell lines used in this study (Dd2-2D4, halofuginone-induced, and HFGR-I) have previously undergone whole genome sequencing in our labs. They were also routinely assayed and EC50s determined using the asexual blood stage growth assay with a panel of standard antimalarial compounds including halofuginone which has differential activity for the three Dd2 lines used in these experiments. Data is cross-referenced for consistency with literature reported values and data previously acquired in the lab.

Blood was tested for blood borne pathogens including human immunodeficiency virus (HIV), hepatitis C virus (HCV), and hepatitis B virus (HBV).

Human HuH7 cells were purchased from Sigma (Cat#: 01042712, Lot:18H009) and used without further authentication.

P. berghei ANKA parasites have previously undergone RNA sequencing in our lab. Liver stage and HuH7 drug assays were performed using atovaquone as a control.

Mycoplasma contamination

Human HuH7 cells tested negative for mycoplasma within 2 months before and after experiments.

Blood and P. falciparum cell lines were not routinely tested for mycoplasma.

Commonly misidentified lines
(See ICLAC register)

No commonly misidentified cell lines were used in this study.
